# Discovery and application of insertion-deletion (INDEL) polymorphisms for QTL mapping of early life-history traits in Atlantic salmon

**DOI:** 10.1186/1471-2164-11-156

**Published:** 2010-03-08

**Authors:** Anti Vasemägi, Riho Gross, Daniel Palm, Tiit Paaver, Craig R Primmer

**Affiliations:** 1 Department of Biology, 20014, University of Turku, Finland; 2 Department of Aquaculture, Institute of Veterinary Medicine and Animal Science, Estonian University of Life Sciences, 51014 Tartu, Estonia; 3 Department of Wildlife, Fish, and Environmental Studies, Swedish University of Agricultural Sciences, SE-901 83 Umeå, Sweden

## Abstract

**Background:**

For decades, linkage mapping has been one of the most powerful and widely used approaches for elucidating the genetic architecture of phenotypic traits of medical, agricultural and evolutionary importance. However, successful mapping of Mendelian and quantitative phenotypic traits depends critically on the availability of fast and preferably high-throughput genotyping platforms. Several array-based single nucleotide polymorphism (SNP) genotyping platforms have been developed for genetic model organisms during recent years but most of these methods become prohibitively expensive for screening large numbers of individuals. Therefore, inexpensive, simple and flexible genotyping solutions that enable rapid screening of intermediate numbers of loci (~75-300) in hundreds to thousands of individuals are still needed for QTL mapping applications in a broad range of organisms.

**Results:**

Here we describe the discovery of and application of insertion-deletion (INDEL) polymorphisms for cost-efficient medium throughput genotyping that enables analysis of >75 loci in a single automated sequencer electrophoresis column with standard laboratory equipment. Genotyping of INDELs requires low start-up costs, includes few standard sample handling steps and is applicable to a broad range of species for which expressed sequence tag (EST) collections are available. As a proof of principle, we generated a partial INDEL linkage map in Atlantic salmon (*Salmo salar*) and rapidly identified a number of quantitative trait loci (QTLs) affecting early life-history traits that are expected to have important fitness consequences in the natural environment.

**Conclusions:**

The INDEL genotyping enabled fast coarse-mapping of chromosomal regions containing QTL, thus providing an efficient means for characterization of genetic architecture in multiple crosses and large pedigrees. This enables not only the discovery of larger number of QTLs with relatively smaller phenotypic effect but also provides a cost-effective means for evaluation of the frequency of segregating QTLs in outbred populations which is important for further understanding how genetic variation underlying phenotypic traits is maintained in the wild.

## Background

Despite the growing number of sequenced genomes, our knowledge of genetic variants that underlie phenotypic differences is far from complete. For several decades, linkage mapping has been one of the most powerful and popular approaches to study the genetic architecture of phenotypic traits. However, successful mapping of both Mendelian and complex traits depends critically on the availability of fast, cost-effective and high-throughput genotyping platform. During recent years, significant breakthroughs in developing high-throughput array-based single nucleotide polymorphism (SNP) genotyping assays for model organisms have been achieved which allow screening of thousands of loci in a highly parallel fashion [1-3]. However, the vast majority of array-based SNP genotyping approaches are not available for non-model species and become prohibitively expensive for screening large numbers of individuals which is commonly required for dissecting of the molecular genetic basis of phenotypic traits. This represents one of the major drawback for quantitative trait locus (QTL) mapping as the power of detecting QTL and the accuracy of estimating QTL effects depends critically on analyses of large number of individuals [4,5]. For example, simulation studies have shown that with sample sizes considerably lower than 500, the power to map QTL of small effect (<5%) is very low and the estimated magnitude of a QTL will be seriously exaggerated [5,6]. On the other hand, increasing marker density beyond 10 cM which usually corresponds to 50 to 200 markers depending on the organism does not provide any considerable increase in power [7,8]. Taken together, inexpensive, simple and flexible genotyping solutions that enable rapid screening of hundreds to thousands of individuals for intermediate numbers of loci (~75-300) would be extremely useful for QTL mapping applications in a broad range of organisms. Such a need is still inadequately met with currently available open-source and commercial genotyping platforms as they require expensive, highly specific laboratory equipment (e.g. array-based SNP genotyping platforms) and/or suffer high initial costs because of the use of long (SNPWave™,) or modified primers (e.g. TaqMan, SNP-SCALE) [9,10].

In contrast to SNPs, other types of genetic variation such as insertion-deletion (INDEL) polymorphisms have received more attention only recently [11-14]. This is surprising as INDELs are relatively abundant, spread throughout the genome, and contribute substantially to both intra- and interspecific divergence [14-18]. Insertion and deletions of single base pairs and monomeric base pair extensions of various lengths are the most common class of INDELs while other types of INDELs including transposon insertions and apparently random DNA sequences appear in lower frequencies [14,15,17]. The latter category, consisting of short (2-10 bp) apparently random DNA insertions-deletions are amenable for fast and cost-effective genotyping as such length variation is similar in form to microsatellite length polymorphisms, but showing no stutter. However, such INDELs have thus far not been fully utilized to develop high-throughput genotyping assays.

Atlantic salmon (*Salmo salar*) is an ideal species for demonstrating the suitability of INDEL genotyping for QTL mapping of ecologically important traits due to the availability of large number of expressed sequence tags (ESTs), high fecundity enabling generation of large QTL mapping families and the availability of extensive ecological knowledge. It exhibits a complex anadromous life cycle: juveniles typically spend one or more years in fresh water before migrating to the sea and subsequently return to fresh water as adults to spawn. In the natural environment, however, the vast majority of fertilized salmonid eggs die during early life-stages as eggs, fry, alevins or parr. Recapture rates suggest that in Atlantic salmon up to 83.5% mortality may occur during the first four months after emergence from the gravel, and highest morality occurs during very short period after emergence [19]. Hence, natural selection is expected to have a strong effect on phenotypic traits expressed during early life-stages. Such traits are considered to have a prominent role in adaptation as it affects juvenile competitive ability, dispersal, foraging, and vulnerability to predation and climatic conditions (e.g. [20]). Nevertheless, the underlying genetic basis of ecologically relevant early life-history traits, such as emergence from gravel and size of fry in Atlantic salmon, is currently unknown.

Here, we describe the discovery of and application of insertion-deletion (INDEL) polymorphisms for QTL mapping of ecologically important traits in Atlantic salmon. As a proof of principle, we generated partial INDEL linkage map and demonstrate rapid identification of a number of QTLs affecting early life-history traits in salmon that are expected to have important fitness consequences in natural environment.

## Results

### INDEL discovery from expressed sequence tags (ESTs)

Clustering of 431,073 Atlantic salmon ESTs resulted in 185,615 singletons and 34,311 contigs with an average size 1,072 bp. More than half of the contigs (53%) contained less than four sequences while 43% of contigs contained 4 to 30 sequences. Only 4% of contigs contained more than 30 sequences. Altogether, AutoSNP identified 6,189 INDELs which corresponds to the average INDEL density of one indel per 5,948 bp (1.68 × 10^-4 ^per bp). Further inspection of the dataset revealed that a significant proportion of identified INDELs contain short repeat motifs, as well as 1 bp mononucleotide insertion-deletions (data not shown).

### Development and the performance of 76 locus single-run INDEL panel

**Figure 1:**
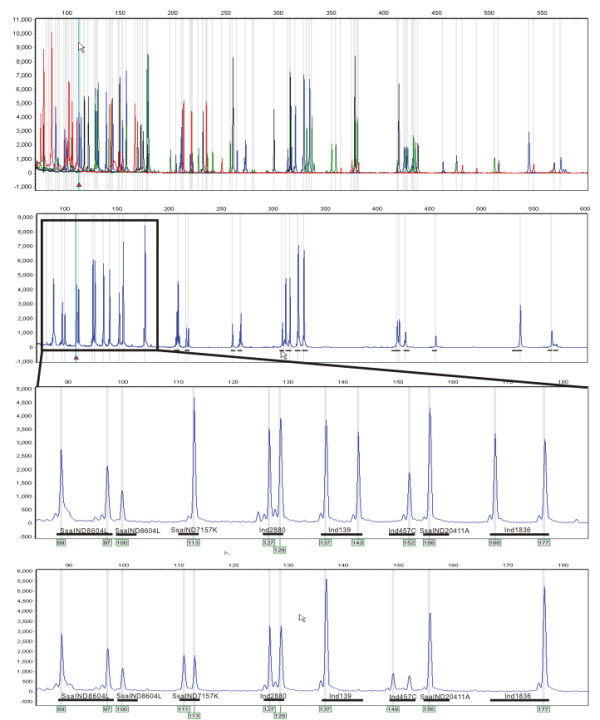
**Electropherogram of the 76 locus single-run INDEL panel in Atlantic salmon**. Upper row corresponds to electropherogram labeled with four different fluorescent tags, three single color (FAM) electropherograms with the enlarged region ranging from 90 to 180 bp consisting of eight INDEL markers are presented below.

Initial screening of 202 INDEL markers in 16 Atlantic salmon individuals from a broad geographical distribution revealed 120 polymorphic loci. Among these, six INDELs were predicted to change the length of the protein (Additional file 1, 2) based on GENSCAN prediction [21]. We subsequently combined up to 12 loci in a single multiplex amplification reaction and developed, without extensive optimization (see Methods), an efficient 76 locus single-run INDEL genotyping panel in Atlantic salmon (Fig. 1; Additional file 2). This simple approach consists of just three basic laboratory steps: i) eight multiplex PCR reactions with M13 tailed primers [22]; ii) pooling of PCR products; iii) capillary electrophoresis. This enables generation of 7,296 genotypes (76 loci × 96 individuals) within a single electrophoresis run which is comparable to the state-of the art array-based SNP genotyping platforms such as fluorescent tag-array mini-sequencing (TAMS) assays in *Drosophila melanogaster *that are able to produce 9,600 genotypes (120 loci × 80 individuals) on a single array [23]. The estimated proportion of loci that lead to high-quality assay of INDEL assay was 63%, since 76 loci giving high quality genotypes out of 120 polymorphic loci were successfully incorporated to INDEL genotyping panel. Based on repeated genotyping of 93 individuals from the R. Selja salmon population, the average calling rate (the proportion of genotypes called) over 56 polymorphic loci was 0.96 (>90% of individuals genotyped in 51 loci). We detected 24 genotype mismatches out of 4783 genotype calls which corresponds to the error rate 0.0049 (0.995 accuracy). Inconsistent genotype calls were detected in six loci out of 56 variable INDELs and in most cases the errors were caused by miscalling apparently heterozygous individual as homozygous.

### Construction of partial INDEL linkage map in Atlantic salmon

From 76 genotyped INDEL markers, 50 loci were polymorphic in at least one of two families and were used to construct INDEL linkage map together with 77 variable microsatellite loci (Additional file 3). The total number of segregating markers in family 1 and 2 was 147 and 139, respectively. Altogether, male and female maps consisted of 23 known and 5 unknown linkage groups (marked as X), and 6 unlinked markers (Additional file 4). INDELs were mapped to 21 linkage groups (up to 6 markers per LG) while two remained unlinked. This corresponds to most but not all chromosomes in Atlantic salmon as the common karyotype in Europe contains 29 linkage groups (2n = 58 [24]). As expected, a considerable proportion of the genome showed very low recombination in males while some regions exhibited similar or even higher levels of recombination in males [25]. This resulted in a shorter linkage map in males compared to females (male and female map lengths: 353 cM and 401 cM; 154 cM and 482 cM in family 1 and 2, respectively). Compared to the ASalBase female microsatellite map consisting of ca 700 markers http://www.asalbase.org the length of the corresponding linkage groups of the initial INDEL map was smaller in most cases, indicating that the coverage of the present INDEL map is still rather low. However, in some cases the length of linkage groups (INDEL map, AS-32, 42.4 cM) exceeded ASalBase map (14.6 cM) (Additional file 4).

### Mapping ecologically relevant early life-history QTLs in Atlantic salmon

**Figure 2:**
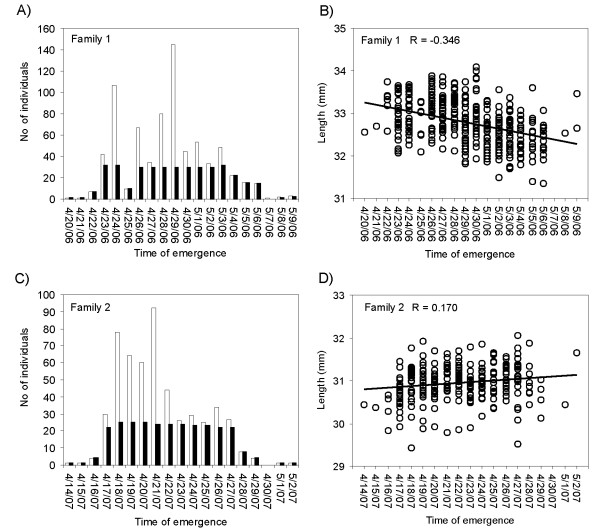
**Measured early life-history traits in Atlantic salmon**. The relationship between time of emergence (ToE) and individual fork length (FL) in family 1 (A, B) and 2 (C, D). White bars correspond to all individuals measured for ToE; black bars correspond to individuals chosen for QTL analyses.

The 76 locus INDEL panel was utilized together with microsatellite markers to identify for a first time QTLs for two ecologically relevant early life-history traits in two full-sib families (Fig. 2). A total of 33 QTL were detected at 5% chromosome-wide significance level (9 QTL at 1% chromosome-wide level), 15 (3) QTL for time of emergence (ToE) and 18 (6) QTLs for fork length (FL) (Table 1. Additional file 5). We expect to observe approximately twelve false positives at the 5% and two false positive QTLs at the 1% chromosome-wide significance level given that the total number of LGs/unlinked markers tested was 113 per trait. Individual QTL explained 5-12% and 5-16% of phenotypic variance for ToE (sire/dam effect range: 1-1.5 days) and FL (sire/dam effect range: 0.11-0.24 mm), respectively. However, due to selective genotyping of the ends of the distribution, the calculated QTL effects are most likely inflated. The total number of QTLs identified from family 1 was 23 (n = 372) while eleven QTLs were detected in family 2 (n = 279). Estimated 95% confidence intervals for QTL positions covered the whole linkage groups, most likely due to low recombination rate in males and relatively moderate number of markers per chromosome. Altogether five QTLs for a particular trait were identified in more than one segregating parent or family (AS-1, AS-12, AS-25, AS-32, X10). In seven cases QTLs for ToE were also associated with FL (AS-5, AS-7, AS-12, AS-14, AS-23, AS-32 and X8). However, when QTL analysis for ToE was executed considering length as covariate, only four ToE QTLs out of seven remained significant at the 5% chromosome-wide level (AS-5, AS-12, AS-23 and X8).

**Table 1:** Detected QTLs sorted by trait (ToE - time of emergence: FL - fork length), family, parent and the proportion of phenotypic variation explained (PVE).

Trait	Family	Mapping parent	LG	QTL position (cM)	PVE	*F*-value			95% C.I. of QTL position (cM)	Markers in region
								
						Obs	5% threshold	1% threshold		
ToE	1	♀	AS-28	0	0.09	8.87**	3.93	7.52		*Omm1134*
ToE	1	♀	AS-23	0	0.09	8.54*	5.24	8.73	0.0 - 12.0	*BHMS7-043, Ssa124, SSf43, 2456V*
ToE	1	♀	AS-14	0	0.07	6.37*	4.14	7.54		*2571c*
ToE	1	♀	X5	0	0.06	5.24*	4.96	8.66	0.0 - 25.0	*EST46, 1309C*
ToE	1	♀	AS-21	0	0.05	4.84*	3.96	6.97		*EST105*
ToE	1	♂	X8	0	0.13	12**	4.31	7.46	0.0 - 18.0	*190S, 8396P, EST127*
ToE	1	♂	AS-12	0	0.12	11.3**	4.02	7.60		*Omm1070*
ToE	1	♂	AS-32	0	0.08	7.91*	5.03	9.48	0.0 - 9.0	*EST44, 1445a, Ssa419UOS*
ToE	1	♂	AS-7	34	0.08	7.78*	4.98	7.78		*BHMS269, SSsp2216*
ToE	1	♂	AS-11	0	0.07	6.78*	3.87	7.04		*Sleel53, EST6, Omm1121, 16424E, Ssa417UOS, EST41*
ToE	1	♂	X12	0	0.06	5.3*	3.70	6.47		*EST70*
ToE	2	♀	AS-4	40	0.1	6.79*	5.21	8.12	0.0 - 40.0	*HSP, 11005 M, OMM1105*
ToE	2	♀	AS-25	1	0.1	6.88*	4.45	7.59	0.0 - 22.0	*2136E, Ssa4DIAS, 4493F*
ToE	2	♂	AS-5	15	0.1	7*	4.90	7.69	0.0 - 35.0	*BHMS7-017, 4151e, EST9, Ind2130, SSsp2201*
ToE	2	♂	AS-25	2	0.08	5.77*	4.01	6.31		*SsaIND2136E, Ssa4DIAS, Ssleer15.1*
FL	1	♀	AS-15	9	0.16	14.8**	4.33	8.23	0.0 - 17.0	*MHCI, 2273K*
FL	1	♀	AS-1	33	0.06	5.5*	4.74	7.58	0.0 - 33.0	*Ssa406UOS, 11971N*
FL	1	♀	AS-23	0	0.06	5.22*	5.12	8.45	0.0 - 9.0	*BHMS7-043, Ssa124, SSf43, 2456V*
FL	1	♀	X10	8	0.05	4.86*	4.20	7.13	0.0 - 8.0	*EST11, 7157K*
FL	1	♀	AS-33	0	0.05	4.58*	4.04	6.90		*BHMS144*
FL	1	♂	X9	0	0.12	11.7**	3.81	6.33		*EST101, Ind1921, 4868 M, 1271X, EST103*
FL	1	♂	X8	18	0.10	9.25**	4.80	8.02	0.0 - 18.0	*190S, 8396P, EST127*
FL	1	♂	X10	0	0.09	7.98**	3.84	7.27	0.0 - 4.0	*EST11, 7157K*
FL	1	♂	AS-12	0	0.08	7.87**	3.81	5.90		*Omm1070*
FL	1	♂	AS-1	7	0.08	7.09*	5.53	8.34	0.0 - 25.0	*EST115, Ssa406UOS, 2044 M*
FL	1	♂	AS-32	0	0.07	6.82*	5.24	8.09	0.0 - 9.0	*EST44,1445a, Ssa419UOS*
FL	1	♂	AS-9	5	0.05	4.52*	4.26	6.74	0.0 - 5.0	*32c, 17300F, BHMS189, EST141, Ind139, Ssosl438*
FL	2	♀	AS-13	27	0.09	6.23*	5.36	10.37	0.0 - 29.0	*Ssosl25, 9552C, EST74, Ssa289*
FL	2	♀	AS-10	7	0.09	6.01*	5.88	8.95	0.0 - 51.0	*CTAX, 8570Q, EST58, EST19, Ind457C, 13066I, Ssosl85, EST107*
FL	2	♀	AS-14	1	0.09	6.00*	4.28	7.04		*2571c, BHMS311*
FL	2	♂	AS-5	30	0.13	9.89**	5.02	8.68	0.0 - 35.0	*BHMS7-017, 4151e, EST9, Ind2130, SSsp2201*
FL	2	♂	AS-32	1	0.08	5.58*	4.27	6.15		*EST44, 4955H*
FL	2	♂	AS-12	2	0.07	4.83*	4.15	7.81		*Omy272UOG, OmyRGT13TUF*

Bold *F*-values indicate values larger than 5% genome-wide significance level threshold. Underlined linkage groups (LG) correspond to the QTL identified more than once in four mapping parents for particular trait. * significant at 5% chromosome-wide level, ** significant at 1% chromosome-wide level.

## Discussion

### Advances and limitations of INDEL genotyping for QTL mapping

We have demonstrated that insertions-deletions can be effectively utilized for QTL mapping applications in non-model organisms and INDELs can serve as useful alternatives for SNP and microsatellite markers, especially for characterization of genetic architecture in multiple crosses and large pedigrees. In the following, we discuss the advances and limitations of INDEL genotyping compared to the alternative existing genotyping methodologies. In terms of number of loci screened, currently available commercial ultra-high SNP genotyping platforms enable typing of orders of magnitude larger number of loci but generally provide rather low sample throughput, while traditional approaches enable genotyping of high numbers of individuals at limited number of loci. The INDEL genotyping strategy descried here falls between these two extremes and has several advantages, as well as limitations, compared to currently available microsatellite and SNP genotyping approaches. First, INDELs are more easily transferable between populations compared to microsatellites and applicable for a wide range of species for which expressed sequence tag collections for *in silico *INDEL identification are available. For example, at the time of writing over 160 species have more than 50 000 ESTs in NCBI EST database. In addition, new massively parallel sequencing technologies provide an extremely fast means to identify large numbers of INDELs [26]. Nevertheless, the frequency of INDELs is expected to be lower compared to SNPs and thus, the development of INDEL assay would require larger number of sequences than development of alternative genotyping approaches that are based on SNPs [10]. Second, genotyping of INDELs is relatively simple and compatible with 384-well format sample processing. This enables rapid screening of large number of samples as it is possible for one person to set up eight amplification reactions and run 384 individuals within a day. Such throughput means that for many species and traits analyzed in a linkage mapping framework sample throughput need not be the primarily limiting factor. However, genotype calling still requires a significant amount of time and effort, although considerably less than for standard microsatellite loci. Also, increasing the number of loci would be extremely useful as only a subset of biallelic markers are segregating in particular cross or family. For example, in the present study, only 50 markers out of 76 (66%) were segregating in the two Atlantic salmon families used for QTL mapping. Third, genotyping INDELs is cost-effective compared to many SNP genotyping approaches that require highly specific laboratory equipment and/or expensive primers [9,10], as the utilization of the tailed primer system [22] enables use of a single fluorescence labelled oligonucleotide for tagging large numbers of individual loci. This allows considerable reductions in primer cost compared to commonly used 5'-end fluorescence labelling of individual oligos. It is also rather flexible, as it is possible to freely change the fluorescence label of particular INDEL which enables easy selection of large number of non-overlapping markers. However, using a universal fluorescent oligonucleotide in addition to locus specific tailed primers complicates the PCR optimization procedure as increasing the concentration of the locus-specific primers does not necessarily result in higher amplification intensity of the fluorescently labeled PCR product. As a result, incorporation of new markers into existing genotyping panels and developing new multiplexes requires re-optimization to find optimal primer concentrations. However, it is also likely that further increases of multiplexing level can be achieved either via simultaneous use of different tailed primers [27], two phase amplification [28] or selective circulation methods [29]. The commercial multiplex PCR chemistry (QIAGEN) used in INDEL genotyping is also more expensive than standard PCR reagents but the extra reagent cost is compensated for by multiplexing up to 12 loci and using small volume reactions. The cost of running a single 76 locus INDEL assay, from which a maximum of 7,296 genotypes (or 4,800 genotypes, assuming that ca 50 loci are segregating in particular QTL mapping study) can be obtained, is currently ~220 USD in our laboratory (~0.03 - 0.046) cents per genotype, including 8 PCRs and capillary electrophoresis). When the cost of 76 unlabeled and four fluorescently labeled M13 primers are included to the calculations, the estimated cost of genotyping 76 loci in 1000 individuals is ~0.05 cents per genotype. Fourth, in contrast to the most array-based genotyping assays, INDEL genotyping using standard electrophoresis procedure does not require specific laboratory equipment or generation of specific libraries and arrays (e.g. [23,30]) making it attractive for laboratories with standard fragment analysis laboratory equipment.

Compared to microsatellite and SNP assays, genotyping of INDELs- is most similar to Multiplex SNP-SCALE [9,10] which also utilizes tailed primer system [22] to reduce primer cost, QIAGEN PCR chemistry for multiplexing and capillary electrophoresis for separation of alleles of different size. However, the largest difference between INDEL genotyping and SNP-SCALE [9,10] is that the latter uses three locus-specific primers to discriminate between alternative SNP alleles (two allele-specific modified oligos as forward primers and unmodified reverse primer). Hence, the initial cost of primers for SNP-SCALE [9,10] is 50% higher compared to INDELs as initial amplification of INDELs requires only two locus-specific unmodified primers. In addition, finding suitable allele-specific primers for SNP allele discrimination is more challenging than designing standard primers flanking particular INDEL. On the other hand, we expect that the calling rate (the proportion of genotypes called) and genotyping error rate for both methods is relatively similar as both approaches are using PCR multiplexing followed by electrophoresis for locus and allele discrimination. It is more difficult to compare the conversion rates of different methods (the proportion of loci that lead to high-quality assay) but reported marker conversion rates for SNP genotyping approaches often range from 50% to 86% [9]. Hence, the estimated conversion rate for the INDEL assay (63%) is comparable with SNP genotyping methods.

### QTLmapping of early life-history traits in Atlantic salmon

To our knowledge, this is the first report of quantitative trait loci affecting time of emergence (ToE) and length during the critical period of shifting from endogenous to exogenous energy supplies in Atlantic salmon [19]. Earlier studies have identified several QTLs that influence size in salmonids at older life stages [31,32]. Compared to the present study, the same linkage groups were identified to harbor QTLs in several cases but it is not clear whether these shared size-related QTLs correspond to the same or separate loci. As the physiological energy conversion mechanisms using endogenous versus exogenous energy supplies are different in fish one might expect a rather different set of genes affecting growth before and after the start of active feeding [33]. As expected, we detected more QTLs when using the male as a mapping parent as a result of reduced recombination compared to females, consistent with the other QTL studies in salmonids (e.g. [31,32,34]). Also, in many instances, several markers showed lack of recombination in males, while in females, the same markers mapped 30-50 cM away from each other. On the other hand, we also observed that in some cases markers appeared to be unlinked in males but were closely linked in female map. These results are accordance with earlier studies in Atlantic salmon that demonstrate the lack of recombination in some genomic regions in males while in other regions, male recombination rates are very high relative to female recombination rates [31,32]. Taken together, low recombination rate over large genomic regions in males enable initial QTL mapping with relatively few loci in Atlantic salmon but this also complicates the estimation of the position and effect of QTL. Consequently, finer-scale localization of QTL in salmon is more feasible from female side using larger number of markers.

Previously, analyses of selection differentials in the natural environment have demonstrated strong directional selection on time of emergence and length at the beginning of exogenous feeding in Atlantic salmon. For example, EINUM and FLEMING [19] showed that the delay of emergence of one standard deviation (SD) resulted in a 39% increase in mortality, while 1 SD decrease in length at emergence resulted in a 25% increase in mortality during a 17 day period in the natural environment. When using these standardized linear selection gradient estimates (*β*) in the context of calculated sire or dam effects, the largest QTL for ToE could increase or decrease the mortality 10-17% while largest QTL for length can affect the mortality rate from 5 to 11%. However, as noted earlier, the calculated QTL effects of may be inflated, but nevertheless, it suggests that given the evidence of strong natural selection combined with large family sizes in salmonids [19,35] it should be feasible to carry out genome-wide screens for identification of the genomic regions affecting survival in natural environment using linkage mapping framework [36]. Compared to analysis of candidate loci such as major histocompatibility (MH) linked genes [37] this would represent a significant step forward and new genetic tools, such as described here, open up new possibilities for further dissection of the genetic basis of phenotypic variation, adaptation and fitness in natural environment [38,39].

## Conclusions

In summary, INDEL genotyping enables fast coarse mapping in large numbers of individuals and families/crosses, thus providing an efficient means for more comprehensive characterization of genetic architecture in multiple crosses and large pedigrees. As such, it may help to answer some essential questions in the evolutionary genetic research, like: To what extent the same QTL are segregating in multiple populations? How many QTLs are affecting fitness related traits in natural populations? We expect that the insertion-deletion polymorphisms can be a valuable marker resource for addressing these and related questions in an increasing number of species.

## Methods

### INDEL discovery and initial polymorphism screen

In total, 431,073 publicly available Atlantic salmon expressed sequence tags (ESTs) were screened for INDELs using the redundancy-based approach with a modified version of autoSNP program [40,41] kindly provided by the authors. AutoSNP uses the TGICL clustering tool [42] and CAP3 [43] with 98% identity criterion to generate alignment data.

Altogether 202 primers pairs were designed using Primer3 software (v. 0.4.0) with the default parameters to amplify 90-580 bp DNA fragments containing 2 to 11 bp INDELs using the M13 tailed primer approach [22]. Loci were screened for polymorphism in 16 individuals from European (River Burrishoole, Ireland and River Narva, Estonia) and North-American (New Brunswick aquaculture strain originating from St. John River, Canada) Atlantic salmon populations.

### Development of INDEL genotyping assay

After the initial polymorphism screen, eight groups of INDELs each consisting 12-14 loci were randomly pooled together according on the fragment sizes (e.g. multiplexes consisting of fragments of 90-250 bp or 250-550 bp length) to minimize unequal amplification rates that depend on fragment length. The first multiplex amplifications were carried out using equal concentration of locus specific forward and reverse primers (0.2 μM each) to screen eight individuals. Large proportion of loci were successfully amplified during the initial multiplex PCR (7-12 loci per multiplex reaction) but in order to further increase the signal strength and adjust the peak intensities of amplified fragments, loci were classified into four categories, corresponding to strong, medium, weak and very weak amplification class. Subsequently, depending on amplification intensity, the following locus specific forward and reverse primer concentrations were used for each category: strong (0.033 and 0.125 μM), medium (0.05 and 0.2 μM), weak (0.05 and 0.3 μM) and very weak amplification (0.075 and 0.3 μM of forward and reverse primer, respectively) (Additional file 2). Loci that did not amplify during the first multiplex PCR were added to alternative multiplex reactions and their amplification success was tested subsequently. After the optimization procedure described above, the final set of loci consisted of 76 INDELs that were successfully multiplexed in eight separate amplification reactions consisting of 8- to 12 INDELs in each multiplex (Additional file 2). Fifty four polymorphic loci were left out from the INDEL genotyping assay either because of overlapping size ranges or weak-failed amplification in the multiplex reaction.

All reactions were carried out in 6 μL reaction volume including ca 10-100 ng of DNA, 0.033-0.3 μM of locus specific forward and reverse primer (Additional file 2), 4 μM of the M13 primer labeled with one of four fluorescent dyes (PET, FAM, NED, or VIC), and 1 × QIAGEN multiplex PCR master mix. The PCR program started with a 15-min initial activation step at 95°C followed by 15 cycles of denaturation at 94°C for 30 s, annealing at 58°C for 90 s and extension at 72°C for 60 s, and 25 cycles of denaturation at 94°C for 30 s, annealing at 52°C for 90 s and extension at 72°C for 60 s. The protocol ended with a final extension at 60°C for 15 min. Amplifications were performed on Applied Bioystems 2720, PTC-100 or PTC-200 (MJ Research) thermal cyclers. The PCR products (1 or 2.5 μL) from eight separate multiplex reactions, containing in total 76 loci, were pooled in 200 μL of distilled water (Additional file 2) and mixed with GS600LIZ size standard (Applied Biosystems) and formamide for a single electrophoresis run on an ABI 3130 × l automated sequencer. In order to estimate the error rate and calling rate (proportion of individuals receiving a genotype) of the INDEL genotyping assay, 93 Atlantic salmon originating from the R. Selja (Estonia) were amplified and genotyped twice.

### Microsatellite genotyping

To incorporate INDELs to the existing linkage map in Atlantic salmon, 77 microsatellite markers were genotyped (Additional file 3). The majority of microsatellite markers were chosen from the Atlantic salmon composite linkage map http://www.asalbase.org. Twenty five EST-derived microsatellite markers [44] had not been previously mapped. GenBank accession numbers of the markers and primer sequences are available in Supplemental Material (Additional file 4). PCR conditions and post-PCR pooling information for 25 markers used by Vähä et al. [45] are available at http://users.utu.fi/jpvaha/. Primer concentrations, PCR conditions and post-PCR pooling information for other microsatellites are available in Supplemental Material (Additional file 6). Microsatellite electrophoresis was performed on an ABI 3130 × l automated sequencer (Applied Biosystems). Both microsatellite and INDEL genotyping was performed with GeneMarker v. 1.6 (Softgenetics) followed by manual corrections.

### Mapping families and measured traits

The fish used to produce F_1 _families originated from the River Narva (Gulf of Finland, the Baltic Sea, Estonia, 59°25'17.63"N; 28° 8'12.53"E) outbred Atlantic salmon population. R. Narva hatchery population has been created by mixing salmon of River Neva (Russia) origin with the fish originating from the rivers flowing to the Gulf of Riga (Latvia) during the 1960s and the stock has been sustained by artificial reproduction since then. Two large F_1 _full sib families were produced to ensure reasonable statistical power for within-family linkage analysis in autumn 2005 (family 1) and 2006 (family 2). Two juvenile traits that have been shown to be under strong natural selection [19] were measured. The first trait, time of emergence (i.e. the time when fry leaves the gravel and starts exogenous feeding; ToE) was measured as described in PALM *et al*. [45]. Shortly, newly hatched salmon fry were placed to polyvinylchloride containers (26 cm long and 10 cm diameter) with two compartments: the lower part filled with natural gravel (diameter 10-30 mm) connected with the upper part where emerged fish can swim freely. The containers were placed to 1.5 m diameter fish tanks in Põlula Fish Rearing Centre, Estonia and ToE was monitored daily from January till the end of the experiment in May. The water temperature during the experiment followed natural fluctuations and increased from ca 4-6°C in January to 8-11°C in the beginning of May. The period of active emergence started at 710 and 850 degree-days and ended at 883 and 983 degree-days in 2006 and 2007, respectively. For QTL analyses, the start of the active emergence was designated as day one. The second trait, fork length (FL) was measured from photographs taken at the time of emergence using ImageJ software [46].

ToE was measured in 741 and 589 fish from family 1 and 2, respectively. Individuals for QTL mapping were selected preferably from the tails of the emergence time distribution in order to increase the power of identification QTLs for ToE, a procedure known as selective genotyping [47]. Thus only 370 and 279 individuals were selected for genotyping from family 1 and 2, respectively. The mean, standard deviation and range (*R*) for two traits were: family 1 (ToE = 9.85 ± 3.87 days, *R *= 1-17; FL = 32.79 ± 0.53 mm, *R *= 31.04-34.09) and family 2 (ToE = 10.88 ± 3.52 days, *R *= 1-19; FL = 30.97 ± 0.42 mm, *R *= 29.43-32.05). In family 1, a negative correlation between the two traits was observed (Spearman's *r*_S _= -0.383, *P *< 10^-6^) while in family 2 there was a weak positive correlation between the traits (Spearman's *r*_S _= 0.156, *P *< 0.01) (Fig. 2). The Box-Cox transformation [48] was used to determine the optimal transformation for trait 1 that deviated from the normal distribution, resulting in approximately normally distributed data. Total DNA was extracted from the fin clips according to LAIRD *et al*. [49].

### Construction of genetic linkage map

Since recombination frequency in salmonid fishes differs considerably between sexes (e.g. [25,31,32]), separate male and female maps were constructed based on segregation data from two full-sib families consisting of 50 segregating INDEL and 77 microsatellite markers using the software package LINKMFEX v.2.3 developed by R. G. Danzmann http://www.uoguelph.ca/~rdanzman/. Module LINKMFEX was used for pairwise recombination estimation and module MAPORD was used to determine the linear order of markers within a linkage group (minimum LOD score set to 4). Map distances were calculated using the Kosambi function with the module MAPDIS. Linkage groups were assigned according to the SALMAP linkage map (AS-1 to AS-33) using microsatellites to infer homologies http://www.asalbase.org. Linkage groups that did not share any markers with SALMAP map were marked as X. Segregation distortion was tested using log-likelihood ratio tests for goodness of fit to Mendelian expectations using the module SEGsort (data not shown).

### QTL mapping

QTL analyses in two F_1 _full-sib families were performed using a regression based approach [50] implemented in the software package QTLexpress half-sib (HS) module [51]. A single-QTL model was used and the analysis was performed at every 1 cM. Because the two traits were significantly correlated with each other, analysis was conducted with and without length as a covariate. The proportion of phenotypic variance explained (PVE) by the QTL was calculated as 4(1-MS_full_/MS_reduced_) where MS_full _corresponds to the mean residual square of the model including the QTL, and MS_reduced _is the mean residual square of the model fitting only a family mean [50]. However, due to the preferential sampling of the ends of the distribution (selective genotyping), the calculated QTL effects may be inflated. When only a single marker was segregating in a linkage group, a particular marker was duplicated and the presence of QTL was tested as a fixed location (S. Knott, pers. comm.). Chromosome- and genome-wide significance thresholds at the 5% and 1% level were determined using 2000 permutations implemented in QTL express [52]. When it was possible to construct combined maps from two parents (10 and 8 linkage groups in males and females, respectively: Additional file 4), QTL analyses was also performed by combining the independent tests from the separate families into an overall test statistic. However, the results from the merged dataset were highly similar to the single family analyses (data not shown) and therefore, we present only results where two families are treated separately.

## Authors' contributions

AV conceived and coordinated the study, carried out the molecular analyses, performed the data analyses and wrote the first draft of the manuscript with contributions from RG, DP, TP and CRP. All authors took part in the planning of the study, and read and improved the manuscript.

## Supplementary Material

Additional file 1 Information on 202 INDELs tested in Atlantic salmon containing GenBank accession numbers, primer sequences, observed sizes of fragments and BLASTX hits. Click here for file

Additional file 2 **Information on developed 76 locus single-run INDEL panel in Atlantic salmon**. Information on fluorescence labeling, primer concentrations, PCR pooling and links to alignments, INDEL motifs and GENESCAN (Burge and Karlin 1997) predictions of genes/exons are available in html format. Click here for file

Additional file 3 Information on GenBank accession numbers, primer sequences and literature references of the genomic and EST-derived microsatellite markers used for construction of Atlantic salmon linkage map. Click here for file

Additional file 4 Linkage information of 50 INDELs and 77 microsatellite markers that were segregating in two families used for generation of Atlantic salmon linkage map. Click here for file

Additional file 5 **Results from interval mapping using Haley-Knott regression in linkage groups larger than 5 cM**. F = QTL Express F statistic; cM = Kosambi centi-Morgan. Marker positions are indicated at the top. Chromosome-wide permutation test significance thresholds (P < 0.05; P < 0.01) are indicated by dotted and dashed lines, respectively. Click here for file

Additional file 6 PCR panels and amplification protocols for microsatellite loci. Click here for file
