# Supplementary material for: Discovery and application of insertion-deletion (INDEL) polymorphisms for QTL mapping of early life-history traits in Atlantic salmon
Source: BMC Genomics. 2010 Mar 8;11:156. doi: 10.1186/1471-2164-11-156 (PMC2838853; doi:10.1186/1471-2164-11-156)
Supplement: Additional file 1 — Information on 202 INDELs tested in Atlantic salmon containing GenBank accession numbers, primer sequences, observed sizes of fragments and BLASTX hits. [file 1471-2164-11-156-S1.DOC]

**Appendix 1.** Information on 202 tested INDELs containing marker names, GenBank accession numbers, observed sizes of fragments, primer sequences and BLASTX hits.

| No. | Marker name | Alternate | GenBank | Observed sizes of fragments | | | Polym | Indel | M13 tail & Forward primer 5'-3' | Reverse primer 5'-3' | Comment | | BlastX hit | | E-value |
| --- | --- | --- | --- | --- | --- | --- | --- | --- | --- | --- | --- | --- | --- | --- | --- |
|  |  | name | Acc. No. | Bur | Can | Narva | code | size | M13 (CACGACGTTGTAAAACGAC) |  |  | |  | |  |
| 1 | *SsaIND100TKU* | *8604L* | EG831853 | 89 | 89 | 89/97 | P | 6 | CACGACGTTGTAAAACGACGGCTGGATTCAGTGCTTTTC | GTTTCAAGGTCTGACCAGCAGATG | microsatellite | | Prefoldin subunit 6 [Salmo salar] 240 6e-62 | | 6.00E-62 |
| 2 | *SsaIND106TKU* | *9552C* | EG757129 | 216218 | 217/219 | 216218 | P | 2 | CACGACGTTGTAAAACGACGCATTTTGAGGTGGGAAAGA | GTTTGGCTGTTGTATGTGATGGTG |  | | - | |  |
| 3 | *SsaIND146TKU* | *17363D* | EG758656 | 100/102 | 100/102 | 100/102 | P | 2 | CACGACGTTGTAAAACGACCCAAAAGAGTATGCCCCTCT | GTTTAGCCTGGTTGCCGTCTAT |  | | - | |  |
| 4 | *SsaIND165TKU* | *21745D* | EG817596 | - | 263 | 260/263 | P | 3 | CACGACGTTGTAAAACGACTGAAGCAGAGCCAGACAGAA | GTTTCATGCCACTTGGACAACAT |  | | - | |  |
| 5 | *SsaIND178TKU* | *20411A* | DW580704 | 156/159 | 156/159 | 156/159 | P | 3 | CACGACGTTGTAAAACGACCAGGGGCACAAACAAAACA | GTTTAGTGGCACACAACGAACATC |  | | - | |  |
| 6 | *SsaIND192TKU* | *Ind2880* | EG786105 | - | - | 127/129 | P | 2 | CACGACGTTGTAAAACGACACTGTCTTTCCCCTCCCTGT | GTTTTTGAGAGAGAAGCTGGTACG |  | | mediator complex subunit 19b [Danio rerio] | | 3.00E-47 |
| 7 | *SsaIND193TKU* | *Ind139* | EG860477 | - | - | 137/143 | P | 6 | CACGACGTTGTAAAACGACCATCCATAAAGTCCCCAAGC | GTTTCAGAGCACTCCAACCACAGA |  | | Peroxiredoxin-5, mitochondrial precursor [Salmo salar] | | 1.00E-76 |
| 8 | *SsaIND194TKU* | *Ind457C* | EG777352 | - | - | 149/152 | P | 3 | CACGACGTTGTAAAACGACTAAAAATGCAAGGCACACCA | GTTTGGAATGCTGGTTCTTCTGTC |  | | - | |  |
| 9 | *SsaIND195TKU* | *Ind1836* | EG854090 | - | - | 168/177 | P | 9 | CACGACGTTGTAAAACGACCAGCAGCAGTGTGTCCTCTC | GTTTCTGAGGGCCGAATCCTAAAT |  | | - | |  |
| 10 | *SsaIND196TKU* | *Ind2679* | EG872866 | - | - | 204/208 | P | 2.2 | CACGACGTTGTAAAACGACGTTTACACAGGCAGCCCAAT | GTTTCCCTTTCCCCACTAAAAACA |  | | - | |  |
| 11 | *SsaIND197TKU* | *Ind2130* | EG887233 | - | - | 269/270 | P | 7 | CACGACGTTGTAAAACGACCATGCCTGAGCCAGCTAAAT | GTTTCTCCTCCTCCTCTCCAACA |  | | vasotocin receptor V1-beta [Takifugu rubripes] | | 2.00E-09 |
| 12 | *SsaIND092TKU* | *7157K* | EG836414 | 111/113 | 113 | 111/113 | P | 2 | CACGACGTTGTAAAACGACTGAGATTGGATAAACCCACAAA | GTTTGGAAAGGACAACATCACAGGA |  | | Carbonyl reductase 1 [Salmo salar] | | 2.00E-58 |
| 13 | *SsaIND096TKU* | *8229A* | CB516218 | 562 | 562 | 562/567 | P | 5 | CACGACGTTGTAAAACGACGACAGCAGCGACAAAAACAA | GTTTCACCCCATTGAAGTTGAGGT |  | | - | |  |
| 14 | *SsaIND117TKU* | *11953L* | EG897476 | 427/430 | 427/430 | 428/430 | P | 3 | CACGACGTTGTAAAACGACTTACCTCCCTCAAACGCAAC | GTTTAGGCGGTCTTTAGGGTTTTC |  | | - | |  |
| 15 | *SsaIND129TKU* | *13526G* | EG932893 | 454/456 | 454/456 | 454/456 | P | 2 | CACGACGTTGTAAAACGACCTGGCATGACACAGTCCAAA | GTTTGCATTGTGGATTACTGCTTCG |  | | - | |  |
| 16 | *SsaIND136TKU* | *15402E* | DY714614 | - | 572 | 570/572 | P | 2 | CACGACGTTGTAAAACGACTTGGATATTCTGGGCTTTGG | GTTTCATTTTGCACAGGAGAACGA |  | | - | |  |
| 17 | *SsaIND174TKU* | *20056D* | DW547818 | 308 | 308/310 | 308 | P | 3 | CACGACGTTGTAAAACGACCGCTACAACACACCCATCAA | GTTTGCTACCACTGAAGAAACTGC | 3 bp INDEL in exon | | hypothetical protein LOC406827 [Danio rerio] | | 5.00E-37 |
| 18 | *SsaIND198TKU* | *Ind1921* | CB508135 | - | - | 312/316 | P | 4 | CACGACGTTGTAAAACGACGCACGCCATAAAGAATGGAT | GTTTGCAGTAGACCCATCACGTC |  | | Calcium-binding mitochondrial carrier protein Aralar1 | | 2.00E-23 |
| 19 | *SsaIND199TKU* | *Ind2070* | CK898265 | - | - | 324/327 | P | 4 | CACGACGTTGTAAAACGACTGACTGACCAACTCCACACA | GTTTCGAAACGACCTCAAAGCACT |  | | - | |  |
| 20 | *SsaIND200TKU* | *Ind2231* | EG759430 | - | - | 329/331 | P | 2 | CACGACGTTGTAAAACGACCCAGCAAACTCGAACATGAA | GTTTAAGAAGCCCTTACCCCAAAA |  | | - | |  |
| 21 | *SsaIND201TKU* | *Ind2377* | EG792034 | - | - | 415/419 | P | 6 | CACGACGTTGTAAAACGACCATCTGCTCAACCCAAGACA | GTTTATTCGGACGTTGCAGATTTC |  | | - | |  |
| 22 | *SsaIND202TKU* | *Ind2330* | EG873536 | - | - | 530/537 | P | 7 | CACGACGTTGTAAAACGACAACTGCCTCTGGGTGAAATG | GTTTGCTCATGGGAAGGGATATAGG |  | | - | |  |
| 23 | *SsaIND007TKU* | *3960I* | EG822398 | 275/277 | - | 275/277 | P | 2 | CACGACGTTGTAAAACGACTCCATTTTGCGTTACCACAG | GTTTGCTGTCCAAGAACATTTCACA |  | | - | |  |
| 24 | *SsaIND008TKU* | *4151e* | EG869785 | 198/200 | 198/200 | 198/200 | P | 2 | CACGACGTTGTAAAACGACTGACATCCAAATTCAACCAGA | GTTTCACCTGGGAACCAGACAGTT |  | | ATP synthase subunit e, mitochondrial [Salmo salar] | | 6.00E-16 |
| 25 | *SsaIND018TKU* | *9520D* | EG791246 | 127/129 | 127 | 126/129 | P | 2 | CACGACGTTGTAAAACGACCATCTGCCCAAACGTATCAA | GTTTCAGAATCGGCTTGGAAGAAG |  | | Small nuclear ribonucleoprotein G [Salmo salar] | | 1.00E-35 |
| 26 | *SsaIND022TKU* | *32c* | DW584142 | 177 | 177 | 175/177 | P | 2 | CACGACGTTGTAAAACGACATGGTTAGCAGTGAGCAGCA | GTTTAATGCCCTGATTCTGTGGAC |  | | H-2 class II histocompatibility antigen gamma chain [Salmo salar] | | 2.00E-72 |
| 27 | *SsaIND032TKU* | *1094G2* | EG930476 | 100/106 | 100/106 | 100/106 | P | 3.6 | CACGACGTTGTAAAACGACGGAGGAGGCAACAAGGGTAT | GTTTAGAAGTGGTTGGGTTGGTGA | 3bp repeat in exon | | hypothetical protein [Danio rerio] | | 3.00E-38 |
| 28 | *SsaIND038TKU* | *1338L* | EG859393 | 218/224 | 218/224 | 218/224 | P | 6 | CACGACGTTGTAAAACGACAGGGGGTGGGAAAAACATAC | GTTTGTGGGGGAATTTAATGAGG |  | | Dolichyl-diphosphooligosaccharide-protein glycosyltransferase [Danio rerio] | | 5.00E-17 |
| 29 | *SsaIND054TKU* | *2456V* | EG792234 | 153/156 | 156 | 153/156 | P | 3 | CACGACGTTGTAAAACGACAGTGGCTAGCTGCTTTGGAA | GTTTCTGAGAGCAGATCCTTCCTT |  | | hypothetical protein LOC550376 [Danio rerio] | | 1.00E-75 |
| 30 | *SsaIND065TKU* | *2889J* | EG931347 | 232/234 | 234 | 232/234 | P | 2 | CACGACGTTGTAAAACGACCCTGTGCACCCATAAATGAA | GTTTAGAGGACGGAACAGGTTGTG |  | | - | |  |
| 31 | *SsaIND095TKU* | *7583I* | EG905690 | 254 | 250/252 | 254 | P | 2 | CACGACGTTGTAAAACGACGTCCCATTATCCAACCAACC | GTTTGCCTTCAGTCATCAAACCA |  | | - | |  |
| 32 | *SsaIND110TKU* | *10585C* | EG761490 | 139 | 141 | 139 | TA P | 2 | CACGACGTTGTAAAACGACTGCGAGGTTCAGAGTTGTCTT | GTTTAATAATGCATGGGGCTGAAG |  | | - | |  |
| 33 | *SsaIND004TKU* | *3355K* | EG903132 | 427/429 | 429 | 427/429 | P | 2 | CACGACGTTGTAAAACGACGCCAAACAGCCATACGAGAT | GTTTCTTCCTTCCCCTCACTCTCC |  | | - | |  |
| 34 | *SsaIND020TKU* | *10259H* | EG840630 | 311/316 | 311/316 | 311/316 | P | 5 | CACGACGTTGTAAAACGACGGTGCCATTCGTTCAGATTT | GTTTGAAGAGGGGTTGGGAATGTT | microsatellite | | - | |  |
| 35 | *SsaIND021TKU* | *11005M* | EG910563 | 373375 | 373 | 373375 | P | 2 | CACGACGTTGTAAAACGACTGTGCTCTCCTCCTTAACAATG | GTTTATCAAGCCACACGTCAACAA |  | | - | |  |
| 36 | *SsaIND049TKU* | *2136E* | CA046731 | 350/354 | 352 | 350/354 | P | 2.4 | CACGACGTTGTAAAACGACGATCCTACTGGCCCTGTGTG | GTTTAGTTATCCAGGCCACAATGC |  | | Annexin A1 [Salmo salar] | | 4.00E-35 |
| 37 | *SsaIND058TKU* | *2571c* | DW471900 | 331 | 333 | 331/333 | P | 2.7 | CACGACGTTGTAAAACGACTGAGCAGAGGATTGAGAAGGA | GTTTGTAGTGGGTGTGTCCAACA |  | | - | |  |
| 38 | *SsaIND076TKU* | *4246L* | EG782907 | 420/422 | 420/422 | 420/422 | P | 2.2 | CACGACGTTGTAAAACGACGCAGTGGTAAGATGGGCACT | GTTTAAATGATGACCGACGATTGG |  | | ribophorin II [Danio rerio] | | 1.00E-11 |
| 39 | *SsaIND113TKU* | *10789B* | EG766107 | 412 | 410/412 | 412 | P | 2 | CACGACGTTGTAAAACGACCATGCCTCTTCTCCCTACCA | GTTTCCCACCATAAAAACAATCCA |  | | similar to CDEP [Ornithorhynchus anatinus] | | 1.00E-21 |
| 40 | *SsaIND150TKU* | *18185F* | CA044968 | 504 | 498 | 504 | TA P | 3 | CACGACGTTGTAAAACGACTTTTGCCATGTTTGCCATTA | GTTTGCCAGTGAAAGCCTGTATGG |  | | unnamed protein product [Tetraodon nigroviridis] | | 3.00E-09 |
| 41 | *SsaIND151TKU* | *18256D* | EG822439 | 402 | 400/402 | 402 | P | 2 | CACGACGTTGTAAAACGACATAAAAGACTGGCCGAAGCA | GTTTAGCTGGACAGAGACGTGTGA |  | | - | |  |
| 42 | *SsaIND017TKU* | *8396P* | EG787097 | 116/120 | 116/120 | 116/120 | P | 4 | CACGACGTTGTAAAACGACATGCTTGCCTCCATACCAAC | GTTTACACAGAAACTGCAGCAAGG |  | | - | |  |
| 43 | *SsaIND023TKU* | *86g* | EG839692 | 203/206 | 206 | 206 | P | 3 | CACGACGTTGTAAAACGACTCCTAAAACACGGAGCCAAG | GTTTGGAATGACCCAGGTATGGT |  | | Myosin regulatory light chain 2, smooth muscle isoform [Salmo salar] | | 2.00E-91 |
| 44 | *SsaIND024TKU* | *190S* | CB500290 | 100/104 | 100/104 | 104105 | P | 3 | CACGACGTTGTAAAACGACTGCACAACAGAGACGAAACC | GTTTGCCATTCAACTCACCCCTAA |  | | - | |  |
| 45 | *SsaIND035TKU* | *1271X* | EG872016 | 166 | 169 | 166/169 | P | 3 | CACGACGTTGTAAAACGACTATTGCCCGGAGTCAAGTTC | GTTTCAAGCAACATGCAGAAAGC | 3bp repeat in exon | | - | |  |
| 46 | *SsaIND037TKU* | *1329g* | EG867098 | 226/228 | 226/228 | 228 | P | 2 | CACGACGTTGTAAAACGACTGGAGAGGTCACAAGCACTG | GTTTACACGGTTTCCTTCTTCCTG |  | | COP9 signalosome complex subunit 6 [Salmo salar] | | 1.00E-66 |
| 47 | *SsaIND051TKU* | *2273K* | DW540946 | 194/197 | 194/197 | 194/197 | P | 3 | CACGACGTTGTAAAACGACCCTGCCTCTCAGGACTGTTC | GTTTAGCGACAAATTACCCATCCA |  | | Chromobox protein homolog 3 [Salmo salar] | | 6.00E-41 |
| 48 | *SsaIND079TKU* | *4868M* | EG770025 | 176 | 176 | 172/176 | P | 4.4 | CACGACGTTGTAAAACGACCAGGGGTCTGTTCAGGATGT | GTTTACTCCTCAAACTGGGGTGTG |  | | - | |  |
| 49 | *SsaIND179TKU* | *20475C* | EG765334 | 186/189 | 186/189 | 186 | P | 3 | CACGACGTTGTAAAACGACACAGCATGCCACCAACACTA | GTTTCTTTTCAGCACAAGGCATGTA |  | | - | |  |
| 50 | *SsaIND185TKU* | *21188C* | EG647956 | 149 | 149 | 147/149 | P | 2 | CACGACGTTGTAAAACGACCCCGATGTGGTAAACAAAGC | GTTTGACCGAACTGGTCCTACCTG |  | | ankyrin repeat, family A (RFXANK-like), 2 [Danio rerio] | | 9e-48 |
| 51 | *SsaIND067TKU* | *3270v* | CB500451 | 372 | - | 381 | P | 3 | CACGACGTTGTAAAACGACGCATGGTTCCAAAGCAAAAA | GTTTCCCGTTCAGAACAAAGCAAA | 3 bp INDEL in exon, obs. INDEL 9bp | | gene trap locus 3 [Mus musculus] | | 2.00E-74 |
| 52 | *SsaIND098TKU* | *8417F* | EG782320 | 468 | 466/468 | 468 | P | 2 | CACGACGTTGTAAAACGACTTTAAAGGCCCAGTGCAATC | GTTTGGGCAGAATGAGCCATCTAA |  | | - | |  |
| 53 | *SsaIND125TKU* | *13049E* | DY692052 | 561/563 | 561/563 | 561/563 | P | 4 | CACGACGTTGTAAAACGACTGACTAAGGTGGTGGGGAAG | GTTTCACTTCAATAATGCAAAACCACA | microsatellite | | - | |  |
| 54 | *SsaIND130TKU* | *13587E* | DY692726 | 412/414 | 414 | 412/414 | P | 2 | CACGACGTTGTAAAACGACGGAGGAATAAGGGAGGAGCA | GTTTCGGGAAATAGTGCTTTTG |  | | caspase-1 [Dicentrarchus labrax] | | 7.00E-25 |
| 55 | *SsaIND142TKU* | *16424E* | CA063272 | 430/432 | 430/432 | 430/432 | P | 2 | CACGACGTTGTAAAACGACAAGGCAGCAAAACTGGTAGC | GTTTGGGGACACTGGAGTGAAAAT |  | | - | |  |
| 56 | *SsaIND147TKU* | *17571D* | DY708327 | 256 | 256 | 248/256 | TA P | 8 | CACGACGTTGTAAAACGACACCGAATAAAGCCGCACTTA | GTTTCCTAATCGCACACCCAAT |  | | - | |  |
| 57 | *SsaIND160TKU* | *20196E* | EG942430 | 294/296 | 294/296 | 296 | P | 2 | CACGACGTTGTAAAACGACAACTCCCCAATCCTTTGACA | GTTTGGCATCAACATTTGAAAACCA |  | | - | |  |
| 58 | *SsaIND183TKU* | *20742A* | CA064223 | 497/508 | 504/508 | 508 | P | 4 | CACGACGTTGTAAAACGACGCCCAAAATGTACAGGCAAT | GTTTGATTCTCATGTTAGCCGTCCA |  | | - | |  |
| 59 | *SsaIND009TKU* | *4237O* | DW473270 | 132/138 | 136/141 | 138/141 | P | 4 | CACGACGTTGTAAAACGACCCTGGGGACAGTACAACCAG | GTTTAATTGGGCTGGGTAGAAAGG |  | | pre-mRNA processing factor 39 homolog [Danio rerio] | | 7.00E-30 |
| 60 | *SsaIND036TKU* | *1309C* | EG808658 | 200/210 | 200/210 | 200/210 | P | 10 | CACGACGTTGTAAAACGACCAAAGGGCTGTTAAAGGAATG | GTTTCTGTATGGTGCATTTAAGATCATTG | 10bp INDEL immediately after exon | | - | |  |
| 61 | *SsaIND080TKU* | *4955H* | EG778546 | 231 | - | 231/233 | P | 2 | CACGACGTTGTAAAACGACAACCTTCCCCTGATCTTGCT | GTTTCACTACAACCGCTCCATCCT |  | | Tumor suppressor candidate 2 [Salmo salar] | | 7.00E-43 |
| 62 | *SsaIND099TKU* | *8570Q* | EG889615 | 100/104 | 100/104 | 100/104 | P | 3 | CACGACGTTGTAAAACGACAGCAGGTCGTGTTTTGAAGC | GTTTCTGATCGTCCTCGCCTTTAC | 3 bp INDEL in exon | | selenoprotein H [Danio rerio] | | 3e-25 |
| 63 | *SsaIND118TKU* | *11971N* | EG796527 | 107/109 | 109 | 109 | P | 2 | CACGACGTTGTAAAACGACTATGCGCTACCCCACTAACG | GTTTAGATACGAGGCAGGGGTGT |  | | - | |  |
| 64 | *SsaIND122TKU* | *12783G* | EG830992 | 244 | 244/246 | 244 | P | 2 | CACGACGTTGTAAAACGACTGTGGGATTCATTTCCTGTG | GTTTGGACTGAGGGCATGCTTTTT |  | | M-phase phosphoprotein 6 [Salmo salar] | | 6.00E-71 |
| 65 | *SsaIND123TKU* | *13028H* | EG823293 | 160/167 | 167 | 160/164/167 | P | 2 | CACGACGTTGTAAAACGACGGGACAACAAGCTCTGCTAAA | GTTTCGTGGACCAGGCTAGGATTA |  | | protein tyrosine kinase 2 beta, b [Danio rerio] | | 2.00E-52 |
| 66 | *SsaIND140TKU* | *15900H* | EG841183 | 221 | 221 | 217/221 | P | 5 | CACGACGTTGTAAAACGACGCTTTCGTTTATTTTGAACCA | GTTTACACACTGGGCTTCGTTTTC |  | | - | |  |
| 67 | *SsaIND145TKU* | *17300F* | EG911321 | 149/153 | 149 | 149/153 | P | 4 | CACGACGTTGTAAAACGACTCCAATTTCATGTTGCTAATGTG | GTTTAACCTTCCCCTAACCCTGTAA |  | | - | |  |
| 68 | *SsaIND171TKU* | *22471D* | EG924462 | 85/90 | 85/90 | 85/90 | P | 5 | CACGACGTTGTAAAACGACAAGAGCCGTTCGTTCTGGT | GTTTATAGTGCTCGCTGCTTGC |  | | - | |  |
| 69 | *SsaIND016TKU* | *7655N* | EG926217 | 413/415 | 415 | 413/415 | P | 2 | CACGACGTTGTAAAACGACGCTGTTGCCCTGTTTTTGTT | GTTTATCGGGGAGCAGTTTCTTTT |  | | - | |  |
| 70 | *SsaIND042TKU* | *1729I* | EG817916 | 357/359 | 359 | 357/359 | P | 2.2 | CACGACGTTGTAAAACGACGGTACTCCGAGCAGAGCAAA | GTTTAGACCTGCACCCAAAAGAAA |  | | - | |  |
| 71 | *SsaIND078TKU* | *4493F* | CA060073 | 474 | 474 | 472/474 | P | 2 | CACGACGTTGTAAAACGACTCTTCCTGTGAAGCACGTTG | GTTTGTTTCGGGAATGTGGGTAT |  | | - | |  |
| 72 | *SsaIND081TKU* | *5134V* | EG844218 | 558 | 558/564 | 558 | P | 6 | CACGACGTTGTAAAACGACAAGTCATGTCCCCGACTCAC | GTTTCTTGGCGCTTTTGGTACAAT |  | | Immediate early response gene 5 protein [Salmo salar] | | 3.00E-35 |
| 73 | *SsaIND102TKU* | *8921B* | CA054270 | 370/376 | 368 | 370/376 | P | 2 | CACGACGTTGTAAAACGACAACCCGTATTGGGGAAAGAC | GTTTCAGCACTGACCATCCACTGT |  | | - | |  |
| 74 | *SsaIND126TKU* | *13066I* | EG807593 | 541/543 | 541 | 541/543 | P | 2 | CACGACGTTGTAAAACGACCCGAGGCTTTTTACAGTTGG | GTTTCTCTGTTGGGTGGAAAAGG |  | | - | |  |
| 75 | *SsaIND143TKU* | *16730E* | EG815522 | 487 | 487/493 | 487/493 | P | 6 | CACGACGTTGTAAAACGACTTTCCAGACAATCCCAGACA | GTTTGGGGTTTGTTTTTGTGGAGA | microsatellite | | - | |  |
| 76 | *SsaIND191TKU* | *21965C* | EG902270 | 392 | 392/398 | 392/398 | P | 6 | CACGACGTTGTAAAACGACGGCCTGGATAATGGGAGAAT | GTTTGTCCTTGATGCCTCTGGAAA |  | | Sperm acrosome membrane-associated protein 4 [Salmo salar] | | 5.00E-27 |
| 77 | *SsaIND001TKU* | *3019T* | CB515254 | 490 | 481/490 | 490 | P | 9 | CACGACGTTGTAAAACGACGCCATGTGATGGTGTGGATA | GTTTGCGTCTGAGTGACCTTTTG |  | | - | |  |
| 78 | *SsaIND097TKU* | *8272L* | EG909736 | 389 | 389/401 | 389 | P | 2 | CACGACGTTGTAAAACGACGCCACCCATTCATTTAGCAT | GTTTGGGGCTATGTTTCAATCTGG |  | | - | |  |
| 79 | *SsaIND002TKU* | *3060S* | DY693476 | 245 | 245/247 | 245/247 | P | 2 | CACGACGTTGTAAAACGACTTGAGCCACACGTACTGGAG | GTTTGAAAGCAGAGCAAGATGGA |  | | Ancient ubiquitous protein 1 [Salmo salar] | | 1.00E-37 |
| 80 | *SsaIND003TKU* | *3335U* | DW536008 | 590 | 590/592 | 590 | P | 2 | CACGACGTTGTAAAACGACTATTTTCCCCTGCTTTCAGC | GTTTGGTCGAGTGAGGCGTATAA |  | | - | |  |
| 81 | *SsaIND005TKU* | *3772K* | DW570989 | 381/383 | 381/383 | 381 | P | 2 | CACGACGTTGTAAAACGACTGCACTATTGGGGTCTTTTTG | GTTTGTGCTCAAAAGGGAAAAGCA |  | | - | |  |
| 82 | *SsaIND101TKU* | *8917C* | EG806338 | 503 | 503 | 503 | M | 2 | CACGACGTTGTAAAACGACATGGTGGCGTTAGGTTCATC | GTTTGGGAACTAGGTGGGCATACA |  | | - | |  |
| 83 | *SsaIND006TKU* | *3838M* | EG852983 | 378 | 378/380/382 | 378 | P | 2 | CACGACGTTGTAAAACGACTGGCTTGACATTTGCACTTC | GTTTAGATTGGTGGTGAGGGACTG |  | | - | |  |
| 84 | *SsaIND103TKU* | *9261A* | EG758703 | 184 | 172/184 | 172/184 | P | 11 | CACGACGTTGTAAAACGACTGCGTTCATTCTGTTCCAAA | GTTTCACAGTGATTCAGACTGTGCATT |  | | - | |  |
| 85 | *SsaIND104TKU* | *9329H* | EG807579 | 450/452 | 450/452 | 450/452 | MB | 2 | CACGACGTTGTAAAACGACGGCAAGTGAGGTGTCCATTT | GTTTCCATCCAATAACATTCCATGTC |  | | - | |  |
| 86 | *SsaIND105TKU* | *9348G* | DY724240 | - | - | - | NA | 4 | CACGACGTTGTAAAACGACGTGCGTCCACTTCCAATTTT | GTTTCAGCATCGTCATCAGGTCAT |  | | S100-A4 [Salmo salar] | | 1.00E-29 |
| 87 | *SsaIND010TKU* | *4319d* | DY735378 | - | - | - | NA | 3 | CACGACGTTGTAAAACGACCAAACGCTGTGAGGAGATGA | GTTTGGTGACGATGACCTGAGAC |  | | unnamed protein product [Tetraodon nigroviridis] | | 5.00E-95 |
| 88 | *SsaIND107TKU* | *10046I* | EG855570 | 372/374 | 361/372 | 372 | P | 2 | CACGACGTTGTAAAACGACGATGAAGCGACTGCTTGTGA | GTTTGGTTAAGGCTGGTCTGTGTC | microsatellite | | - | |  |
| 89 | *SsaIND011TKU* | *4707f* | EG822391 | 564 | 564 | 564/566 | P | 2 | CACGACGTTGTAAAACGACGGTTCTGAAGCCATTTCAGG | GTTTATTTGGGGTATGCCAACAAG |  | | - | |  |
| 90 | *SsaIND108TKU* | *10252C* | EG759880 | 396/398 | 391/400 | 396/398 | MB | 2 | CACGACGTTGTAAAACGACTGTTTTGGGCTGTGTATTTGA | GTTTGTGCGAGGTAGAAGGCAGTC |  | | - | |  |
| 91 | *SsaIND012TKU* | *4883Z* | CA042409 | - | - | - | NA | 4 | CACGACGTTGTAAAACGACTGCCATTTTAATTCAGGCAAA | GTTTGCGGAAAGTTAGTGATTGCAG |  | | - | |  |
| 92 | *SsaIND109TKU* | *10431E* | CA053993 | - | - | 373/375 | MB | 7 | CACGACGTTGTAAAACGACCAACAGGGGGAAATAAACACA | GTTTGTTGGGGGATTATGGTTCCT |  | | - | |  |
| 93 | *SsaIND013TKU* | *5397X* | DW567958 | 100/233 | 100/233 | 100/233 | Dupl | 2 | CACGACGTTGTAAAACGACGGTCCATCGGCTTAAAAATG | GTTTGCGCCTGCATTTGAAGTAAC |  | | - | |  |
| 94 | *SsaIND014TKU* | *7090M* | CB511444 | 133/135 | 133/135 | 133/135 | Dupl | 2 | CACGACGTTGTAAAACGACGGGGAGGGGTTTCAGTTCT | GTTTCAGTGGACCCCCTGTG |  | | similar to Protein kinase C-binding protein NELL2  precursor (NEL-like protein 2) (Nel-related protein 2) [Danio rerio] | | 2.00E-43 |
| 95 | *SsaIND111TKU* | *10699B* | EG808811 | 301/303 | 303 | 301/303 | P | 2 | CACGACGTTGTAAAACGACCTGTGCATTGAGTTCCCTGT | GTTTGCATTTTGGACGGATGTAA |  | | - | |  |
| 96 | *SsaIND015TKU* | *7444J* | EG912942 | - | - | - | NA | 3 | CACGACGTTGTAAAACGACCACATGTTTAGGAGGCACCTG | GTTTACAAGGAGCTGGCTGAAGAA |  | | similar to BTB/POZ domain-containing protein KCTD17  [Danio rerio] | | 2.00E-79 |
| 97 | *SsaIND112TKU* | *10766D* | EG934479 | 496 | 500 | 496 | TA P | 5 | CACGACGTTGTAAAACGACACAGTGTCAACCATGCCTCA | GTTTGAACCAAGGGAGCTTCGTAA |  | | - | |  |
| 98 | *SsaIND114TKU* | *10950K* | DW472505 | - | - | - | NA | 2 | CACGACGTTGTAAAACGACTGTATCCAAACCTCCCCAGA | GTTTCCATATTGCTTCCTTTTGCAC |  | | - | |  |
| 99 | *SsaIND019TKU* | *10023K* | EG834896 | - | - | - | NA | 3 | CACGACGTTGTAAAACGACAGTCTAGGCGTGCTGTCGTT | GTTTGATCCCGATGACAAATG |  | | - | |  |
| 100 | *SsaIND115TKU* | *11149O* | EG918960 | 513 | 513 | 513 | M | 3 | CACGACGTTGTAAAACGACAGCTTTCCATGTCCTTGCAG | GTTTGGACCGAAGAAGAATCCACA |  | | - | |  |
| 101 | *SsaIND116TKU* | *11797E* | DW568160 | 521 | 521 | 521 | M | 2 | CACGACGTTGTAAAACGACTTGCTGCGTGAAGTTGTCTC | GTTTCCAGGACATCCACATGACTG |  | | - | |  |
| 102 | *SsaIND119TKU* | *12042D* | CB502715 | 141/150/253 | 150/253/256 | 141/150/253/256 | Dupl | 6 | CACGACGTTGTAAAACGACCAATGAATTTGTGTTGGTTCAAA | GTTTGCTTCCTGTATCCTAAATTAACA |  | | rCG39392, isoform CRA_a [Rattus norvegicus] | | 2.00E-16 |
| 103 | *SsaIND120TKU* | *12140G* | EG777191 | 454 | 454 | 454 | M | 2 | CACGACGTTGTAAAACGACCATCTACGCGCATCCATCTA | GTTTATTGAAGCGAATGAGGATGG | microsatellite | | - | |  |
| 104 | *SsaIND121TKU* | *12735D* | EG765476 | 128 | 128 | 128 | M | 3 | CACGACGTTGTAAAACGACTTGTCATAGCGGATGAAGGA | GTTTGGTGAACAACAGCCGAGAG |  | | unnamed protein product [Tetraodon nigroviridis] | | 4.00E-13 |
| 105 | *SsaIND025TKU* | *228t* | EG773471 | - | - | - | NA | 5 | CACGACGTTGTAAAACGACTTAGCCTTGGCAGGACTCAT | GTTTCCAGAGGTGATGGCAGTACA |  | | H-2 class II histocompatibility antigen, A-K beta chain precursor  [Salmo salar] | | 1.00E-30 |
| 106 | *SsaIND026TKU* | *467C* | EG765224 | 399 | 387 | 399 | TA P | 11 | CACGACGTTGTAAAACGACACTCATCTGGCACTCCAAGC | GTTTGACTTTGCCCTTCATTCCAA |  | | Cytochrome b-c1 complex subunit 7 [Salmo salar] | | 3.00E-41 |
| 107 | *SsaIND027TKU* | *548d* | EG831071 | 357/359 | 359 | 359 | P | 2 | CACGACGTTGTAAAACGACTGGGAAAGATGAAAGCCTTG | GTTTAATGGGACAGCCTTGTGATG |  | | - | |  |
| 108 | *SsaIND124TKU* | *13034D* | DY705294 | - | - | - | NA | 3 | CACGACGTTGTAAAACGACCAAACCTGTCTCCTGCTTCA | GTTTGTCTGGTCCTGCTGGTCAAT |  | | Type-1 angiotensin II receptor-associated protein-like [Salmo  salar] | | 2.00E-94 |
| 109 | *SsaIND028TKU* | *775D* | EG779661 | 324/328 | 324/328 | 324/328 | Dupl | 5 | CACGACGTTGTAAAACGACGGAACAACTTGACCCAAGACA | GTTTCCATTTCCCAAAAAGTGCAT |  | | parvalbumin beta [Salmo salar] | | 3.00E-43 |
| 110 | *SsaIND029TKU* | *905K* | EG762941 | 422/423 | 424 | 422/423 | P | 3 | CACGACGTTGTAAAACGACCAACCTCCGTCACATAGCAA | GTTTGAACAGGGCTTTCCAGAAGA |  | | - | |  |
| 111 | *SsaIND030TKU* | *974c* | CA049882 | 148/152 | 152/153 | 152 | P | 3 | CACGACGTTGTAAAACGACTTTTGTTTGCCCCCAAGATA | GTTTACAGCATGGACCCTATTTGC |  | | - | |  |
| 112 | *SsaIND031TKU* | *1021r* | EG921046 | - | - | - | NA | 2 | CACGACGTTGTAAAACGACACATGGGTCAGAGGAGCAGT | GTTTATGAGGTGGGAGACCAAGG |  | | - | |  |
| 113 | *SsaIND127TKU* | *13319F* | DW568138 | 310/326/328 | 310 | 310/326/328 | P | 2 | CACGACGTTGTAAAACGACCGTGTTTGAGAGCATCGAAA | GTTTGTTTAAGGTTGGGGTGAGG |  | | - | |  |
| 114 | *SsaIND128TKU* | *13349E* | EG897081 | 449/454 | 454/458 | 449/454/458 | Dupl | 2 | CACGACGTTGTAAAACGACGTTCCGATAGAGGGGGAGAG | GTTTGGGTGTCCTGCTGAAGAGAG |  | | - | |  |
| 115 | *SsaIND033TKU* | *1126u* | EG867411 | 124/126 | 124/126 | 124/126 | Dupl | 2 | CACGACGTTGTAAAACGACGCCTCGCACAAACACTCTGT | GTTTAAAACCTGGGGGAGATGAAG |  | | Transcription initiation factor TFIID subunit 12 [Salmo salar] | | 2.00E-87 |
| 116 | *SsaIND034TKU* | *1160C* | EG769785 | 115 | 113/115 | 115 | P | 2 | CACGACGTTGTAAAACGACTCTTGGGTCCAAGTACCACAC | GTTTCTCCTTTTGCCCCTGAACAT |  | | - | |  |
| 117 | *SsaIND131TKU* | *13714E* | DW569883 | 119/127 | 119/127 | 119/127 | P | 2 | CACGACGTTGTAAAACGACGGACAGAGTAGGGGAGGACA | GTTTACAGGATGCACCCACTCC |  | | CL011 protein [Salmo salar] | | 3.00E-57 |
| 118 | *SsaIND132TKU* | *13831D* | CA055778 | 203 | 203 | 203 | M | 2 | CACGACGTTGTAAAACGACTGCATGGACAGACACAAGAAG | GTTTGGAGCTGGTGGTTTTTCAGA |  | | - | |  |
| 119 | *SsaIND133TKU* | *14487D* | EG907723 | 292/294 | 292/294 | - | Dupl | 3 | CACGACGTTGTAAAACGACCAAAAGAGGACCTGTAAAACCA | GTTTCACACTGGGGAGAAACCTC |  | | hypothetical protein [Danio rerio] | | 7.00E-53 |
| 120 | *SsaIND134TKU* | *15107E* | EG831338 | 509 | 509/511 | 511/514 | P | 2 | CACGACGTTGTAAAACGACATGCAGGTGGACTGTTTTCA | GTTTGCGGGACAGAAATTCCATAA |  | | - | |  |
| 121 | *SsaIND039TKU* | *1445a* | EG861258 | 100/103 | 100 | 100/103 | P | 3 | CACGACGTTGTAAAACGACCCCAAGGTCTGAGAAGTCCA | GTTTGCCATGCAGGATAATGACAA | 3 bp indel in exon | | mRNA turnover protein 4 homolog [Salmo salar] | | 5.00E-84 |
| 122 | *SsaIND135TKU* | *15353C* | CB510341 | 316 | 322 | 314/316 | P | 6 | CACGACGTTGTAAAACGACCAGCACTATCCAAAGGCGTA | GTTTGGTGACAGGACAGGTGTGT |  | | - | |  |
| 123 | *SsaIND040TKU* | *1536l* | DY734180 | 707 | 707 | 707 | M | 2 | CACGACGTTGTAAAACGACCTGCCGTTTTAGCATGGAAT | GTTTATACTGGAAGGCCGGTTGT |  | | Leukocyte surface antigen CD53 [Salmo salar] | | 1.00E-38 |
| 124 | *SsaIND137TKU* | *15429F* | EG760556 | - | 215 | 215 | M | 2 | CACGACGTTGTAAAACGACTGTAGCTTTCAGGGGCAAAA | GTTTATGCGAGCGATTCAAGAAAT |  | | - | |  |
| 125 | *SsaIND041TKU* | *1553K* | EG916238 | - | - | - | NA | 3 | CACGACGTTGTAAAACGACATGACACAGCCACATCAACG | GTTTGCTTGCAGTTGCTCCTCTGT |  | | Ubiquinone biosynthesis protein COQ9, mitochondrial precursor  [Salmo salar] | | 1.00E-131 |
| 126 | *SsaIND138TKU* | *15689A* | EG844287 | - | - | - | NA | 4 | CACGACGTTGTAAAACGACTCAACCCTCACCCTCTGTGT | GTTTCTGTCAAGCCCAAGTCATCA |  | | - | |  |
| 127 | *SsaIND139TKU* | *15792G* | CB499269 | 353 | 353 | 353 | M | 2 | CACGACGTTGTAAAACGACTGTGCAAAGCCAAAATCAAA | GTTTCTGCCCTTAACTCACCCTTG |  | | - | |  |
| 128 | *SsaIND043TKU* | *1792S* | DW553979 | - | - | - | NA | 11 | CACGACGTTGTAAAACGACAGAGCATCGACCGCAACTAC | GTTTGGCCGAGGTGATAAAACAGA |  | | sorting nexin 12 [Danio rerio] | | 2.00E-84 |
| 129 | *SsaIND044TKU* | *1808e* | EG802295 | 126/109/102 | 126/109/102 | 126/109/102 | Dupl | 8 | CACGACGTTGTAAAACGACTGTGGGGCTGTAAACATGAA | GTTTGACAAGGAGGGAGGATGTGA |  | | U6 snRNA-associated Sm-like protein LSm7 [Salmo salar] | | 2.00E-39 |
| 130 | *SsaIND141TKU* | *15938E* | DY720719 | 142/154 | 152 | 142/154 | P | 2 | CACGACGTTGTAAAACGACCAAGCAAATAATGGGTCTCCA | GTTTGGGCAGAGGGTTACTTCACA |  | | - | |  |
| 131 | *SsaIND045TKU* | *1904X* | EG802622 | 103/109 | 103/109 | 103/109 | P | 8 | CACGACGTTGTAAAACGACGCCTACCAAGCTCCGTGTAA | GTTTCTAGATGAGGCAGGGCAGTC |  | | peripheral-type benzodiazepine receptor [Oncorhynchus mykiss] | | 4.00E-80 |
| 132 | *SsaIND046TKU* | *2044M* | EG767150 | 385/387 | 387 | 385/387 | P | 2 | CACGACGTTGTAAAACGACTCAAACTTATTCAACTAAAAACCATCA | GTTTTGTGGGCCTATTATCCATCC |  | | - | |  |
| 133 | *SsaIND047TKU* | *2056U* | EG869243 | 224 | 224 | 224 | M | 3 | CACGACGTTGTAAAACGACTTCGAGCCAGCTGAAGTTTT | GTTTCATCCATGTCATTGGCTGAC |  | | Anamorsin [Salmo salar] | | 6.00E-125 |
| 134 | *SsaIND144TKU* | *17105A* | CA061972 | 249/252 | 249/252 | 249/252 | Dupl | 7 | CACGACGTTGTAAAACGACGGGCGGTAGACTCACACAGT | GTTTGTCGCAGTCAGTTTCAGGTG |  | | - | |  |
| 135 | *SsaIND048TKU* | *2100N* | EG828635 | - | - | - | NA | 5 | CACGACGTTGTAAAACGACTCGCCATCTTGATGACAAAG | GTTTAGGACAGACCGAAGAGACGA |  | | - | |  |
| 136 | *SsaIND050TKU* | *2147a* | EG832340 | - | - | - | NA | 3 | CACGACGTTGTAAAACGACGGGTCTGGAACACCTGATTT | GTTTAAGCAACCGCTCCCATTAC |  | | - | |  |
| 137 | *SsaIND148TKU* | *18127E* | DW554328 | 78/82/496/499 | 78/82/496/499 | - | Dupl | 3 | CACGACGTTGTAAAACGACGATGACGACGAAGAGGAAGG | GTTTGGCATCCAGATCGTACTTTGA | microsatellite | | Zinc finger Ran-binding domain-containing protein 2 [Salmo salar] | | 2.00E-75 |
| 138 | *SsaIND052TKU* | *2317C* | EG769537 | 192/194 | 192 | 192 | P | 3 | CACGACGTTGTAAAACGACCCAGGCAGAACAGAAAAAGC | GTTTCCGTTGGGTGTTTACAGGAG |  | | CTLA-2-beta [Salmo salar] | | 6.00E-47 |
| 139 | *SsaIND149TKU* | *18130A* | EG904506 | 322/376 | 322/376 | 322/376 | Dupl | 2 | CACGACGTTGTAAAACGACCAAGCAGTGGCAAGACATTT | GTTTGGTCCTGCGTTTTCAATGAG |  | | Regulator of G-protein signaling 18 [Salmo salar] | | 2.00E-08 |
| 140 | *SsaIND053TKU* | *2373C* | CB512348 | 170 | 168 | 170 | TA P | 3 | CACGACGTTGTAAAACGACATAATCTGTGCAGGGCCAAG | GTTTGTATTTATGGGTGGTCTGG |  | | - | |  |
| 141 | *SsaIND055TKU* | *2479B* | DW535520 | 404/206/409 | 406/409 | 404/409 | Dupl | 2 | CACGACGTTGTAAAACGACACCGCTAATGTATCCCACCA | GTTTAACACCAAGCCTCTTCCAGA |  | | - | |  |
| 142 | *SsaIND152TKU* | *18362E* | EG768029 | - | - | - | NA | 2 | CACGACGTTGTAAAACGACCGAACATCGACTCCAAGCA | GTTTGTTTAAGCGCAGTAGCTTGTC |  | | - | |  |
| 143 | *SsaIND056TKU* | *2504L* | EG910115 | 221 | 221/223 | 221 | P | 2 | CACGACGTTGTAAAACGACACGGAAGGCGTGAAAATACA | GTTTCGCAGTTCGCTTGGTAAAA |  | | Transcription initiation factor TFIID subunit 10 [Salmo salar] | | 9.00E-87 |
| 144 | *SsaIND153TKU* | *18365B* | EG855392 | - | - | 445 | M | 2 | CACGACGTTGTAAAACGACGGTGCTCTTTGGGACTCTCTC | GTTTGGACTTTCCTGTGTGGTGA |  | | - | |  |
| 145 | *SsaIND057TKU* | *2520f* | EG831898 | 281/285 | 281/285 | 281/285 | Dupl | 3 | CACGACGTTGTAAAACGACGCACGTCATCGAAGTTCTCA | GTTTCTGGCCATAGTGGTCTGGAT |  | | 39S ribosomal protein L21, mitochondrial precursor [Salmo salar] | | 6.00E-116 |
| 146 | *SsaIND154TKU* | *18384F* | EG792153 | - | 548 | 504 | TA P | 2 | CACGACGTTGTAAAACGACGCGGGCTTTTCCTTTGACTA | GTTTAGAGTTGGTCGTTTGGTTGC |  | | ribosomal protein L7a [Danio rerio] | | 5.00E-67 |
| 147 | *SsaIND155TKU* | *18936C* | EG857747 | - | - | - | NA | 2 | CACGACGTTGTAAAACGACACGCACTCACACCCAACATA | GTTTCCATCCTGACCCATACAAGG |  | | - | |  |
| 148 | *SsaIND059TKU* | *2635O* | EG879232 | 104 | 102 | 104 | TA P | 2 | CACGACGTTGTAAAACGACTGGCAGTTTCTGGATGAGTC | GTTTATGCAATCTGATGCCTTCCT |  | | - | |  |
| 149 | *SsaIND156TKU* | *19146D* | DW536282 | - | - | - | MB | 3 | CACGACGTTGTAAAACGACGCGTCAAGCAGCCTAAGGTA | GTTTCAAAACGGAAGGGATGAAAC |  | | - | |  |
| 150 | *SsaIND060TKU* | *2665F* | EG930276 | 281/417 | 281/417 | 281/417 | Dupl | 6 | CACGACGTTGTAAAACGACACATGCCCTCCAGTCTCTGT | GTTTCGGAGATGAGTACGATGTGG |  | | Methylosome subunit pICln [Salmo salar] | | 9.00E-70 |
| 151 | *SsaIND157TKU* | *19822E* | EG785587 | - | 432/434 | 432 | P | 2 | CACGACGTTGTAAAACGACACAGACGAGGCGTTTTACCA | GTTTAACATGCTCTCCCAACATGA |  | | - | |  |
| 152 | *SsaIND061TKU* | *2679J* | EG872866 | 202/204 | 200/201/204 | 202/204 | Dupl | 2 | CACGACGTTGTAAAACGACCCCTTTCCCCACTAAAAACA | GTTTACACAGGCAGCCCAAT |  | | - | |  |
| 153 | *SsaIND158TKU* | *19987E* | EG804059 | - | 651 | 651 | M | 5 | CACGACGTTGTAAAACGACGCAGTCCCCAAGAAAATCAA | GTTTAGCTTTTTGGAAGCAGACGA |  | | - | |  |
| 154 | *SsaIND062TKU* | *2740B* | EG940631 | 311 | 311/313 | 311 | P | 2 | CACGACGTTGTAAAACGACTTCACGCTATTTTGACATTGG | GTTTACCGTGGTCTGAATGCTTGT |  | | - | |  |
| 155 | *SsaIND159TKU* | *20193B* | DW562072 | - | - | - | NA | 3 | CACGACGTTGTAAAACGACGGCCAAGAGGAAAAGAGAGG | GTTTAGGCACACCTTTACAAACTTTC |  | | unnamed protein product [Tetraodon nigroviridis] | | 2.00E-53 |
| 156 | *SsaIND063TKU* | *2791Y* | CA050502 | 98 | 92/98 | 98 | P | 6 | CACGACGTTGTAAAACGACACAAATAGCGGCAAGGCTAA | GTTTCAGGCGAAGATTGATTTGAAG |  | | Splicing factor, arginine/serine-rich 3 [Salmo salar] | | 4.00E-38 |
| 157 | *SsaIND064TKU* | *2836R* | EG876660 | 194/197/251 | 197/241/251 | 194/197 | Dupl | 5 | CACGACGTTGTAAAACGACGGACTGATCACACACCGAGA | GTTTCATTTCTGACTGAGCCGACA |  | | similar to vertebrate ubiquitin-fold modifier conjugating  enzyme 1 (UFC1) (zgc:100800) [Danio rerio] | | 2.00E-42 |
| 158 | *SsaIND161TKU* | *20657D* | EG942655 | 278 | 278 | 278 | M | 2 | CACGACGTTGTAAAACGACTCAAAACGAAAAGGGCAGAT | GTTTAATCGGACCCCAGAAACAG |  | | 39S ribosomal protein L43, mitochondrial precursor [Salmo salar] | | 3.00E-79 |
| 159 | *SsaIND162TKU* | *20746D* | EG834006 | 482 | 482 | 482 | M | 10 | CACGACGTTGTAAAACGACGTCTCTACCCCTCCCCTCTG | GTTTCCACAGTTCACACGTCACA |  | | - | |  |
| 160 | *SsaIND066TKU* | *3153M* | DN047699 | - | - | - | NA | 2 | CACGACGTTGTAAAACGACAAATCAGAATGGGCGTCAAG | GTTTCGGGCAAGAAGAGTTCAAAG |  | | COP9 signalosome complex subunit 7a [Salmo salar] | | 5.00E-19 |
| 161 | *SsaIND163TKU* | *21305A* | EG887233 | - | - | - | NA | 7 | CACGACGTTGTAAAACGACCTGCTGTAACCCGTGGATCT | GTTTACCGCCTGGGTTTTAGATG |  | | vasotocin receptor V1-beta [Takifugu rubripes] | | 2.00E-09 |
| 162 | *SsaIND164TKU* | *21350D* | CA054553 | - | - | - | NA | 2 | CACGACGTTGTAAAACGACATGAAGCACCCCACAGAAAC | GTTTTCCCCATGCTGCTATCT |  | | - | |  |
| 163 | *SsaIND068TKU* | *3271R* | EG932186 | 204/206 | 204/206 | 204/206 | Dupl | 2 | CACGACGTTGTAAAACGACTGCAACACAAAAGCAACACA | GTTTCATTTTGCTGCCAATAAGC |  | | 2-amino-3-ketobutyrate coenzyme A ligase, mitochondrial precursor [Salmo salar] | | 5.00E-90 |
| 164 | *SsaIND069TKU* | *3332W* | DY713366 | 410 | 410 | 410 | M | 5 | CACGACGTTGTAAAACGACTTGAAGGCAGTGAAGTGTGG | GTTTGGAATGAGCATTCAAGAGG |  | | eukaryotic translation initiation factor 4E binding protein 1  [Salmo salar] | | 3.00E-33 |
| 165 | *SsaIND166TKU* | *21966C* | DY703352 | - | - | - | NA | 2 | CACGACGTTGTAAAACGACTCATTTTCCCTCAAAGCTAACA | GTTTCTGCCTGTGAGACTGCCTTT |  | | - | |  |
| 166 | *SsaIND070TKU* | *3552e* | EG840650 | 339 | 339 | 339 | M | 3 | CACGACGTTGTAAAACGACTGGAGGCAGAAGATGCTATG | GTTTCATTGCCTCTGGAGCATTTT |  | | cathelicidin [Salmo salar] | | 3.00E-74 |
| 167 | *SsaIND167TKU* | *22042B* | DW562042 | - | - | - | NA | 2 | CACGACGTTGTAAAACGACGAACAGGCCCATAGTTGCTC | GTTTGCTATGCGGGTGTCTGCTAT |  | - | |  | |
| 168 | *SsaIND071TKU* | *3585B* | CX355566 | 339/377/378 | 339/377/378 | 339/377/378 | Dupl | 3 | CACGACGTTGTAAAACGACGAGCCCTACAAGGTGTACCG | GTTTAAGCTGCTGCTGCACTTTTA |  | interferon regulatory factor 8 [Danio rerio] | | 1.00E-67 | |
| 169 | *SsaIND168TKU* | *22201C* | EG851967 | - | - | - | NA | 2 | CACGACGTTGTAAAACGACTGGCACATTCCCTCTGGATA | GTTTATGTGAATGAAGGCGGGTAA |  | - | |  | |
| 170 | *SsaIND072TKU* | *3656H* | EG790869 | - | - | - | NA | 2 | CACGACGTTGTAAAACGACTCACAACCTCTCCACAGTCG | GTTTAATACCCCCAAGGAGCAGAC |  | LYR motif-containing protein 5 [Salmo salar] | | 4.00E-30 | |
| 171 | *SsaIND169TKU* | *22294A* | EG922089 | 317/313 | 315 | 317/315 | P | 2 | CACGACGTTGTAAAACGACGAAGGGCTCTGACAACGAAC | GTTTCCTGTATCGACTCCGTGCT |  | - | |  | |
| 172 | *SsaIND073TKU* | *3883H* | EG800682 | - | - | - | NA | 3 | CACGACGTTGTAAAACGACGCTGCTGGACAAGGAAAATG | GTTTGGTGACAAGAAGGACAAAGG |  | - | |  | |
| 173 | *SsaIND170TKU* | *22330B* | CB500508 | - | 362/364 | 364 | P | 4 | CACGACGTTGTAAAACGACTTTAGTTTGGACCCCTGTCG | GTTTCAAGTCAAGCACTACCGTCAA |  | - | |  | |
| 174 | *SsaIND074TKU* | *4043l* | EG817394 | 518 | 516 | 518 | TA P | 2 | CACGACGTTGTAAAACGACCAATCTCCAAAATCCTCCACA | GTTTAGCACAATCAAGCACACTGC |  | - | |  | |
| 175 | *SsaIND075TKU* | *4102L* | EG826294 | 297 | 297 | 294/297 | P | 3 | CACGACGTTGTAAAACGACGCATCCAGGTGAATTTCCAT | GTTTGGGTGGAGGAATGTTTCTG |  | unnamed protein product [Tetraodon nigroviridis] | | 6.00E-08 | |
| 176 | *SsaIND172TKU* | *22549A* | EG903249 | 253 | 253 | 253 | M | 2 | CACGACGTTGTAAAACGACCATGGGACTGAACACCAAGA | GTTTGGGGAATTGAGGACTGACC |  | - | |  | |
| 177 | *SsaIND173TKU* | *10240A* | CX357212 | 534 | 534 | 534 | M | 3 | CACGACGTTGTAAAACGACGAGTGGCTGGAAATCCTCAA | GTTTCTGGAACTGGATGGACTGCT |  | Mediator of RNA polymerase II transcription subunit 15 [Salmo  salar] | | 4.00E-09 | |
| 178 | *SsaIND077TKU* | *4367G* | DY712536 | 208/201 | 208/201 | 208/201 | Dupl | 8 | CACGACGTTGTAAAACGACGGAAAAGCACCACCGAATAA | GTTTCCGCTCAGGTCAAAGGTTAC |  | Claudin-6 [Salmo salar] | | 8.00E-49 | |
| 179 | *SsaIND175TKU* | *20326D* | DW581136 | - | - | - | MB | 3 | CACGACGTTGTAAAACGACTCAGAGTGGGATGCTTGGAT | GTTTGGTCCCTGTAGTGCTGGTTT |  | NADH dehydrogenase (ubiquinone) 1 alpha subcomplex, assembly factor 2 [Danio rerio] | | 2.00E-60 | |
| 180 | *SsaIND176TKU* | *20384C* | CA041230 | - | - | - | MB | 2 | CACGACGTTGTAAAACGACCTGTCCAACTCCAGCCAAAG | GTTTCGTCCATCCACAGTAGAGCA |  | similar to mannose receptor, C type 2 [Danio rerio] | | 6.00E-63 | |
| 181 | *SsaIND177TKU* | *20399D* | CB507259 | 508 | 508/511 | 508 | P | 2 | CACGACGTTGTAAAACGACGCCAGAGAGGAATGCAATGT | GTTTAAACGGAAGGGAAAAGTTGG |  | - | |  | |
| 182 | *SsaIND082TKU* | *5409S* | CB510997 | - | - | 357/359 | P | 2 | CACGACGTTGTAAAACGACTGTATGCCTTTAGCTGCATTG | GTTTGCCACGTACTCTCTGCTTCC |  | - | |  | |
| 183 | *SsaIND083TKU* | *5977B* | CA059152 | 221 | 221 | 221 | P | 6 | CACGACGTTGTAAAACGACTTTTCCTTCTCAGCCTCCTG | GTTTTTCTCTCCATCACAGCCAAA |  | proteasome (prosome, macropain) 26S subunit,  non-ATPase, 1 [Danio rerio] | | 5.00E-26 | |
| 184 | *SsaIND180TKU* | *20528B* | DY707595 | 572 | 572 | 572 | M | 6 | CACGACGTTGTAAAACGACCTGCCTACGCTGGCTACAG | GTTTATATCCCCCTGGCTGAGAGT |  | - | |  | |
| 185 | *SsaIND084TKU* | *6180S* | DW581845 | - | 478 | 478 | M | 6 | CACGACGTTGTAAAACGACTTGCATTTGGAACCACTCAA | GTTTAGCCAAAACTGCCTGTGAAG |  | - | |  | |
| 186 | *SsaIND181TKU* | *20664B* | EG893630 | 397 | 397/400 | - | P | 2 | CACGACGTTGTAAAACGACACACCACACGACGGACACTA | GTTTATGAACAGAAGGGGACAGCA | microsatellite | protein phosphatase 2, regulatory subunit B', gamma isoform [Danio rerio] | | 1.00E-24 | |
| 187 | *SsaIND085TKU* | *6241N* | EG832232 | 105/111 | 105/111 | 105 | P | 6 | CACGACGTTGTAAAACGACACACAGTTTGGGGCTGGAT | GTTTGGGAGAGATTCAGTAGGACTGT |  | WD repeat-containing protein 57 [Salmo salar] | | 3.00E-98 | |
| 188 | *SsaIND182TKU* | *20705B* | CK898265 | 190/217 | 190/217 | 190/217 | Dupl | 4 | CACGACGTTGTAAAACGACGTGGTTGAAGGGAACTGTGG | GTTTGACAGCAACACGCAGAGAGA |  | - | |  | |
| 189 | *SsaIND086TKU* | *6616C* | DW340467 | - | - | - | NA | 3 | CACGACGTTGTAAAACGACTTGTCTCAGTGACCCACCAG | GTTTACAGGGAGCCCATAAGGATT |  | zgc:158852 [Danio rerio] | | 1.00E-17 | |
| 190 | *SsaIND087TKU* | *6711M* | EG911678 | 287 | 287 | 287 | M | 2 | CACGACGTTGTAAAACGACTGCAACATACCAAATTTCAACA | GTTTGTATGGGTGCTGATGGTC |  | - | |  | |
| 191 | *SsaIND184TKU* | *20793A* | EG823717 | 476 | 472/476 | 474/476 | P | 2 | CACGACGTTGTAAAACGACGCATGTAAACTGCCCAGATG | GTTTCCGACCACAAATGTATTGC |  | - | |  | |
| 192 | *SsaIND088TKU* | *6714D* | DY691857 | - | - | - | NA | 10 | CACGACGTTGTAAAACGACACAACCTTGGACGGCAAATA | GTTTAGGCATTCTGGGTACAGTCG |  | - | |  | |
| 193 | *SsaIND089TKU* | *6856G* | DY691885 | - | - | - | NA | 2 | CACGACGTTGTAAAACGACTGGTACACGAACATTCTTAGCAG | GTTTATCAACCCTCGTTGCTGTG |  | - | |  | |
| 194 | *SsaIND186TKU* | *21237D* | DW553282 | - | - | - | NA | 3 | CACGACGTTGTAAAACGACATGATGCACAGAGGCAAGGT | GTTTGTGGCTCTCCTCACGATCC |  | PR/SET domain containing protein 8a [Danio rerio] | | 4.00E-08 | |
| 195 | *SsaIND090TKU* | *7073C* | EG833055 | 425 | 423/425 | 425 | P | 3 | CACGACGTTGTAAAACGACCAATTTCCACCAATCCCAAC | GTTTGACATGGCCTTCCATTACC |  | - | |  | |
| 196 | *SsaIND187TKU* | *21454C* | DY708576 | 224 | 224 | 224 | M | 2 | CACGACGTTGTAAAACGACCAGCCACTCCAGACTCCACT | GTTTCACTGCATGGCAATTTGTCT | microsatellite | - | |  | |
| 197 | *SsaIND091TKU* | *7116D* | CA043071 | - | - | - | NA | 7 | CACGACGTTGTAAAACGACGAATAGAACCCACCCCCAGT | GTTTACCTAACGCTGTGACCCAAC |  | - | |  | |
| 198 | *SsaIND188TKU* | *21570C* | DW547933 | 284 | 284 | 284 | M | 2 | CACGACGTTGTAAAACGACTGATGGCCAGTGGTAGAATG | GTTTCTGCTGGTCATTTTGGAGGT |  | - | |  | |
| 199 | *SsaIND189TKU* | *21651B* | DW583985 | 322/324 | 322/324 | 322/324 | P | 2 | CACGACGTTGTAAAACGACAGTCGGAACGCTGTCATTTC | GTTTCTCTGTCGGTCTCCATCTCC |  | - | |  | |
| 200 | *SsaIND093TKU* | *7319D* | EG893074 | - | - | - | NA | 7 | CACGACGTTGTAAAACGACAACATAACCACTGGGGCAAC | GTTTGGATCTATGAGCTGCCATCC |  | Group XIIA secretory phospholipase A2 precursor [Salmo salar] | | 3.00E-38 | |
| 201 | *SsaIND190TKU* | *21733A* | EG759930 | 463 | 463 | 463 | M | 3 | CACGACGTTGTAAAACGACTCAACCAAGACAGGGACAGA | GTTTGAGGAAGAGGGGGAGAAT |  | - | |  | |
| 202 | *SsaIND094TKU* | *7439A* | EG809820 | 332/465 | 332/465 | 332/465 | Dupl | 4 | CACGACGTTGTAAAACGACTCAGGGGAAATGTCTGAGGT | GTTTAGTTACCTGGAGGGGCTCAT |  | slowmo homolog 2 [Salmo salar] | | 4.00E-31 | |

NOTE P – Polymorphic; Dupl – Duplicated, MB – Multiple bands; M – Monomorphic; NA – No amplification; TA P – Trans-Atlantic polymorphism.

*To facilitate genotyping through enhancing 3`adenylation, four nucleotides (GTTT) were added to the 3`end of each reverse primer.
