# Supplementary material for: Discovery and application of insertion-deletion (INDEL) polymorphisms for QTL mapping of early life-history traits in Atlantic salmon
Source: BMC Genomics. 2010 Mar 8;11:156. doi: 10.1186/1471-2164-11-156 (PMC2838853; doi:10.1186/1471-2164-11-156)
Supplement: Additional file 2 — Information on developed 76 locus single-run INDEL panel in Atlantic salmon. Information on fluorescence labeling, primer concentrations, PCR pooling and links to alignments, INDEL motifs and GENESCAN (Burge and Karlin 1997) predictions of genes/exons are available in html format. [file 1471-2164-11-156-S2.ZIP › Additionalfile2/Genscan_output/GENSCAN output DY735378.htm]

GENSCAN output


  


### GENSCANW output for sequence 02:47:45

  
  

```
GENSCAN 1.0	Date run: 23-May-108	Time: 02:47:46

Sequence gi : 790 bp : 52.15% C+G : Isochore 3 (51 - 57 C+G%)

Parameter matrix: HumanIso.smat

Predicted genes/exons:


Gn.Ex Type S .Begin ...End .Len Fr Ph I/Ac Do/T CodRg P.... Tscr..
----- ---- - ------ ------ ---- -- -- ---- ---- ----- ----- ------

 1.01 Init +     69    709  641  2  2   64   75   903 0.861  81.49
```

Click **here** to view a PDF image of the predicted gene(s)

Click **here** for a PostScript image of the predicted gene(s)

```
Predicted peptide sequence(s):

Predicted coding sequence(s):


>gi|GENSCAN_predicted_peptide_1|214_aa
MASERSFPDSFIDDNPQKAIEELNEALEVNSDNSEWFCQRAYAHLQLKNYSCAVDDAKKA
QQLNPSLALAFMRTGMAEFHLNHFESAHAAFSQGHSLDGSDNTFQMWIKRCEEMMEMANQ
NGKENKQTPTPHIKHDWYQTESQVIVTIMVKNVPKDGVNINFDERQLSAVVKLPSGGDFS
LTVNLLHPIVPQHSTFKVLSTKVEIKMKKTEGIX

>gi|GENSCAN_predicted_CDS_1|642_bp
atggcatccgaacgcagtttccccgatagcttcatagacgacaacccacagaaagctatc
gaggaacttaatgaagcattggaagtaaactcggacaattcagaatggttctgccaacga
gcctatgcccacctccaactgaaaaactacagctgtgctgtcgatgatgccaagaaagcc
cagcagctcaaccccagtcttgctcttgccttcatgagaacaggaatggcagagttccac
ctgaaccacttcgagtcggcacatgctgccttcagtcagggacacagcctggacggctcc
gataacactttccaaatgtggatcaaacgctgtgaggagatgatggagatggccaatcag
aatggaaaggagaacaagcagacaccgacgccacacattaagcatgactggtaccagaca
gagtctcaggtcatcgtcaccatcatggtgaaaaatgtgcctaaggatggggtcaacatc
aacttcgacgagagacagttgtcggcggtggtgaagcttccgtcggggggggacttcagc
ctgactgtgaacctgctgcatcccatcgtcccccaacacagcaccttcaaggtcctctcc
actaaggtggagattaaaatgaagaagacggaggggatcagn


Explanation

Gn.Ex : gene number, exon number (for reference)
Type  : Init = Initial exon (ATG to 5' splice site)
        Intr = Internal exon (3' splice site to 5' splice site)
        Term = Terminal exon (3' splice site to stop codon)
        Sngl = Single-exon gene (ATG to stop)
        Prom = Promoter (TATA box / initation site)
        PlyA = poly-A signal (consensus: AATAAA)
S     : DNA strand (+ = input strand; - = opposite strand)
Begin : beginning of exon or signal (numbered on input strand)
End   : end point of exon or signal (numbered on input strand)
Len   : length of exon or signal (bp)
Fr    : reading frame (a forward strand codon ending at x has frame x mod 3)
Ph    : net phase of exon (exon length modulo 3)
I/Ac  : initiation signal or 3' splice site score (tenth bit units)
Do/T  : 5' splice site or termination signal score (tenth bit units)
CodRg : coding region score (tenth bit units)
P     : probability of exon (sum over all parses containing exon)
Tscr  : exon score (depends on length, I/Ac, Do/T and CodRg scores)

Comments

The SCORE of a predicted feature (e.g., exon or splice site) is a
log-odds measure of the quality of the feature based on local sequence
properties. For example, a predicted 5' splice site with
score > 100 is strong; 50-100 is moderate; 0-50 is weak; and
below 0 is poor (more than likely not a real donor site).

The PROBABILITY of a predicted exon is the estimated probability under
GENSCAN's model of genomic sequence structure that the exon is correct.
This probability depends in general on global as well as local sequence
properties, e.g., it depends on how well the exon fits with neighboring
exons.  It has been shown that predicted exons with higher probabilities
are more likely to be correct than those with lower probabilities.
```
