# Supplementary material for: Discovery and application of insertion-deletion (INDEL) polymorphisms for QTL mapping of early life-history traits in Atlantic salmon
Source: BMC Genomics. 2010 Mar 8;11:156. doi: 10.1186/1471-2164-11-156 (PMC2838853; doi:10.1186/1471-2164-11-156)
Supplement: Additional file 2 — Information on developed 76 locus single-run INDEL panel in Atlantic salmon. Information on fluorescence labeling, primer concentrations, PCR pooling and links to alignments, INDEL motifs and GENESCAN (Burge and Karlin 1997) predictions of genes/exons are available in html format. [file 1471-2164-11-156-S2.ZIP › Additionalfile2/Ind139Blast.htm]

Blast Result


|  |  |
| --- | --- |
|  | Blast 2 Sequences results |

|  |  |  |  |  |  |
| --- | --- | --- | --- | --- | --- |
| PubMed | Entrez | BLAST | OMIM | Taxonomy | Structure |

**BLAST 2 SEQUENCES RESULTS VERSION BLASTN 2.2.18 [Mar-02-2008]**


Match:
Mismatch:
gap open:
gap extension:    
x\_dropoff: 
expect:
wordsize: 
Filter 
View option 
 Standard
 Mismatch-highlighting
   
  
Masking character option 
 X for protein, n for nucleotide
 Lower case
   
Masking color option 
 Black
 Grey
 Red
   
  
Show CDS translation


---


  
 **Sequence 1**: gi|117528836|EST\_ssal\_eve\_53720 ssaleve thyroid Salmo salar cDNA Salmo salar cDNA clone ssal\_eve\_573\_077\_rev 3', mRNA sequence.  
Length = 759
(1 .. 759)
  
  
 **Sequence 2**: gi|117857261|EST\_ssal\_evf\_32118 ssalevf mixed\_tissue Salmo salar cDNA Salmo salar cDNA clone ssal\_evf\_542\_260\_rev 3', mRNA sequence.  
Length = 763
(1 .. 763)
  
  
  

|  |  |  |  |  |
| --- | --- | --- | --- | --- |
|  |  | **2** |  | **1** |

  
NOTE:Bitscore and expect value are calculated based on the size of the nr database.  
  
NOTE:If protein translation is reversed, please repeat the search with reverse strand of the query sequence.  
  

  
  
  

```
 Score = 1369 bits (712),  Expect = 0.0
 Identities = 752/760 (98%), Gaps = 7/760 (0%)
 Strand=Plus/Plus

Query  1    GCTCTGGCAGGACATTTTATTAGGTAACAGATTTGAAAGTATACAGAAAGGATAGGATTT  60
            ||||||||||||||||||||||||||||||||||||||||||||||||||||||||||||
Sbjct  1    GCTCTGGCAGGACATTTTATTAGGTAACAGATTTGAAAGTATACAGAAAGGATAGGATTT  60

Query  61   AAGTGCCATTCAGCTTAATTGTGCGTTTAAATTCTGCTATATTTTTAGACAATCTCCCTG  120
            ||||||||||||||||||||||||||||||||||||||||||||||||||||||||||||
Sbjct  61   AAGTGCCATTCAGCTTAATTGTGCGTTTAAATTCTGCTATATTTTTAGACAATCTCCCTG  120

Query  121  AGCAGCCAGTACTGACGTCAAGTAGGACTGCCCTGCCATCATCCATAAAGTCCCCAAGCC  180
            ||||||||||||||||||||||||||||||||||||||||||||||||||||||||||||
Sbjct  121  AGCAGCCAGTACTGACGTCAAGTAGGACTGCCCTGCCATCATCCATAAAGTCCCCAAGCC  180

Query  181  CGGTGGAAAAAAACAGCACACCTCTTTGCAGTCTTGAGCACATCAGAATCAGAATGATAG  240
            ||||||||||||||||||||||||||||||||||||||||||||||||      ||||||
Sbjct  181  CGGTGGAAAAAAACAGCACACCTCTTTGCAGTCTTGAGCACATCAGAA------TGATAG  234

Query  241  TGAGTAGTAAATAAAGTTAGACTTCTGTGGTTGGAGTGCTCTGGTCCCAATGGGCCTACA  300
            ||||||||||||||||||||||||||||||||||||||||||||||||||||||||||||
Sbjct  235  TGAGTAGTAAATAAAGTTAGACTTCTGTGGTTGGAGTGCTCTGGTCCCAATGGGCCTACA  294

Query  301  CCAGCTCAGAGAGCATGTTGGAAGCCAGGCTGCAGCTGAGGCCGGTGCCGTCAGGCTCCA  360
            ||||||||||||||||||||||||||||||||||||||||||||||||||||||||||||
Sbjct  295  CCAGCTCAGAGAGCATGTTGGAAGCCAGGCTGCAGCTGAGGCCGGTGCCGTCAGGCTCCA  354

Query  361  CGTTGATCTTCTTCACAACTCCATCCTCCACCACCATAGCGTACCTCTGGGAGCGCTTGT  420
            ||||||||||||||||||||||||||||||||||||||||||||||||||||||||||||
Sbjct  355  CGTTGATCTTCTTCACAACTCCATCCTCCACCACCATAGCGTACCTCTGGGAGCGCTTGT  414

Query  421  TTCCAAGCGCAGCCACAATCTGATCGTTGTCTAGCAACAGGTCCACTGCCTTAGTGAATT  480
            ||||||||||||||||||||||||||||||||||||||||||||||||||||||||||||
Sbjct  415  TTCCAAGCGCAGCCACAATCTGATCGTTGTCTAGCAACAGGTCCACTGCCTTAGTGAATT  474

Query  481  CTCCAGTAGGATCAGCTAGCATTCGCACCTTGCCTTCTGCTCCATGTTCCTTTCCCCAGG  540
            ||||||||||||||||||||||||||||||||||||||||||||||||||||||||||||
Sbjct  475  CTCCAGTAGGATCAGCTAGCATTCGCACCTTGCCTTCTGCTCCATGTTCCTTTCCCCAGG  534

Query  541  CAGCCATAACGAATGCGTCGTTAACGGAGACGCAGGCGACCTCCTGTACACCCTTGCACT  600
            ||||||||||||||||||||||||||||||||||||||||||||||||||||||||||||
Sbjct  535  CAGCCATAACGAATGCGTCGTTAACGGAGACGCAGGCGACCTCCTGTACACCCTTGCACT  594

Query  601  TCAGCTCTGCTGCCTGCTCCACAAAGCCTGGAAGGTGAGTCTTGGAACACCCAGGAGTGA  660
            ||||||||||||||||||||||||||||||||||||||||||||||||||||||||||||
Sbjct  595  TCAGCTCTGCTGCCTGCTCCACAAAGCCTGGAAGGTGAGTCTTGGAACACCCAGGAGTGA  654

Query  661  AGGCCCCTGGCACAGCAAAGAGAACTCCCTTCTTCCCCTTGAACAGCTGGTCCATAGACA  720
            ||||||||||||||||||||| ||||||||||||||||||||||||||||||||||||||
Sbjct  655  AGGCCCCTGGCACAGCAAAGAAAACTCCCTTCTTCCCCTTGAACAGCTGGTCCATAGACA  714

Query  721  CCTTATTGCCTGGCTCA-TTCTCCTGAACCTCCACAGCAG  759
            ||||||||||||||||| ||||||||||||||||||||||
Sbjct  715  CCTTATTGCCTGGCTCATTTCTCCTGAACCTCCACAGCAG  754
```

```
CPU time:     0.05 user secs.	    0.04 sys. secs	    0.09 total secs.
```
