# Supplementary material for: Discovery and application of insertion-deletion (INDEL) polymorphisms for QTL mapping of early life-history traits in Atlantic salmon
Source: BMC Genomics. 2010 Mar 8;11:156. doi: 10.1186/1471-2164-11-156 (PMC2838853; doi:10.1186/1471-2164-11-156)
Supplement: Additional file 2 — Information on developed 76 locus single-run INDEL panel in Atlantic salmon. Information on fluorescence labeling, primer concentrations, PCR pooling and links to alignments, INDEL motifs and GENESCAN (Burge and Karlin 1997) predictions of genes/exons are available in html format. [file 1471-2164-11-156-S2.ZIP › Additionalfile2/Ind1836Blast.htm]

Blast Result


|  |  |
| --- | --- |
|  | Blast 2 Sequences results |

|  |  |  |  |  |  |
| --- | --- | --- | --- | --- | --- |
| PubMed | Entrez | BLAST | OMIM | Taxonomy | Structure |

**BLAST 2 SEQUENCES RESULTS VERSION BLASTN 2.2.18 [Mar-02-2008]**


Match:
Mismatch:
gap open:
gap extension:    
x\_dropoff: 
expect:
wordsize: 
Filter 
View option 
 Standard
 Mismatch-highlighting
   
  
Masking character option 
 X for protein, n for nucleotide
 Lower case
   
Masking color option 
 Black
 Grey
 Red
   
  
Show CDS translation


---


  
 **Sequence 1**: gi|117522363|EST\_ssal\_eve\_14874 ssaleve thyroid Salmo salar cDNA Salmo salar cDNA clone ssal\_eve\_520\_077\_rev 3', mRNA sequence.  
Length = 772
(1 .. 772)
  
  
 **Sequence 2**: gi|117544508|EST\_ssal\_eve\_19718 ssaleve thyroid Salmo salar cDNA Salmo salar cDNA clone ssal\_eve\_526\_318\_rev 3', mRNA sequence.  
Length = 784
(1 .. 784)
  
  
  

|  |  |  |  |  |
| --- | --- | --- | --- | --- |
|  |  | **2** |  | **1** |

  
NOTE:Bitscore and expect value are calculated based on the size of the nr database.  
  
NOTE:If protein translation is reversed, please repeat the search with reverse strand of the query sequence.  
  

  
  
  

```
 Score = 1433 bits (745),  Expect = 0.0
 Identities = 768/777 (98%), Gaps = 9/777 (1%)
 Strand=Plus/Plus

Query  5    AGAATAACTATGTTTATTTTCAGATTGTATCAAAAGCATAACAAGCATAAAACATAGACA  64
            ||||||||||||||||||||||||||||||||||||||||||||||||||||||||||||
Sbjct  1    AGAATAACTATGTTTATTTTCAGATTGTATCAAAAGCATAACAAGCATAAAACATAGACA  60

Query  65   TACATTATTTTGTAGAGAGGTGAATACACAACTGTCCATAATACAGCTAGTTGGCCACTG  124
            ||||||||||||||||||||||||||||||||||||||||||||||||||||||||||||
Sbjct  61   TACATTATTTTGTAGAGAGGTGAATACACAACTGTCCATAATACAGCTAGTTGGCCACTG  120

Query  125  ACATAGTAAACCGGCCAAAAAGAGAAAGAGATAACGCAAAACAGTGCTTGCTTATAAACA  184
            ||||||||||||||||||||||||||||||||||||||||||||||||||||||||||||
Sbjct  121  ACATAGTAAACCGGCCAAAAAGAGAAAGAGATAACGCAAAACAGTGCTTGCTTATAAACA  180

Query  185  ATTAACATTCCAGTGTAAAAGAATTCAGTATATTTTGAGGATACAAAATGTATAAATAGC  244
            ||||||||||||||||||||||||||||||||||||||||||||||||||||||||||||
Sbjct  181  ATTAACATTCCAGTGTAAAAGAATTCAGTATATTTTGAGGATACAAAATGTATAAATAGC  240

Query  245  TCTTCATTCTCTTTTAAAATCATTTCATTATGTCTGTGCCAGTAAATGTGGTGGATTCTC  304
            ||||||||||||||||||||||||||||||||||||||||||||||||||||||||||||
Sbjct  241  TCTTCATTCTCTTTTAAAATCATTTCATTATGTCTGTGCCAGTAAATGTGGTGGATTCTC  300

Query  305  ATCTACATCTATTCATCCAATTACTCACAAAATAACTTACTGTCACAACAAGAATACAAA  364
            ||||||||||||||||||||||||||||||||||||||||||||||||||||||||||||
Sbjct  301  ATCTACATCTATTCATCCAATTACTCACAAAATAACTTACTGTCACAACAAGAATACAAA  360

Query  365  CTTTTCCATTTACATTCCAAAGCCTTTTGGGGACCAAAATTATTGTAACTGAAAGAGTTG  424
            ||||||||||||||||||||||||||||||||||||||||||||||||||||||||||||
Sbjct  361  CTTTTCCATTTACATTCCAAAGCCTTTTGGGGACCAAAATTATTGTAACTGAAAGAGTTG  420

Query  425  TAGCAGGTGGTGCAGAGATATAGGACACAAACCTTTTGCCGTAACTAAGGCACAGCAACA  484
            ||||||||||||||||||||||||||||||||||||||||||||||||||||||||||||
Sbjct  421  TAGCAGGTGGTGCAGAGATATAGGACACAAACCTTTTGCCGTAACTAAGGCACAGCAACA  480

Query  485  TCTCAGTTCAATACAATCCCTTATACATCATCAAACATCAGCAGCAGTGTGTCCTCTCCA  544
            ||||||||||||||||||||||||||||||||||||||||||||||||||||||||||||
Sbjct  481  TCTCAGTTCAATACAATCCCTTATACATCATCAAACATCAGCAGCAGTGTGTCCTCTCCA  540

Query  545  CAACCCTCTACTCTCCGAGGCCTGATTCCTAACTTGATACCAATCCATAAAAAATTCCCA  604
            ||||||||||||||||||||||||||||||||||||||||||||||||||||||||||||
Sbjct  541  CAACCCTCTACTCTCCGAGGCCTGATTCCTAACTTGATACCAATCCATAAAAAATTCCCA  600

Query  605  T---------TGCCCAATTTACACAAAACCTAAGTTACGCAAGCTTATGTATCTCAATTT  655
            |         ||||||||||||||||||||||||||||||||||||||||||||||||||
Sbjct  601  TTGACGCCAATGCCCAATTTACACAAAACCTAAGTTACGCAAGCTTATGTATCTCAATTT  660

Query  656  AGGATTCGGCCCTCAGCCAATGGCAGTCGGAACTGAGCATGGCCTGGGTGGGGCTCCACT  715
            ||||||||||||||||||||||||||||||||||||||||||||||||||||||||||||
Sbjct  661  AGGATTCGGCCCTCAGCCAATGGCAGTCGGAACTGAGCATGGCCTGGGTGGGGCTCCACT  720

Query  716  GTAGATCTGTTTTGGTTGGGGCTCAGTGGAACCATAAGCTAACCTGAGTGAGCTTAG  772
            |||||||||||||||||||||||||||||||||||||||||||||||||||||||||
Sbjct  721  GTAGATCTGTTTTGGTTGGGGCTCAGTGGAACCATAAGCTAACCTGAGTGAGCTTAG  777
```

```
CPU time:     0.05 user secs.	    0.03 sys. secs	    0.08 total secs.
```
