# Supplementary material for: Discovery and application of insertion-deletion (INDEL) polymorphisms for QTL mapping of early life-history traits in Atlantic salmon
Source: BMC Genomics. 2010 Mar 8;11:156. doi: 10.1186/1471-2164-11-156 (PMC2838853; doi:10.1186/1471-2164-11-156)
Supplement: Additional file 2 — Information on developed 76 locus single-run INDEL panel in Atlantic salmon. Information on fluorescence labeling, primer concentrations, PCR pooling and links to alignments, INDEL motifs and GENESCAN (Burge and Karlin 1997) predictions of genes/exons are available in html format. [file 1471-2164-11-156-S2.ZIP › Additionalfile2/Ind1921Blast.htm]

Blast Result


|  |  |
| --- | --- |
|  | Blast 2 Sequences results |

|  |  |  |  |  |  |
| --- | --- | --- | --- | --- | --- |
| PubMed | Entrez | BLAST | OMIM | Taxonomy | Structure |

**BLAST 2 SEQUENCES RESULTS VERSION BLASTN 2.2.18 [Mar-02-2008]**


Match:
Mismatch:
gap open:
gap extension:    
x\_dropoff: 
expect:
wordsize: 
Filter 
View option 
 Standard
 Mismatch-highlighting
   
  
Masking character option 
 X for protein, n for nucleotide
 Lower case
   
Masking color option 
 Black
 Grey
 Red
   
  
Show CDS translation


---


  
 **Sequence 1**: gi|29319893|ssalob507116 reproductive Salmo salar cDNA, mRNA sequence.  
Length = 662
(1 .. 662)
  
  
 **Sequence 2**: gi|117835170|EST\_ssal\_evf\_10236 ssalevf mixed\_tissue Salmo salar cDNA Salmo salar cDNA clone ssal\_evf\_512\_022\_rev 3', mRNA sequence.  
Length = 589
(1 .. 589)
  
  
  

|  |  |  |  |  |
| --- | --- | --- | --- | --- |
|  |  | **2** |  | **1** |

  
NOTE:Bitscore and expect value are calculated based on the size of the nr database.  
  
NOTE:If protein translation is reversed, please repeat the search with reverse strand of the query sequence.  
  

  
  
  

```
 Score = 1102 bits (573),  Expect = 0.0
 Identities = 588/593 (99%), Gaps = 4/593 (0%)
 Strand=Plus/Plus

Query  1    GGGAAATCATTTAAATGTATTTATTGTATTTTTGTTGAAATAAATCAAAATGGTTTAATT  60
            ||||||||||||||||||||||||||||||||||||||||||||||||||||||||||||
Sbjct  1    GGGAAATCATTTAAATGTATTTATTGTATTTTTGTTGAAATAAATCAAAATGGTTTAATT  60

Query  61   TTAATTGTGAAAAAAGTACATTGAGATTCATGACAGCATATGTGGGAGACATTCTCTGTA  120
            ||||||||||||||||||||||||||||||||||||||||||||||||||||||||||||
Sbjct  61   TTAATTGTGAAAAAAGTACATTGAGATTCATGACAGCATATGTGGGAGACATTCTCTGTA  120

Query  121  GTATCTATGTAACGCTTTTGATTAGGCACGCCATAAAGAATGGATACTGCACATTAAGCA  180
            ||||||||||||||||||||||||||||||||||||||||||||||||||||||||||||
Sbjct  121  GTATCTATGTAACGCTTTTGATTAGGCACGCCATAAAGAATGGATACTGCACATTAAGCA  180

Query  181  ATTTTCCAATACAGCATAATATCTTCAGGTAGTTCTCCCTTCGATCAAACTTCTAAAGAT  240
            ||||||||||||||||||||||||||||||||||||||||||||||||||||||||||||
Sbjct  181  ATTTTCCAATACAGCATAATATCTTCAGGTAGTTCTCCCTTCGATCAAACTTCTAAAGAT  240

Query  241  TCCGGTTCTGATATCTTAACTGCCAAATTTTATTTAAAGCAATAGTCAAGTTGAAGGTTA  300
            ||||||||||||||||||||||||||||||||||||||||||||||||||||||||||||
Sbjct  241  TCCGGTTCTGATATCTTAACTGCCAAATTTTATTTAAAGCAATAGTCAAGTTGAAGGTTA  300

Query  301  CATTTTCCCATATATTACAAAAACAAGTGGAGATTTTGTGCATCGAAGTTAAAGGAACAT  360
            ||||||||||||||||||||||||||||||||||||||||| ||||||||||||||||||
Sbjct  301  CATTTTCCCATATATTACAAAAACAAGTGGAGATTTTGTGCGTCGAAGTTAAAGGAACAT  360

Query  361  AGAAAAACACTGGCTGATGTATGTATGTATGAGGAGACCACTACATTCCTGTGTGTAGTA  420
            ||||||||||||||||    ||||||||||||||||||||||||||||||||||||||||
Sbjct  361  AGAAAAACACTGGCTG----ATGTATGTATGAGGAGACCACTACATTCCTGTGTGTAGTA  416

Query  421  ATGACGTGATGGGTCTACTGCAGTTGAAGTGGACACGGGCATCGAAGCACAGATGGTGAA  480
            ||||||||||||||||||||||||||||||||||||||||||||||||||||||||||||
Sbjct  417  ATGACGTGATGGGTCTACTGCAGTTGAAGTGGACACGGGCATCGAAGCACAGATGGTGAA  476

Query  481  CAGCAAACATCAGTCATCAGGACAGCAAACGCATGCTCAAGGTGGTGTAACCACAAACAA  540
            ||||||||||||||||||||||||||||||||||||||||||||||||||||||||||||
Sbjct  477  CAGCAAACATCAGTCATCAGGACAGCAAACGCATGCTCAAGGTGGTGTAACCACAAACAA  536

Query  541  GTTGAGTGGCCTGTGAAACTGAAGCTAAACCCACAACACTACCGTATGGATTC  593
            |||||||||||||||||||||||||||||||||||||||||||||||||||||
Sbjct  537  GTTGAGTGGCCTGTGAAACTGAAGCTAAACCCACAACACTACCGTATGGATTC  589
```

```
CPU time:     0.05 user secs.	    0.04 sys. secs	    0.09 total secs.
```
