# Supplementary material for: Discovery and application of insertion-deletion (INDEL) polymorphisms for QTL mapping of early life-history traits in Atlantic salmon
Source: BMC Genomics. 2010 Mar 8;11:156. doi: 10.1186/1471-2164-11-156 (PMC2838853; doi:10.1186/1471-2164-11-156)
Supplement: Additional file 2 — Information on developed 76 locus single-run INDEL panel in Atlantic salmon. Information on fluorescence labeling, primer concentrations, PCR pooling and links to alignments, INDEL motifs and GENESCAN (Burge and Karlin 1997) predictions of genes/exons are available in html format. [file 1471-2164-11-156-S2.ZIP › Additionalfile2/Ind2070Blast.htm]

Blast Result


|  |  |
| --- | --- |
|  | Blast 2 Sequences results |

|  |  |  |  |  |  |
| --- | --- | --- | --- | --- | --- |
| PubMed | Entrez | BLAST | OMIM | Taxonomy | Structure |

**BLAST 2 SEQUENCES RESULTS VERSION BLASTN 2.2.18 [Mar-02-2008]**


Match:
Mismatch:
gap open:
gap extension:    
x\_dropoff: 
expect:
wordsize: 
Filter 
View option 
 Standard
 Mismatch-highlighting
   
  
Masking character option 
 X for protein, n for nucleotide
 Lower case
   
Masking color option 
 Black
 Grey
 Red
   
  
Show CDS translation


---


  
 **Sequence 1**: gi|45327998|SGP161832 Atlantic salmon Testis cDNA library Salmo salar cDNA clone MG4-0363 5', mRNA sequence.  
Length = 519
(1 .. 519)
  
  
 **Sequence 2**: gi|85055446|EST\_ssal\_rgb2\_48043 rgb2 Salmo salar cDNA clone ssal\_rgb2\_577\_230\_rev 5', mRNA sequence.  
Length = 726
(1 .. 726)
  
  
  

|  |  |  |  |  |
| --- | --- | --- | --- | --- |
|  |  | **2** |  | **1** |

  
NOTE:Bitscore and expect value are calculated based on the size of the nr database.  
  
NOTE:If protein translation is reversed, please repeat the search with reverse strand of the query sequence.  
  

  
  
  

```
 Score =  954 bits (496),  Expect = 0.0
 Identities = 513/519 (98%), Gaps = 4/519 (0%)
 Strand=Plus/Plus

Query  1    AAGGGGGCTTGATCGAGAAGTGAAGCTAGGGTCAATATGTCACCGTGCACAACGGCCCCA  60
            ||||||||||||||||||||||||||||||||||||||||||||||||||||||||||||
Sbjct  170  AAGGGGGCTTGATCGAGAAGTGAAGCTAGGGTCAATATGTCACCGTGCACAACGGCCCCA  229

Query  61   CTGCGGAACAGCAGGAAGGAGCGTTCGTGGACACTCGCTCACACACACCACACACACTTG  120
            |||||||||||||||||||||||||||||||||||||||||| |||||||||||||||||
Sbjct  230  CTGCGGAACAGCAGGAAGGAGCGTTCGTGGACACTCGCTCACGCACACCACACACACTTG  289

Query  121  TGATGACATAGTCAGGGGGGCCATTGCTAGCCATATTCCAACCTTTTTTCCCTCGTCTTT  180
            ||||||||||||||||||||||||||||||||||||||||||||||||||||||||||||
Sbjct  290  TGATGACATAGTCAGGGGGGCCATTGCTAGCCATATTCCAACCTTTTTTCCCTCGTCTTT  349

Query  181  TCTTTTATGAAGCCTTTATTAATGGCAGTGTGTGTTTATGCATGTTTATATTACTGGTGG  240
            ||||||||||||||||||||||||||||||||||||||||||||||||||||||||||||
Sbjct  350  TCTTTTATGAAGCCTTTATTAATGGCAGTGTGTGTTTATGCATGTTTATATTACTGGTGG  409

Query  241  TTGAAGGGAACTGTGGAGTATAACAACAAAATTGTGGAGGTAATTGGAATGGCTCTATGG  300
            ||||||||||||||||||||||||||||||||||||||||||||||||||||||||||||
Sbjct  410  TTGAAGGGAACTGTGGAGTATAACAACAAAATTGTGGAGGTAATTGGAATGGCTCTATGG  469

Query  301  GCGAGTTAAGTTTGTTAATGTGTCGAAATGACTCTCCATCAATCTCTCACTGTTTGTTAG  360
            |||||||||    |||||||||||||||||||||||||||||||||||||||||||||||
Sbjct  470  GCGAGTTAA----GTTAATGTGTCGAAATGACTCTCCATCAATCTCTCACTGTTTGTTAG  525

Query  361  TCTCACTACCCTCTGTAGTATAGAATCTCTCTGCGTGTTGCTGTCTCATTCTGGTTTCAT  420
            ||||||||||||||||||||||||||||||||||||||||||||||||||||||||||||
Sbjct  526  TCTCACTACCCTCTGTAGTATAGAATCTCTCTGCGTGTTGCTGTCTCATTCTGGTTTCAT  585

Query  421  TCTTTCCAAGTGACTCATAGACGGAGCTGTAACGTATTCCTGTACCAAAACGACTGGTCC  480
            ||||||||||||||||||||| ||||||||||||||||||||||||||||||||||||||
Sbjct  586  TCTTTCCAAGTGACTCATAGAAGGAGCTGTAACGTATTCCTGTACCAAAACGACTGGTCC  645

Query  481  CACAGCAGATCATGTCAAACTTTCCACATAGGTTACGTT  519
            |||||||||||||||||||||||||||||||||||||||
Sbjct  646  CACAGCAGATCATGTCAAACTTTCCACATAGGTTACGTT  684
```

```
CPU time:     0.05 user secs.	    0.04 sys. secs	    0.09 total secs.
```
