# Supplementary material for: Discovery and application of insertion-deletion (INDEL) polymorphisms for QTL mapping of early life-history traits in Atlantic salmon
Source: BMC Genomics. 2010 Mar 8;11:156. doi: 10.1186/1471-2164-11-156 (PMC2838853; doi:10.1186/1471-2164-11-156)
Supplement: Additional file 2 — Information on developed 76 locus single-run INDEL panel in Atlantic salmon. Information on fluorescence labeling, primer concentrations, PCR pooling and links to alignments, INDEL motifs and GENESCAN (Burge and Karlin 1997) predictions of genes/exons are available in html format. [file 1471-2164-11-156-S2.ZIP › Additionalfile2/Ind2130Blast.htm]

Blast Result


|  |  |
| --- | --- |
|  | Blast 2 Sequences results |

|  |  |  |  |  |  |
| --- | --- | --- | --- | --- | --- |
| PubMed | Entrez | BLAST | OMIM | Taxonomy | Structure |

**BLAST 2 SEQUENCES RESULTS VERSION BLASTN 2.2.18 [Mar-02-2008]**


Match:
Mismatch:
gap open:
gap extension:    
x\_dropoff: 
expect:
wordsize: 
Filter 
View option 
 Standard
 Mismatch-highlighting
   
  
Masking character option 
 X for protein, n for nucleotide
 Lower case
   
Masking color option 
 Black
 Grey
 Red
   
  
Show CDS translation


---


  
 **Sequence 1**: gi|117566257|EST\_ssal\_evf\_45411 ssalevf mixed\_tissue Salmo salar cDNA Salmo salar cDNA clone ssal\_evf\_560\_266\_fwd 5', mRNA sequence.  
Length = 716
(1 .. 716)
  
  
 **Sequence 2**: gi|117568693|EST\_ssal\_evf\_47603 ssalevf mixed\_tissue Salmo salar cDNA Salmo salar cDNA clone ssal\_evf\_563\_243\_rev 3', mRNA sequence.  
Length = 565
(1 .. 565)
  
  
  

|  |  |  |  |  |
| --- | --- | --- | --- | --- |
|  |  | **2** |  | **1** |

  
NOTE:Bitscore and expect value are calculated based on the size of the nr database.  
  
NOTE:If protein translation is reversed, please repeat the search with reverse strand of the query sequence.  
  

  
  
  

```
 Score =  885 bits (460),  Expect = 0.0
 Identities = 479/486 (98%), Gaps = 7/486 (1%)
 Strand=Plus/Minus

Query  231  GGAACTGTCAATCAATCCCGGTGGAGTCTTGACCGCGCGCTCACAGTAAACACTGTCCAT  290
            ||||||||||||||||||||||||||||||||||||||||||||||||||||||||||| 
Sbjct  562  GGAACTGTCAATCAATCCCGGTGGAGTCTTGACCGCGCGCTCACAGTAAACACTGTCCA-  504

Query  291  ATGGTTCAGTTTACGCGCCACAGCGACGACGACTGTCGTACGTTTAGGGGGAGGCTTAAT  350
                  ||||||||||||||||||||||||||||||||||||||||||||||||||||||
Sbjct  503  ------CAGTTTACGCGCCACAGCGACGACGACTGTCGTACGTTTAGGGGGAGGCTTAAT  450

Query  351  GGAACCAATGTGAACAGAGTCAGTGCATCCATCTGTCCTGGAGATTGTGTTTCTGCTCAG  410
            ||||||||||||||||||||||||||||||||||||||||||||||||||||||||||||
Sbjct  449  GGAACCAATGTGAACAGAGTCAGTGCATCCATCTGTCCTGGAGATTGTGTTTCTGCTCAG  390

Query  411  AGAGGTATTCAACACTCATCAATCCGTCAGTGGAAATGTGGCAGGCAATAACTAGGACAT  470
            ||||||||||||||||||||||||||||||||||||||||||||||||||||||||||||
Sbjct  389  AGAGGTATTCAACACTCATCAATCCGTCAGTGGAAATGTGGCAGGCAATAACTAGGACAT  330

Query  471  CCTGTCTCTTCTTGCTAAATGTGAACTGACACAGTCAGGAGGCATCTAAAACCCAGGCGG  530
            ||||||||||||||||||||||||||||||||||||||||||||||||||||||||||||
Sbjct  329  CCTGTCTCTTCTTGCTAAATGTGAACTGACACAGTCAGGAGGCATCTAAAACCCAGGCGG  270

Query  531  TACTAATATGTATGTTCTTACAGGCTCTTATGGGCCCAGACTCTTGCAGCGATCAGAATC  590
            ||||||||||||||||||||||||||||||||||||||||||||||||||||||||||||
Sbjct  269  TACTAATATGTATGTTCTTACAGGCTCTTATGGGCCCAGACTCTTGCAGCGATCAGAATC  210

Query  591  GTTGCCATTAACCAGTCATTTCAAATGAACCGAGAGGTCAGGCCTGTGTTGTTCAGTAAC  650
            ||||||||||||||||||||||||||||||||||||||||||||||||||||||||||||
Sbjct  209  GTTGCCATTAACCAGTCATTTCAAATGAACCGAGAGGTCAGGCCTGTGTTGTTCAGTAAC  150

Query  651  TCATCATATGCCTAATATATGGTAATTGCCATTTTTAAATGACTGGTTGACTGTGACTTG  710
            ||||||||||||||||||||||||||||||||||||||||||||||||||||||||||||
Sbjct  149  TCATCATATGCCTAATATATGGTAATTGCCATTTTTAAATGACTGGTTGACTGTGACTTG  90

Query  711  GTGATT  716
            ||||||
Sbjct  89   GTGATT  84
```

```
CPU time:     0.05 user secs.	    0.04 sys. secs	    0.09 total secs.
```
