# Supplementary material for: Discovery and application of insertion-deletion (INDEL) polymorphisms for QTL mapping of early life-history traits in Atlantic salmon
Source: BMC Genomics. 2010 Mar 8;11:156. doi: 10.1186/1471-2164-11-156 (PMC2838853; doi:10.1186/1471-2164-11-156)
Supplement: Additional file 2 — Information on developed 76 locus single-run INDEL panel in Atlantic salmon. Information on fluorescence labeling, primer concentrations, PCR pooling and links to alignments, INDEL motifs and GENESCAN (Burge and Karlin 1997) predictions of genes/exons are available in html format. [file 1471-2164-11-156-S2.ZIP › Additionalfile2/Ind2231Blast.htm]

Blast Result


|  |  |
| --- | --- |
|  | Blast 2 Sequences results |

|  |  |  |  |  |  |
| --- | --- | --- | --- | --- | --- |
| PubMed | Entrez | BLAST | OMIM | Taxonomy | Structure |

**BLAST 2 SEQUENCES RESULTS VERSION BLASTN 2.2.18 [Mar-02-2008]**


Match:
Mismatch:
gap open:
gap extension:    
x\_dropoff: 
expect:
wordsize: 
Filter 
View option 
 Standard
 Mismatch-highlighting
   
  
Masking character option 
 X for protein, n for nucleotide
 Lower case
   
Masking color option 
 Black
 Grey
 Red
   
  
Show CDS translation


---


  
 **Sequence 1**: gi|117427206|EST\_ssal\_sjb\_7893 ssalsjb mixed\_tissue Salmo salar cDNA Salmo salar cDNA clone ssal\_sjb\_016\_226\_rev 5', mRNA sequence.  
Length = 763
(1 .. 763)
  
  
 **Sequence 2**: gi|117485007|EST\_ssal\_evd\_19481 ssalevd thymus Salmo salar cDNA Salmo salar cDNA clone ssal\_evd\_525\_016\_rev 3', mRNA sequence.  
Length = 737
(1 .. 737)
  
  
  

|  |  |  |  |  |
| --- | --- | --- | --- | --- |
|  |  | **2** |  | **1** |

  
NOTE:Bitscore and expect value are calculated based on the size of the nr database.  
  
NOTE:If protein translation is reversed, please repeat the search with reverse strand of the query sequence.  
  

  
  
  

```
 Score = 1371 bits (713),  Expect = 0.0
 Identities = 730/736 (99%), Gaps = 2/736 (0%)
 Strand=Plus/Plus

Query  2    CATCAAGGAACATTTATTTTTCTTATTCTGTAATGAAATACATACTGTAAGTGCAAAGAG  61
            ||||||||||||||||||||||||||||||||||||||||||||||||||||||||||||
Sbjct  2    CATCAAGGAACATTTATTTTTCTTATTCTGTAATGAAATACATACTGTAAGTGCAAAGAG  61

Query  62   TTAGTCCAGGGTCTAAGAACTACATTGAGTATAGTTGGACAGTTAATTCAAGCAAATTAA  121
            ||||||||||||||||||||||||||||||||||||||||||||||||||| ||||||||
Sbjct  62   TTAGTCCAGGGTCTAAGAACTACATTGAGTATAGTTGGACAGTTAATTCAAACAAATTAA  121

Query  122  ATATCATCAGATATTCCTTGTCTTCATAGTAGTCAAACAACGGTACCTAAAACACCAACT  181
            ||||||||||||||||||||||||||||||||||||||||||||||||||||||||| ||
Sbjct  122  ATATCATCAGATATTCCTTGTCTTCATAGTAGTCAAACAACGGTACCTAAAACACCAGCT  181

Query  182  TGTGTATAGTTCCTTATAGTTATACAAAAGCAAAAACCCTACAATTACCACATCTAAAAC  241
            ||||||||||||||||||||||||||||||||||||||||||||||||||||||||||||
Sbjct  182  TGTGTATAGTTCCTTATAGTTATACAAAAGCAAAAACCCTACAATTACCACATCTAAAAC  241

Query  242  ACATATTGTCAAATTATTTTGTGCTTGAAAACACCAAAAGGAAATCATTTTATATATATT  301
            ||||||||||||||||||||||||||||||||||||||||||||||||||||||||||||
Sbjct  242  ACATATTGTCAAATTATTTTGTGCTTGAAAACACCAAAAGGAAATCATTTTATATATATT  301

Query  302  GTCATATACTGAGAGAAGCCCCACCAGCAAACTCGAACATGAAGCGGTATTTAACATTGG  361
            ||||||||||||||||||||||||||||||||||||||||||||||||||||||||||||
Sbjct  302  GTCATATACTGAGAGAAGCCCCACCAGCAAACTCGAACATGAAGCGGTATTTAACATTGG  361

Query  362  CAGCAGAGCATTTCTGTAGGCATCTCTCTTTTTTGTGCCAAATCTTAGTTTGTTGTAAGC  421
            ||||||||||||||||||||||||||||||||||||||||||||||||||||||||||||
Sbjct  362  CAGCAGAGCATTTCTGTAGGCATCTCTCTTTTTTGTGCCAAATCTTAGTTTGTTGTAAGC  421

Query  422  GCAGCAAAAAAACGTAGCATGAAAAACTGCACAAAATATATCAAATATTTACTCAAGGCT  481
            ||||||||||||||||||||||||||||||||||||||||||||||||||||||||||||
Sbjct  422  GCAGCAAAAAAACGTAGCATGAAAAACTGCACAAAATATATCAAATATTTACTCAAGGCT  481

Query  482  TGACGTCATGCAC--TTAACAAGCAACACAAAAGAAAAAAAGGCTGGTTCTTTTTGTTCA  539
            |||||||||||||  |||||||||||||||||||||||||||||||||||||||||||||
Sbjct  482  TGACGTCATGCACTTTTAACAAGCAACACAAAAGAAAAAAAGGCTGGTTCTTTTTGTTCA  541

Query  540  GAATTGTGTACTTGTCTACCTAGACATAGGATCTTGAACATGAGTGATTTACTTTGTGTT  599
            |||||||||||||||||||||||||||| |||||||||||||||||||||||||||||||
Sbjct  542  GAATTGTGTACTTGTCTACCTAGACATAAGATCTTGAACATGAGTGATTTACTTTGTGTT  601

Query  600  AACCGTACTAGACAGTTTTGGGGTAAGGGCTTCTTCAGTGGTTGGTTTTAGAGAACACCT  659
            ||||||||||||||||||||||||||||||||||||||||||||||||||||||||||||
Sbjct  602  AACCGTACTAGACAGTTTTGGGGTAAGGGCTTCTTCAGTGGTTGGTTTTAGAGAACACCT  661

Query  660  AGATGAAATGGTACTACCCCACAGATAATGACAAGGCTCTCCAACAATGATCAAGCTGTC  719
            ||||||||||||||||||||||||||||||||||||||||| ||||||||||||||||||
Sbjct  662  AGATGAAATGGTACTACCCCACAGATAATGACAAGGCTCTCTAACAATGATCAAGCTGTC  721

Query  720  TGAGCTGGACCTCGCT  735
            ||||||||||||||||
Sbjct  722  TGAGCTGGACCTCGCT  737
```

```
CPU time:     0.05 user secs.	    0.03 sys. secs	    0.08 total secs.
```
