# Supplementary material for: Discovery and application of insertion-deletion (INDEL) polymorphisms for QTL mapping of early life-history traits in Atlantic salmon
Source: BMC Genomics. 2010 Mar 8;11:156. doi: 10.1186/1471-2164-11-156 (PMC2838853; doi:10.1186/1471-2164-11-156)
Supplement: Additional file 2 — Information on developed 76 locus single-run INDEL panel in Atlantic salmon. Information on fluorescence labeling, primer concentrations, PCR pooling and links to alignments, INDEL motifs and GENESCAN (Burge and Karlin 1997) predictions of genes/exons are available in html format. [file 1471-2164-11-156-S2.ZIP › Additionalfile2/Ind2330Blast.htm]

Blast Result


|  |  |
| --- | --- |
|  | Blast 2 Sequences results |

|  |  |  |  |  |  |
| --- | --- | --- | --- | --- | --- |
| PubMed | Entrez | BLAST | OMIM | Taxonomy | Structure |

**BLAST 2 SEQUENCES RESULTS VERSION BLASTN 2.2.18 [Mar-02-2008]**


Match:
Mismatch:
gap open:
gap extension:    
x\_dropoff: 
expect:
wordsize: 
Filter 
View option 
 Standard
 Mismatch-highlighting
   
  
Masking character option 
 X for protein, n for nucleotide
 Lower case
   
Masking color option 
 Black
 Grey
 Red
   
  
Show CDS translation


---


  
 **Sequence 1**: gi|117542091|EST\_ssal\_eve\_34375 ssaleve thyroid Salmo salar cDNA Salmo salar cDNA clone ssal\_eve\_546\_264\_fwd 5', mRNA sequence.  
Length = 758
(1 .. 758)
  
  
 **Sequence 2**: gi|117502058|EST\_ssal\_eve\_45761 ssaleve thyroid Salmo salar cDNA Salmo salar cDNA clone ssal\_eve\_562\_096\_fwd 5', mRNA sequence.  
Length = 749
(1 .. 749)
  
  
  

|  |  |  |  |  |
| --- | --- | --- | --- | --- |
|  |  | **2** |  | **1** |

  
NOTE:Bitscore and expect value are calculated based on the size of the nr database.  
  
NOTE:If protein translation is reversed, please repeat the search with reverse strand of the query sequence.  
  

  
  
  

```
 Score = 1377 bits (716),  Expect = 0.0
 Identities = 741/751 (98%), Gaps = 7/751 (0%)
 Strand=Plus/Plus

Query  8    CTCTTCAGTTCAAGCTAAATGCAAAGGTGGACAAACCGCATACGATGAACCCTGCCCAAC  67
            ||||||||||||||||||||||||||||||||||||||||||||||||||||||||||||
Sbjct  6    CTCTTCAGTTCAAGCTAAATGCAAAGGTGGACAAACCGCATACGATGAACCCTGCCCAAC  65

Query  68   CCAATATGGCTGCATTACTACTGCAGAATAGCCCAATGATATCTTCTGATCAGACCCCAG  127
            ||||||||||||||||||||||||||||||||||||||||||||||||||||||||||||
Sbjct  66   CCAATATGGCTGCATTACTACTGCAGAATAGCCCAATGATATCTTCTGATCAGACCCCAG  125

Query  128  ACGGTGGATTAACAAATGGATATGCAGAAGGGTTAGTCAATCGGAAGACCCTATAAAATG  187
            ||||||||||||||||||||||| ||||||||||||||||||||||||||||||||||||
Sbjct  126  ACGGTGGATTAACAAATGGATATACAGAAGGGTTAGTCAATCGGAAGACCCTATAAAATG  185

Query  188  GAATTAGAATGAACCAGTGGAAGTTCTAATTGGAGGTTTATAACATCAGATGGCTTGAGA  247
            ||||||||||||||||||||||||||||||||||||||||||||||||||||||||||||
Sbjct  186  GAATTAGAATGAACCAGTGGAAGTTCTAATTGGAGGTTTATAACATCAGATGGCTTGAGA  245

Query  248  ATTAACCACTGAACATTCTGAAGGTCTCGTACCAACTGAAGTGGTAACGACCCAATCACC  307
            ||||||||||||||||||||||||||||||||||||||||||||||||||||||||||||
Sbjct  246  ATTAACCACTGAACATTCTGAAGGTCTCGTACCAACTGAAGTGGTAACGACCCAATCACC  305

Query  308  TTGGGATTGAACGGCTGGTCTTGCAACTGCCTCTGGGTGAAATGACTGGCATTGACCAAT  367
            ||||||||||||||||||||||||||||||||||||||||||||||||||||||||||||
Sbjct  306  TTGGGATTGAACGGCTGGTCTTGCAACTGCCTCTGGGTGAAATGACTGGCATTGACCAAT  365

Query  368  TACACTTCAACGTTGATATACCCGTAATCAGGGTTAAGGTCTTGCTAAAAGATGGCTGAG  427
            ||||||||||||||||||||||||||||||||||||||||||||||||||||||||||||
Sbjct  366  TACACTTCAACGTTGATATACCCGTAATCAGGGTTAAGGTCTTGCTAAAAGATGGCTGAG  425

Query  428  GCCTGTCATACACCATAAGGGCTTTCTCTTGCCAGCATAGCATCAATCACATTAACTGTT  487
            ||||||||||||||||||||||||||||||||||||||||||||||||||||||||||||
Sbjct  426  GCCTGTCATACACCATAAGGGCTTTCTCTTGCCAGCATAGCATCAATCACATTAACTGTT  485

Query  488  ACACTCTAGTGGGAATTCCACTAAGGGTCTGTATGTAGCTGCTGCTCAAATGGATAAGGC  547
            ||||||||||||||||||||||||| ||||||||||||||||||||||||||||||||||
Sbjct  486  ACACTCTAGTGGGAATTCCACTAAGCGTCTGTATGTAGCTGCTGCTCAAATGGATAAGGC  545

Query  548  CTACATCCTTCCATGCACACTGTTCACGATTTTCAATGAAAGTGAGGTTTGTAAGATGGC  607
            |||||||||||||||||       ||||||||||||||||||||||||||||||||||||
Sbjct  546  CTACATCCTTCCATGCA-------CACGATTTTCAATGAAAGTGAGGTTTGTAAGATGGC  598

Query  608  TAAATAAAGAAATTATTTGTGTCCTATATCCCTTCCCATGAGCCAGGTTGACAACTACTT  667
            ||||||||||||||||||||||||||||||||||||||||||||||||||||||||||||
Sbjct  599  TAAATAAAGAAATTATTTGTGTCCTATATCCCTTCCCATGAGCCAGGTTGACAACTACTT  658

Query  668  ATGTATTGTGTGGCAGACTTACAGTGATGGGAAAATATTTTGCTCTATACTTGTAAAGCC  727
            ||||||||||||||||||||||||||||||||||||||||||||||||||||||||||||
Sbjct  659  ATGTATTGTGTGGCAGACTTACAGTGATGGGAAAATATTTTGCTCTATACTTGTAAAGCC  718

Query  728  CTTTGACTAAAATAAACATGAATTAACCCTG  758
            |||||||||||||||| ||||||||||||||
Sbjct  719  CTTTGACTAAAATAAAGATGAATTAACCCTG  749
```

```
CPU time:     0.05 user secs.	    0.03 sys. secs	    0.08 total secs.
```
