# Supplementary material for: Discovery and application of insertion-deletion (INDEL) polymorphisms for QTL mapping of early life-history traits in Atlantic salmon
Source: BMC Genomics. 2010 Mar 8;11:156. doi: 10.1186/1471-2164-11-156 (PMC2838853; doi:10.1186/1471-2164-11-156)
Supplement: Additional file 2 — Information on developed 76 locus single-run INDEL panel in Atlantic salmon. Information on fluorescence labeling, primer concentrations, PCR pooling and links to alignments, INDEL motifs and GENESCAN (Burge and Karlin 1997) predictions of genes/exons are available in html format. [file 1471-2164-11-156-S2.ZIP › Additionalfile2/Ind2377Blast.htm]

Blast Result


|  |  |
| --- | --- |
|  | Blast 2 Sequences results |

|  |  |  |  |  |  |
| --- | --- | --- | --- | --- | --- |
| PubMed | Entrez | BLAST | OMIM | Taxonomy | Structure |

**BLAST 2 SEQUENCES RESULTS VERSION BLASTN 2.2.18 [Mar-02-2008]**


Match:
Mismatch:
gap open:
gap extension:    
x\_dropoff: 
expect:
wordsize: 
Filter 
View option 
 Standard
 Mismatch-highlighting
   
  
Masking character option 
 X for protein, n for nucleotide
 Lower case
   
Masking color option 
 Black
 Grey
 Red
   
  
Show CDS translation


---


  
 **Sequence 1**: gi|117459815|EST\_ssal\_evd\_12150 ssalevd thymus Salmo salar cDNA Salmo salar cDNA clone ssal\_evd\_515\_042\_rev 3', mRNA sequence.  
Length = 583
(1 .. 583)
  
  
 **Sequence 2**: gi|24344918|ssalpha501101 gut Salmo salar cDNA, mRNA sequence.  
Length = 554
(1 .. 554)
  
  
  

|  |  |  |  |  |
| --- | --- | --- | --- | --- |
|  |  | **2** |  | **1** |

  
NOTE:Bitscore and expect value are calculated based on the size of the nr database.  
  
NOTE:If protein translation is reversed, please repeat the search with reverse strand of the query sequence.  
  

  
  
  

```
 Score =  940 bits (489),  Expect = 0.0
 Identities = 526/542 (97%), Gaps = 6/542 (1%)
 Strand=Plus/Plus

Query  38   ATATATATACACACACACTATTGATTCATCTGCTCAACCCAAGACACTATTTGTATTATA  97
            ||||||||||||||||||||||||||||||||||||||||||||||||||||||||||||
Sbjct  1    ATATATATACACACACACTATTGATTCATCTGCTCAACCCAAGACACTATTTGTATTATA  60

Query  98   TTTGTAACACTCGTACTTGACACAACATTCATTTCCACTGTATCTTCTAGTTTCTACATT  157
            ||||||||||||||||||||||||||||||||||||||||||||||||||||||||||||
Sbjct  61   TTTGTAACACTCGTACTTGACACAACATTCATTTCCACTGTATCTTCTAGTTTCTACATT  120

Query  158  TTAGTTATTTAGCAAACGCGGTTATCCAAAGAGAGAGACACACCCTGAGTGTCCCAAGAA  217
            |||||||||||||| ||||||||||||| |||||||||||||||||||||||||||||||
Sbjct  121  TTAGTTATTTAGCAGACGCGGTTATCCAGAGAGAGAGACACACCCTGAGTGTCCCAAGAA  180

Query  218  TTTACTGAACAATGGGGGTCATGACTACAAACAAAAACCGCAGTACAATCAAAACAACTG  277
            |||||||||||||| |||||||||||||||||| ||||||||||||||||| ||||||||
Sbjct  181  TTTACTGAACAATGTGGGTCATGACTACAAACAGAAACCGCAGTACAATCAGAACAACTG  240

Query  278  GTTTATTAAAAAACTGCATGTTAAAAAAAGGAGGGGAGGAGGGACAGTTCATTATGACAC  337
            |||||||| |||||||||||||| | ||||||||||||||||||||||||||||||||||
Sbjct  241  GTTTATTAGAAAACTGCATGTTAGAGAAAGGAGGGGAGGAGGGACAGTTCATTATGACAC  300

Query  338  ATTCAGAGATGAGGACAACTGCACATTTTCGTTAGACATTCTGGGTATGTCCCAAATGAC  397
            |||||||      |||||||||||||||||||||||||||||||||||||||||||||||
Sbjct  301  ATTCAGA------GACAACTGCACATTTTCGTTAGACATTCTGGGTATGTCCCAAATGAC  354

Query  398  ACCCTAATTCCCTCTATACGGTACTATTTCAAACAACACGGAACTTGGAAATCTGCAACG  457
            ||||||||||||||||||||||||||||||| ||||||||||||||||||||||||||||
Sbjct  355  ACCCTAATTCCCTCTATACGGTACTATTTCAGACAACACGGAACTTGGAAATCTGCAACG  414

Query  458  TCCGAATTCAAAAATTCGGGCATCGTCCTAGAGTTCCCTGAAGTCTCCGACCTGAAGATC  517
            |||||||||| |||||||||||||||||||||||||||||||||||||||||||||||||
Sbjct  415  TCCGAATTCAGAAATTCGGGCATCGTCCTAGAGTTCCCTGAAGTCTCCGACCTGAAGATC  474

Query  518  TCACCATCACGATTTTTAACAGATTATCATTTTTCTTATATTATAAAGTTCCCAGTTGTC  577
            ||||||||||||||||||||||||||||||||||||||||||||||||||||||||||||
Sbjct  475  TCACCATCACGATTTTTAACAGATTATCATTTTTCTTATATTATAAAGTTCCCAGTTGTC  534

Query  578  TT  579
            ||
Sbjct  535  TT  536
```

```
CPU time:     0.05 user secs.	    0.03 sys. secs	    0.08 total secs.
```
