# Supplementary material for: Discovery and application of insertion-deletion (INDEL) polymorphisms for QTL mapping of early life-history traits in Atlantic salmon
Source: BMC Genomics. 2010 Mar 8;11:156. doi: 10.1186/1471-2164-11-156 (PMC2838853; doi:10.1186/1471-2164-11-156)
Supplement: Additional file 2 — Information on developed 76 locus single-run INDEL panel in Atlantic salmon. Information on fluorescence labeling, primer concentrations, PCR pooling and links to alignments, INDEL motifs and GENESCAN (Burge and Karlin 1997) predictions of genes/exons are available in html format. [file 1471-2164-11-156-S2.ZIP › Additionalfile2/Ind2679Blast.htm]

Blast Result


|  |  |
| --- | --- |
|  | Blast 2 Sequences results |

|  |  |  |  |  |  |
| --- | --- | --- | --- | --- | --- |
| PubMed | Entrez | BLAST | OMIM | Taxonomy | Structure |

**BLAST 2 SEQUENCES RESULTS VERSION BLASTN 2.2.18 [Mar-02-2008]**


Match:
Mismatch:
gap open:
gap extension:    
x\_dropoff: 
expect:
wordsize: 
Filter 
View option 
 Standard
 Mismatch-highlighting
   
  
Masking character option 
 X for protein, n for nucleotide
 Lower case
   
Masking color option 
 Black
 Grey
 Red
   
  
Show CDS translation


---


  
 **Sequence 1**: gi|117541421|EST\_ssal\_eve\_33772 ssaleve thyroid Salmo salar cDNA Salmo salar cDNA clone ssal\_eve\_545\_328\_rev 3', mRNA sequence.  
Length = 713
(1 .. 713)
  
  
 **Sequence 2**: gi|117529865|EST\_ssal\_eve\_54647 ssaleve thyroid Salmo salar cDNA Salmo salar cDNA clone ssal\_eve\_574\_188\_rev 3', mRNA sequence.  
Length = 713
(1 .. 713)
  
  
  

|  |  |  |  |  |
| --- | --- | --- | --- | --- |
|  |  | **2** |  | **1** |

  
NOTE:Bitscore and expect value are calculated based on the size of the nr database.  
  
NOTE:If protein translation is reversed, please repeat the search with reverse strand of the query sequence.  
  

  
  
  

```
 Score = 1248 bits (649),  Expect = 0.0
 Identities = 689/699 (98%), Gaps = 6/699 (0%)
 Strand=Plus/Plus

Query  9    GTAAAAGTGTATGACGTTTATTCCAGATGTTACTGCCAGTCCCTGTTACAAAACATTATA  68
            ||||||||||||||||||||||||||||||||||||||||||||||||||||||||||||
Sbjct  1    GTAAAAGTGTATGACGTTTATTCCAGATGTTACTGCCAGTCCCTGTTACAAAACATTATA  60

Query  69   GGCTTTCCAGAATCATTGTCTTTAAAAAAAAAAAAAAGTTTACAGACAAACTGGTTAACT  128
            |||||||||||||||||||||||||||||||||||  |||||||||||||||||||||||
Sbjct  61   GGCTTTCCAGAATCATTGTCTTTAAAAAAAAAAAA--GTTTACAGACAAACTGGTTAACT  118

Query  129  GAGGCGTAAGGCACAGGCATCAACTTGTTTATTGGATACGTTCACATTGAATGAGTTAGC  188
            ||||||||||||||||||||||||||||||||||||||||||||||||||||||||||||
Sbjct  119  GAGGCGTAAGGCACAGGCATCAACTTGTTTATTGGATACGTTCACATTGAATGAGTTAGC  178

Query  189  TTAGCACTGGCCCAGTAAGTGGGTGGTTGAAAACTTTATCGAAAAGATCAACCACAGAAT  248
            ||||||||||||||||||||||||||||||||||||||||||||||||||||||||||||
Sbjct  179  TTAGCACTGGCCCAGTAAGTGGGTGGTTGAAAACTTTATCGAAAAGATCAACCACAGAAT  238

Query  249  AAGTCTAACTTTCCCTTTCCCCACTAAAAACAGTGTTTGACATAATATCCCTGAAAAAAA  308
            ||||||||||||||||||||||||||||||||||||||||||||||||||||||||||||
Sbjct  239  AAGTCTAACTTTCCCTTTCCCCACTAAAAACAGTGTTTGACATAATATCCCTGAAAAAAA  298

Query  309  AAAACACTTGTCAACATGCCATACAGACACAATTGATGTGTTTAAAGACACAGTTCTATT  368
            ||| ||||||||||||||||||||||||||||||||| ||||||||||||  ||||||||
Sbjct  299  AAA-CACTTGTCAACATGCCATACAGACACAATTGATATGTTTAAAGACA--GTTCTATT  355

Query  369  CTGATCTTTTGCTAATAATTGCTATTTTGATCAATGAGCTCAGATCTTGTACCAATAAAA  428
            ||||||||||||||||||||||||||||||||||||||||||||||||||||||||||||
Sbjct  356  CTGATCTTTTGCTAATAATTGCTATTTTGATCAATGAGCTCAGATCTTGTACCAATAAAA  415

Query  429  TTGGGCTGCCTGTGTAAACACAGCCTATGTTCCAGTCTGTATTGTAAACAGTAGATCATT  488
            ||||||||||||||||||||||||||||||||||||||||||||||||||||||||||||
Sbjct  416  TTGGGCTGCCTGTGTAAACACAGCCTATGTTCCAGTCTGTATTGTAAACAGTAGATCATT  475

Query  489  TCACATTGAAATCACAGTAGCAGGGGTTTAGAAACCATCACACCGACTCCATACCAGTTC  548
            ||||||||||||||||||||||||||||||||||||||||||||||||||||||||||||
Sbjct  476  TCACATTGAAATCACAGTAGCAGGGGTTTAGAAACCATCACACCGACTCCATACCAGTTC  535

Query  549  CCTCTATAGTACACTATTTAGACTCTGGTCCAAAGGTGTGCACTATATAGGGAATAAGGT  608
            ||||||||||||||||||||||||||||||||||||||||||||||||||||||||||| 
Sbjct  536  CCTCTATAGTACACTATTTAGACTCTGGTCCAAAGGTGTGCACTATATAGGGAATAAGGC  595

Query  609  GCCATTTGGCCAGTCCAAGGCTACTTAAGCTCTCCACAGTCTGCGATGGTGATCCTCTTG  668
            ||||||||||| ||||||||||||||||||||||||||||||||||||||||||||||||
Sbjct  596  GCCATTTGGCCGGTCCAAGGCTACTTAAGCTCTCCACAGTCTGCGATGGTGATCCTCTTG  655

Query  669  GATGTACGCCCAGAACGGGATCCGAACGAACTCCACTTT  707
            ||||||||||||||| ||||||||||| |||||||||||
Sbjct  656  GATGTACGCCCAGAAAGGGATCCGAAC-AACTCCACTTT  693
```

```
CPU time:     0.06 user secs.	    0.05 sys. secs	    0.11 total secs.
```
