# Supplementary material for: Discovery and application of insertion-deletion (INDEL) polymorphisms for QTL mapping of early life-history traits in Atlantic salmon
Source: BMC Genomics. 2010 Mar 8;11:156. doi: 10.1186/1471-2164-11-156 (PMC2838853; doi:10.1186/1471-2164-11-156)
Supplement: Additional file 2 — Information on developed 76 locus single-run INDEL panel in Atlantic salmon. Information on fluorescence labeling, primer concentrations, PCR pooling and links to alignments, INDEL motifs and GENESCAN (Burge and Karlin 1997) predictions of genes/exons are available in html format. [file 1471-2164-11-156-S2.ZIP › Additionalfile2/Ind2880Blast.htm]

Blast Result


|  |  |
| --- | --- |
|  | Blast 2 Sequences results |

|  |  |  |  |  |  |
| --- | --- | --- | --- | --- | --- |
| PubMed | Entrez | BLAST | OMIM | Taxonomy | Structure |

**BLAST 2 SEQUENCES RESULTS VERSION BLASTN 2.2.18 [Mar-02-2008]**


Match:
Mismatch:
gap open:
gap extension:    
x\_dropoff: 
expect:
wordsize: 
Filter 
View option 
 Standard
 Mismatch-highlighting
   
  
Masking character option 
 X for protein, n for nucleotide
 Lower case
   
Masking color option 
 Black
 Grey
 Red
   
  
Show CDS translation


---


  
 **Sequence 1**: gi|117453886|EST\_ssal\_evd\_6813 ssalevd thymus Salmo salar cDNA Salmo salar cDNA clone ssal\_evd\_507\_312\_rev 3', mRNA sequence.  
Length = 755
(1 .. 755)
  
  
 **Sequence 2**: gi|117834848|EST\_ssal\_evf\_9946 ssalevf mixed\_tissue Salmo salar cDNA Salmo salar cDNA clone ssal\_evf\_511\_254\_rev 3', mRNA sequence.  
Length = 761
(1 .. 761)
  
  
  

|  |  |  |  |  |
| --- | --- | --- | --- | --- |
|  |  | **2** |  | **1** |

  
NOTE:Bitscore and expect value are calculated based on the size of the nr database.  
  
NOTE:If protein translation is reversed, please repeat the search with reverse strand of the query sequence.  
  

  
  
  

```
 Score = 1377 bits (716),  Expect = 0.0
 Identities = 742/750 (98%), Gaps = 6/750 (0%)
 Strand=Plus/Plus

Query  6    AAAATCAGCGTTTATTGGAATATAATGTGGTCAGAAAATATCACAACCAAATGAAAAAGG  65
            ||||||||||||||||||||||||||||||||||||||||||||||||||||||||||||
Sbjct  4    AAAATCAGCGTTTATTGGAATATAATGTGGTCAGAAAATATCACAACCAAATGAAAAAGG  63

Query  66   GGAACATAATATACACTCATTCAGAATATCAGTGGCCTTCCGTCTCTCATCCTCAAACAA  125
            ||||||||||||||||||||||||||||||||||||||||||||||||||||||||||||
Sbjct  64   GGAACATAATATACACTCATTCAGAATATCAGTGGCCTTCCGTCTCTCATCCTCAAACAA  123

Query  126  TATTACACTGCTGACATCCAATCTACCACACACACACACTGACGACACATACATAGACTG  185
            |||||||||||||||||||||||||||||||||||||  |||||||||||||||||||||
Sbjct  124  TATTACACTGCTGACATCCAATCTACCACACACACAC--TGACGACACATACATAGACTG  181

Query  186  TCTTTCCCCTCCCTGTCACACGAGCATTATCTCTTGTATCTAAATACATACTCTCCTAAA  245
            ||||||||||||||||||||| ||||||||||||||||||||||||||||||||||||||
Sbjct  182  TCTTTCCCCTCCCTGTCACACAAGCATTATCTCTTGTATCTAAATACATACTCTCCTAAA  241

Query  246  CATTTCCTGATTCTTCTAAACACACTCGTACCAGCTTCTCTCTCAAAAAAACACACACAC  305
            |||||||||||||||||||||||||||||||||||||||||||||||||||||||    |
Sbjct  242  CATTTCCTGATTCTTCTAAACACACTCGTACCAGCTTCTCTCTCAAAAAAACACA----C  297

Query  306  TTGTTGTCTCTGGTCTATCGGAGGCTGTTGGGCTGAGAGCCAGCCAGGCCAGGGTGGTCC  365
            ||||||||||||||||||||||||||||||||||||||||||||||||||||||||||||
Sbjct  298  TTGTTGTCTCTGGTCTATCGGAGGCTGTTGGGCTGAGAGCCAGCCAGGCCAGGGTGGTCC  357

Query  366  GGACTGTGGCGGTTCTTCTTCTTTTTCTTGTCTTTCTTTTTCTTCTTGCGGTCAGGGTCA  425
            ||||||||||||||||||||||||||||||||||||||||||||||||||||||||||||
Sbjct  358  GGACTGTGGCGGTTCTTCTTCTTTTTCTTGTCTTTCTTTTTCTTCTTGCGGTCAGGGTCA  417

Query  426  TCATCCCTTTTCTTCTTCTTCTTCTTGGGGTCAGAGTCTGAGGGCGTCTCTTGCGGTATG  485
            ||||||||||||||||||||||||||||||||||||||||||||||||||||||||||||
Sbjct  418  TCATCCCTTTTCTTCTTCTTCTTCTTGGGGTCAGAGTCTGAGGGCGTCTCTTGCGGTATG  477

Query  486  GGATCCTGTGGTCGGTTGTGTTTGTGCTTATGCTTGCTCTTCTTCTTAGGAGGCTGGATG  545
            ||||||||||||||||||||||||||||||||||||||||||||||||||||||||||||
Sbjct  478  GGATCCTGTGGTCGGTTGTGTTTGTGCTTATGCTTGCTCTTCTTCTTAGGAGGCTGGATG  537

Query  546  TGCATCAGTCGGTACTGCTCCGGTAGCGGTCCGGTGTGCAGTCTGAAGCCGGTGAGGCTG  605
            ||||||||||||||||||||||||||||||||||||||||||||||||||||||||||||
Sbjct  538  TGCATCAGTCGGTACTGCTCCGGTAGCGGTCCGGTGTGCAGTCTGAAGCCGGTGAGGCTG  597

Query  606  GCTCCGGTCAGAGGGCTGAAGGAGTTGCCACACACTGGAGGCTTCTCTATCAGAGAGTGC  665
            ||||||||||||||||||||||||||||||||||||||||||||||||||||||||| ||
Sbjct  598  GCTCCGGTCAGAGGGCTGAAGGAGTTGCCACACACTGGAGGCTTCTCTATCAGAGAGCGC  657

Query  666  AACGAACTGCCATCCTGGGTACCAGGCCAGTCTATCATACCTGGTAACTCTGGCAGGAAG  725
            ||||||||||||||||||||||||||||||||||||||||||||||||||||||||||||
Sbjct  658  AACGAACTGCCATCCTGGGTACCAGGCCAGTCTATCATACCTGGTAACTCTGGCAGGAAG  717

Query  726  TTGCTGAGCTTCTCCTTGACTTTCTTGCCA  755
            ||||||||||||||||||||||||||||||
Sbjct  718  TTGCTGAGCTTCTCCTTGACTTTCTTGCCA  747
```

```
CPU time:     0.05 user secs.	    0.03 sys. secs	    0.08 total secs.
```
