# Supplementary material for: Discovery and application of insertion-deletion (INDEL) polymorphisms for QTL mapping of early life-history traits in Atlantic salmon
Source: BMC Genomics. 2010 Mar 8;11:156. doi: 10.1186/1471-2164-11-156 (PMC2838853; doi:10.1186/1471-2164-11-156)
Supplement: Additional file 2 — Information on developed 76 locus single-run INDEL panel in Atlantic salmon. Information on fluorescence labeling, primer concentrations, PCR pooling and links to alignments, INDEL motifs and GENESCAN (Burge and Karlin 1997) predictions of genes/exons are available in html format. [file 1471-2164-11-156-S2.ZIP › Additionalfile2/Ind457Blast.htm]

Blast Result


|  |  |
| --- | --- |
|  | Blast 2 Sequences results |

|  |  |  |  |  |  |
| --- | --- | --- | --- | --- | --- |
| PubMed | Entrez | BLAST | OMIM | Taxonomy | Structure |

**BLAST 2 SEQUENCES RESULTS VERSION BLASTN 2.2.18 [Mar-02-2008]**


Match:
Mismatch:
gap open:
gap extension:    
x\_dropoff: 
expect:
wordsize: 
Filter 
View option 
 Standard
 Mismatch-highlighting
   
  
Masking character option 
 X for protein, n for nucleotide
 Lower case
   
Masking color option 
 Black
 Grey
 Red
   
  
Show CDS translation


---


  
 **Sequence 1**: gi|117445129|EST\_ssal\_evd\_3999 ssalevd thymus Salmo salar cDNA Salmo salar cDNA clone ssal\_evd\_503\_380\_rev 3', mRNA sequence.  
Length = 406
(1 .. 406)
  
  
 **Sequence 2**: gi|117480229|EST\_ssal\_evd\_32521 ssalevd thymus Salmo salar cDNA Salmo salar cDNA clone ssal\_evd\_543\_104\_rev 3', mRNA sequence.  
Length = 668
(1 .. 668)
  
  
  

|  |  |  |  |  |
| --- | --- | --- | --- | --- |
|  |  | **2** |  | **1** |

  
NOTE:Bitscore and expect value are calculated based on the size of the nr database.  
  
NOTE:If protein translation is reversed, please repeat the search with reverse strand of the query sequence.  
  

  
  
  

```
 Score =  737 bits (383),  Expect = 0.0
 Identities = 396/400 (99%), Gaps = 3/400 (0%)
 Strand=Plus/Plus

Query  3    ACAAGTGAAGTTTAATTATTTTAGTTGTACTGCATTTCTGCCCAATATAAAAATTGAAAT  62
            ||||||||||||||||||||||||||||||||||||||||||||||||||||||||||||
Sbjct  3    ACAAGTGAAGTTTAATTATTTTAGTTGTACTGCATTTCTGCCCAATATAAAAATTGAAAT  62

Query  63   ATCCAAGGACAATACATAGAAATTAAGTGCTGTTACAGTAGTTTGACAAATTTTTAAAAG  122
            |||||||||||||||||||||||||||||||||||||||||||||||||||||| |||||
Sbjct  63   ATCCAAGGACAATACATAGAAATTAAGTGCTGTTACAGTAGTTTGACAAATTTTAAAAAG  122

Query  123  TAGGTTGCCATTCAAACAATGTGGACCAAACAGTAGGGGGCATCTGAGCAGGACTTTGAG  182
            ||||||||||||||||||||||||||||||||||||||||||||||||||||||||||||
Sbjct  123  TAGGTTGCCATTCAAACAATGTGGACCAAACAGTAGGGGGCATCTGAGCAGGACTTTGAG  182

Query  183  TTGTAAAAATGCAAGGCACACCATGCATCATGAGCAATACAATGAATAAACTATTTTTTG  242
            ||||||||||||||||||||||||||||||||||||||||||||||||||||||||||||
Sbjct  183  TTGTAAAAATGCAAGGCACACCATGCATCATGAGCAATACAATGAATAAACTATTTTTTG  242

Query  243  AAATAAAGGTGCTTAGACAAGCCCCGACATTAAAGTGTCCTTTAAATGTTAAGGAGGGAC  302
            ||||||||||||||||||||||||||||||||||||||||||||||||||   |||||||
Sbjct  243  AAATAAAGGTGCTTAGACAAGCCCCGACATTAAAGTGTCCTTTAAATGTT---GAGGGAC  299

Query  303  AGAAGAACCAGCATTCCAAATACTCCTAGTCAAACCTTTCGAGACAGACATCCAGTTCCA  362
            ||||||||||||||||||||||||||||||||||||||||||||||||||||||||||||
Sbjct  300  AGAAGAACCAGCATTCCAAATACTCCTAGTCAAACCTTTCGAGACAGACATCCAGTTCCA  359

Query  363  CTGACATACATGTGAATCCACAAATTAATGTTCGGATTTG  402
            ||||||||||||||||||||||||||||||||||||||||
Sbjct  360  CTGACATACATGTGAATCCACAAATTAATGTTCGGATTTG  399
```

```
CPU time:     0.05 user secs.	    0.03 sys. secs	    0.08 total secs.
```
