# Supplementary material for: Discovery and application of insertion-deletion (INDEL) polymorphisms for QTL mapping of early life-history traits in Atlantic salmon
Source: BMC Genomics. 2010 Mar 8;11:156. doi: 10.1186/1471-2164-11-156 (PMC2838853; doi:10.1186/1471-2164-11-156)
Supplement: Additional file 2 — Information on developed 76 locus single-run INDEL panel in Atlantic salmon. Information on fluorescence labeling, primer concentrations, PCR pooling and links to alignments, INDEL motifs and GENESCAN (Burge and Karlin 1997) predictions of genes/exons are available in html format. [file 1471-2164-11-156-S2.ZIP › Additionalfile2/index.html]

| Appendix 2. Information on developed 76 locus single-run INDEL panel including fluorescence labeling, primer concentrations, PCR pooling, alignments of homologous sequences, INDEL motifs and GENESCAN (Burge and Karlin 1997) predictions of genes/exons. | | | | | | | | | | | | | | | | | | | |  |
|  | | | | | | | | | | | | | | | | | | | | |
| No. | Marker name | Alternate | GenBank | Observed sizes of Fragments | | | Polym | Indel | PCR | Fluor. | Primer conc. (pmol/μL) | | PCR pooling | Alignment | GENSCANW | GENSCANW | LG | M13 tail + Forward primer 5'-3' | Reverse primer 5'-3' | Comment |
|  |  | name | Acc. No. | Bur | Can | Narva | code | size |  | label | Forw. | Rev. | μl |  | prediction | output |  | M13 -CACGACGTTGTAAAACGAC |  |  |
| 1 | SsaIND100TKU | 8604L | EG831853 | 89 | 89 | 89/97 | P | 6 | 1a | FAM | 0.05 | 0.3 | 1 | results8604.html | EG831853.htm | EG831853.pdf | AS-10 | CACGACGTTGTAAAACGACGGCTGGATTCAGTGCTTTTC | GTTTCAAGGTCTGACCAGCAGATG | microsatellite |
| 2 | SsaIND106TKU | 9552C | EG757129 | 216218 | 217/219 | 216218 | P | 2 | 1a | FAM | 0.05 | 0.2 | 1 | results9552.html | - |  | AS-13 | CACGACGTTGTAAAACGACGCATTTTGAGGTGGGAAAGA | GTTTGGCTGTTGTATGTGATGGTG |  |
| 3 | SsaIND146TKU | 17363D | EG758656 | 100/102 | 100/102 | 100/102 | P | 2 | 1a | FAM | 0.05 | 0.3 | 1 | results17363.html | - |  | - | CACGACGTTGTAAAACGACCCAAAAGAGTATGCCCCTCT | GTTTAGCCTGGTTGCCGTCTAT |  |
| 4 | SsaIND165TKU | 21745D | EG817596 | - | 263 | 260/263 | P | 3 | 1a | FAM | 0.05 | 0.2 | 1 | results21745.html | - |  | - | CACGACGTTGTAAAACGACTGAAGCAGAGCCAGACAGAA | GTTTCATGCCACTTGGACAACAT |  |
| 5 | SsaIND178TKU | 20411A | DW580704 | 156/159 | 156/159 | 156/159 | P | 3 | 1a | FAM | 0.033 | 0.125 | 1 | results20411.html | - |  | - | CACGACGTTGTAAAACGACCAGGGGCACAAACAAAACA | GTTTAGTGGCACACAACGAACATC |  |
| 6 | SsaIND192TKU | Ind2880 | EG786105 | - | - | 127/129 | P | 2 | 1a | FAM | 0.033 | 0.125 | 1 | Ind2880Blast.htm | EG786105.htm | EG786105.pdf | AS-10 | CACGACGTTGTAAAACGACACTGTCTTTCCCCTCCCTGT | GTTTTTGAGAGAGAAGCTGGTACG |  |
| 7 | SsaIND193TKU | Ind139 | EG860477 | - | - | 137/143 | P | 6 | 1a | FAM | 0.033 | 0.125 | 1 | Ind139Blast.htm | EG860477.htm | EG860477.pdf | AS-9 | CACGACGTTGTAAAACGACCATCCATAAAGTCCCCAAGC | GTTTCAGAGCACTCCAACCACAGA |  |
| 8 | SsaIND194TKU | Ind457C | EG777352 | - | - | 149/152 | P | 3 | 1a | FAM | 0.033 | 0.125 | 1 | Ind457Blast.htm | - |  | AS-10 | CACGACGTTGTAAAACGACTAAAAATGCAAGGCACACCA | GTTTGGAATGCTGGTTCTTCTGTC |  |
| 9 | SsaIND195TKU | Ind1836 | EG854090 | - | - | 168/177 | P | 9 | 1a | FAM | 0.033 | 0.125 | 1 | Ind1836Blast.htm | EG854090.htm | EG854090.pdf | AS-9 | CACGACGTTGTAAAACGACCAGCAGCAGTGTGTCCTCTC | GTTTCTGAGGGCCGAATCCTAAAT |  |
| 10 | SsaIND196TKU | Ind2679 | EG872866 | - | - | 204/208 | P | 2.2 | 1a | FAM | 0.05 | 0.2 | 1 | Ind2679Blast.htm | - |  | AS-13 | CACGACGTTGTAAAACGACGTTTACACAGGCAGCCCAAT | GTTTCCCTTTCCCCACTAAAAACA |  |
| 11 | SsaIND197TKU | Ind2130 | EG887233 | - | - | 269/270 | P | 7 | 1a | FAM | 0.05 | 0.2 | 1 | Ind2130Blast.htm | - |  | AS-5 | CACGACGTTGTAAAACGACCATGCCTGAGCCAGCTAAAT | GTTTCTCCTCCTCCTCTCCAACA |  |
| 12 | SsaIND092TKU | 7157K | EG836414 | 111/113 | 113 | 111/113 | P | 2 | 1a | FAM | 0.033 | 0.125 | 1 | results7157.html | EG836414.htm | EG836414.pdf | X10 | CACGACGTTGTAAAACGACTGAGATTGGATAAACCCACAAA | GTTTGGAAAGGACAACATCACAGGA |  |
| 13 | SsaIND096TKU | 8229A | CB516218 | 562 | 562 | 562/567 | P | 5 | 1b | FAM | 0.05 | 0.3 | 2.5 | results8229.html | - |  | X15 | CACGACGTTGTAAAACGACGACAGCAGCGACAAAAACAA | GTTTCACCCCATTGAAGTTGAGGT |  |
| 14 | SsaIND117TKU | 11953L | EG897476 | 427/430 | 427/430 | 428/430 | P | 3 | 1b | FAM | 0.05 | 0.3 | 2.5 | results11953.html | - |  | X5 | CACGACGTTGTAAAACGACTTACCTCCCTCAAACGCAAC | GTTTAGGCGGTCTTTAGGGTTTTC |  |
| 15 | SsaIND129TKU | 13526G | EG932893 | 454/456 | 454/456 | 454/456 | P | 2 | 1b | FAM | 0.05 | 0.3 | 2.5 | results13526.html | - |  | - | CACGACGTTGTAAAACGACCTGGCATGACACAGTCCAAA | GTTTGCATTGTGGATTACTGCTTCG |  |
| 16 | SsaIND136TKU | 15402E | DY714614 | - | 572 | 570/572 | P | 2 | 1b | FAM | 0.05 | 0.3 | 2.5 | results15402.html | - |  | - | CACGACGTTGTAAAACGACTTGGATATTCTGGGCTTTGG | GTTTCATTTTGCACAGGAGAACGA |  |
| 17 | SsaIND174TKU | 20056D | DW547818 | 308/310 | 308/310 | 308/310 | P | 3 | 1b | FAM | 0.05 | 0.2 | 2.5 | results20056.html | DW547818.htm | DW547818.pdf | - | CACGACGTTGTAAAACGACCGCTACAACACACCCATCAA | GTTTGCTACCACTGAAGAAACTGC | 3 bp  INDEL in exon |
| 18 | SsaIND198TKU | Ind1921 | CB508135 | - | - | 312/316 | P | 4 | 1b | FAM | 0.05 | 0.2 | 2.5 | Ind1921Blast.htm | - |  | X9 | CACGACGTTGTAAAACGACGCACGCCATAAAGAATGGAT | GTTTGCAGTAGACCCATCACGTC |  |
| 19 | SsaIND199TKU | Ind2070 | CK898265 | - | - | 324/327 | P | 4 | 1b | FAM | 0.05 | 0.2 | 2.5 | Ind2070Blast.htm | - |  | AS-4a | CACGACGTTGTAAAACGACTGACTGACCAACTCCACACA | GTTTCGAAACGACCTCAAAGCACT |  |
| 20 | SsaIND200TKU | Ind2231 | EG759430 | - | - | 329/331 | P | 2 | 1b | FAM | 0.05 | 0.2 | 2.5 | Ind2231Blast.htm | - |  | X13 | CACGACGTTGTAAAACGACCCAGCAAACTCGAACATGAA | GTTTAAGAAGCCCTTACCCCAAAA |  |
| 21 | SsaIND201TKU | Ind2377 | EG792034 | - | - | 415/419 | P | 6 | 1b | FAM | 0.05 | 0.2 | 2.5 | Ind2377Blast.htm | - |  | AS-5 | CACGACGTTGTAAAACGACCATCTGCTCAACCCAAGACA | GTTTATTCGGACGTTGCAGATTTC |  |
| 22 | SsaIND202TKU | Ind2330 | EG873536 | - | - | 530/537 | P | 7 | 1b | FAM | 0.05 | 0.2 | 2.5 | Ind2330Blast.htm | - |  | - | CACGACGTTGTAAAACGACAACTGCCTCTGGGTGAAATG | GTTTGCTCATGGGAAGGGATATAGG |  |
| 23 | SsaIND007TKU | 3960I | EG822398 | 275/277 | - | 275/277 | P | 2 | 2a | VIC | 0.075 | 0.3 | 1 | results3960.html | EG822398.htm | EG822398.pdf | X3 | CACGACGTTGTAAAACGACTCCATTTTGCGTTACCACAG | GTTTGCTGTCCAAGAACATTTCACA |  |
| 24 | SsaIND008TKU | 4151e | EG869785 | 198/200 | 198/200 | 198/200 | P | 2 | 2a | VIC | 0.075 | 0.3 | 1 | results4151.html | EG869785.htm | EG869785.pdf | AS-5 | CACGACGTTGTAAAACGACTGACATCCAAATTCAACCAGA | GTTTCACCTGGGAACCAGACAGTT |  |
| 25 | SsaIND018TKU | 9520D | EG791246 | 127/129 | 127 | 126/129 | P | 2 | 2a | VIC | 0.033 | 0.125 | 1 | results9520.html | EG791246.htm | EG791246.pdf | AS-8 | CACGACGTTGTAAAACGACCATCTGCCCAAACGTATCAA | GTTTCAGAATCGGCTTGGAAGAAG |  |
| 26 | SsaIND022TKU | 32c | DW584142 | 177 | 177 | 175/177 | P | 2 | 2a | VIC | 0.033 | 0.125 | 1 | results32.html | DW584142.htm | DW584142.pdf | AS-9 | CACGACGTTGTAAAACGACATGGTTAGCAGTGAGCAGCA | GTTTAATGCCCTGATTCTGTGGAC |  |
| 27 | SsaIND032TKU | 1094G2 | EG930476 | 100/106 | 100/106 | 100/106 | P | 3.6 | 2a | VIC | 0.05 | 0.2 | 1 | results1094.html | EG930476.htm | EG930476.pdf | AS-32 | CACGACGTTGTAAAACGACGGAGGAGGCAACAAGGGTAT | GTTTAGAAGTGGTTGGGTTGGTGA | 3bp repeat in exon |
| 28 | SsaIND038TKU | 1338L | EG859393 | 218/224 | 218/224 | 218/224 | P | 6 | 2a | VIC | 0.033 | 0.125 | 1 | results1338.html | - |  | AS-8 | CACGACGTTGTAAAACGACAGGGGGTGGGAAAAACATAC | GTTTGTGGGGGAATTTAATGAGG |  |
| 29 | SsaIND054TKU | 2456V | EG792234 | 153/156 | 156 | 153/156 | P | 3 | 2a | VIC | 0.05 | 0.2 | 1 | results2456.html | EG792234.htm | EG792234.pdf | AS-23 | CACGACGTTGTAAAACGACAGTGGCTAGCTGCTTTGGAA | GTTTCTGAGAGCAGATCCTTCCTT |  |
| 30 | SsaIND065TKU | 2889J | EG931347 | 232/234 | 234 | 232/234 | P | 2 | 2a | VIC | 0.05 | 0.2 | 1 | results2889.html | - |  | AS-21 | CACGACGTTGTAAAACGACCCTGTGCACCCATAAATGAA | GTTTAGAGGACGGAACAGGTTGTG |  |
| 31 | SsaIND095TKU | 7583I | EG905690 | 254 | 250/252 | 254 | P | 2 | 2a | VIC | 0.075 | 0.3 | 1 | results7583.html | - |  | - | CACGACGTTGTAAAACGACGTCCCATTATCCAACCAACC | GTTTGCCTTCAGTCATCAAACCA |  |
| 32 | SsaIND110TKU | 10585C | EG761490 | 139 | 141 | 139 | TA P | 2 | 2a | VIC | 0.05 | 0.2 | 1 | results10585.html | - |  | - | CACGACGTTGTAAAACGACTGCGAGGTTCAGAGTTGTCTT | GTTTAATAATGCATGGGGCTGAAG |  |
| 33 | SsaIND004TKU | 3355K | EG903132 | 427/429 | 429 | 427/429 | P | 2 | 2b | VIC | 0.05 | 0.2 | 2.5 | results3355.html | - |  | - | CACGACGTTGTAAAACGACGCCAAACAGCCATACGAGAT | GTTTCTTCCTTCCCCTCACTCTCC |  |
| 34 | SsaIND020TKU | 10259H | EG840630 | 311/316 | 311/316 | 311/316 | P | 5 | 2b | VIC | 0.05 | 0.2 | 2.5 | results10259.html | - |  | - | CACGACGTTGTAAAACGACGGTGCCATTCGTTCAGATTT | GTTTGAAGAGGGGTTGGGAATGTT | microsatellite |
| 35 | SsaIND021TKU | 11005M | EG910563 | 373375 | 373 | 373375 | P | 2 | 2b | VIC | 0.05 | 0.3 | 2.5 | results11005.html | - |  | AS-4a | CACGACGTTGTAAAACGACTGTGCTCTCCTCCTTAACAATG | GTTTATCAAGCCACACGTCAACAA |  |
| 36 | SsaIND049TKU | 2136E | CA046731 | 350/354 | 352 | 350/354 | P | 2.4 | 2b | VIC | 0.05 | 0.2 | 2.5 | results2136.html | CA046731.htm | CA046731.pdf | AS-25 | CACGACGTTGTAAAACGACGATCCTACTGGCCCTGTGTG | GTTTAGTTATCCAGGCCACAATGC |  |
| 37 | SsaIND058TKU | 2571c | DW471900 | 331 | 333 | 331/333 | P | 2.7 | 2b | VIC | 0.05 | 0.3 | 2.5 | results2571.html | - |  | AS-14 | CACGACGTTGTAAAACGACTGAGCAGAGGATTGAGAAGGA | GTTTGTAGTGGGTGTGTCCAACA |  |
| 38 | SsaIND076TKU | 4246L | EG782907 | 420/422 | 420/422 | 420/422 | P | 2.2 | 2b | VIC | 0.075 | 0.3 | 2.5 | results4246.html | EG782907.htm | EG782907.pdf | AS-32 | CACGACGTTGTAAAACGACGCAGTGGTAAGATGGGCACT | GTTTAAATGATGACCGACGATTGG |  |
| 39 | SsaIND113TKU | 10789B | EG766107 | 412 | 410/412 | 412 | P | 2 | 2b | VIC | 0.075 | 0.3 | 2.5 | results10789.html | - |  | - | CACGACGTTGTAAAACGACCATGCCTCTTCTCCCTACCA | GTTTCCCACCATAAAAACAATCCA |  |
| 40 | SsaIND150TKU | 18185F | CA044968 | 504 | 498 | 504 | TA P | 3 | 2b | VIC | 0.075 | 0.3 | 2.5 | results18185.html | - |  | - | CACGACGTTGTAAAACGACTTTTGCCATGTTTGCCATTA | GTTTGCCAGTGAAAGCCTGTATGG |  |
| 41 | SsaIND151TKU | 18256D | EG822439 | 402 | 400/402 | 402 | P | 2 | 2b | VIC | 0.075 | 0.3 | 2.5 | results18256.html | - |  | - | CACGACGTTGTAAAACGACATAAAAGACTGGCCGAAGCA | GTTTAGCTGGACAGAGACGTGTGA |  |
| 42 | SsaIND017TKU | 8396P | EG787097 | 116/120 | 116/120 | 116/120 | P | 4 | 3a | NED | 0.033 | 0.125 | 1 | results8396.html | - |  | X8 | CACGACGTTGTAAAACGACATGCTTGCCTCCATACCAAC | GTTTACACAGAAACTGCAGCAAGG |  |
| 43 | SsaIND023TKU | 86g | EG839692 | 203/206 | 206 | 206 | P | 3 | 3a | NED | 0.05 | 0.2 | 1 | results86.html | EG839692.htm | EG839692.pdf | - | CACGACGTTGTAAAACGACTCCTAAAACACGGAGCCAAG | GTTTGGAATGACCCAGGTATGGT |  |
| 44 | SsaIND024TKU | 190S | CB500290 | 100/104 | 100/104 | 104105 | P | 3 | 3a | NED | 0.05 | 0.2 | 1 | results190.html | - |  | X8 | CACGACGTTGTAAAACGACTGCACAACAGAGACGAAACC | GTTTGCCATTCAACTCACCCCTAA |  |
| 45 | SsaIND035TKU | 1271X | EG872016 | 166 | 169 | 166/169 | P | 3 | 3a | NED | 0.033 | 0.125 | 1 | results1271.html | EG872016.htm | EG872016.pdf | X9 | CACGACGTTGTAAAACGACTATTGCCCGGAGTCAAGTTC | GTTTCAAGCAACATGCAGAAAGC | 3bp repeat in exon |
| 46 | SsaIND037TKU | 1329g | EG867098 | 226/228 | 226/228 | 228 | P | 2 | 3a | NED | 0.05 | 0.2 | 1 | results1329.html | EG867098.htm | EG867098.pdf | - | CACGACGTTGTAAAACGACTGGAGAGGTCACAAGCACTG | GTTTACACGGTTTCCTTCTTCCTG |  |
| 47 | SsaIND051TKU | 2273K | DW540946 | 194/197 | 194/197 | 194/197 | P | 3 | 3a | NED | 0.075 | 0.3 | 1 | results2273.html | DW540946.html | DW540946.pdf | AS-15 | CACGACGTTGTAAAACGACCCTGCCTCTCAGGACTGTTC | GTTTAGCGACAAATTACCCATCCA |  |
| 48 | SsaIND079TKU | 4868M | EG770025 | 176 | 176 | 172/176 | P | 4.4 | 3a | NED | 0.033 | 0.125 | 1 | results4868.html | - |  | X9 | CACGACGTTGTAAAACGACCAGGGGTCTGTTCAGGATGT | GTTTACTCCTCAAACTGGGGTGTG |  |
| 49 | SsaIND179TKU | 20475C | EG765334 | 186/189 | 186/189 | 186 | P | 3 | 3a | NED | 0.075 | 0.3 | 1 | results20475.html | - |  | - | CACGACGTTGTAAAACGACACAGCATGCCACCAACACTA | GTTTCTTTTCAGCACAAGGCATGTA |  |
| 50 | SsaIND185TKU | 21188C | EG647956 | 149 | 149 | 147/149 | P | 2 | 3a | NED | 0.033 | 0.125 | 1 | results21188.html | EG647956.htm | EG647956.pdf | AS-9 | CACGACGTTGTAAAACGACCCCGATGTGGTAAACAAAGC | GTTTGACCGAACTGGTCCTACCTG |  |
| 51 | SsaIND067TKU | 3270v | CB500451 | 372 | - | 381 | P | 3 | 3b | NED | 0.05 | 0.2 | 2.5 | results3270.html | CB500451.htm | CB500451.pdf | - | CACGACGTTGTAAAACGACGCATGGTTCCAAAGCAAAAA | GTTTCCCGTTCAGAACAAAGCAAA | 3 bp INDEL in exon, observed INDEL 9bp long |
| 52 | SsaIND098TKU | 8417F | EG782320 | 468 | 466/468 | 468 | P | 2 | 3b | NED | 0.075 | 0.3 | 2.5 | results8417.html | - |  | - | CACGACGTTGTAAAACGACTTTAAAGGCCCAGTGCAATC | GTTTGGGCAGAATGAGCCATCTAA |  |
| 53 | SsaIND125TKU | 13049E | DY692052 | 561/563 | 561/563 | 561/563 | P | 4 | 3b | NED | 0.075 | 0.3 | 2.5 | results13049.html | - |  | - | CACGACGTTGTAAAACGACTGACTAAGGTGGTGGGGAAG | GTTTCACTTCAATAATGCAAAACCACA | microsatellite |
| 54 | SsaIND130TKU | 13587E | DY692726 | 412/414 | 414 | 412/414 | P | 2 | 3b | NED | 0.075 | 0.3 | 2.5 | results13587.html | DY692726.htm | DY692726.pdf | - | CACGACGTTGTAAAACGACGGAGGAATAAGGGAGGAGCA | GTTTCGGGAAATAGTGCTTTTG |  |
| 55 | SsaIND142TKU | 16424E | CA063272 | 430/432 | 430/432 | 430/432 | P | 2 | 3b | NED | 0.075 | 0.3 | 2.5 | results16424.html | - |  | AS-11 | CACGACGTTGTAAAACGACAAGGCAGCAAAACTGGTAGC | GTTTGGGGACACTGGAGTGAAAAT |  |
| 56 | SsaIND147TKU | 17571D | DY708327 | 256 | 256 | 248/256 | TA P | 8 | 3b | NED | 0.05 | 0.2 | 2.5 | results17571.html | - |  | - | CACGACGTTGTAAAACGACACCGAATAAAGCCGCACTTA | GTTTCCTAATCGCACACCCAAT |  |
| 57 | SsaIND160TKU | 20196E | EG942430 | 294/296 | 294/296 | 296 | P | 2 | 3b | NED | 0.05 | 0.2 | 2.5 | results20196.html | - |  | - | CACGACGTTGTAAAACGACAACTCCCCAATCCTTTGACA | GTTTGGCATCAACATTTGAAAACCA |  |
| 58 | SsaIND183TKU | 20742A | CA064223 | 497/508 | 504/508 | 508 | P | 4 | 3b | NED | 0.075 | 0.3 | 2.5 | results20742.html | - |  | - | CACGACGTTGTAAAACGACGCCCAAAATGTACAGGCAAT | GTTTGATTCTCATGTTAGCCGTCCA |  |
| 59 | SsaIND009TKU | 4237O | DW473270 | 132/138 | 136/141 | 138/141 | P | 4 | 4a | PET | 0.05 | 0.2 | 1 | results4237.html | DW473270.htm | DW473270 .pdf | - | CACGACGTTGTAAAACGACCCTGGGGACAGTACAACCAG | GTTTAATTGGGCTGGGTAGAAAGG |  |
| 60 | SsaIND036TKU | 1309C | EG808658 | 200/210 | 200/210 | 200/210 | P | 10 | 4a | PET | 0.033 | 0.125 | 1 | results1309.html | EG808658.htm | EG808658.pdf | X5 | CACGACGTTGTAAAACGACCAAAGGGCTGTTAAAGGAATG | GTTTCTGTATGGTGCATTTAAGATCATTG | 10bp  INDEL immediately after exon |
| 61 | SsaIND080TKU | 4955H | EG778546 | 231 | - | 231/233 | P | 2 | 4a | PET | 0.033 | 0.125 | 1 | results4955.html | EG778546.htm | EG778546.pdf | AS-32 | CACGACGTTGTAAAACGACAACCTTCCCCTGATCTTGCT | GTTTCACTACAACCGCTCCATCCT |  |
| 62 | SsaIND099TKU | 8570Q | EG889615 | 100/104 | 100/104 | 100/104 | P | 3 | 4a | PET | 0.033 | 0.125 | 1 | results8570.html | EG889615.htm | EG889615.pdf | AS-10 | CACGACGTTGTAAAACGACAGCAGGTCGTGTTTTGAAGC | GTTTCTGATCGTCCTCGCCTTTAC | 3 bp  INDEL in exon |
| 63 | SsaIND118TKU | 11971N | EG796527 | 107/109 | 109 | 109 | P | 2 | 4a | PET | 0.033 | 0.125 | 1 | results11971.html | EG796527.htm | EG796527.pdf | AS-1 | CACGACGTTGTAAAACGACTATGCGCTACCCCACTAACG | GTTTAGATACGAGGCAGGGGTGT |  |
| 64 | SsaIND122TKU | 12783G | EG830992 | 244 | 244/246 | 244 | P | 2 | 4a | PET | 0.075 | 0.3 | 1 | results12783.html | EG830992.htm | EG830992.pdf | - | CACGACGTTGTAAAACGACTGTGGGATTCATTTCCTGTG | GTTTGGACTGAGGGCATGCTTTTT |  |
| 65 | SsaIND123TKU | 13028H | EG823293 | 160/167 | 167 | 160/164/167 | P | 2 | 4a | PET | 0.05 | 0.2 | 1 | results13028.html | EG823293.htm | EG823293.pdf | - | CACGACGTTGTAAAACGACGGGACAACAAGCTCTGCTAAA | GTTTCGTGGACCAGGCTAGGATTA |  |
| 66 | SsaIND140TKU | 15900H | EG841183 | 221 | 221 | 217/221 | P | 5 | 4a | PET | 0.033 | 0.125 | 1 | results15900.html | - |  | AS-7 | CACGACGTTGTAAAACGACGCTTTCGTTTATTTTGAACCA | GTTTACACACTGGGCTTCGTTTTC |  |
| 67 | SsaIND145TKU | 17300F | EG911321 | 149/153 | 149 | 149/153 | P | 4 | 4a | PET | 0.05 | 0.2 | 1 | results17300.html | - |  | AS-9 | CACGACGTTGTAAAACGACTCCAATTTCATGTTGCTAATGTG | GTTTAACCTTCCCCTAACCCTGTAA |  |
| 68 | SsaIND171TKU | 22471D | EG924462 | 85/90 | 85/90 | 85/90 | P | 5 | 4a | PET | 0.033 | 0.125 | 1 | results22471.html | - |  | AS-7 | CACGACGTTGTAAAACGACAAGAGCCGTTCGTTCTGGT | GTTTATAGTGCTCGCTGCTTGC |  |
| 69 | SsaIND016TKU | 7655N | EG926217 | 413/415 | 415 | 413/415 | P | 2 | 4b | PET | 0.05 | 0.2 | 2.5 | results7655.html | EG926217.htm | EG926217.pdf | AS-10 | CACGACGTTGTAAAACGACGCTGTTGCCCTGTTTTTGTT | GTTTATCGGGGAGCAGTTTCTTTT |  |
| 70 | SsaIND042TKU | 1729I | EG817916 | 357/359 | 359 | 357/359 | P | 2.2 | 4b | PET | 0.075 | 0.3 | 2.5 | results1729.html | - |  | AS-18 | CACGACGTTGTAAAACGACGGTACTCCGAGCAGAGCAAA | GTTTAGACCTGCACCCAAAAGAAA |  |
| 71 | SsaIND078TKU | 4493F | CA060073 | 474 | 474 | 472/474 | P | 2 | 4b | PET | 0.05 | 0.2 | 2.5 | results4493.html | - |  | AS-25 | CACGACGTTGTAAAACGACTCTTCCTGTGAAGCACGTTG | GTTTGTTTCGGGAATGTGGGTAT |  |
| 72 | SsaIND081TKU | 5134V | EG844218 | 558 | 558/564 | 558 | P | 6 | 4b | PET | 0.075 | 0.3 | 2.5 | results5134.html | - |  | - | CACGACGTTGTAAAACGACAAGTCATGTCCCCGACTCAC | GTTTCTTGGCGCTTTTGGTACAAT |  |
| 73 | SsaIND102TKU | 8921B | CA054270 | 370/376 | 368 | 370/376 | P | 2 | 4b | PET | 0.05 | 0.2 | 2.5 | results8921.html | - |  | - | CACGACGTTGTAAAACGACAACCCGTATTGGGGAAAGAC | GTTTCAGCACTGACCATCCACTGT |  |
| 74 | SsaIND126TKU | 13066I | EG807593 | 541/543 | 541 | 541/543 | P | 2 | 4b | PET | 0.075 | 0.3 | 2.5 | results13066.html | - |  | AS-10 | CACGACGTTGTAAAACGACCCGAGGCTTTTTACAGTTGG | GTTTCTCTGTTGGGTGGAAAAGG |  |
| 75 | SsaIND143TKU | 16730E | EG815522 | 487 | 487/493 | 487/493 | P | 6 | 4b | PET | 0.075 | 0.3 | 2.5 | results16730.html | - |  | - | CACGACGTTGTAAAACGACTTTCCAGACAATCCCAGACA | GTTTGGGGTTTGTTTTTGTGGAGA | microsatellite |
| 76 | SsaIND191TKU | 21965C | EG902270 | 392 | 392/398 | 392/398 | P | 6 | 4b | PET | 0.075 | 0.3 | 2.5 | results21965.html | - |  | - | CACGACGTTGTAAAACGACGGCCTGGATAATGGGAGAAT | GTTTGTCCTTGATGCCTCTGGAAA |  |
|  | | | | | | | | | | | | | | | | | | | | |
| NOTE: | P - polymorphism; | | |  | | | | | | | | | | | | | | | | |
|  | TA P - Trans-Atlantic polymorphism. | | | |  | | | | | | | | | | | | | | | |
|  | | | | | | | | | | | | | | | | | | | | |
|  | | | | | | | | | | | | | | | | | | | | |
|  | | | | | | |  |  | | | | | | | | | | | |  |
|  |  |  |  |  |  |  |  |  |  |  |  |  |  |  |  |  |  |  |  |  |
