# Supplementary material for: Discovery and application of insertion-deletion (INDEL) polymorphisms for QTL mapping of early life-history traits in Atlantic salmon
Source: BMC Genomics. 2010 Mar 8;11:156. doi: 10.1186/1471-2164-11-156 (PMC2838853; doi:10.1186/1471-2164-11-156)
Supplement: Additional file 2 — Information on developed 76 locus single-run INDEL panel in Atlantic salmon. Information on fluorescence labeling, primer concentrations, PCR pooling and links to alignments, INDEL motifs and GENESCAN (Burge and Karlin 1997) predictions of genes/exons are available in html format. [file 1471-2164-11-156-S2.ZIP › Additionalfile2/snpsummary10023.html]

```
Cluster 3442 Contig 1

prev  Summary    Contig List  next
```

Size of Consensus sequence = 1631

Number of sequences = 17

Minimum redundancy = 6

Key

A gi|117513707|gb|EG845466.1|EG845466 EST\_ssal\_eve\_58211 ssaleve thyroid Salmo salar cDNA Salmo salar cDNA clone ssal\_eve\_579\_131\_fwd 3', mRNA sequence  
B gi|117513706|gb|EG845465.1|EG845465 EST\_ssal\_eve\_58210 ssaleve thyroid Salmo salar cDNA Salmo salar cDNA clone ssal\_eve\_579\_131\_rev 5', mRNA sequence  
C gi|89863790|gb|DY719913.1|DY719913 EST\_ssal\_rgb2\_75652 ssalrgb2 mixed\_tissue Salmo salar cDNA Salmo salar cDNA clone ssal\_rgb2\_624\_147\_fwd 3', mRNA sequence  
D gi|45327062|gb|CK897329.1|CK897329 SGP134497 Atlantic salmon Testis cDNA library Salmo salar cDNA clone MG6-0355 5', mRNA sequence  
E gi|117449772|gb|EG781991.1|EG781991 EST\_ssal\_evd\_38575 ssalevd thymus Salmo salar cDNA Salmo salar cDNA clone ssal\_evd\_551\_202\_fwd 3', mRNA sequence  
F gi|117449773|gb|EG781992.1|EG781992 EST\_ssal\_evd\_38576 ssalevd thymus Salmo salar cDNA Salmo salar cDNA clone ssal\_evd\_551\_202\_rev 5', mRNA sequence  
G gi|85043802|gb|DW571980.1|DW571980 EST\_ssal\_rgb2\_36399 rgb2 Salmo salar cDNA clone ssal\_rgb2\_559\_126\_fwd 3', mRNA sequence  
H gi|85050176|gb|DW578354.1|DW578354 EST\_ssal\_rgb2\_42773 rgb2 Salmo salar cDNA clone ssal\_rgb2\_569\_129\_fwd 3', mRNA sequence  
I gi|117519632|gb|EG851360.1|EG851360 EST\_ssal\_eve\_8617 ssaleve thyroid Salmo salar cDNA Salmo salar cDNA clone ssal\_eve\_510\_252\_rev 5', mRNA sequence  
J gi|117519631|gb|EG851359.1|EG851359 EST\_ssal\_eve\_8616 ssaleve thyroid Salmo salar cDNA Salmo salar cDNA clone ssal\_eve\_510\_252\_fwd 3', mRNA sequence  
K gi|117503137|gb|EG834896.1|EG834896 EST\_ssal\_eve\_46700 ssaleve thyroid Salmo salar cDNA Salmo salar cDNA clone ssal\_eve\_563\_193\_rev 5', mRNA sequence  
L gi|117834492|gb|EG907188.1|EG907188 EST\_ssal\_evf\_9626 ssalevf mixed\_tissue Salmo salar cDNA Salmo salar cDNA clone ssal\_evf\_511\_090\_rev 5', mRNA sequence  
M gi|117834493|gb|EG907189.1|EG907189 EST\_ssal\_evf\_9627 ssalevf mixed\_tissue Salmo salar cDNA Salmo salar cDNA clone ssal\_evf\_511\_090\_fwd 3', mRNA sequence  
N gi|117503136|gb|EG834895.1|EG834895 EST\_ssal\_eve\_46699 ssaleve thyroid Salmo salar cDNA Salmo salar cDNA clone ssal\_eve\_563\_193\_fwd 3', mRNA sequence  
O gi|85043801|gb|DW571979.1|DW571979 EST\_ssal\_rgb2\_36398 rgb2 Salmo salar cDNA clone ssal\_rgb2\_559\_126\_rev 5', mRNA sequence  
P gi|89863791|gb|DY719914.1|DY719914 EST\_ssal\_rgb2\_75653 ssalrgb2 mixed\_tissue Salmo salar cDNA Salmo salar cDNA clone ssal\_rgb2\_624\_147\_rev 5', mRNA sequence  
Q gi|85050177|gb|DW578355.1|DW578355 EST\_ssal\_rgb2\_42774 rgb2 Salmo salar cDNA clone ssal\_rgb2\_569\_129\_rev 5', mRNA sequence

3 SNPs detected

A B C D E F G H I J K L M N O P Q  cosegregation weighted

380 - - - - A A - - A A A A . . . . .   3/3 70.59
381 - - - - A A - - A A A A . . . . .   3/3 70.59
382 - - - - G G - - G G G G . . . . .   3/3 70.59
