# Supplementary material for: Discovery and application of insertion-deletion (INDEL) polymorphisms for QTL mapping of early life-history traits in Atlantic salmon
Source: BMC Genomics. 2010 Mar 8;11:156. doi: 10.1186/1471-2164-11-156 (PMC2838853; doi:10.1186/1471-2164-11-156)
Supplement: Additional file 2 — Information on developed 76 locus single-run INDEL panel in Atlantic salmon. Information on fluorescence labeling, primer concentrations, PCR pooling and links to alignments, INDEL motifs and GENESCAN (Burge and Karlin 1997) predictions of genes/exons are available in html format. [file 1471-2164-11-156-S2.ZIP › Additionalfile2/snpsummary1021.html]

```
Cluster 126 Contig 2

prev  Summary    Contig List  next
```

Size of Consensus sequence = 2687

Number of sequences = 97

Minimum redundancy = 6

Key

A gi|45314837|gb|CK885206.1|CK885206 SGP165575 Atlantic salmon Intestine cDNA library Salmo salar cDNA clone T4-0704 5', mRNA sequence  
B gi|117567415|gb|EG888391.1|EG888391 EST\_ssal\_evf\_46454 ssalevf mixed\_tissue Salmo salar cDNA Salmo salar cDNA clone ssal\_evf\_562\_033\_fwd 3', mRNA sequence  
C gi|57126788|gb|CX358229.1|CX358229 ssalrgb536089\_rev\_0 mixed\_tissue Salmo salar cDNA, mRNA sequence  
D gi|85054246|gb|DW582424.1|DW582424 EST\_ssal\_rgb2\_46843 rgb2 Salmo salar cDNA clone ssal\_rgb2\_575\_271\_rev 5', mRNA sequence  
E gi|117864491|gb|EG937187.1|EG937187 EST\_ssal\_evf\_21036 ssalevf mixed\_tissue Salmo salar cDNA Salmo salar cDNA clone ssal\_evf\_526\_256\_rev 5', mRNA sequence  
F gi|117864492|gb|EG937188.1|EG937188 EST\_ssal\_evf\_21037 ssalevf mixed\_tissue Salmo salar cDNA Salmo salar cDNA clone ssal\_evf\_526\_256\_fwd 3', mRNA sequence  
G gi|117541302|gb|EG872747.1|EG872747 EST\_ssal\_eve\_33665 ssaleve thyroid Salmo salar cDNA Salmo salar cDNA clone ssal\_eve\_545\_269\_fwd 3', mRNA sequence  
H gi|117513234|gb|EG844993.1|EG844993 EST\_ssal\_eve\_37788 ssaleve thyroid Salmo salar cDNA Salmo salar cDNA clone ssal\_eve\_551\_172\_fwd 3', mRNA sequence  
I gi|117868189|gb|EG940885.1|EG940885 EST\_ssal\_evf\_24365 ssalevf mixed\_tissue Salmo salar cDNA Salmo salar cDNA clone ssal\_evf\_532\_092\_fwd 3', mRNA sequence  
J gi|45323925|gb|CK894192.1|CK894192 SGP159797 Atlantic salmon Spleen cDNA library Salmo salar cDNA clone MI5-0202 5', mRNA sequence  
K gi|117860553|gb|EG933249.1|EG933249 EST\_ssal\_evf\_35082 ssalevf mixed\_tissue Salmo salar cDNA Salmo salar cDNA clone ssal\_evf\_546\_264\_fwd 3', mRNA sequence  
L gi|85042401|gb|DW570579.1|DW570579 EST\_ssal\_rgb2\_34998 rgb2 Salmo salar cDNA clone ssal\_rgb2\_556\_243\_rev 5', mRNA sequence  
M gi|89880793|gb|DY736916.1|DY736916 EST\_ssal\_rgb2\_92655 ssalrgb2 mixed\_tissue Salmo salar cDNA Salmo salar cDNA clone ssal\_rgb2\_651\_203\_rev 5', mRNA sequence  
N gi|117837160|gb|EG909856.1|EG909856 EST\_ssal\_evf\_12027 ssalevf mixed\_tissue Salmo salar cDNA Salmo salar cDNA clone ssal\_evf\_514\_210\_fwd 3', mRNA sequence  
O gi|45326442|gb|CK896709.1|CK896709 SGP159261 Atlantic salmon Swimbladder cDNA library Salmo salar cDNA clone SB5-0671 5', mRNA sequence  
P gi|85042062|gb|DW570240.1|DW570240 EST\_ssal\_rgb2\_34659 rgb2 Salmo salar cDNA clone ssal\_rgb2\_556\_039\_rev 5', mRNA sequence  
Q gi|117852878|gb|EG925574.1|EG925574 EST\_ssal\_evf\_28174 ssalevf mixed\_tissue Salmo salar cDNA Salmo salar cDNA clone ssal\_evf\_537\_136\_fwd 3', mRNA sequence  
R gi|45316195|gb|CK886564.1|CK886564 SGP148423 Atlantic salmon Kidney cDNA library Salmo salar cDNA clone BN7-0534 5', mRNA sequence  
S gi|117427094|gb|EG759318.1|EG759318 EST\_ssal\_sjb\_7793 ssalsjb mixed\_tissue Salmo salar cDNA Salmo salar cDNA clone ssal\_sjb\_016\_171\_fwd 3', mRNA sequence  
T gi|76594457|gb|DV106128.1|DV106128 SGP280710 Atlantic salmon Liver cDNA library Salmo salar cDNA clone L4-1524 5', mRNA sequence  
U gi|117851978|gb|EG924674.1|EG924674 EST\_ssal\_evf\_27364 ssalevf mixed\_tissue Salmo salar cDNA Salmo salar cDNA clone ssal\_evf\_536\_105\_fwd 3', mRNA sequence  
V gi|85048165|gb|DW576343.1|DW576343 EST\_ssal\_rgb2\_40762 rgb2 Salmo salar cDNA clone ssal\_rgb2\_566\_077\_rev 5', mRNA sequence  
W gi|117836434|gb|EG909130.1|EG909130 EST\_ssal\_evf\_53922 ssalevf mixed\_tissue Salmo salar cDNA Salmo salar cDNA clone ssal\_evf\_572\_094\_fwd 3', mRNA sequence  
X gi|117436020|gb|EG768243.1|EG768243 EST\_ssal\_evd\_44202 ssalevd thymus Salmo salar cDNA Salmo salar cDNA clone ssal\_evd\_559\_128\_fwd 3', mRNA sequence  
Y gi|89874304|gb|DY730427.1|DY730427 EST\_ssal\_rgb2\_86166 ssalrgb2 mixed\_tissue Salmo salar cDNA Salmo salar cDNA clone ssal\_rgb2\_640\_376\_rev 5', mRNA sequence  
Z gi|117426030|gb|EG758254.1|EG758254 EST\_ssal\_sjb\_6834 ssalsjb mixed\_tissue Salmo salar cDNA Salmo salar cDNA clone ssal\_sjb\_014\_377\_fwd 3', mRNA sequence  
a gi|117865207|gb|EG937903.1|EG937903 EST\_ssal\_evf\_21682 ssalevf mixed\_tissue Salmo salar cDNA Salmo salar cDNA clone ssal\_evf\_527\_201\_fwd 3', mRNA sequence  
b gi|117821736|gb|EG894432.1|EG894432 EST\_ssal\_evf\_51890 ssalevf mixed\_tissue Salmo salar cDNA Salmo salar cDNA clone ssal\_evf\_569\_179\_fwd 3', mRNA sequence  
c gi|117866344|gb|EG939040.1|EG939040 EST\_ssal\_evf\_22705 ssalevf mixed\_tissue Salmo salar cDNA Salmo salar cDNA clone ssal\_evf\_529\_345\_fwd 3', mRNA sequence  
d gi|117848351|gb|EG921047.1|EG921047 EST\_ssal\_evf\_299 ssalevf mixed\_tissue Salmo salar cDNA Salmo salar cDNA clone ssal\_evf\_002\_066\_fwd 3', mRNA sequence  
e gi|59834557|gb|DN140240.1|DN140240 SGP266265 Atlantic salmon Head kidney cDNA library Salmo salar cDNA clone FN4-3619 5', mRNA sequence  
f gi|117832353|gb|EG905049.1|EG905049 EST\_ssal\_evf\_7702 ssalevf mixed\_tissue Salmo salar cDNA Salmo salar cDNA clone ssal\_evf\_508\_241\_fwd 3', mRNA sequence  
g gi|117503082|gb|EG834841.1|EG834841 EST\_ssal\_eve\_46650 ssaleve thyroid Salmo salar cDNA Salmo salar cDNA clone ssal\_eve\_563\_168\_fwd 3', mRNA sequence  
h gi|117862934|gb|EG935630.1|EG935630 EST\_ssal\_evf\_17007 ssalevf mixed\_tissue Salmo salar cDNA Salmo salar cDNA clone ssal\_evf\_521\_102\_fwd 3', mRNA sequence  
i gi|117859835|gb|EG932531.1|EG932531 EST\_ssal\_evf\_34435 ssalevf mixed\_tissue Salmo salar cDNA Salmo salar cDNA clone ssal\_evf\_545\_315\_fwd 3', mRNA sequence  
j gi|85042400|gb|DW570578.1|DW570578 EST\_ssal\_rgb2\_34997 rgb2 Salmo salar cDNA clone ssal\_rgb2\_556\_243\_fwd 3', mRNA sequence  
k gi|84975337|gb|DW473738.1|DW473738 SGP315714 Atlantic salmon Spleen cDNA library Salmo salar cDNA clone MI5-3936 5', mRNA sequence  
l gi|117829732|gb|EG902428.1|EG902428 EST\_ssal\_evf\_2920 ssalevf mixed\_tissue Salmo salar cDNA Salmo salar cDNA clone ssal\_evf\_502\_075\_fwd 3', mRNA sequence  
m gi|117445421|gb|EG777644.1|EG777644 EST\_ssal\_evd\_4263 ssalevd thymus Salmo salar cDNA Salmo salar cDNA clone ssal\_evd\_504\_134\_rev 5', mRNA sequence  
n gi|117445422|gb|EG777645.1|EG777645 EST\_ssal\_evd\_4264 ssalevd thymus Salmo salar cDNA Salmo salar cDNA clone ssal\_evd\_504\_134\_fwd 3', mRNA sequence  
o gi|117525188|gb|EG856915.1|EG856915 EST\_ssal\_eve\_50514 ssaleve thyroid Salmo salar cDNA Salmo salar cDNA clone ssal\_eve\_568\_300\_fwd 3', mRNA sequence  
p gi|85042063|gb|DW570241.1|DW570241 EST\_ssal\_rgb2\_34660 rgb2 Salmo salar cDNA clone ssal\_rgb2\_556\_039\_fwd 3', mRNA sequence  
q gi|85054245|gb|DW582423.1|DW582423 EST\_ssal\_rgb2\_46842 rgb2 Salmo salar cDNA clone ssal\_rgb2\_575\_271\_fwd 3', mRNA sequence  
r gi|117427095|gb|EG759319.1|EG759319 EST\_ssal\_sjb\_7794 ssalsjb mixed\_tissue Salmo salar cDNA Salmo salar cDNA clone ssal\_sjb\_016\_171\_rev 5', mRNA sequence  
s gi|24388733|gb|CA058490.1|CA058490 ssalrga513291 mixed\_tissue Salmo salar cDNA, mRNA sequence  
t gi|24386467|gb|CA056224.1|CA056224 ssalrgb536089 mixed\_tissue Salmo salar cDNA, mRNA sequence  
u gi|117857953|gb|EG930649.1|EG930649 EST\_ssal\_evf\_32741 ssalevf mixed\_tissue Salmo salar cDNA Salmo salar cDNA clone ssal\_evf\_543\_201\_fwd 3', mRNA sequence  
v gi|117853141|gb|EG925837.1|EG925837 EST\_ssal\_evf\_28411 ssalevf mixed\_tissue Salmo salar cDNA Salmo salar cDNA clone ssal\_evf\_537\_257\_fwd 3', mRNA sequence  
w gi|117823123|gb|EG895819.1|EG895819 EST\_ssal\_evf\_4737 ssalevf mixed\_tissue Salmo salar cDNA Salmo salar cDNA clone ssal\_evf\_504\_250\_fwd 3', mRNA sequence  
x gi|117862945|gb|EG935641.1|EG935641 EST\_ssal\_evf\_17008 ssalevf mixed\_tissue Salmo salar cDNA Salmo salar cDNA clone ssal\_evf\_521\_102\_rev 5', mRNA sequence  
y gi|117513233|gb|EG844992.1|EG844992 EST\_ssal\_eve\_37787 ssaleve thyroid Salmo salar cDNA Salmo salar cDNA clone ssal\_eve\_551\_172\_rev 5', mRNA sequence  
z gi|117859836|gb|EG932532.1|EG932532 EST\_ssal\_evf\_34436 ssalevf mixed\_tissue Salmo salar cDNA Salmo salar cDNA clone ssal\_evf\_545\_315\_rev 5', mRNA sequence  
A gi|117866342|gb|EG939038.1|EG939038 EST\_ssal\_evf\_22704 ssalevf mixed\_tissue Salmo salar cDNA Salmo salar cDNA clone ssal\_evf\_529\_345\_rev 5', mRNA sequence  
B gi|117823124|gb|EG895820.1|EG895820 EST\_ssal\_evf\_4738 ssalevf mixed\_tissue Salmo salar cDNA Salmo salar cDNA clone ssal\_evf\_504\_250\_rev 5', mRNA sequence  
C gi|24341213|gb|CA040287.1|CA040287 ssalshc503337 spleen Salmo salar cDNA, mRNA sequence  
D gi|117831468|gb|EG904164.1|EG904164 EST\_ssal\_evf\_53475 ssalevf mixed\_tissue Salmo salar cDNA Salmo salar cDNA clone ssal\_evf\_571\_247\_rev 5', mRNA sequence  
E gi|117831480|gb|EG904176.1|EG904176 EST\_ssal\_evf\_53476 ssalevf mixed\_tissue Salmo salar cDNA Salmo salar cDNA clone ssal\_evf\_571\_247\_fwd 3', mRNA sequence  
F gi|117836423|gb|EG909119.1|EG909119 EST\_ssal\_evf\_53921 ssalevf mixed\_tissue Salmo salar cDNA Salmo salar cDNA clone ssal\_evf\_572\_094\_rev 5', mRNA sequence  
G gi|117860552|gb|EG933248.1|EG933248 EST\_ssal\_evf\_35081 ssalevf mixed\_tissue Salmo salar cDNA Salmo salar cDNA clone ssal\_evf\_546\_264\_rev 5', mRNA sequence  
H gi|117829743|gb|EG902439.1|EG902439 EST\_ssal\_evf\_2921 ssalevf mixed\_tissue Salmo salar cDNA Salmo salar cDNA clone ssal\_evf\_502\_075\_rev 5', mRNA sequence  
I gi|117821737|gb|EG894433.1|EG894433 EST\_ssal\_evf\_51891 ssalevf mixed\_tissue Salmo salar cDNA Salmo salar cDNA clone ssal\_evf\_569\_179\_rev 5', mRNA sequence  
J gi|117821793|gb|EG894489.1|EG894489 EST\_ssal\_evf\_51942 ssalevf mixed\_tissue Salmo salar cDNA Salmo salar cDNA clone ssal\_evf\_569\_206\_fwd 3', mRNA sequence  
K gi|117821794|gb|EG894490.1|EG894490 EST\_ssal\_evf\_51943 ssalevf mixed\_tissue Salmo salar cDNA Salmo salar cDNA clone ssal\_evf\_569\_206\_rev 5', mRNA sequence  
L gi|117525189|gb|EG856916.1|EG856916 EST\_ssal\_eve\_50515 ssaleve thyroid Salmo salar cDNA Salmo salar cDNA clone ssal\_eve\_568\_300\_rev 5', mRNA sequence  
M gi|117850424|gb|EG923120.1|EG923120 EST\_ssal\_evf\_53381 ssalevf mixed\_tissue Salmo salar cDNA Salmo salar cDNA clone ssal\_evf\_571\_198\_rev 5', mRNA sequence  
N gi|117436021|gb|EG768244.1|EG768244 EST\_ssal\_evd\_44203 ssalevd thymus Salmo salar cDNA Salmo salar cDNA clone ssal\_evd\_559\_128\_rev 5', mRNA sequence  
O gi|45316850|gb|CK887219.1|CK887219 SGP149139 Atlantic salmon Kidney cDNA library Salmo salar cDNA clone BN6-0834 5', mRNA sequence  
P gi|117567414|gb|EG888390.1|EG888390 EST\_ssal\_evf\_46453 ssalevf mixed\_tissue Salmo salar cDNA Salmo salar cDNA clone ssal\_evf\_562\_033\_rev 5', mRNA sequence  
Q gi|117503081|gb|EG834840.1|EG834840 EST\_ssal\_eve\_46649 ssaleve thyroid Salmo salar cDNA Salmo salar cDNA clone ssal\_eve\_563\_168\_rev 5', mRNA sequence  
R gi|117541303|gb|EG872748.1|EG872748 EST\_ssal\_eve\_33666 ssaleve thyroid Salmo salar cDNA Salmo salar cDNA clone ssal\_eve\_545\_269\_rev 5', mRNA sequence  
S gi|117865206|gb|EG937902.1|EG937902 EST\_ssal\_evf\_21681 ssalevf mixed\_tissue Salmo salar cDNA Salmo salar cDNA clone ssal\_evf\_527\_201\_rev 5', mRNA sequence  
T gi|117868190|gb|EG940886.1|EG940886 EST\_ssal\_evf\_24366 ssalevf mixed\_tissue Salmo salar cDNA Salmo salar cDNA clone ssal\_evf\_532\_092\_rev 5', mRNA sequence  
U gi|85048166|gb|DW576344.1|DW576344 EST\_ssal\_rgb2\_40763 rgb2 Salmo salar cDNA clone ssal\_rgb2\_566\_077\_fwd 3', mRNA sequence  
V gi|117852880|gb|EG925576.1|EG925576 EST\_ssal\_evf\_28175 ssalevf mixed\_tissue Salmo salar cDNA Salmo salar cDNA clone ssal\_evf\_537\_136\_rev 5', mRNA sequence  
W gi|117513990|gb|EG845749.1|EG845749 EST\_ssal\_eve\_58466 ssaleve thyroid Salmo salar cDNA Salmo salar cDNA clone ssal\_eve\_579\_264\_fwd 3', mRNA sequence  
X gi|117513991|gb|EG845750.1|EG845750 EST\_ssal\_eve\_58467 ssaleve thyroid Salmo salar cDNA Salmo salar cDNA clone ssal\_eve\_579\_264\_rev 5', mRNA sequence  
Y gi|117832354|gb|EG905050.1|EG905050 EST\_ssal\_evf\_7703 ssalevf mixed\_tissue Salmo salar cDNA Salmo salar cDNA clone ssal\_evf\_508\_241\_rev 5', mRNA sequence  
Z gi|117851980|gb|EG924676.1|EG924676 EST\_ssal\_evf\_27365 ssalevf mixed\_tissue Salmo salar cDNA Salmo salar cDNA clone ssal\_evf\_536\_105\_rev 5', mRNA sequence  
a gi|117456934|gb|EG789153.1|EG789153 EST\_ssal\_evd\_9558 ssalevd thymus Salmo salar cDNA Salmo salar cDNA clone ssal\_evd\_511\_210\_rev 5', mRNA sequence  
b gi|89880792|gb|DY736915.1|DY736915 EST\_ssal\_rgb2\_92654 ssalrgb2 mixed\_tissue Salmo salar cDNA Salmo salar cDNA clone ssal\_rgb2\_651\_203\_fwd 3', mRNA sequence  
c gi|29316605|gb|CB505379.1|CB505379 ssalmge504160 gut Salmo salar cDNA, mRNA sequence  
d gi|117495833|gb|EG828050.1|EG828050 EST\_ssal\_eve\_40537 ssaleve thyroid Salmo salar cDNA Salmo salar cDNA clone ssal\_eve\_555\_062\_rev 5', mRNA sequence  
e gi|45326868|gb|CK897135.1|CK897135 SGP159697 Atlantic salmon Swimbladder cDNA library Salmo salar cDNA clone SB5-1283 5', mRNA sequence  
f gi|117495834|gb|EG828051.1|EG828051 EST\_ssal\_eve\_40538 ssaleve thyroid Salmo salar cDNA Salmo salar cDNA clone ssal\_eve\_555\_062\_fwd 3', mRNA sequence  
g gi|117525175|gb|EG856902.1|EG856902 EST\_ssal\_eve\_50502 ssaleve thyroid Salmo salar cDNA Salmo salar cDNA clone ssal\_eve\_568\_293\_rev 5', mRNA sequence  
h gi|117525176|gb|EG856903.1|EG856903 EST\_ssal\_eve\_50503 ssaleve thyroid Salmo salar cDNA Salmo salar cDNA clone ssal\_eve\_568\_293\_fwd 3', mRNA sequence  
i gi|89874303|gb|DY730426.1|DY730426 EST\_ssal\_rgb2\_86165 ssalrgb2 mixed\_tissue Salmo salar cDNA Salmo salar cDNA clone ssal\_rgb2\_640\_376\_fwd 3', mRNA sequence  
j gi|24343382|gb|CA042477.1|CA042477 ssalplnb501196 gut Salmo salar cDNA, mRNA sequence  
k gi|117853140|gb|EG925836.1|EG925836 EST\_ssal\_evf\_28410 ssalevf mixed\_tissue Salmo salar cDNA Salmo salar cDNA clone ssal\_evf\_537\_257\_rev 5', mRNA sequence  
l gi|117440941|gb|EG773164.1|EG773164 EST\_ssal\_evd\_48632 ssalevd thymus Salmo salar cDNA Salmo salar cDNA clone ssal\_evd\_565\_148\_fwd 3', mRNA sequence  
m gi|117440942|gb|EG773165.1|EG773165 EST\_ssal\_evd\_48633 ssalevd thymus Salmo salar cDNA Salmo salar cDNA clone ssal\_evd\_565\_148\_rev 5', mRNA sequence  
n gi|117857954|gb|EG930650.1|EG930650 EST\_ssal\_evf\_32742 ssalevf mixed\_tissue Salmo salar cDNA Salmo salar cDNA clone ssal\_evf\_543\_201\_rev 5', mRNA sequence  
o gi|117492162|gb|EG824379.1|EG824379 EST\_ssal\_evd\_25921 ssalevd thymus Salmo salar cDNA Salmo salar cDNA clone ssal\_evd\_533\_290\_rev 5', mRNA sequence  
p gi|117492163|gb|EG824380.1|EG824380 EST\_ssal\_evd\_25922 ssalevd thymus Salmo salar cDNA Salmo salar cDNA clone ssal\_evd\_533\_290\_fwd 3', mRNA sequence  
q gi|117426031|gb|EG758255.1|EG758255 EST\_ssal\_sjb\_6835 ssalsjb mixed\_tissue Salmo salar cDNA Salmo salar cDNA clone ssal\_sjb\_014\_377\_rev 5', mRNA sequence  
r gi|117848350|gb|EG921046.1|EG921046 EST\_ssal\_evf\_298 ssalevf mixed\_tissue Salmo salar cDNA Salmo salar cDNA clone ssal\_evf\_002\_066\_rev 5', mRNA sequence  
s gi|45328614|gb|CK898881.1|CK898881 SGP162465 Atlantic salmon Testis cDNA library Salmo salar cDNA clone MG4-1148 5', mRNA sequence

3 SNPs detected

A B C D E F G H I J K L M N O P Q R S T U V W X Y Z a b c d e f g h i j k l m n o p q r s t u v w x y z A B C D E F G H I J K L M N O P Q R S T U V W X Y Z a b c d e f g h i j k l m n o p q r s  cosegregation weighted

2374 . . . . . . . . . . . . . . . . . . . . . . . . . . . . . . . - - - - T C - - - - T T C C C C C - - - - - - - - - - - - - - - - - C C - - C C C T - - - - - C T C C C C C C C C C C C C C C C C C   1/3 22.68
2375 . . . . . . . . . . . . . . . . . . . . . . . . . . . . . . . - - - - T T - - - - T T T T T T T - - - - - - - - - - - - - - - - - T T - - T T T T - - - - - T T T T T T T T T T T T T T T T T C T   1/3 22.68
2657 . . . . . . . . . . . . . . . . . . . . . . . . . . . . . . . . . . . . . . . . . . . . . . . . . C . . C T T T T T C T T . . C C C . T C C T C . T . . T T . T T T T T T T T T T T T T T T T T T   1/3 13.40
