# Supplementary material for: Discovery and application of insertion-deletion (INDEL) polymorphisms for QTL mapping of early life-history traits in Atlantic salmon
Source: BMC Genomics. 2010 Mar 8;11:156. doi: 10.1186/1471-2164-11-156 (PMC2838853; doi:10.1186/1471-2164-11-156)
Supplement: Additional file 2 — Information on developed 76 locus single-run INDEL panel in Atlantic salmon. Information on fluorescence labeling, primer concentrations, PCR pooling and links to alignments, INDEL motifs and GENESCAN (Burge and Karlin 1997) predictions of genes/exons are available in html format. [file 1471-2164-11-156-S2.ZIP › Additionalfile2/snpsummary10259.html]

```
Cluster 3568 Contig 1

prev  Summary    Contig List  next
```

Size of Consensus sequence = 1278

Number of sequences = 17

Minimum redundancy = 6

Key

A gi|117842771|gb|EG915467.1|EG915467 EST\_ssal\_evf\_54816 ssalevf mixed\_tissue Salmo salar cDNA Salmo salar cDNA clone ssal\_evf\_573\_186\_rev 5', mRNA sequence  
B gi|117860631|gb|EG933327.1|EG933327 EST\_ssal\_evf\_35152 ssalevf mixed\_tissue Salmo salar cDNA Salmo salar cDNA clone ssal\_evf\_546\_300\_rev 5', mRNA sequence  
C gi|85049679|gb|DW577857.1|DW577857 EST\_ssal\_rgb2\_42276 rgb2 Salmo salar cDNA clone ssal\_rgb2\_568\_195\_fwd 3', mRNA sequence  
D gi|84568902|gb|DW340521.1|DW340521 SGP310596 Atlantic salmon Ovaries cDNA library Salmo salar cDNA clone KG4-3851 5', mRNA sequence  
E gi|117448263|gb|EG780482.1|EG780482 EST\_ssal\_evd\_37217 ssalevd thymus Salmo salar cDNA Salmo salar cDNA clone ssal\_evd\_549\_261\_rev 5', mRNA sequence  
F gi|117427121|gb|EG759345.1|EG759345 EST\_ssal\_sjb\_7816 ssalsjb mixed\_tissue Salmo salar cDNA Salmo salar cDNA clone ssal\_sjb\_016\_183\_rev 5', mRNA sequence  
G gi|117456257|gb|EG788476.1|EG788476 EST\_ssal\_evd\_8948 ssalevd thymus Salmo salar cDNA Salmo salar cDNA clone ssal\_evd\_510\_275\_rev 5', mRNA sequence  
H gi|117508871|gb|EG840630.1|EG840630 EST\_ssal\_eve\_9605 ssaleve thyroid Salmo salar cDNA Salmo salar cDNA clone ssal\_eve\_512\_382\_rev 5', mRNA sequence  
I gi|117429044|gb|EG761268.1|EG761268 EST\_ssal\_sjb\_9547 ssalsjb mixed\_tissue Salmo salar cDNA Salmo salar cDNA clone ssal\_sjb\_020\_073\_rev 5', mRNA sequence  
J gi|117456258|gb|EG788477.1|EG788477 EST\_ssal\_evd\_8949 ssalevd thymus Salmo salar cDNA Salmo salar cDNA clone ssal\_evd\_510\_275\_fwd 3', mRNA sequence  
K gi|117860630|gb|EG933326.1|EG933326 EST\_ssal\_evf\_35151 ssalevf mixed\_tissue Salmo salar cDNA Salmo salar cDNA clone ssal\_evf\_546\_300\_fwd 3', mRNA sequence  
L gi|29320304|gb|CB508578.1|CB508578 ssalob509191 reproductive Salmo salar cDNA, mRNA sequence  
M gi|24342791|gb|CA041871.1|CA041871 ssalmgf001052 gut Salmo salar cDNA, mRNA sequence  
N gi|117429042|gb|EG761266.1|EG761266 EST\_ssal\_sjb\_9546 ssalsjb mixed\_tissue Salmo salar cDNA Salmo salar cDNA clone ssal\_sjb\_020\_073\_fwd 3', mRNA sequence  
O gi|117508860|gb|EG840619.1|EG840619 EST\_ssal\_eve\_9604 ssaleve thyroid Salmo salar cDNA Salmo salar cDNA clone ssal\_eve\_512\_382\_fwd 3', mRNA sequence  
P gi|117842772|gb|EG915468.1|EG915468 EST\_ssal\_evf\_54817 ssalevf mixed\_tissue Salmo salar cDNA Salmo salar cDNA clone ssal\_evf\_573\_186\_fwd 3', mRNA sequence  
Q gi|117448264|gb|EG780483.1|EG780483 EST\_ssal\_evd\_37218 ssalevd thymus Salmo salar cDNA Salmo salar cDNA clone ssal\_evd\_549\_261\_fwd 3', mRNA sequence

5 SNPs detected

A B C D E F G H I J K L M N O P Q  cosegregation weighted

528 . A - - - - A A - A A A - - . . .   5/5 76.47
529 . G - - - - G G - G G G - - . . .   5/5 76.47
530 . G - - - - G G - G G G - - . . .   5/5 76.47
531 . A - - - - A A - A A A - - . . .   5/5 76.47
532 . A - - - - A A - A A A - - . . .   5/5 76.47
