# Supplementary material for: Discovery and application of insertion-deletion (INDEL) polymorphisms for QTL mapping of early life-history traits in Atlantic salmon
Source: BMC Genomics. 2010 Mar 8;11:156. doi: 10.1186/1471-2164-11-156 (PMC2838853; doi:10.1186/1471-2164-11-156)
Supplement: Additional file 2 — Information on developed 76 locus single-run INDEL panel in Atlantic salmon. Information on fluorescence labeling, primer concentrations, PCR pooling and links to alignments, INDEL motifs and GENESCAN (Burge and Karlin 1997) predictions of genes/exons are available in html format. [file 1471-2164-11-156-S2.ZIP › Additionalfile2/snpsummary10585.html]

```
Cluster 3740 Contig 1

prev  Summary    Contig List  next
```

Size of Consensus sequence = 1456

Number of sequences = 5

Minimum redundancy = 2

Key

A gi|117429264|gb|EG761488.1|EG761488 EST\_ssal\_sjb\_9746 ssalsjb mixed\_tissue Salmo salar cDNA Salmo salar cDNA clone ssal\_sjb\_020\_189\_fwd 3', mRNA sequence  
B gi|24382456|gb|CA052213.1|CA052213 ssalrga507113 mixed\_tissue Salmo salar cDNA, mRNA sequence  
C gi|117429266|gb|EG761490.1|EG761490 EST\_ssal\_sjb\_9747 ssalsjb mixed\_tissue Salmo salar cDNA Salmo salar cDNA clone ssal\_sjb\_020\_189\_rev 5', mRNA sequence  
D gi|85052293|gb|DW580471.1|DW580471 EST\_ssal\_rgb2\_44890 rgb2 Salmo salar cDNA clone ssal\_rgb2\_572\_264\_fwd 3', mRNA sequence  
E gi|24380600|gb|CA050357.1|CA050357 ssalrgb520260 mixed\_tissue Salmo salar cDNA, mRNA sequence

2 SNPs detected

A B C D E  cosegregation weighted

543 T - T - .   2/2 80.00
544 C - C - .   2/2 80.00
