# Supplementary material for: Discovery and application of insertion-deletion (INDEL) polymorphisms for QTL mapping of early life-history traits in Atlantic salmon
Source: BMC Genomics. 2010 Mar 8;11:156. doi: 10.1186/1471-2164-11-156 (PMC2838853; doi:10.1186/1471-2164-11-156)
Supplement: Additional file 2 — Information on developed 76 locus single-run INDEL panel in Atlantic salmon. Information on fluorescence labeling, primer concentrations, PCR pooling and links to alignments, INDEL motifs and GENESCAN (Burge and Karlin 1997) predictions of genes/exons are available in html format. [file 1471-2164-11-156-S2.ZIP › Additionalfile2/snpsummary10789.html]

```
Cluster 3850 Contig 2

prev  Summary    Contig List  next
```

Size of Consensus sequence = 807

Number of sequences = 5

Minimum redundancy = 2

Key

A gi|89840549|gb|DY698348.1|DY698348 EST\_ssal\_rgb2\_54087 ssalrgb2 mixed\_tissue Salmo salar cDNA Salmo salar cDNA clone ssal\_rgb2\_587\_173\_rev 5', mRNA sequence  
B gi|117433884|gb|EG766107.1|EG766107 EST\_ssal\_evd\_42279 ssalevd thymus Salmo salar cDNA Salmo salar cDNA clone ssal\_evd\_556\_250\_fwd 3', mRNA sequence  
C gi|117472978|gb|EG805197.1|EG805197 EST\_ssal\_evd\_5997 ssalevd thymus Salmo salar cDNA Salmo salar cDNA clone ssal\_evd\_506\_272\_fwd 3', mRNA sequence  
D gi|117472977|gb|EG805196.1|EG805196 EST\_ssal\_evd\_5996 ssalevd thymus Salmo salar cDNA Salmo salar cDNA clone ssal\_evd\_506\_272\_rev 5', mRNA sequence  
E gi|29311511|gb|CB500285.1|CB500285 ssalbrh003027 head Salmo salar cDNA, mRNA sequence

2 SNPs detected

A B C D E  cosegregation weighted

498 . C - - C   2/2 80.00
499 . T - - T   2/2 80.00
