# Supplementary material for: Discovery and application of insertion-deletion (INDEL) polymorphisms for QTL mapping of early life-history traits in Atlantic salmon
Source: BMC Genomics. 2010 Mar 8;11:156. doi: 10.1186/1471-2164-11-156 (PMC2838853; doi:10.1186/1471-2164-11-156)
Supplement: Additional file 2 — Information on developed 76 locus single-run INDEL panel in Atlantic salmon. Information on fluorescence labeling, primer concentrations, PCR pooling and links to alignments, INDEL motifs and GENESCAN (Burge and Karlin 1997) predictions of genes/exons are available in html format. [file 1471-2164-11-156-S2.ZIP › Additionalfile2/snpsummary1094.html]

```
Cluster 139 Contig 2

prev  Summary    Contig List  next
```

Size of Consensus sequence = 1531

Number of sequences = 103

Minimum redundancy = 6

Key

A gi|117441759|gb|EG773982.1|EG773982 EST\_ssal\_evd\_49368 ssalevd thymus Salmo salar cDNA Salmo salar cDNA clone ssal\_evd\_566\_159\_rev 5', mRNA sequence  
B gi|117528146|gb|EG859787.1|EG859787 EST\_ssal\_eve\_48528 ssaleve thyroid Salmo salar cDNA Salmo salar cDNA clone ssal\_eve\_566\_022\_rev 5', mRNA sequence  
C gi|117528157|gb|EG859798.1|EG859798 EST\_ssal\_eve\_48529 ssaleve thyroid Salmo salar cDNA Salmo salar cDNA clone ssal\_eve\_566\_022\_fwd 3', mRNA sequence  
D gi|117543392|gb|EG874837.1|EG874837 EST\_ssal\_eve\_35546 ssaleve thyroid Salmo salar cDNA Salmo salar cDNA clone ssal\_eve\_548\_114\_rev 5', mRNA sequence  
E gi|117447244|gb|EG779463.1|EG779463 EST\_ssal\_evd\_36300 ssalevd thymus Salmo salar cDNA Salmo salar cDNA clone ssal\_evd\_548\_175\_rev 5', mRNA sequence  
F gi|117459312|gb|EG791531.1|EG791531 EST\_ssal\_evd\_11699 ssalevd thymus Salmo salar cDNA Salmo salar cDNA clone ssal\_evd\_514\_194\_fwd 3', mRNA sequence  
G gi|117511543|gb|EG843302.1|EG843302 EST\_ssal\_eve\_36265 ssaleve thyroid Salmo salar cDNA Salmo salar cDNA clone ssal\_eve\_549\_123\_rev 5', mRNA sequence  
H gi|89874593|gb|DY730716.1|DY730716 EST\_ssal\_rgb2\_86455 ssalrgb2 mixed\_tissue Salmo salar cDNA Salmo salar cDNA clone ssal\_rgb2\_641\_164\_fwd 3', mRNA sequence  
I gi|117527600|gb|EG859241.1|EG859241 EST\_ssal\_eve\_52609 ssaleve thyroid Salmo salar cDNA Salmo salar cDNA clone ssal\_eve\_571\_256\_rev 5', mRNA sequence  
J gi|117512557|gb|EG844316.1|EG844316 EST\_ssal\_eve\_37178 ssaleve thyroid Salmo salar cDNA Salmo salar cDNA clone ssal\_eve\_550\_233\_rev 5', mRNA sequence  
K gi|117549247|gb|EG880692.1|EG880692 EST\_ssal\_eve\_23983 ssaleve thyroid Salmo salar cDNA Salmo salar cDNA clone ssal\_eve\_532\_259\_rev 5', mRNA sequence  
L gi|117439138|gb|EG771361.1|EG771361 EST\_ssal\_evd\_47009 ssalevd thymus Salmo salar cDNA Salmo salar cDNA clone ssal\_evd\_563\_065\_rev 5', mRNA sequence  
M gi|117848512|gb|EG921208.1|EG921208 EST\_ssal\_evf\_444 ssalevf mixed\_tissue Salmo salar cDNA Salmo salar cDNA clone ssal\_evf\_003\_047\_fwd 3', mRNA sequence  
N gi|117461925|gb|EG794144.1|EG794144 EST\_ssal\_evd\_53908 ssalevd thymus Salmo salar cDNA Salmo salar cDNA clone ssal\_evd\_572\_251\_rev 5', mRNA sequence  
O gi|117467382|gb|EG799601.1|EG799601 EST\_ssal\_evd\_52599 ssalevd thymus Salmo salar cDNA Salmo salar cDNA clone ssal\_evd\_570\_343\_rev 5', mRNA sequence  
P gi|117467393|gb|EG799612.1|EG799612 EST\_ssal\_evd\_52600 ssalevd thymus Salmo salar cDNA Salmo salar cDNA clone ssal\_evd\_570\_343\_fwd 3', mRNA sequence  
Q gi|117463455|gb|EG795674.1|EG795674 EST\_ssal\_evd\_15427 ssalevd thymus Salmo salar cDNA Salmo salar cDNA clone ssal\_evd\_519\_225\_rev 5', mRNA sequence  
R gi|117455820|gb|EG788039.1|EG788039 EST\_ssal\_evd\_8555 ssalevd thymus Salmo salar cDNA Salmo salar cDNA clone ssal\_evd\_510\_067\_rev 5', mRNA sequence  
S gi|117461037|gb|EG793256.1|EG793256 EST\_ssal\_evd\_13250 ssalevd thymus Salmo salar cDNA Salmo salar cDNA clone ssal\_evd\_516\_229\_rev 5', mRNA sequence  
T gi|117458443|gb|EG790662.1|EG790662 EST\_ssal\_evd\_10915 ssalevd thymus Salmo salar cDNA Salmo salar cDNA clone ssal\_evd\_513\_162\_rev 5', mRNA sequence  
U gi|24391031|gb|CA060788.1|CA060788 ssalrga508105 mixed\_tissue Salmo salar cDNA, mRNA sequence  
V gi|117851386|gb|EG924082.1|EG924082 EST\_ssal\_evf\_26830 ssalevf mixed\_tissue Salmo salar cDNA Salmo salar cDNA clone ssal\_evf\_535\_213\_rev 5', mRNA sequence  
W gi|24344561|gb|CA043641.1|CA043641 ssalplnb510023 gut Salmo salar cDNA, mRNA sequence  
X gi|117520517|gb|EG852245.1|EG852245 EST\_ssal\_eve\_13213 ssaleve thyroid Salmo salar cDNA Salmo salar cDNA clone ssal\_eve\_517\_368\_rev 5', mRNA sequence  
Y gi|117527955|gb|EG859596.1|EG859596 EST\_ssal\_eve\_52928 ssaleve thyroid Salmo salar cDNA Salmo salar cDNA clone ssal\_eve\_572\_041\_rev 5', mRNA sequence  
Z gi|117527803|gb|EG859444.1|EG859444 EST\_ssal\_eve\_52791 ssaleve thyroid Salmo salar cDNA Salmo salar cDNA clone ssal\_eve\_571\_348\_rev 5', mRNA sequence  
a gi|117475503|gb|EG807722.1|EG807722 EST\_ssal\_evd\_17866 ssalevd thymus Salmo salar cDNA Salmo salar cDNA clone ssal\_evd\_522\_337\_rev 5', mRNA sequence  
b gi|117857779|gb|EG930475.1|EG930475 EST\_ssal\_evf\_32585 ssalevf mixed\_tissue Salmo salar cDNA Salmo salar cDNA clone ssal\_evf\_543\_119\_rev 5', mRNA sequence  
c gi|117485491|gb|EG817708.1|EG817708 EST\_ssal\_evd\_19917 ssalevd thymus Salmo salar cDNA Salmo salar cDNA clone ssal\_evd\_525\_242\_fwd 3', mRNA sequence  
d gi|117485492|gb|EG817709.1|EG817709 EST\_ssal\_evd\_19918 ssalevd thymus Salmo salar cDNA Salmo salar cDNA clone ssal\_evd\_525\_242\_rev 5', mRNA sequence  
e gi|117488571|gb|EG820788.1|EG820788 EST\_ssal\_evd\_22690 ssalevd thymus Salmo salar cDNA Salmo salar cDNA clone ssal\_evd\_529\_146\_rev 5', mRNA sequence  
f gi|117518879|gb|EG850607.1|EG850607 EST\_ssal\_eve\_7939 ssaleve thyroid Salmo salar cDNA Salmo salar cDNA clone ssal\_eve\_509\_289\_rev 5', mRNA sequence  
g gi|117847892|gb|EG920588.1|EG920588 EST\_ssal\_evf\_59427 ssalevf mixed\_tissue Salmo salar cDNA Salmo salar cDNA clone ssal\_evf\_579\_327\_rev 5', mRNA sequence  
h gi|117847891|gb|EG920587.1|EG920587 EST\_ssal\_evf\_59426 ssalevf mixed\_tissue Salmo salar cDNA Salmo salar cDNA clone ssal\_evf\_579\_327\_fwd 3', mRNA sequence  
i gi|117502137|gb|EG833933.1|EG833933 EST\_ssal\_eve\_45832 ssaleve thyroid Salmo salar cDNA Salmo salar cDNA clone ssal\_eve\_562\_131\_rev 5', mRNA sequence  
j gi|117504191|gb|EG835950.1|EG835950 EST\_ssal\_eve\_47648 ssaleve thyroid Salmo salar cDNA Salmo salar cDNA clone ssal\_eve\_564\_303\_rev 5', mRNA sequence  
k gi|117467703|gb|EG799922.1|EG799922 EST\_ssal\_evd\_56713 ssalevd thymus Salmo salar cDNA Salmo salar cDNA clone ssal\_evd\_576\_187\_rev 5', mRNA sequence  
l gi|117499256|gb|EG831473.1|EG831473 EST\_ssal\_eve\_43619 ssaleve thyroid Salmo salar cDNA Salmo salar cDNA clone ssal\_eve\_559\_116\_rev 5', mRNA sequence  
m gi|117835103|gb|EG907799.1|EG907799 EST\_ssal\_evf\_10176 ssalevf mixed\_tissue Salmo salar cDNA Salmo salar cDNA clone ssal\_evf\_511\_374\_rev 5', mRNA sequence  
n gi|117867306|gb|EG940002.1|EG940002 EST\_ssal\_evf\_23571 ssalevf mixed\_tissue Salmo salar cDNA Salmo salar cDNA clone ssal\_evf\_531\_030\_rev 5', mRNA sequence  
o gi|117528940|gb|EG860581.1|EG860581 EST\_ssal\_eve\_53815 ssaleve thyroid Salmo salar cDNA Salmo salar cDNA clone ssal\_eve\_573\_125\_rev 5', mRNA sequence  
p gi|117512073|gb|EG843832.1|EG843832 EST\_ssal\_eve\_36742 ssaleve thyroid Salmo salar cDNA Salmo salar cDNA clone ssal\_eve\_550\_004\_rev 5', mRNA sequence  
q gi|117522478|gb|EG854205.1|EG854205 EST\_ssal\_eve\_14977 ssaleve thyroid Salmo salar cDNA Salmo salar cDNA clone ssal\_eve\_520\_131\_rev 5', mRNA sequence  
r gi|117513927|gb|EG845686.1|EG845686 EST\_ssal\_eve\_49059 ssaleve thyroid Salmo salar cDNA Salmo salar cDNA clone ssal\_eve\_566\_307\_rev 5', mRNA sequence  
s gi|85042414|gb|DW570592.1|DW570592 EST\_ssal\_rgb2\_35011 rgb2 Salmo salar cDNA clone ssal\_rgb2\_556\_250\_fwd 3', mRNA sequence  
t gi|117854259|gb|EG926955.1|EG926955 EST\_ssal\_evf\_29416 ssalevf mixed\_tissue Salmo salar cDNA Salmo salar cDNA clone ssal\_evf\_539\_006\_rev 5', mRNA sequence  
u gi|117529008|gb|EG860649.1|EG860649 EST\_ssal\_eve\_53876 ssaleve thyroid Salmo salar cDNA Salmo salar cDNA clone ssal\_eve\_573\_158\_rev 5', mRNA sequence  
v gi|85042085|gb|DW570263.1|DW570263 EST\_ssal\_rgb2\_34682 rgb2 Salmo salar cDNA clone ssal\_rgb2\_556\_053\_fwd 3', mRNA sequence  
w gi|117432167|gb|EG764391.1|EG764391 EST\_ssal\_sjb\_2273 ssalsjb mixed\_tissue Salmo salar cDNA Salmo salar cDNA clone ssal\_sjb\_008\_098\_rev 5', mRNA sequence  
x gi|117434878|gb|EG767101.1|EG767101 EST\_ssal\_evd\_1885 ssalevd thymus Salmo salar cDNA Salmo salar cDNA clone ssal\_evd\_501\_055\_rev 5', mRNA sequence  
y gi|117569110|gb|EG890086.1|EG890086 EST\_ssal\_evf\_47978 ssalevf mixed\_tissue Salmo salar cDNA Salmo salar cDNA clone ssal\_evf\_564\_055\_rev 5', mRNA sequence  
z gi|89869807|gb|DY725930.1|DY725930 EST\_ssal\_rgb2\_81669 ssalrgb2 mixed\_tissue Salmo salar cDNA Salmo salar cDNA clone ssal\_rgb2\_634\_031\_fwd 3', mRNA sequence  
A gi|117518481|gb|EG850240.1|EG850240 EST\_ssal\_eve\_7609 ssaleve thyroid Salmo salar cDNA Salmo salar cDNA clone ssal\_eve\_509\_120\_rev 5', mRNA sequence  
B gi|117492728|gb|EG824945.1|EG824945 EST\_ssal\_evd\_26431 ssalevd thymus Salmo salar cDNA Salmo salar cDNA clone ssal\_evd\_534\_171\_rev 5', mRNA sequence  
C gi|117512072|gb|EG843831.1|EG843831 EST\_ssal\_eve\_36741 ssaleve thyroid Salmo salar cDNA Salmo salar cDNA clone ssal\_eve\_550\_004\_fwd 3', mRNA sequence  
D gi|117458444|gb|EG790663.1|EG790663 EST\_ssal\_evd\_10916 ssalevd thymus Salmo salar cDNA Salmo salar cDNA clone ssal\_evd\_513\_162\_fwd 3', mRNA sequence  
E gi|89830588|gb|DY692848.1|DY692848 EST\_ssal\_plnb\_5758 ssalplnb mixed\_tissue Salmo salar cDNA Salmo salar cDNA clone ssal\_plnb\_027\_175\_fwd 3', mRNA sequence  
F gi|117488572|gb|EG820789.1|EG820789 EST\_ssal\_evd\_22691 ssalevd thymus Salmo salar cDNA Salmo salar cDNA clone ssal\_evd\_529\_146\_fwd 3', mRNA sequence  
G gi|117857780|gb|EG930476.1|EG930476 EST\_ssal\_evf\_32586 ssalevf mixed\_tissue Salmo salar cDNA Salmo salar cDNA clone ssal\_evf\_543\_119\_fwd 3', mRNA sequence  
H gi|85042084|gb|DW570262.1|DW570262 EST\_ssal\_rgb2\_34681 rgb2 Salmo salar cDNA clone ssal\_rgb2\_556\_053\_rev 5', mRNA sequence  
I gi|70786102|gb|DR695742.1|DR695742 SMT3-0045 Atlantic Salmon macrophage - Aeromonas in vitro infection Salmo salar cDNA clone SMT3-0045, mRNA sequence  
J gi|85163185|gb|DW592106.1|DW592106 smus1-014AG02.g1\_#0014.0 DIAS\_SMUS Salmo salar cDNA 5', mRNA sequence  
K gi|85153825|gb|DW588217.1|DW588217 sbra1-001BF07.y1\_#0525.2 DIAS\_SBRA Salmo salar cDNA 5', mRNA sequence  
L gi|89830587|gb|DY692847.1|DY692847 EST\_ssal\_plnb\_5757 ssalplnb mixed\_tissue Salmo salar cDNA Salmo salar cDNA clone ssal\_plnb\_027\_175\_rev 5', mRNA sequence  
M gi|117569109|gb|EG890085.1|EG890085 EST\_ssal\_evf\_47977 ssalevf mixed\_tissue Salmo salar cDNA Salmo salar cDNA clone ssal\_evf\_564\_055\_fwd 3', mRNA sequence  
N gi|117513939|gb|EG845698.1|EG845698 EST\_ssal\_eve\_49060 ssaleve thyroid Salmo salar cDNA Salmo salar cDNA clone ssal\_eve\_566\_307\_fwd 3', mRNA sequence  
O gi|85153543|gb|DW588116.1|DW588116 sbra1-001AE10.y1\_#0524.2 DIAS\_SBRA Salmo salar cDNA 5', mRNA sequence  
P gi|117527802|gb|EG859443.1|EG859443 EST\_ssal\_eve\_52790 ssaleve thyroid Salmo salar cDNA Salmo salar cDNA clone ssal\_eve\_571\_348\_fwd 3', mRNA sequence  
Q gi|85042415|gb|DW570593.1|DW570593 EST\_ssal\_rgb2\_35012 rgb2 Salmo salar cDNA clone ssal\_rgb2\_556\_250\_rev 5', mRNA sequence  
R gi|89869808|gb|DY725931.1|DY725931 EST\_ssal\_rgb2\_81670 ssalrgb2 mixed\_tissue Salmo salar cDNA Salmo salar cDNA clone ssal\_rgb2\_634\_031\_rev 5', mRNA sequence  
S gi|117520518|gb|EG852246.1|EG852246 EST\_ssal\_eve\_13214 ssaleve thyroid Salmo salar cDNA Salmo salar cDNA clone ssal\_eve\_517\_368\_fwd 3', mRNA sequence  
T gi|117867307|gb|EG940003.1|EG940003 EST\_ssal\_evf\_23572 ssalevf mixed\_tissue Salmo salar cDNA Salmo salar cDNA clone ssal\_evf\_531\_030\_fwd 3', mRNA sequence  
U gi|117461914|gb|EG794133.1|EG794133 EST\_ssal\_evd\_53907 ssalevd thymus Salmo salar cDNA Salmo salar cDNA clone ssal\_evd\_572\_251\_fwd 3', mRNA sequence  
V gi|117441760|gb|EG773983.1|EG773983 EST\_ssal\_evd\_49369 ssalevd thymus Salmo salar cDNA Salmo salar cDNA clone ssal\_evd\_566\_159\_fwd 3', mRNA sequence  
W gi|24350558|gb|CA045032.1|CA045032 ssalpla003029 gut Salmo salar cDNA, mRNA sequence  
X gi|29314093|gb|CB502867.1|CB502867 ssalplnb510023\_rev gut Salmo salar cDNA, mRNA sequence  
Y gi|117528939|gb|EG860580.1|EG860580 EST\_ssal\_eve\_53814 ssaleve thyroid Salmo salar cDNA Salmo salar cDNA clone ssal\_eve\_573\_125\_fwd 3', mRNA sequence  
Z gi|117522479|gb|EG854206.1|EG854206 EST\_ssal\_eve\_14978 ssaleve thyroid Salmo salar cDNA Salmo salar cDNA clone ssal\_eve\_520\_131\_fwd 3', mRNA sequence  
a gi|117529009|gb|EG860650.1|EG860650 EST\_ssal\_eve\_53877 ssaleve thyroid Salmo salar cDNA Salmo salar cDNA clone ssal\_eve\_573\_158\_fwd 3', mRNA sequence  
b gi|117502136|gb|EG833932.1|EG833932 EST\_ssal\_eve\_45831 ssaleve thyroid Salmo salar cDNA Salmo salar cDNA clone ssal\_eve\_562\_131\_fwd 3', mRNA sequence  
c gi|117527954|gb|EG859595.1|EG859595 EST\_ssal\_eve\_52927 ssaleve thyroid Salmo salar cDNA Salmo salar cDNA clone ssal\_eve\_572\_041\_fwd 3', mRNA sequence  
d gi|117499257|gb|EG831474.1|EG831474 EST\_ssal\_eve\_43620 ssaleve thyroid Salmo salar cDNA Salmo salar cDNA clone ssal\_eve\_559\_116\_fwd 3', mRNA sequence  
e gi|89874592|gb|DY730715.1|DY730715 EST\_ssal\_rgb2\_86454 ssalrgb2 mixed\_tissue Salmo salar cDNA Salmo salar cDNA clone ssal\_rgb2\_641\_164\_rev 5', mRNA sequence  
f gi|117434766|gb|EG766989.1|EG766989 EST\_ssal\_evd\_1884 ssalevd thymus Salmo salar cDNA Salmo salar cDNA clone ssal\_evd\_501\_055\_fwd 3', mRNA sequence  
g gi|117511544|gb|EG843303.1|EG843303 EST\_ssal\_eve\_36266 ssaleve thyroid Salmo salar cDNA Salmo salar cDNA clone ssal\_eve\_549\_123\_fwd 3', mRNA sequence  
h gi|117447243|gb|EG779462.1|EG779462 EST\_ssal\_evd\_36299 ssalevd thymus Salmo salar cDNA Salmo salar cDNA clone ssal\_evd\_548\_175\_fwd 3', mRNA sequence  
i gi|117835104|gb|EG907800.1|EG907800 EST\_ssal\_evf\_10177 ssalevf mixed\_tissue Salmo salar cDNA Salmo salar cDNA clone ssal\_evf\_511\_374\_fwd 3', mRNA sequence  
j gi|117851385|gb|EG924081.1|EG924081 EST\_ssal\_evf\_26829 ssalevf mixed\_tissue Salmo salar cDNA Salmo salar cDNA clone ssal\_evf\_535\_213\_fwd 3', mRNA sequence  
k gi|117432166|gb|EG764390.1|EG764390 EST\_ssal\_sjb\_2272 ssalsjb mixed\_tissue Salmo salar cDNA Salmo salar cDNA clone ssal\_sjb\_008\_098\_fwd 3', mRNA sequence  
l gi|45321728|gb|CK891995.1|CK891995 SGP151993 Atlantic salmon Skin cDNA library Salmo salar cDNA clone HU3-0265 5', mRNA sequence  
m gi|117549246|gb|EG880691.1|EG880691 EST\_ssal\_eve\_23982 ssaleve thyroid Salmo salar cDNA Salmo salar cDNA clone ssal\_eve\_532\_259\_fwd 3', mRNA sequence  
n gi|117518480|gb|EG850239.1|EG850239 EST\_ssal\_eve\_7608 ssaleve thyroid Salmo salar cDNA Salmo salar cDNA clone ssal\_eve\_509\_120\_fwd 3', mRNA sequence  
o gi|117455819|gb|EG788038.1|EG788038 EST\_ssal\_evd\_8554 ssalevd thymus Salmo salar cDNA Salmo salar cDNA clone ssal\_evd\_510\_067\_fwd 3', mRNA sequence  
p gi|117467705|gb|EG799924.1|EG799924 EST\_ssal\_evd\_56714 ssalevd thymus Salmo salar cDNA Salmo salar cDNA clone ssal\_evd\_576\_187\_fwd 3', mRNA sequence  
q gi|117439139|gb|EG771362.1|EG771362 EST\_ssal\_evd\_47010 ssalevd thymus Salmo salar cDNA Salmo salar cDNA clone ssal\_evd\_563\_065\_fwd 3', mRNA sequence  
r gi|117463454|gb|EG795673.1|EG795673 EST\_ssal\_evd\_15426 ssalevd thymus Salmo salar cDNA Salmo salar cDNA clone ssal\_evd\_519\_225\_fwd 3', mRNA sequence  
s gi|89855217|gb|DY711340.1|DY711340 EST\_ssal\_rgb2\_67079 ssalrgb2 mixed\_tissue Salmo salar cDNA Salmo salar cDNA clone ssal\_rgb2\_609\_014\_rev 5', mRNA sequence  
t gi|117475514|gb|EG807733.1|EG807733 EST\_ssal\_evd\_17867 ssalevd thymus Salmo salar cDNA Salmo salar cDNA clone ssal\_evd\_522\_337\_fwd 3', mRNA sequence  
u gi|117461038|gb|EG793257.1|EG793257 EST\_ssal\_evd\_13251 ssalevd thymus Salmo salar cDNA Salmo salar cDNA clone ssal\_evd\_516\_229\_fwd 3', mRNA sequence  
v gi|117504192|gb|EG835951.1|EG835951 EST\_ssal\_eve\_47649 ssaleve thyroid Salmo salar cDNA Salmo salar cDNA clone ssal\_eve\_564\_303\_fwd 3', mRNA sequence  
w gi|117446326|gb|EG778547.1|EG778547 EST\_ssal\_evd\_5075 ssalevd thymus Salmo salar cDNA Salmo salar cDNA clone ssal\_evd\_505\_169\_fwd 3', mRNA sequence  
x gi|117492729|gb|EG824946.1|EG824946 EST\_ssal\_evd\_26432 ssalevd thymus Salmo salar cDNA Salmo salar cDNA clone ssal\_evd\_534\_171\_fwd 3', mRNA sequence  
y gi|117527602|gb|EG859243.1|EG859243 EST\_ssal\_eve\_52610 ssaleve thyroid Salmo salar cDNA Salmo salar cDNA clone ssal\_eve\_571\_256\_fwd 3', mRNA sequence

9 SNPs detected

A B C D E F G H I J K L M N O P Q R S T U V W X Y Z a b c d e f g h i j k l m n o p q r s t u v w x y z A B C D E F G H I J K L M N O P Q R S T U V W X Y Z a b c d e f g h i j k l m n o p q r s t u v w x y  cosegregation weighted

837 . . . . . . . . . . . . . . . . . . . . . . . . . . . . . . . . . . . . . . . . . . . . . . . . . . . . . . . . - A A A A . . - . A . A A A - - - . . . - - - - A A A A . . A A A . A A A A A A A A A A A A .   3/9 11.65
838 . . . . . . . . . . . . . . . . . . . . . . . . . . . . . . . . . . . . . . . . . . . . . . . . . . . . . . . . - T T T T . . - . T . T T T - - - . . . - - - - T T T T . . T T T . T T T T T T T T T T T T .   3/9 11.65
839 . . . . . . . . . . . . . . . . . . . . . . . . . . . . . . . . . . . . . . . . . . . . . . . . . . . . . . . . - G G G G . . - . G . G G G - - - . . . - - - - G G G G . . G G G . G G G G G G G G G G G G .   3/9 11.65
840 . . . . . . . . . . . . . . . . . . . . . . . . . . . . . . . . . . . . . . . . . . . . . . . . . . . . . . . . - A A A - . . - . A . A A A - - - . . . - - - - A A A A . . A A A . A A A A A A A A A A A A .   3/9 11.65
841 . . . . . . . . . . . . . . . . . . . . . . . . . . . . . . . . . . . . . . . . . . . . . . . . . . . . . . . . - T T T - . . - . T . T T T - - - . . . - - - - T T T T . . T T T . T T T T T T T T T T T T .   3/9 11.65
842 . . . . . . . . . . . . . . . . . . . . . . . . . . . . . . . . . . . . . . . . . . . . . . . . . . . . . . . . - G G G - . . - . G . G G G - - - . . . - - - - G G G G . . G G G . G G G G G G G G G G G G .   3/9 11.65
1484 . . . . . . . . . . . . . . . . . . . . . . . . . . . . . . . . . . . . . . . . . . . . . . . . . . . . . . . . . . . . . . . T T T . . A A T T T T T T . T T T A . A - - - - - - - - - - - - - - - - - - - -   3/9 11.65
1485 . . . . . . . . . . . . . . . . . . . . . . . . . . . . . . . . . . . . . . . . . . . . . . . . . . . . . . . . . . . . . . . G G G . . T T G G G G G G . G G G T . T - - - - - - - - - - - - - - - - - - - -   3/9 11.65
1486 . . . . . . . . . . . . . . . . . . . . . . . . . . . . . . . . . . . . . . . . . . . . . . . . . . . . . . . . . . . . . . . T T T . . . . T T T T T T . T T T . . . - - - - - - - - - - - - - - - - - - - -   3/9 10.36
