# Supplementary material for: Discovery and application of insertion-deletion (INDEL) polymorphisms for QTL mapping of early life-history traits in Atlantic salmon
Source: BMC Genomics. 2010 Mar 8;11:156. doi: 10.1186/1471-2164-11-156 (PMC2838853; doi:10.1186/1471-2164-11-156)
Supplement: Additional file 2 — Information on developed 76 locus single-run INDEL panel in Atlantic salmon. Information on fluorescence labeling, primer concentrations, PCR pooling and links to alignments, INDEL motifs and GENESCAN (Burge and Karlin 1997) predictions of genes/exons are available in html format. [file 1471-2164-11-156-S2.ZIP › Additionalfile2/snpsummary11005.html]

```
Cluster 3975 Contig 1

prev  Summary    Contig List  next
```

Size of Consensus sequence = 1014

Number of sequences = 14

Minimum redundancy = 5

Key

A gi|117476787|gb|EG809006.1|EG809006 EST\_ssal\_evd\_29425 ssalevd thymus Salmo salar cDNA Salmo salar cDNA clone ssal\_evd\_538\_227\_rev 5', mRNA sequence  
B gi|117476786|gb|EG809005.1|EG809005 EST\_ssal\_evd\_29424 ssalevd thymus Salmo salar cDNA Salmo salar cDNA clone ssal\_evd\_538\_227\_fwd 3', mRNA sequence  
C gi|89874583|gb|DY730706.1|DY730706 EST\_ssal\_rgb2\_86445 ssalrgb2 mixed\_tissue Salmo salar cDNA Salmo salar cDNA clone ssal\_rgb2\_641\_158\_fwd 3', mRNA sequence  
D gi|85049237|gb|DW577415.1|DW577415 EST\_ssal\_rgb2\_41834 rgb2 Salmo salar cDNA clone ssal\_rgb2\_567\_313\_fwd 3', mRNA sequence  
E gi|117507402|gb|EG839161.1|EG839161 EST\_ssal\_eve\_2137 ssaleve thyroid Salmo salar cDNA Salmo salar cDNA clone ssal\_eve\_501\_368\_rev 5', mRNA sequence  
F gi|117846130|gb|EG918826.1|EG918826 EST\_ssal\_evf\_57841 ssalevf mixed\_tissue Salmo salar cDNA Salmo salar cDNA clone ssal\_evf\_577\_241\_rev 5', mRNA sequence  
G gi|117507403|gb|EG839162.1|EG839162 EST\_ssal\_eve\_2138 ssaleve thyroid Salmo salar cDNA Salmo salar cDNA clone ssal\_eve\_501\_368\_fwd 3', mRNA sequence  
H gi|117467121|gb|EG799340.1|EG799340 EST\_ssal\_evd\_56188 ssalevd thymus Salmo salar cDNA Salmo salar cDNA clone ssal\_evd\_575\_293\_fwd 3', mRNA sequence  
I gi|117467122|gb|EG799341.1|EG799341 EST\_ssal\_evd\_56189 ssalevd thymus Salmo salar cDNA Salmo salar cDNA clone ssal\_evd\_575\_293\_rev 5', mRNA sequence  
J gi|117831043|gb|EG903739.1|EG903739 EST\_ssal\_evf\_3038 ssalevf mixed\_tissue Salmo salar cDNA Salmo salar cDNA clone ssal\_evf\_502\_136\_fwd 3', mRNA sequence  
K gi|117831054|gb|EG903750.1|EG903750 EST\_ssal\_evf\_3039 ssalevf mixed\_tissue Salmo salar cDNA Salmo salar cDNA clone ssal\_evf\_502\_136\_rev 5', mRNA sequence  
L gi|117843580|gb|EG916276.1|EG916276 EST\_ssal\_evf\_52765 ssalevf mixed\_tissue Salmo salar cDNA Salmo salar cDNA clone ssal\_evf\_570\_258\_rev 5', mRNA sequence  
M gi|117837867|gb|EG910563.1|EG910563 EST\_ssal\_evf\_54051 ssalevf mixed\_tissue Salmo salar cDNA Salmo salar cDNA clone ssal\_evf\_572\_163\_rev 5', mRNA sequence  
N gi|117837878|gb|EG910574.1|EG910574 EST\_ssal\_evf\_54052 ssalevf mixed\_tissue Salmo salar cDNA Salmo salar cDNA clone ssal\_evf\_572\_163\_fwd 3', mRNA sequence

2 SNPs detected

A B C D E F G H I J K L M N  cosegregation weighted

335 - - - - - - - T T T T - T T   2/2 100.00
336 - - - - - - - T T T T - T T   2/2 100.00
