# Supplementary material for: Discovery and application of insertion-deletion (INDEL) polymorphisms for QTL mapping of early life-history traits in Atlantic salmon
Source: BMC Genomics. 2010 Mar 8;11:156. doi: 10.1186/1471-2164-11-156 (PMC2838853; doi:10.1186/1471-2164-11-156)
Supplement: Additional file 2 — Information on developed 76 locus single-run INDEL panel in Atlantic salmon. Information on fluorescence labeling, primer concentrations, PCR pooling and links to alignments, INDEL motifs and GENESCAN (Burge and Karlin 1997) predictions of genes/exons are available in html format. [file 1471-2164-11-156-S2.ZIP › Additionalfile2/snpsummary1126.html]

```
Cluster 144 Contig 5

prev  Summary    Contig List  next
```

Size of Consensus sequence = 1496

Number of sequences = 73

Minimum redundancy = 6

Key

A gi|29317980|gb|CB509269.1|CB509269 ssalcna007085 muscle\_skin Salmo salar cDNA, mRNA sequence  
B gi|117495509|gb|EG827726.1|EG827726 EST\_ssal\_eve\_40245 ssaleve thyroid Salmo salar cDNA Salmo salar cDNA clone ssal\_eve\_554\_293\_fwd 3', mRNA sequence  
C gi|117495510|gb|EG827727.1|EG827727 EST\_ssal\_eve\_40246 ssaleve thyroid Salmo salar cDNA Salmo salar cDNA clone ssal\_eve\_554\_293\_rev 5', mRNA sequence  
D gi|89858010|gb|DY714133.1|DY714133 EST\_ssal\_rgb2\_69873 ssalrgb2 mixed\_tissue Salmo salar cDNA Salmo salar cDNA clone ssal\_rgb2\_613\_113\_fwd 3', mRNA sequence  
E gi|117531864|gb|EG863309.1|EG863309 EST\_ssal\_eve\_48845 ssaleve thyroid Salmo salar cDNA Salmo salar cDNA clone ssal\_eve\_566\_193\_rev 5', mRNA sequence  
F gi|85020372|gb|DW549028.1|DW549028 EST\_ssal\_rgb2\_13447 rgb2 Salmo salar cDNA clone ssal\_rgb2\_522\_180\_fwd 3', mRNA sequence  
G gi|85051749|gb|DW579927.1|DW579927 EST\_ssal\_rgb2\_44346 rgb2 Salmo salar cDNA clone ssal\_rgb2\_571\_325\_fwd 3', mRNA sequence  
H gi|89858985|gb|DY715108.1|DY715108 EST\_ssal\_rgb2\_70848 ssalrgb2 mixed\_tissue Salmo salar cDNA Salmo salar cDNA clone ssal\_rgb2\_614\_319\_fwd 3', mRNA sequence  
I gi|89872045|gb|DY728168.1|DY728168 EST\_ssal\_rgb2\_83907 ssalrgb2 mixed\_tissue Salmo salar cDNA Salmo salar cDNA clone ssal\_rgb2\_637\_211\_fwd 3', mRNA sequence  
J gi|117439980|gb|EG772203.1|EG772203 EST\_ssal\_evd\_47767 ssalevd thymus Salmo salar cDNA Salmo salar cDNA clone ssal\_evd\_564\_079\_rev 5', mRNA sequence  
K gi|117494027|gb|EG826244.1|EG826244 EST\_ssal\_eve\_38912 ssaleve thyroid Salmo salar cDNA Salmo salar cDNA clone ssal\_eve\_552\_370\_rev 5', mRNA sequence  
L gi|117492580|gb|EG824797.1|EG824797 EST\_ssal\_evd\_26299 ssalevd thymus Salmo salar cDNA Salmo salar cDNA clone ssal\_evd\_534\_102\_rev 5', mRNA sequence  
M gi|84982975|gb|DW533325.1|DW533325 EST\_ssal\_plnb\_1961 plnb Salmo salar cDNA clone ssal\_plnb\_015\_042\_fwd 3', mRNA sequence  
N gi|57010566|gb|CX282056.1|CX282056 ssalkld006076\_0 kidney Salmo salar cDNA, mRNA sequence  
O gi|24342422|gb|CA041502.1|CA041502 ssalplnb507215 gut Salmo salar cDNA, mRNA sequence  
P gi|85023622|gb|DW552278.1|DW552278 EST\_ssal\_rgb2\_16697 rgb2 Salmo salar cDNA clone ssal\_rgb2\_527\_209\_fwd 3', mRNA sequence  
Q gi|24384814|gb|CA054571.1|CA054571 ssalrgb517376 mixed\_tissue Salmo salar cDNA, mRNA sequence  
R gi|89883083|gb|DY739206.1|DY739206 EST\_ssal\_rgb2\_94945 ssalrgb2 mixed\_tissue Salmo salar cDNA Salmo salar cDNA clone ssal\_rgb2\_655\_214\_fwd 3', mRNA sequence  
S gi|85044872|gb|DW573050.1|DW573050 EST\_ssal\_rgb2\_37469 rgb2 Salmo salar cDNA clone ssal\_rgb2\_561\_005\_fwd 3', mRNA sequence  
T gi|84982438|gb|DW532788.1|DW532788 EST\_ssal\_plnb\_1424 plnb Salmo salar cDNA clone ssal\_plnb\_011\_061\_rev 5', mRNA sequence  
U gi|24381229|gb|CA050986.1|CA050986 ssalrgb526259 mixed\_tissue Salmo salar cDNA, mRNA sequence  
V gi|85052901|gb|DW581079.1|DW581079 EST\_ssal\_rgb2\_45498 rgb2 Salmo salar cDNA clone ssal\_rgb2\_573\_235\_fwd 3', mRNA sequence  
W gi|25997275|gb|CA768020.1|CA768020 ssalplnb507368 gut Salmo salar cDNA, mRNA sequence  
X gi|117500600|gb|EG832636.1|EG832636 EST\_ssal\_eve\_44665 ssaleve thyroid Salmo salar cDNA Salmo salar cDNA clone ssal\_eve\_560\_281\_rev 5', mRNA sequence  
Y gi|117463913|gb|EG796132.1|EG796132 EST\_ssal\_evd\_15839 ssalevd thymus Salmo salar cDNA Salmo salar cDNA clone ssal\_evd\_520\_054\_rev 5', mRNA sequence  
Z gi|117506930|gb|EG838689.1|EG838689 EST\_ssal\_eve\_1712 ssaleve thyroid Salmo salar cDNA Salmo salar cDNA clone ssal\_eve\_501\_150\_rev 5', mRNA sequence  
a gi|24342413|gb|CA041493.1|CA041493 ssalplnb507202 gut Salmo salar cDNA, mRNA sequence  
b gi|117548855|gb|EG880300.1|EG880300 EST\_ssal\_eve\_23630 ssaleve thyroid Salmo salar cDNA Salmo salar cDNA clone ssal\_eve\_532\_078\_rev 5', mRNA sequence  
c gi|24336869|gb|CA037534.1|CA037534 ssallna003090 liver Salmo salar cDNA, mRNA sequence  
d gi|26000029|gb|CA770774.1|CA770774 ssalnwh006014 whole Salmo salar cDNA, mRNA sequence  
e gi|117424094|gb|EG756318.1|EG756318 EST\_ssal\_sjb\_5089 ssalsjb mixed\_tissue Salmo salar cDNA Salmo salar cDNA clone ssal\_sjb\_012\_177\_rev 5', mRNA sequence  
f gi|117437659|gb|EG769882.1|EG769882 EST\_ssal\_evd\_45677 ssalevd thymus Salmo salar cDNA Salmo salar cDNA clone ssal\_evd\_561\_132\_rev 5', mRNA sequence  
g gi|117535965|gb|EG867410.1|EG867410 EST\_ssal\_eve\_28861 ssaleve thyroid Salmo salar cDNA Salmo salar cDNA clone ssal\_eve\_539\_081\_rev 5', mRNA sequence  
h gi|85052369|gb|DW580547.1|DW580547 EST\_ssal\_rgb2\_44966 rgb2 Salmo salar cDNA clone ssal\_rgb2\_572\_308\_fwd 3', mRNA sequence  
i gi|117858984|gb|EG931680.1|EG931680 EST\_ssal\_evf\_33670 ssalevf mixed\_tissue Salmo salar cDNA Salmo salar cDNA clone ssal\_evf\_544\_297\_rev 5', mRNA sequence  
j gi|117424095|gb|EG756319.1|EG756319 EST\_ssal\_sjb\_5090 ssalsjb mixed\_tissue Salmo salar cDNA Salmo salar cDNA clone ssal\_sjb\_012\_177\_fwd 3', mRNA sequence  
k gi|117439604|gb|EG771827.1|EG771827 EST\_ssal\_evd\_47428 ssalevd thymus Salmo salar cDNA Salmo salar cDNA clone ssal\_evd\_563\_283\_rev 5', mRNA sequence  
l gi|117494800|gb|EG827017.1|EG827017 EST\_ssal\_eve\_39608 ssaleve thyroid Salmo salar cDNA Salmo salar cDNA clone ssal\_eve\_553\_344\_rev 5', mRNA sequence  
m gi|117437658|gb|EG769881.1|EG769881 EST\_ssal\_evd\_45676 ssalevd thymus Salmo salar cDNA Salmo salar cDNA clone ssal\_evd\_561\_132\_fwd 3', mRNA sequence  
n gi|117858985|gb|EG931681.1|EG931681 EST\_ssal\_evf\_33671 ssalevf mixed\_tissue Salmo salar cDNA Salmo salar cDNA clone ssal\_evf\_544\_297\_fwd 3', mRNA sequence  
o gi|117859198|gb|EG931894.1|EG931894 EST\_ssal\_evf\_33862 ssalevf mixed\_tissue Salmo salar cDNA Salmo salar cDNA clone ssal\_evf\_545\_012\_rev 5', mRNA sequence  
p gi|117545264|gb|EG876709.1|EG876709 EST\_ssal\_eve\_20399 ssaleve thyroid Salmo salar cDNA Salmo salar cDNA clone ssal\_eve\_527\_283\_rev 5', mRNA sequence  
q gi|117492579|gb|EG824796.1|EG824796 EST\_ssal\_evd\_26298 ssalevd thymus Salmo salar cDNA Salmo salar cDNA clone ssal\_evd\_534\_102\_fwd 3', mRNA sequence  
r gi|70786756|gb|DR696396.1|DR696396 SMV6-0015 Atlantic Salmon macrophage - Aeromonas in vivo infection Salmo salar cDNA clone SMV6-0015, mRNA sequence  
s gi|117456570|gb|EG788789.1|EG788789 EST\_ssal\_evd\_53426 ssalevd thymus Salmo salar cDNA Salmo salar cDNA clone ssal\_evd\_572\_002\_fwd 3', mRNA sequence  
t gi|76595465|gb|DV107136.1|DV107136 SGP283351 Atlantic salmon gills SSH cDNA library Salmo salar cDNA clone IGO-0383, mRNA sequence  
u gi|117535966|gb|EG867411.1|EG867411 EST\_ssal\_eve\_28862 ssaleve thyroid Salmo salar cDNA Salmo salar cDNA clone ssal\_eve\_539\_081\_fwd 3', mRNA sequence  
v gi|85020371|gb|DW549027.1|DW549027 EST\_ssal\_rgb2\_13446 rgb2 Salmo salar cDNA clone ssal\_rgb2\_522\_180\_rev 5', mRNA sequence  
w gi|84972501|gb|DW470902.1|DW470902 SGP313534 Atlantic salmon Testis cDNA library Salmo salar cDNA clone MG4-4309 5', mRNA sequence  
x gi|45312717|gb|CK883086.1|CK883086 SGP147799 Atlantic salmon Heart cDNA library Salmo salar cDNA clone H6-0822 5', mRNA sequence  
y gi|117531875|gb|EG863320.1|EG863320 EST\_ssal\_eve\_48846 ssaleve thyroid Salmo salar cDNA Salmo salar cDNA clone ssal\_eve\_566\_193\_fwd 3', mRNA sequence  
z gi|89883082|gb|DY739205.1|DY739205 EST\_ssal\_rgb2\_94944 ssalrgb2 mixed\_tissue Salmo salar cDNA Salmo salar cDNA clone ssal\_rgb2\_655\_214\_rev 5', mRNA sequence  
A gi|89858984|gb|DY715107.1|DY715107 EST\_ssal\_rgb2\_70847 ssalrgb2 mixed\_tissue Salmo salar cDNA Salmo salar cDNA clone ssal\_rgb2\_614\_319\_rev 5', mRNA sequence  
B gi|85052902|gb|DW581080.1|DW581080 EST\_ssal\_rgb2\_45499 rgb2 Salmo salar cDNA clone ssal\_rgb2\_573\_235\_rev 5', mRNA sequence  
C gi|89852451|gb|DY708574.1|DY708574 EST\_ssal\_rgb2\_64313 ssalrgb2 mixed\_tissue Salmo salar cDNA Salmo salar cDNA clone ssal\_rgb2\_603\_304\_rev 5', mRNA sequence  
D gi|117494028|gb|EG826245.1|EG826245 EST\_ssal\_eve\_38913 ssaleve thyroid Salmo salar cDNA Salmo salar cDNA clone ssal\_eve\_552\_370\_fwd 3', mRNA sequence  
E gi|89858009|gb|DY714132.1|DY714132 EST\_ssal\_rgb2\_69872 ssalrgb2 mixed\_tissue Salmo salar cDNA Salmo salar cDNA clone ssal\_rgb2\_613\_113\_rev 5', mRNA sequence  
F gi|117463915|gb|EG796134.1|EG796134 EST\_ssal\_evd\_15840 ssalevd thymus Salmo salar cDNA Salmo salar cDNA clone ssal\_evd\_520\_054\_fwd 3', mRNA sequence  
G gi|70785732|gb|DR695372.1|DR695372 SM1-0037 Atlantic Salmon macrophage Salmo salar cDNA clone SM1-0037, mRNA sequence  
H gi|45314742|gb|CK885111.1|CK885111 SGP165477 Atlantic salmon Intestine cDNA library Salmo salar cDNA clone T4-0180 5', mRNA sequence  
I gi|57121795|gb|CX353236.1|CX353236 ssalrgb526259\_rev\_0 mixed\_tissue Salmo salar cDNA, mRNA sequence  
J gi|84982439|gb|DW532789.1|DW532789 EST\_ssal\_plnb\_1425 plnb Salmo salar cDNA clone ssal\_plnb\_011\_061\_fwd 3', mRNA sequence  
K gi|84982976|gb|DW533326.1|DW533326 EST\_ssal\_plnb\_1962 plnb Salmo salar cDNA clone ssal\_plnb\_015\_042\_rev 5', mRNA sequence  
L gi|117494801|gb|EG827018.1|EG827018 EST\_ssal\_eve\_39609 ssaleve thyroid Salmo salar cDNA Salmo salar cDNA clone ssal\_eve\_553\_344\_fwd 3', mRNA sequence  
M gi|117859199|gb|EG931895.1|EG931895 EST\_ssal\_evf\_33863 ssalevf mixed\_tissue Salmo salar cDNA Salmo salar cDNA clone ssal\_evf\_545\_012\_fwd 3', mRNA sequence  
N gi|117506929|gb|EG838688.1|EG838688 EST\_ssal\_eve\_1711 ssaleve thyroid Salmo salar cDNA Salmo salar cDNA clone ssal\_eve\_501\_150\_fwd 3', mRNA sequence  
O gi|117497766|gb|EG829983.1|EG829983 EST\_ssal\_eve\_42277 ssaleve thyroid Salmo salar cDNA Salmo salar cDNA clone ssal\_eve\_557\_193\_fwd 3', mRNA sequence  
P gi|117545263|gb|EG876708.1|EG876708 EST\_ssal\_eve\_20398 ssaleve thyroid Salmo salar cDNA Salmo salar cDNA clone ssal\_eve\_527\_283\_fwd 3', mRNA sequence  
Q gi|85051750|gb|DW579928.1|DW579928 EST\_ssal\_rgb2\_44347 rgb2 Salmo salar cDNA clone ssal\_rgb2\_571\_325\_rev 5', mRNA sequence  
R gi|117548856|gb|EG880301.1|EG880301 EST\_ssal\_eve\_23631 ssaleve thyroid Salmo salar cDNA Salmo salar cDNA clone ssal\_eve\_532\_078\_fwd 3', mRNA sequence  
S gi|89872044|gb|DY728167.1|DY728167 EST\_ssal\_rgb2\_83906 ssalrgb2 mixed\_tissue Salmo salar cDNA Salmo salar cDNA clone ssal\_rgb2\_637\_211\_rev 5', mRNA sequence  
T gi|117500601|gb|EG832637.1|EG832637 EST\_ssal\_eve\_44666 ssaleve thyroid Salmo salar cDNA Salmo salar cDNA clone ssal\_eve\_560\_281\_fwd 3', mRNA sequence  
U gi|84208057|gb|DW179848.1|DW179848 SGP303447 Atlantic salmon Gills II cDNA library Salmo salar cDNA clone SS0075B08 5', mRNA sequence

3 SNPs detected

A B C D E F G H I J K L M N O P Q R S T U V W X Y Z a b c d e f g h i j k l m n o p q r s t u v w x y z A B C D E F G H I J K L M N O P Q R S T U  cosegregation weighted

420 . . . A T A T A T T T A T T T A A A A T T T T T T T T T T T A T T A T A T T T T T T A . T . . . . . . . . . . . . . . . . . . . . . . . . . . . .   1/3 18.72
752 . . . . . . . . . . . . . . . . . . . . . . . . . . . . . . - . . . . - - - - - - - A A - - - A . . . A A - A . A - . . . . . - - - - - - - - - .   2/3 26.48
753 . . . . . . . . . . . . . . . . . . . . . . . . . . . . . . - . . . . - - - - - - - A A - - - A . . . A A - A . A - . . . . . - - - - - - - - - .   2/3 26.48
