# Supplementary material for: Discovery and application of insertion-deletion (INDEL) polymorphisms for QTL mapping of early life-history traits in Atlantic salmon
Source: BMC Genomics. 2010 Mar 8;11:156. doi: 10.1186/1471-2164-11-156 (PMC2838853; doi:10.1186/1471-2164-11-156)
Supplement: Additional file 2 — Information on developed 76 locus single-run INDEL panel in Atlantic salmon. Information on fluorescence labeling, primer concentrations, PCR pooling and links to alignments, INDEL motifs and GENESCAN (Burge and Karlin 1997) predictions of genes/exons are available in html format. [file 1471-2164-11-156-S2.ZIP › Additionalfile2/snpsummary1160.html]

```
Cluster 153 Contig 1

prev  Summary    Contig List  next
```

Size of Consensus sequence = 1785

Number of sequences = 118

Minimum redundancy = 6

Key

A gi|85038393|gb|DW566571.1|DW566571 EST\_ssal\_rgb2\_30990 rgb2 Salmo salar cDNA clone ssal\_rgb2\_550\_080\_fwd 3', mRNA sequence  
B gi|117501003|gb|EG833021.1|EG833021 EST\_ssal\_eve\_45012 ssaleve thyroid Salmo salar cDNA Salmo salar cDNA clone ssal\_eve\_561\_080\_rev 5', mRNA sequence  
C gi|117437562|gb|EG769785.1|EG769785 EST\_ssal\_evd\_45590 ssalevd thymus Salmo salar cDNA Salmo salar cDNA clone ssal\_evd\_561\_087\_rev 5', mRNA sequence  
D gi|117437563|gb|EG769786.1|EG769786 EST\_ssal\_evd\_45591 ssalevd thymus Salmo salar cDNA Salmo salar cDNA clone ssal\_evd\_561\_087\_fwd 3', mRNA sequence  
E gi|117465844|gb|EG798063.1|EG798063 EST\_ssal\_evd\_55039 ssalevd thymus Salmo salar cDNA Salmo salar cDNA clone ssal\_evd\_574\_072\_rev 5', mRNA sequence  
F gi|117483319|gb|EG815536.1|EG815536 EST\_ssal\_evd\_35303 ssalevd thymus Salmo salar cDNA Salmo salar cDNA clone ssal\_evd\_547\_029\_rev 5', mRNA sequence  
G gi|117849025|gb|EG921721.1|EG921721 EST\_ssal\_evf\_906 ssalevf mixed\_tissue Salmo salar cDNA Salmo salar cDNA clone ssal\_evf\_005\_123\_rev 5', mRNA sequence  
H gi|117466881|gb|EG799100.1|EG799100 EST\_ssal\_evd\_52212 ssalevd thymus Salmo salar cDNA Salmo salar cDNA clone ssal\_evd\_570\_141\_rev 5', mRNA sequence  
I gi|89854875|gb|DY710998.1|DY710998 EST\_ssal\_rgb2\_66737 ssalrgb2 mixed\_tissue Salmo salar cDNA Salmo salar cDNA clone ssal\_rgb2\_608\_201\_fwd 3', mRNA sequence  
J gi|24346753|gb|CA044470.1|CA044470 ssalela002074 gut Salmo salar cDNA, mRNA sequence  
K gi|24393465|gb|CA063222.1|CA063222 ssalrgb509381 mixed\_tissue Salmo salar cDNA, mRNA sequence  
L gi|117533550|gb|EG864995.1|EG864995 EST\_ssal\_eve\_57788 ssaleve thyroid Salmo salar cDNA Salmo salar cDNA clone ssal\_eve\_578\_297\_rev 5', mRNA sequence  
M gi|24386980|gb|CA056737.1|CA056737 ssalrgb536297 mixed\_tissue Salmo salar cDNA, mRNA sequence  
N gi|117469301|gb|EG801520.1|EG801520 EST\_ssal\_evd\_58152 ssalevd thymus Salmo salar cDNA Salmo salar cDNA clone ssal\_evd\_578\_182\_rev 5', mRNA sequence  
O gi|117469302|gb|EG801521.1|EG801521 EST\_ssal\_evd\_58153 ssalevd thymus Salmo salar cDNA Salmo salar cDNA clone ssal\_evd\_578\_182\_fwd 3', mRNA sequence  
P gi|117569172|gb|EG890148.1|EG890148 EST\_ssal\_evf\_48035 ssalevf mixed\_tissue Salmo salar cDNA Salmo salar cDNA clone ssal\_evf\_564\_086\_rev 5', mRNA sequence  
Q gi|117462049|gb|EG794268.1|EG794268 EST\_ssal\_evd\_14161 ssalevd thymus Salmo salar cDNA Salmo salar cDNA clone ssal\_evd\_517\_317\_rev 5', mRNA sequence  
R gi|117849147|gb|EG921843.1|EG921843 EST\_ssal\_evf\_53266 ssalevf mixed\_tissue Salmo salar cDNA Salmo salar cDNA clone ssal\_evf\_571\_138\_rev 5', mRNA sequence  
S gi|117462734|gb|EG794953.1|EG794953 EST\_ssal\_evd\_14778 ssalevd thymus Salmo salar cDNA Salmo salar cDNA clone ssal\_evd\_518\_257\_rev 5', mRNA sequence  
T gi|117441334|gb|EG773557.1|EG773557 EST\_ssal\_evd\_48985 ssalevd thymus Salmo salar cDNA Salmo salar cDNA clone ssal\_evd\_565\_335\_rev 5', mRNA sequence  
U gi|117494682|gb|EG826899.1|EG826899 EST\_ssal\_eve\_39501 ssaleve thyroid Salmo salar cDNA Salmo salar cDNA clone ssal\_eve\_553\_290\_rev 5', mRNA sequence  
V gi|117821265|gb|EG893961.1|EG893961 EST\_ssal\_evf\_51466 ssalevf mixed\_tissue Salmo salar cDNA Salmo salar cDNA clone ssal\_evf\_568\_340\_rev 5', mRNA sequence  
W gi|117445689|gb|EG777912.1|EG777912 EST\_ssal\_evd\_4504 ssalevd thymus Salmo salar cDNA Salmo salar cDNA clone ssal\_evd\_504\_257\_rev 5', mRNA sequence  
X gi|117524644|gb|EG856371.1|EG856371 EST\_ssal\_eve\_50023 ssaleve thyroid Salmo salar cDNA Salmo salar cDNA clone ssal\_eve\_568\_040\_rev 5', mRNA sequence  
Y gi|117522419|gb|EG854146.1|EG854146 EST\_ssal\_eve\_14923 ssaleve thyroid Salmo salar cDNA Salmo salar cDNA clone ssal\_eve\_520\_102\_rev 5', mRNA sequence  
Z gi|117466992|gb|EG799211.1|EG799211 EST\_ssal\_evd\_52213 ssalevd thymus Salmo salar cDNA Salmo salar cDNA clone ssal\_evd\_570\_141\_fwd 3', mRNA sequence  
a gi|117569578|gb|EG890554.1|EG890554 EST\_ssal\_evf\_48400 ssalevf mixed\_tissue Salmo salar cDNA Salmo salar cDNA clone ssal\_evf\_564\_281\_rev 5', mRNA sequence  
b gi|117502080|gb|EG833876.1|EG833876 EST\_ssal\_eve\_45781 ssaleve thyroid Salmo salar cDNA Salmo salar cDNA clone ssal\_eve\_562\_106\_rev 5', mRNA sequence  
c gi|117569173|gb|EG890149.1|EG890149 EST\_ssal\_evf\_48036 ssalevf mixed\_tissue Salmo salar cDNA Salmo salar cDNA clone ssal\_evf\_564\_086\_fwd 3', mRNA sequence  
d gi|117518909|gb|EG850637.1|EG850637 EST\_ssal\_eve\_7966 ssaleve thyroid Salmo salar cDNA Salmo salar cDNA clone ssal\_eve\_509\_303\_rev 5', mRNA sequence  
e gi|24380840|gb|CA050597.1|CA050597 ssalrgb526052 mixed\_tissue Salmo salar cDNA, mRNA sequence  
f gi|117462048|gb|EG794267.1|EG794267 EST\_ssal\_evd\_14160 ssalevd thymus Salmo salar cDNA Salmo salar cDNA clone ssal\_evd\_517\_317\_fwd 3', mRNA sequence  
g gi|117445402|gb|EG777625.1|EG777625 EST\_ssal\_evd\_4245 ssalevd thymus Salmo salar cDNA Salmo salar cDNA clone ssal\_evd\_504\_124\_rev 5', mRNA sequence  
h gi|29323875|gb|CB512649.1|CB512649 ssalrgb551313 mixed\_tissue Salmo salar cDNA, mRNA sequence  
i gi|117524643|gb|EG856370.1|EG856370 EST\_ssal\_eve\_50022 ssaleve thyroid Salmo salar cDNA Salmo salar cDNA clone ssal\_eve\_568\_040\_fwd 3', mRNA sequence  
j gi|117530801|gb|EG862248.1|EG862248 EST\_ssal\_eve\_55315 ssaleve thyroid Salmo salar cDNA Salmo salar cDNA clone ssal\_eve\_575\_150\_fwd 3', mRNA sequence  
k gi|117840942|gb|EG913638.1|EG913638 EST\_ssal\_evf\_15432 ssalevf mixed\_tissue Salmo salar cDNA Salmo salar cDNA clone ssal\_evf\_519\_060\_rev 5', mRNA sequence  
l gi|117441332|gb|EG773555.1|EG773555 EST\_ssal\_evd\_48984 ssalevd thymus Salmo salar cDNA Salmo salar cDNA clone ssal\_evd\_565\_335\_fwd 3', mRNA sequence  
m gi|117512715|gb|EG844474.1|EG844474 EST\_ssal\_eve\_9114 ssaleve thyroid Salmo salar cDNA Salmo salar cDNA clone ssal\_eve\_512\_128\_rev 5', mRNA sequence  
n gi|117502081|gb|EG833877.1|EG833877 EST\_ssal\_eve\_45782 ssaleve thyroid Salmo salar cDNA Salmo salar cDNA clone ssal\_eve\_562\_106\_fwd 3', mRNA sequence  
o gi|24391442|gb|CA061199.1|CA061199 ssalrga509063 mixed\_tissue Salmo salar cDNA, mRNA sequence  
p gi|117443428|gb|EG775651.1|EG775651 EST\_ssal\_evd\_50871 ssalevd thymus Salmo salar cDNA Salmo salar cDNA clone ssal\_evd\_568\_199\_rev 5', mRNA sequence  
q gi|117443429|gb|EG775652.1|EG775652 EST\_ssal\_evd\_50872 ssalevd thymus Salmo salar cDNA Salmo salar cDNA clone ssal\_evd\_568\_199\_fwd 3', mRNA sequence  
r gi|117459922|gb|EG792141.1|EG792141 EST\_ssal\_evd\_12247 ssalevd thymus Salmo salar cDNA Salmo salar cDNA clone ssal\_evd\_515\_092\_fwd 3', mRNA sequence  
s gi|117504477|gb|EG836236.1|EG836236 EST\_ssal\_eve\_47906 ssaleve thyroid Salmo salar cDNA Salmo salar cDNA clone ssal\_eve\_565\_063\_rev 5', mRNA sequence  
t gi|24384957|gb|CA054714.1|CA054714 ssalrgb511028 mixed\_tissue Salmo salar cDNA, mRNA sequence  
u gi|117437232|gb|EG769455.1|EG769455 EST\_ssal\_evd\_45293 ssalevd thymus Salmo salar cDNA Salmo salar cDNA clone ssal\_evd\_560\_310\_rev 5', mRNA sequence  
v gi|85028162|gb|DW556818.1|DW556818 EST\_ssal\_rgb2\_21237 rgb2 Salmo salar cDNA clone ssal\_rgb2\_534\_231\_fwd 3', mRNA sequence  
w gi|117445691|gb|EG777914.1|EG777914 EST\_ssal\_evd\_4505 ssalevd thymus Salmo salar cDNA Salmo salar cDNA clone ssal\_evd\_504\_257\_fwd 3', mRNA sequence  
x gi|117437879|gb|EG770102.1|EG770102 EST\_ssal\_evd\_45875 ssalevd thymus Salmo salar cDNA Salmo salar cDNA clone ssal\_evd\_561\_235\_rev 5', mRNA sequence  
y gi|117467691|gb|EG799910.1|EG799910 EST\_ssal\_evd\_56702 ssalevd thymus Salmo salar cDNA Salmo salar cDNA clone ssal\_evd\_576\_181\_rev 5', mRNA sequence  
z gi|117522561|gb|EG854288.1|EG854288 EST\_ssal\_eve\_15051 ssaleve thyroid Salmo salar cDNA Salmo salar cDNA clone ssal\_eve\_520\_170\_rev 5', mRNA sequence  
A gi|117867765|gb|EG940461.1|EG940461 EST\_ssal\_evf\_23984 ssalevf mixed\_tissue Salmo salar cDNA Salmo salar cDNA clone ssal\_evf\_531\_275\_fwd 3', mRNA sequence  
B gi|117541226|gb|EG872671.1|EG872671 EST\_ssal\_eve\_33597 ssaleve thyroid Salmo salar cDNA Salmo salar cDNA clone ssal\_eve\_545\_232\_rev 5', mRNA sequence  
C gi|89829776|gb|DY692343.1|DY692343 EST\_ssal\_plnb\_5253 ssalplnb mixed\_tissue Salmo salar cDNA Salmo salar cDNA clone ssal\_plnb\_025\_273\_fwd 3', mRNA sequence  
D gi|24387892|gb|CA057649.1|CA057649 ssalrgb507057 mixed\_tissue Salmo salar cDNA, mRNA sequence  
E gi|117488125|gb|EG820342.1|EG820342 EST\_ssal\_evd\_22289 ssalevd thymus Salmo salar cDNA Salmo salar cDNA clone ssal\_evd\_528\_320\_rev 5', mRNA sequence  
F gi|117836670|gb|EG909366.1|EG909366 EST\_ssal\_evf\_11587 ssalevf mixed\_tissue Salmo salar cDNA Salmo salar cDNA clone ssal\_evf\_513\_361\_fwd 3', mRNA sequence  
G gi|117522420|gb|EG854147.1|EG854147 EST\_ssal\_eve\_14924 ssaleve thyroid Salmo salar cDNA Salmo salar cDNA clone ssal\_eve\_520\_102\_fwd 3', mRNA sequence  
H gi|29314740|gb|CB503514.1|CB503514 ssalmge505319 gut Salmo salar cDNA, mRNA sequence  
I gi|117526954|gb|EG858681.1|EG858681 EST\_ssal\_eve\_52104 ssaleve thyroid Salmo salar cDNA Salmo salar cDNA clone ssal\_eve\_570\_372\_rev 5', mRNA sequence  
J gi|117486746|gb|EG818963.1|EG818963 EST\_ssal\_evd\_21047 ssalevd thymus Salmo salar cDNA Salmo salar cDNA clone ssal\_evd\_527\_067\_rev 5', mRNA sequence  
K gi|117469856|gb|EG802075.1|EG802075 EST\_ssal\_evd\_58651 ssalevd thymus Salmo salar cDNA Salmo salar cDNA clone ssal\_evd\_579\_063\_rev 5', mRNA sequence  
L gi|45319147|gb|CK889414.1|CK889414 SGP161434 Atlantic salmon Liver cDNA library Salmo salar cDNA clone L4-1108 5', mRNA sequence  
M gi|45319175|gb|CK889442.1|CK889442 SGP161465 Atlantic salmon Liver cDNA library Salmo salar cDNA clone L4-1140 5', mRNA sequence  
N gi|24381804|gb|CA051561.1|CA051561 ssalrgb531243 mixed\_tissue Salmo salar cDNA, mRNA sequence  
O gi|117821730|gb|EG894426.1|EG894426 EST\_ssal\_evf\_51885 ssalevf mixed\_tissue Salmo salar cDNA Salmo salar cDNA clone ssal\_evf\_569\_176\_rev 5', mRNA sequence  
P gi|117462733|gb|EG794952.1|EG794952 EST\_ssal\_evd\_14777 ssalevd thymus Salmo salar cDNA Salmo salar cDNA clone ssal\_evd\_518\_257\_fwd 3', mRNA sequence  
Q gi|117451414|gb|EG783633.1|EG783633 EST\_ssal\_evd\_2563 ssalevd thymus Salmo salar cDNA Salmo salar cDNA clone ssal\_evd\_502\_017\_rev 5', mRNA sequence  
R gi|89874758|gb|DY730881.1|DY730881 EST\_ssal\_rgb2\_86620 ssalrgb2 mixed\_tissue Salmo salar cDNA Salmo salar cDNA clone ssal\_rgb2\_641\_269\_rev 5', mRNA sequence  
S gi|117437231|gb|EG769454.1|EG769454 EST\_ssal\_evd\_45292 ssalevd thymus Salmo salar cDNA Salmo salar cDNA clone ssal\_evd\_560\_310\_fwd 3', mRNA sequence  
T gi|117821264|gb|EG893960.1|EG893960 EST\_ssal\_evf\_51465 ssalevf mixed\_tissue Salmo salar cDNA Salmo salar cDNA clone ssal\_evf\_568\_340\_fwd 3', mRNA sequence  
U gi|117442280|gb|EG774503.1|EG774503 EST\_ssal\_evd\_49837 ssalevd thymus Salmo salar cDNA Salmo salar cDNA clone ssal\_evd\_567\_027\_fwd 3', mRNA sequence  
V gi|117298016|gb|EG647829.1|EG647829 SGP259399 Atlantic salmon normalized Brain cDNA library Salmo salar cDNA clone NB1-1132 5', mRNA sequence  
W gi|117445400|gb|EG777623.1|EG777623 EST\_ssal\_evd\_4244 ssalevd thymus Salmo salar cDNA Salmo salar cDNA clone ssal\_evd\_504\_124\_fwd 3', mRNA sequence  
X gi|117472378|gb|EG804597.1|EG804597 EST\_ssal\_evd\_1657 ssalevd thymus Salmo salar cDNA Salmo salar cDNA clone ssal\_evd\_006\_308\_fwd 3', mRNA sequence  
Y gi|85048005|gb|DW576183.1|DW576183 EST\_ssal\_rgb2\_40602 rgb2 Salmo salar cDNA clone ssal\_rgb2\_565\_364\_rev 5', mRNA sequence  
Z gi|117526953|gb|EG858680.1|EG858680 EST\_ssal\_eve\_52103 ssaleve thyroid Salmo salar cDNA Salmo salar cDNA clone ssal\_eve\_570\_372\_fwd 3', mRNA sequence  
a gi|45307325|gb|CK877694.1|CK877694 SGP139027 Atlantic salmon Gills cDNA library Salmo salar cDNA clone G6-1064 5', mRNA sequence  
b gi|85028161|gb|DW556817.1|DW556817 EST\_ssal\_rgb2\_21236 rgb2 Salmo salar cDNA clone ssal\_rgb2\_534\_231\_rev 5', mRNA sequence  
c gi|117469857|gb|EG802076.1|EG802076 EST\_ssal\_evd\_58652 ssalevd thymus Salmo salar cDNA Salmo salar cDNA clone ssal\_evd\_579\_063\_fwd 3', mRNA sequence  
d gi|117483320|gb|EG815537.1|EG815537 EST\_ssal\_evd\_35304 ssalevd thymus Salmo salar cDNA Salmo salar cDNA clone ssal\_evd\_547\_029\_fwd 3', mRNA sequence  
e gi|117840941|gb|EG913637.1|EG913637 EST\_ssal\_evf\_15431 ssalevf mixed\_tissue Salmo salar cDNA Salmo salar cDNA clone ssal\_evf\_519\_060\_fwd 3', mRNA sequence  
f gi|117512826|gb|EG844585.1|EG844585 EST\_ssal\_eve\_9115 ssaleve thyroid Salmo salar cDNA Salmo salar cDNA clone ssal\_eve\_512\_128\_fwd 3', mRNA sequence  
g gi|117494683|gb|EG826900.1|EG826900 EST\_ssal\_eve\_39502 ssaleve thyroid Salmo salar cDNA Salmo salar cDNA clone ssal\_eve\_553\_290\_fwd 3', mRNA sequence  
h gi|117864876|gb|EG937572.1|EG937572 EST\_ssal\_evf\_21384 ssalevf mixed\_tissue Salmo salar cDNA Salmo salar cDNA clone ssal\_evf\_527\_049\_fwd 3', mRNA sequence  
i gi|117849135|gb|EG921831.1|EG921831 EST\_ssal\_evf\_53265 ssalevf mixed\_tissue Salmo salar cDNA Salmo salar cDNA clone ssal\_evf\_571\_138\_fwd 3', mRNA sequence  
j gi|85038394|gb|DW566572.1|DW566572 EST\_ssal\_rgb2\_30991 rgb2 Salmo salar cDNA clone ssal\_rgb2\_550\_080\_rev 5', mRNA sequence  
k gi|89843981|gb|DY700261.1|DY700261 EST\_ssal\_rgb2\_56000 ssalrgb2 mixed\_tissue Salmo salar cDNA Salmo salar cDNA clone ssal\_rgb2\_590\_191\_rev 5', mRNA sequence  
l gi|89829774|gb|DY692342.1|DY692342 EST\_ssal\_plnb\_5252 ssalplnb mixed\_tissue Salmo salar cDNA Salmo salar cDNA clone ssal\_plnb\_025\_273\_rev 5', mRNA sequence  
m gi|29324680|gb|CB513454.1|CB513454 ssalrgb531243\_rev mixed\_tissue Salmo salar cDNA, mRNA sequence  
n gi|117465845|gb|EG798064.1|EG798064 EST\_ssal\_evd\_55040 ssalevd thymus Salmo salar cDNA Salmo salar cDNA clone ssal\_evd\_574\_072\_fwd 3', mRNA sequence  
o gi|117522560|gb|EG854287.1|EG854287 EST\_ssal\_eve\_15050 ssaleve thyroid Salmo salar cDNA Salmo salar cDNA clone ssal\_eve\_520\_170\_fwd 3', mRNA sequence  
p gi|117569579|gb|EG890555.1|EG890555 EST\_ssal\_evf\_48401 ssalevf mixed\_tissue Salmo salar cDNA Salmo salar cDNA clone ssal\_evf\_564\_281\_fwd 3', mRNA sequence  
q gi|85015853|gb|DW544509.1|DW544509 EST\_ssal\_rgb2\_8928 rgb2 Salmo salar cDNA clone ssal\_rgb2\_515\_169\_rev 5', mRNA sequence  
r gi|117467692|gb|EG799911.1|EG799911 EST\_ssal\_evd\_56703 ssalevd thymus Salmo salar cDNA Salmo salar cDNA clone ssal\_evd\_576\_181\_fwd 3', mRNA sequence  
s gi|117451403|gb|EG783622.1|EG783622 EST\_ssal\_evd\_2562 ssalevd thymus Salmo salar cDNA Salmo salar cDNA clone ssal\_evd\_502\_017\_fwd 3', mRNA sequence  
t gi|57124684|gb|CX356125.1|CX356125 ssalrgb526052\_rev\_0 mixed\_tissue Salmo salar cDNA, mRNA sequence  
u gi|75981150|gb|AM041445.1|AM041445 AM041445 pGemT-easy Atlantic salmon gill subtracted library (Aeromonas salmonicda infected salmon subtracted with uninfected) Salmo salar cDNA clone GT1D2, mRNA sequence  
v gi|59834919|gb|DN140602.1|DN140602 SGP266642 Atlantic salmon Head kidney cDNA library Salmo salar cDNA clone FN4-4064 5', mRNA sequence  
w gi|84973710|gb|DW472111.1|DW472111 SGP299678 Atlantic salmon Spleen cDNA library Salmo salar cDNA clone MI5-2101 5', mRNA sequence  
x gi|60104398|gb|DN164005.1|DN164005 SGP270859 Atlantic salmon White muscle cDNA library Salmo salar cDNA clone HM4-3628 5', mRNA sequence  
y gi|83640350|gb|DW006940.1|DW006940 SGP296438 Atlantic salmon Brain cDNA library Salmo salar cDNA clone HJ4-3666 5', mRNA sequence  
z gi|45322253|gb|CK892520.1|CK892520 SGP152556 Atlantic salmon Skin cDNA library Salmo salar cDNA clone HU4-0489 5', mRNA sequence  
A gi|29328137|gb|CB516911.1|CB516911 ssalrgb509381\_rev mixed\_tissue Salmo salar cDNA, mRNA sequence  
B gi|117504478|gb|EG836237.1|EG836237 EST\_ssal\_eve\_47907 ssaleve thyroid Salmo salar cDNA Salmo salar cDNA clone ssal\_eve\_565\_063\_fwd 3', mRNA sequence  
C gi|89854874|gb|DY710997.1|DY710997 EST\_ssal\_rgb2\_66736 ssalrgb2 mixed\_tissue Salmo salar cDNA Salmo salar cDNA clone ssal\_rgb2\_608\_201\_rev 5', mRNA sequence  
D gi|117518908|gb|EG850636.1|EG850636 EST\_ssal\_eve\_7965 ssaleve thyroid Salmo salar cDNA Salmo salar cDNA clone ssal\_eve\_509\_303\_fwd 3', mRNA sequence  
E gi|117429119|gb|EG761343.1|EG761343 EST\_ssal\_sjb\_9615 ssalsjb mixed\_tissue Salmo salar cDNA Salmo salar cDNA clone ssal\_sjb\_020\_115\_fwd 3', mRNA sequence  
F gi|117821729|gb|EG894425.1|EG894425 EST\_ssal\_evf\_51884 ssalevf mixed\_tissue Salmo salar cDNA Salmo salar cDNA clone ssal\_evf\_569\_176\_fwd 3', mRNA sequence  
G gi|117486747|gb|EG818964.1|EG818964 EST\_ssal\_evd\_21048 ssalevd thymus Salmo salar cDNA Salmo salar cDNA clone ssal\_evd\_527\_067\_fwd 3', mRNA sequence  
H gi|117541225|gb|EG872670.1|EG872670 EST\_ssal\_eve\_33596 ssaleve thyroid Salmo salar cDNA Salmo salar cDNA clone ssal\_eve\_545\_232\_fwd 3', mRNA sequence  
I gi|117533549|gb|EG864994.1|EG864994 EST\_ssal\_eve\_57787 ssaleve thyroid Salmo salar cDNA Salmo salar cDNA clone ssal\_eve\_578\_297\_fwd 3', mRNA sequence  
J gi|117437876|gb|EG770099.1|EG770099 EST\_ssal\_evd\_45874 ssalevd thymus Salmo salar cDNA Salmo salar cDNA clone ssal\_evd\_561\_235\_fwd 3', mRNA sequence  
K gi|117488124|gb|EG820341.1|EG820341 EST\_ssal\_evd\_22288 ssalevd thymus Salmo salar cDNA Salmo salar cDNA clone ssal\_evd\_528\_320\_fwd 3', mRNA sequence  
L gi|117849026|gb|EG921722.1|EG921722 EST\_ssal\_evf\_907 ssalevf mixed\_tissue Salmo salar cDNA Salmo salar cDNA clone ssal\_evf\_005\_123\_fwd 3', mRNA sequence  
M gi|117501004|gb|EG833022.1|EG833022 EST\_ssal\_eve\_45013 ssaleve thyroid Salmo salar cDNA Salmo salar cDNA clone ssal\_eve\_561\_080\_fwd 3', mRNA sequence  
N gi|40541688|gb|BM413950.1|BM413950 EST00872 Atlantic salmon Lambda Zap Express ovary cDNA library Salmo salar cDNA clone oyr\_004\_f06 5' similar to DKFZP56 4B167 protein, NP\_056230, mRNA sequence

8 SNPs detected

A B C D E F G H I J K L M N O P Q R S T U V W X Y Z a b c d e f g h i j k l m n o p q r s t u v w x y z A B C D E F G H I J K L M N O P Q R S T U V W X Y Z a b c d e f g h i j k l m n o p q r s t u v w x y z A B C D E F G H I J K L M N  cosegregation weighted

363 T T T T - T T - T T T - T T T T T - T T T T T - T - - - T T T T - T - T . T . - . . . . . . . . . . . . . . . . . . . . . . . . . . . . . . . . . . . . . . . . . . . . . . . . . . . . . . . . . . . . . . . . . . . . . . . . . . . . . .   5/8 20.13
364 A A A A - A A - A A A - A A A A A - A A A A A - A - - - A A A A - A - A . A . - . . . . . . . . . . . . . . . . . . . . . . . . . . . . . . . . . . . . . . . . . . . . . . . . . . . . . . . . . . . . . . . . . . . . . . . . . . . . . .   5/8 20.13
383 . T A A G G T G G T T T T T T G G T G T T T G T T T T T T T T T T T T T . T . T . . . . . . . . . . . . . . . . . . . . . . . . . . . . . . . . . . . . . . . . . . . . . . . . . . . . . . . . . . . . . . . . . . . . . . . . . . . . . .   1/8 3.92
451 . T T T - T T T T T T T T T T - - T T T T T - T T T T T - T T - T T T T . T . T . . . . . . . T - . . . . . . . . . . . . . . . . . . . . . . . . . . . . . . . . . . . . . . . . . . . . . . . . . . . . . . . . . . . . . . . . . . . . .   1/8 4.13
452 . - - - - - - - - - - T - - - - - T T - - - - T - - T - - - - - - - T T . - . - . . . . . . . - - . . . . . . . . . . . . . . . . . . . . . . . . . . . . . . . . . . . . . . . . . . . . . . . . . . . . . . . . . . . . . . . . . . . . .   1/8 4.13
892 . . . . . . . . . . . . . . . . . . . . . . . . . . . . . . . . . C A C C C C A C A A A A C C C C A A A C C A C C A C C C C C C C C C C C C C C . . A A . . . . C . . . . C . C . A . . . . . . . . . . . . . . . . . . . . . . . . . . . .   5/8 23.83
1377 . . . . . . . . . . . . . . . . . . . . . . . . . . . . . . . . . . . . . . . . . . . . . . . . . . . . . . . . . . . . . . . . . . . . . . . C A C A A C C C C C C C C C C A C C A C A A A C A C C C C C C C C C A C C A C C C A A C C C C   5/8 24.89
1729 . . . . . . . . . . . . . . . . . . . . . . . . . . . . . . . . . . . . . . . . . . . . . . . . . . . . . . . . . . . . . . . . . . . . . . . . . . . . . . . . C C C C . . C . . . . C C C . C G . . . . . . . . G . G G . G G G G G G G G   5/8 11.12
