# Supplementary material for: Discovery and application of insertion-deletion (INDEL) polymorphisms for QTL mapping of early life-history traits in Atlantic salmon
Source: BMC Genomics. 2010 Mar 8;11:156. doi: 10.1186/1471-2164-11-156 (PMC2838853; doi:10.1186/1471-2164-11-156)
Supplement: Additional file 2 — Information on developed 76 locus single-run INDEL panel in Atlantic salmon. Information on fluorescence labeling, primer concentrations, PCR pooling and links to alignments, INDEL motifs and GENESCAN (Burge and Karlin 1997) predictions of genes/exons are available in html format. [file 1471-2164-11-156-S2.ZIP › Additionalfile2/snpsummary11953.html]

```
Cluster 4526 Contig 1

prev  Summary    Contig List  next
```

Size of Consensus sequence = 781

Number of sequences = 12

Minimum redundancy = 4

Key

A gi|117498212|gb|EG830429.1|EG830429 EST\_ssal\_eve\_42679 ssaleve thyroid Salmo salar cDNA Salmo salar cDNA clone ssal\_eve\_558\_019\_rev 5', mRNA sequence  
B gi|117485305|gb|EG817522.1|EG817522 EST\_ssal\_evd\_17014 ssalevd thymus Salmo salar cDNA Salmo salar cDNA clone ssal\_evd\_521\_283\_rev 5', mRNA sequence  
C gi|29317350|gb|CB506124.1|CB506124 ssalmgd504342 gut Salmo salar cDNA, mRNA sequence  
D gi|117526070|gb|EG857797.1|EG857797 EST\_ssal\_eve\_51309 ssaleve thyroid Salmo salar cDNA Salmo salar cDNA clone ssal\_eve\_569\_336\_fwd 3', mRNA sequence  
E gi|117526072|gb|EG857799.1|EG857799 EST\_ssal\_eve\_51310 ssaleve thyroid Salmo salar cDNA Salmo salar cDNA clone ssal\_eve\_569\_336\_rev 5', mRNA sequence  
F gi|117498213|gb|EG830430.1|EG830430 EST\_ssal\_eve\_42680 ssaleve thyroid Salmo salar cDNA Salmo salar cDNA clone ssal\_eve\_558\_019\_fwd 3', mRNA sequence  
G gi|89872942|gb|DY729065.1|DY729065 EST\_ssal\_rgb2\_84804 ssalrgb2 mixed\_tissue Salmo salar cDNA Salmo salar cDNA clone ssal\_rgb2\_638\_356\_fwd 3', mRNA sequence  
H gi|117521915|gb|EG853642.1|EG853642 EST\_ssal\_eve\_14470 ssaleve thyroid Salmo salar cDNA Salmo salar cDNA clone ssal\_eve\_519\_251\_rev 5', mRNA sequence  
I gi|89861563|gb|DY717686.1|DY717686 EST\_ssal\_rgb2\_73425 ssalrgb2 mixed\_tissue Salmo salar cDNA Salmo salar cDNA clone ssal\_rgb2\_618\_342\_fwd 3', mRNA sequence  
J gi|84987181|gb|DW537531.1|DW537531 EST\_ssal\_rgb2\_1950 rgb2 Salmo salar cDNA clone ssal\_rgb2\_504\_090\_fwd 3', mRNA sequence  
K gi|117485316|gb|EG817533.1|EG817533 EST\_ssal\_evd\_17015 ssalevd thymus Salmo salar cDNA Salmo salar cDNA clone ssal\_evd\_521\_283\_fwd 3', mRNA sequence  
L gi|117824780|gb|EG897476.1|EG897476 EST\_ssal\_evf\_36629 ssalevf mixed\_tissue Salmo salar cDNA Salmo salar cDNA clone ssal\_evf\_548\_304\_rev 5', mRNA sequence

3 SNPs detected

A B C D E F G H I J K L  cosegregation weighted

309 A A - - - A T - - T A -   1/3 33.33
432 - - C - - - C - C C - -   2/3 66.67
434 - - A - - - A - A A - -   2/3 66.67
