# Supplementary material for: Discovery and application of insertion-deletion (INDEL) polymorphisms for QTL mapping of early life-history traits in Atlantic salmon
Source: BMC Genomics. 2010 Mar 8;11:156. doi: 10.1186/1471-2164-11-156 (PMC2838853; doi:10.1186/1471-2164-11-156)
Supplement: Additional file 2 — Information on developed 76 locus single-run INDEL panel in Atlantic salmon. Information on fluorescence labeling, primer concentrations, PCR pooling and links to alignments, INDEL motifs and GENESCAN (Burge and Karlin 1997) predictions of genes/exons are available in html format. [file 1471-2164-11-156-S2.ZIP › Additionalfile2/snpsummary11971.html]

```
Cluster 4538 Contig 1

prev  Summary    Contig List  next
```

Size of Consensus sequence = 610

Number of sequences = 14

Minimum redundancy = 5

Key

A gi|117543312|gb|EG874757.1|EG874757 EST\_ssal\_eve\_35474 ssaleve thyroid Salmo salar cDNA Salmo salar cDNA clone ssal\_eve\_548\_071\_fwd 3', mRNA sequence  
B gi|117482970|gb|EG815187.1|EG815187 EST\_ssal\_evd\_34989 ssalevd thymus Salmo salar cDNA Salmo salar cDNA clone ssal\_evd\_546\_244\_fwd 3', mRNA sequence  
C gi|117482972|gb|EG815189.1|EG815189 EST\_ssal\_evd\_34990 ssalevd thymus Salmo salar cDNA Salmo salar cDNA clone ssal\_evd\_546\_244\_rev 5', mRNA sequence  
D gi|117468813|gb|EG801032.1|EG801032 EST\_ssal\_evd\_57712 ssalevd thymus Salmo salar cDNA Salmo salar cDNA clone ssal\_evd\_577\_321\_fwd 3', mRNA sequence  
E gi|117835415|gb|EG908111.1|EG908111 EST\_ssal\_evf\_10457 ssalevf mixed\_tissue Salmo salar cDNA Salmo salar cDNA clone ssal\_evf\_512\_142\_rev 5', mRNA sequence  
F gi|117835416|gb|EG908112.1|EG908112 EST\_ssal\_evf\_10458 ssalevf mixed\_tissue Salmo salar cDNA Salmo salar cDNA clone ssal\_evf\_512\_142\_fwd 3', mRNA sequence  
G gi|117468814|gb|EG801033.1|EG801033 EST\_ssal\_evd\_57713 ssalevd thymus Salmo salar cDNA Salmo salar cDNA clone ssal\_evd\_577\_321\_rev 5', mRNA sequence  
H gi|117543311|gb|EG874756.1|EG874756 EST\_ssal\_eve\_35473 ssaleve thyroid Salmo salar cDNA Salmo salar cDNA clone ssal\_eve\_548\_071\_rev 5', mRNA sequence  
I gi|117464307|gb|EG796526.1|EG796526 EST\_ssal\_evd\_16194 ssalevd thymus Salmo salar cDNA Salmo salar cDNA clone ssal\_evd\_520\_242\_fwd 3', mRNA sequence  
J gi|117475767|gb|EG807986.1|EG807986 EST\_ssal\_evd\_28508 ssalevd thymus Salmo salar cDNA Salmo salar cDNA clone ssal\_evd\_537\_124\_rev 5', mRNA sequence  
K gi|117475768|gb|EG807987.1|EG807987 EST\_ssal\_evd\_28509 ssalevd thymus Salmo salar cDNA Salmo salar cDNA clone ssal\_evd\_537\_124\_fwd 3', mRNA sequence  
L gi|117492608|gb|EG824825.1|EG824825 EST\_ssal\_evd\_26323 ssalevd thymus Salmo salar cDNA Salmo salar cDNA clone ssal\_evd\_534\_115\_rev 5', mRNA sequence  
M gi|117492609|gb|EG824826.1|EG824826 EST\_ssal\_evd\_26324 ssalevd thymus Salmo salar cDNA Salmo salar cDNA clone ssal\_evd\_534\_115\_fwd 3', mRNA sequence  
N gi|117464308|gb|EG796527.1|EG796527 EST\_ssal\_evd\_16195 ssalevd thymus Salmo salar cDNA Salmo salar cDNA clone ssal\_evd\_520\_242\_rev 5', mRNA sequence

3 SNPs detected

A B C D E F G H I J K L M N  cosegregation weighted

537 . G G G G G G G - - - - - -   3/3 92.86
538 . A A A A A A A - - - - - -   3/3 92.86
542 . G G G G G G G A A A A A A   3/3 92.86
