# Supplementary material for: Discovery and application of insertion-deletion (INDEL) polymorphisms for QTL mapping of early life-history traits in Atlantic salmon
Source: BMC Genomics. 2010 Mar 8;11:156. doi: 10.1186/1471-2164-11-156 (PMC2838853; doi:10.1186/1471-2164-11-156)
Supplement: Additional file 2 — Information on developed 76 locus single-run INDEL panel in Atlantic salmon. Information on fluorescence labeling, primer concentrations, PCR pooling and links to alignments, INDEL motifs and GENESCAN (Burge and Karlin 1997) predictions of genes/exons are available in html format. [file 1471-2164-11-156-S2.ZIP › Additionalfile2/snpsummary1271.html]

```
Cluster 172 Contig 4

prev  Summary    Contig List  next
```

Size of Consensus sequence = 2337

Number of sequences = 51

Minimum redundancy = 6

Key

A gi|45309836|gb|CK880205.1|CK880205 SGP140501 Atlantic salmon Head kidney cDNA library Salmo salar cDNA clone FN6-0418 5', mRNA sequence  
B gi|117852542|gb|EG925238.1|EG925238 EST\_ssal\_evf\_27871 ssalevf mixed\_tissue Salmo salar cDNA Salmo salar cDNA clone ssal\_evf\_536\_365\_rev 5', mRNA sequence  
C gi|117852543|gb|EG925239.1|EG925239 EST\_ssal\_evf\_27872 ssalevf mixed\_tissue Salmo salar cDNA Salmo salar cDNA clone ssal\_evf\_536\_365\_fwd 3', mRNA sequence  
D gi|119021060|gb|EG355329.2|EG355329 Ss\_Kidn\_24G05\_M13 Kidney SSH library Salmo salar cDNA clone Ss\_Kidn\_24G05 5', mRNA sequence  
E gi|117823096|gb|EG895792.1|EG895792 EST\_ssal\_evf\_4713 ssalevf mixed\_tissue Salmo salar cDNA Salmo salar cDNA clone ssal\_evf\_504\_238\_fwd 3', mRNA sequence  
F gi|117823097|gb|EG895793.1|EG895793 EST\_ssal\_evf\_4714 ssalevf mixed\_tissue Salmo salar cDNA Salmo salar cDNA clone ssal\_evf\_504\_238\_rev 5', mRNA sequence  
G gi|24341587|gb|CA040661.1|CA040661 ssalshc003018 spleen Salmo salar cDNA, mRNA sequence  
H gi|117848933|gb|EG921629.1|EG921629 EST\_ssal\_evf\_823 ssalevf mixed\_tissue Salmo salar cDNA Salmo salar cDNA clone ssal\_evf\_005\_080\_fwd 3', mRNA sequence  
I gi|117870441|gb|EG943137.1|EG943137 EST\_ssal\_evf\_26393 ssalevf mixed\_tissue Salmo salar cDNA Salmo salar cDNA clone ssal\_evf\_534\_366\_rev 5', mRNA sequence  
J gi|117870442|gb|EG943138.1|EG943138 EST\_ssal\_evf\_26394 ssalevf mixed\_tissue Salmo salar cDNA Salmo salar cDNA clone ssal\_evf\_534\_366\_fwd 3', mRNA sequence  
K gi|45319397|gb|CK889664.1|CK889664 SGP149886 Atlantic salmon Ovaries cDNA library Salmo salar cDNA clone KG6-0185 5', mRNA sequence  
L gi|117844232|gb|EG916928.1|EG916928 EST\_ssal\_evf\_56133 ssalevf mixed\_tissue Salmo salar cDNA Salmo salar cDNA clone ssal\_evf\_575\_109\_rev 5', mRNA sequence  
M gi|117848934|gb|EG921630.1|EG921630 EST\_ssal\_evf\_824 ssalevf mixed\_tissue Salmo salar cDNA Salmo salar cDNA clone ssal\_evf\_005\_080\_rev 5', mRNA sequence  
N gi|117461822|gb|EG794041.1|EG794041 EST\_ssal\_evd\_13957 ssalevd thymus Salmo salar cDNA Salmo salar cDNA clone ssal\_evd\_517\_211\_rev 5', mRNA sequence  
O gi|89869642|gb|DY725765.1|DY725765 EST\_ssal\_rgb2\_81504 ssalrgb2 mixed\_tissue Salmo salar cDNA Salmo salar cDNA clone ssal\_rgb2\_633\_318\_fwd 3', mRNA sequence  
P gi|25999915|gb|CA770660.1|CA770660 ssalshc505033 spleen Salmo salar cDNA, mRNA sequence  
Q gi|117461821|gb|EG794040.1|EG794040 EST\_ssal\_evd\_13956 ssalevd thymus Salmo salar cDNA Salmo salar cDNA clone ssal\_evd\_517\_211\_fwd 3', mRNA sequence  
R gi|25998004|gb|CA768749.1|CA768749 ssalga002011 head Salmo salar cDNA, mRNA sequence  
S gi|117510731|gb|EG842490.1|EG842490 EST\_ssal\_eve\_5134 ssaleve thyroid Salmo salar cDNA Salmo salar cDNA clone ssal\_eve\_505\_381\_rev 5', mRNA sequence  
T gi|24340730|gb|CA039889.1|CA039889 ssalshc501149 spleen Salmo salar cDNA, mRNA sequence  
U gi|117851530|gb|EG924226.1|EG924226 EST\_ssal\_evf\_26960 ssalevf mixed\_tissue Salmo salar cDNA Salmo salar cDNA clone ssal\_evf\_535\_281\_rev 5', mRNA sequence  
V gi|29312649|gb|CB501423.1|CB501423 ssalga507301 head Salmo salar cDNA, mRNA sequence  
W gi|117506921|gb|EG838680.1|EG838680 EST\_ssal\_eve\_1704 ssaleve thyroid Salmo salar cDNA Salmo salar cDNA clone ssal\_eve\_501\_146\_rev 5', mRNA sequence  
X gi|117540571|gb|EG872016.1|EG872016 EST\_ssal\_eve\_33007 ssaleve thyroid Salmo salar cDNA Salmo salar cDNA clone ssal\_eve\_544\_310\_rev 5', mRNA sequence  
Y gi|29313905|gb|CB502679.1|CB502679 ssalmge503147 gut Salmo salar cDNA, mRNA sequence  
Z gi|117538041|gb|EG869486.1|EG869486 EST\_ssal\_eve\_18301 ssaleve thyroid Salmo salar cDNA Salmo salar cDNA clone ssal\_eve\_524\_363\_rev 5', mRNA sequence  
a gi|117538052|gb|EG869497.1|EG869497 EST\_ssal\_eve\_18302 ssaleve thyroid Salmo salar cDNA Salmo salar cDNA clone ssal\_eve\_524\_363\_fwd 3', mRNA sequence  
b gi|117505071|gb|EG836830.1|EG836830 EST\_ssal\_eve\_11438 ssaleve thyroid Salmo salar cDNA Salmo salar cDNA clone ssal\_eve\_515\_210\_rev 5', mRNA sequence  
c gi|117540570|gb|EG872015.1|EG872015 EST\_ssal\_eve\_33006 ssaleve thyroid Salmo salar cDNA Salmo salar cDNA clone ssal\_eve\_544\_310\_fwd 3', mRNA sequence  
d gi|89871922|gb|DY728045.1|DY728045 EST\_ssal\_rgb2\_83784 ssalrgb2 mixed\_tissue Salmo salar cDNA Salmo salar cDNA clone ssal\_rgb2\_637\_137\_fwd 3', mRNA sequence  
e gi|117456238|gb|EG788457.1|EG788457 EST\_ssal\_evd\_8930 ssalevd thymus Salmo salar cDNA Salmo salar cDNA clone ssal\_evd\_510\_265\_rev 5', mRNA sequence  
f gi|117480747|gb|EG812964.1|EG812964 EST\_ssal\_evd\_32988 ssalevd thymus Salmo salar cDNA Salmo salar cDNA clone ssal\_evd\_543\_347\_fwd 3', mRNA sequence  
g gi|117480748|gb|EG812965.1|EG812965 EST\_ssal\_evd\_32989 ssalevd thymus Salmo salar cDNA Salmo salar cDNA clone ssal\_evd\_543\_347\_rev 5', mRNA sequence  
h gi|117844233|gb|EG916929.1|EG916929 EST\_ssal\_evf\_56134 ssalevf mixed\_tissue Salmo salar cDNA Salmo salar cDNA clone ssal\_evf\_575\_109\_fwd 3', mRNA sequence  
i gi|85054995|gb|DW583173.1|DW583173 EST\_ssal\_rgb2\_47592 rgb2 Salmo salar cDNA clone ssal\_rgb2\_576\_326\_fwd 3', mRNA sequence  
j gi|117503117|gb|EG834876.1|EG834876 EST\_ssal\_eve\_46681 ssaleve thyroid Salmo salar cDNA Salmo salar cDNA clone ssal\_eve\_563\_184\_rev 5', mRNA sequence  
k gi|117430000|gb|EG762224.1|EG762224 EST\_ssal\_sjb\_322 ssalsjb mixed\_tissue Salmo salar cDNA Salmo salar cDNA clone ssal\_sjb\_003\_017\_fwd 3', mRNA sequence  
l gi|84973597|gb|DW471998.1|DW471998 SGP286115 Atlantic salmon Spleen cDNA library Salmo salar cDNA clone MI5-1968 5', mRNA sequence  
m gi|117505070|gb|EG836829.1|EG836829 EST\_ssal\_eve\_11437 ssaleve thyroid Salmo salar cDNA Salmo salar cDNA clone ssal\_eve\_515\_210\_fwd 3', mRNA sequence  
n gi|117851529|gb|EG924225.1|EG924225 EST\_ssal\_evf\_26959 ssalevf mixed\_tissue Salmo salar cDNA Salmo salar cDNA clone ssal\_evf\_535\_281\_fwd 3', mRNA sequence  
o gi|117456239|gb|EG788458.1|EG788458 EST\_ssal\_evd\_8931 ssalevd thymus Salmo salar cDNA Salmo salar cDNA clone ssal\_evd\_510\_265\_fwd 3', mRNA sequence  
p gi|85054996|gb|DW583174.1|DW583174 EST\_ssal\_rgb2\_47593 rgb2 Salmo salar cDNA clone ssal\_rgb2\_576\_326\_rev 5', mRNA sequence  
q gi|117510732|gb|EG842491.1|EG842491 EST\_ssal\_eve\_5135 ssaleve thyroid Salmo salar cDNA Salmo salar cDNA clone ssal\_eve\_505\_381\_fwd 3', mRNA sequence  
r gi|117506920|gb|EG838679.1|EG838679 EST\_ssal\_eve\_1703 ssaleve thyroid Salmo salar cDNA Salmo salar cDNA clone ssal\_eve\_501\_146\_fwd 3', mRNA sequence  
s gi|89871921|gb|DY728044.1|DY728044 EST\_ssal\_rgb2\_83783 ssalrgb2 mixed\_tissue Salmo salar cDNA Salmo salar cDNA clone ssal\_rgb2\_637\_137\_rev 5', mRNA sequence  
t gi|24345289|gb|CA044213.1|CA044213 ssalplnb513112 gut Salmo salar cDNA, mRNA sequence  
u gi|89831673|gb|DY693531.1|DY693531 EST\_ssal\_rgb2\_49270 ssalrgb2 mixed\_tissue Salmo salar cDNA Salmo salar cDNA clone ssal\_rgb2\_579\_348\_rev 5', mRNA sequence  
v gi|89869641|gb|DY725764.1|DY725764 EST\_ssal\_rgb2\_81503 ssalrgb2 mixed\_tissue Salmo salar cDNA Salmo salar cDNA clone ssal\_rgb2\_633\_318\_rev 5', mRNA sequence  
w gi|89850631|gb|DY706754.1|DY706754 EST\_ssal\_rgb2\_62493 ssalrgb2 mixed\_tissue Salmo salar cDNA Salmo salar cDNA clone ssal\_rgb2\_600\_344\_rev 5', mRNA sequence  
x gi|15844079|gb|BG934251.1|BG934251 SK1-0546 Atlantic Salmon kidney Salmo salar cDNA clone SK1-0546 similar to Actin related protein (arp3), mRNA sequence  
y gi|15844151|gb|BG934323.1|BG934323 SK1-0622 Atlantic Salmon kidney Salmo salar cDNA clone SK1-0622 similar to Actin related protein (arp3), mRNA sequence

5 SNPs detected

A B C D E F G H I J K L M N O P Q R S T U V W X Y Z a b c d e f g h i j k l m n o p q r s t u v w x y  cosegregation weighted

73 T T T . - - T - . . . - - . . T . . T T . . - T - . . . . . . . . . . . . . . . . . . . . . . . . . .   1/5 5.88
194 G A G G G A G G G G G G G A . G G G A G G G G A G G G A G G G G G G G G . . . . . . . . . . . . . . .   1/5 13.73
638 . . . . . . . T T T . . . . . A - - - - - - T T T - - - T - - T T T - T T - - - . - . . . . . . . . .   3/5 34.12
639 . . . . . . . C C C . . . . . T - - - - - - C C C - - - C - - C C C - C C - - - . - . . . . . . . . .   3/5 34.12
640 . . . . . . . A A A . . . . . . - - - - - - A A A - - - A - - A A A - A A - - - . - . . . . . . . . .   3/5 32.94
