# Supplementary material for: Discovery and application of insertion-deletion (INDEL) polymorphisms for QTL mapping of early life-history traits in Atlantic salmon
Source: BMC Genomics. 2010 Mar 8;11:156. doi: 10.1186/1471-2164-11-156 (PMC2838853; doi:10.1186/1471-2164-11-156)
Supplement: Additional file 2 — Information on developed 76 locus single-run INDEL panel in Atlantic salmon. Information on fluorescence labeling, primer concentrations, PCR pooling and links to alignments, INDEL motifs and GENESCAN (Burge and Karlin 1997) predictions of genes/exons are available in html format. [file 1471-2164-11-156-S2.ZIP › Additionalfile2/snpsummary12783.html]

```
Cluster 5033 Contig 1

prev  Summary    Contig List  next
```

Size of Consensus sequence = 1468

Number of sequences = 11

Minimum redundancy = 4

Key

A gi|117498774|gb|EG830991.1|EG830991 EST\_ssal\_eve\_43184 ssaleve thyroid Salmo salar cDNA Salmo salar cDNA clone ssal\_eve\_558\_278\_fwd 3', mRNA sequence  
B gi|117424503|gb|EG756727.1|EG756727 EST\_ssal\_sjb\_5457 ssalsjb mixed\_tissue Salmo salar cDNA Salmo salar cDNA clone ssal\_sjb\_012\_384\_fwd 3', mRNA sequence  
C gi|117426570|gb|EG758794.1|EG758794 EST\_ssal\_sjb\_7320 ssalsjb mixed\_tissue Salmo salar cDNA Salmo salar cDNA clone ssal\_sjb\_015\_275\_fwd 3', mRNA sequence  
D gi|117432683|gb|EG764907.1|EG764907 EST\_ssal\_sjb\_2737 ssalsjb mixed\_tissue Salmo salar cDNA Salmo salar cDNA clone ssal\_sjb\_008\_372\_fwd 3', mRNA sequence  
E gi|117426673|gb|EG758897.1|EG758897 EST\_ssal\_sjb\_7413 ssalsjb mixed\_tissue Salmo salar cDNA Salmo salar cDNA clone ssal\_sjb\_015\_330\_fwd 3', mRNA sequence  
F gi|117424504|gb|EG756728.1|EG756728 EST\_ssal\_sjb\_5458 ssalsjb mixed\_tissue Salmo salar cDNA Salmo salar cDNA clone ssal\_sjb\_012\_384\_rev 5', mRNA sequence  
G gi|117498775|gb|EG830992.1|EG830992 EST\_ssal\_eve\_43185 ssaleve thyroid Salmo salar cDNA Salmo salar cDNA clone ssal\_eve\_558\_278\_rev 5', mRNA sequence  
H gi|117426674|gb|EG758898.1|EG758898 EST\_ssal\_sjb\_7414 ssalsjb mixed\_tissue Salmo salar cDNA Salmo salar cDNA clone ssal\_sjb\_015\_330\_rev 5', mRNA sequence  
I gi|84567661|gb|DW339280.1|DW339280 SGP300525 Atlantic salmon Ovaries cDNA library Salmo salar cDNA clone KG4-2234 5', mRNA sequence  
J gi|117505111|gb|EG836870.1|EG836870 EST\_ssal\_eve\_11474 ssaleve thyroid Salmo salar cDNA Salmo salar cDNA clone ssal\_eve\_515\_230\_rev 5', mRNA sequence  
K gi|117505112|gb|EG836871.1|EG836871 EST\_ssal\_eve\_11475 ssaleve thyroid Salmo salar cDNA Salmo salar cDNA clone ssal\_eve\_515\_230\_fwd 3', mRNA sequence

2 SNPs detected

A B C D E F G H I J K  cosegregation weighted

698 . . - - - - T - T T T   2/2 81.82
699 . . - - - - T - T T T   2/2 81.82
