# Supplementary material for: Discovery and application of insertion-deletion (INDEL) polymorphisms for QTL mapping of early life-history traits in Atlantic salmon
Source: BMC Genomics. 2010 Mar 8;11:156. doi: 10.1186/1471-2164-11-156 (PMC2838853; doi:10.1186/1471-2164-11-156)
Supplement: Additional file 2 — Information on developed 76 locus single-run INDEL panel in Atlantic salmon. Information on fluorescence labeling, primer concentrations, PCR pooling and links to alignments, INDEL motifs and GENESCAN (Burge and Karlin 1997) predictions of genes/exons are available in html format. [file 1471-2164-11-156-S2.ZIP › Additionalfile2/snpsummary13028.html]

```
Cluster 5187 Contig 1

prev  Summary    Contig List  next
```

Size of Consensus sequence = 861

Number of sequences = 11

Minimum redundancy = 4

Key

A gi|89873727|gb|DY729850.1|DY729850 EST\_ssal\_rgb2\_85589 ssalrgb2 mixed\_tissue Salmo salar cDNA Salmo salar cDNA clone ssal\_rgb2\_640\_039\_fwd 3', mRNA sequence  
B gi|24390695|gb|CA060452.1|CA060452 ssalrga508034 mixed\_tissue Salmo salar cDNA, mRNA sequence  
C gi|89849300|gb|DY705423.1|DY705423 EST\_ssal\_rgb2\_61162 ssalrgb2 mixed\_tissue Salmo salar cDNA Salmo salar cDNA clone ssal\_rgb2\_598\_168\_fwd 3', mRNA sequence  
D gi|89860827|gb|DY716950.1|DY716950 EST\_ssal\_rgb2\_72689 ssalrgb2 mixed\_tissue Salmo salar cDNA Salmo salar cDNA clone ssal\_rgb2\_617\_279\_fwd 3', mRNA sequence  
E gi|62630495|gb|CX727576.1|CX727576 EST00257 SSH3 Salmo salar cDNA similar to proline-rich tyrosine kinase 2, mRNA sequence  
F gi|85043517|gb|DW571695.1|DW571695 EST\_ssal\_rgb2\_36114 rgb2 Salmo salar cDNA clone ssal\_rgb2\_558\_291\_fwd 3', mRNA sequence  
G gi|85040429|gb|DW568607.1|DW568607 EST\_ssal\_rgb2\_33026 rgb2 Salmo salar cDNA clone ssal\_rgb2\_553\_187\_fwd 3', mRNA sequence  
H gi|117491076|gb|EG823293.1|EG823293 EST\_ssal\_evd\_24944 ssalevd thymus Salmo salar cDNA Salmo salar cDNA clone ssal\_evd\_532\_167\_rev 5', mRNA sequence  
I gi|24381153|gb|CA050910.1|CA050910 ssalrgb526219 mixed\_tissue Salmo salar cDNA, mRNA sequence  
J gi|25998765|gb|CA769510.1|CA769510 ssalrgb526355 mixed\_tissue Salmo salar cDNA, mRNA sequence  
K gi|117491077|gb|EG823294.1|EG823294 EST\_ssal\_evd\_24945 ssalevd thymus Salmo salar cDNA Salmo salar cDNA clone ssal\_evd\_532\_167\_fwd 3', mRNA sequence

6 SNPs detected

A B C D E F G H I J K  cosegregation weighted

201 T T - - . - T - T T -   6/6 90.91
202 G G - - . - G - G G -   6/6 90.91
203 T T - - . - T - T T -   6/6 90.91
204 G G - - . - G - G G -   6/6 90.91
205 T T - - . - T - T T -   6/6 90.91
206 G G - - . - G - G G -   6/6 90.91
