# Supplementary material for: Discovery and application of insertion-deletion (INDEL) polymorphisms for QTL mapping of early life-history traits in Atlantic salmon
Source: BMC Genomics. 2010 Mar 8;11:156. doi: 10.1186/1471-2164-11-156 (PMC2838853; doi:10.1186/1471-2164-11-156)
Supplement: Additional file 2 — Information on developed 76 locus single-run INDEL panel in Atlantic salmon. Information on fluorescence labeling, primer concentrations, PCR pooling and links to alignments, INDEL motifs and GENESCAN (Burge and Karlin 1997) predictions of genes/exons are available in html format. [file 1471-2164-11-156-S2.ZIP › Additionalfile2/snpsummary13049.html]

```
Cluster 5199 Contig 1

prev  Summary    Contig List  next
```

Size of Consensus sequence = 800

Number of sequences = 6

Minimum redundancy = 2

Key

A gi|84982513|gb|DW532863.1|DW532863 EST\_ssal\_plnb\_1499 plnb Salmo salar cDNA clone ssal\_plnb\_012\_024\_rev 5', mRNA sequence  
B gi|24391696|gb|CA061453.1|CA061453 ssalrgb534381 mixed\_tissue Salmo salar cDNA, mRNA sequence  
C gi|24335265|gb|CA036423.1|CA036423 ssalob008020 reproductive Salmo salar cDNA, mRNA sequence  
D gi|117477120|gb|EG809339.1|EG809339 EST\_ssal\_evd\_29724 ssalevd thymus Salmo salar cDNA Salmo salar cDNA clone ssal\_evd\_538\_384\_rev 5', mRNA sequence  
E gi|89829293|gb|DY692052.1|DY692052 EST\_ssal\_plnb\_4962 ssalplnb mixed\_tissue Salmo salar cDNA Salmo salar cDNA clone ssal\_plnb\_025\_115\_rev 5', mRNA sequence  
F gi|89829294|gb|DY692053.1|DY692053 EST\_ssal\_plnb\_4963 ssalplnb mixed\_tissue Salmo salar cDNA Salmo salar cDNA clone ssal\_plnb\_025\_115\_fwd 3', mRNA sequence

5 SNPs detected

A B C D E F  cosegregation weighted

145 - A A - - -   5/5 100.00
146 - C C - - -   5/5 100.00
147 - A A - - -   5/5 100.00
148 - C C - - -   5/5 100.00
413 C A A C C C   5/5 100.00
