# Supplementary material for: Discovery and application of insertion-deletion (INDEL) polymorphisms for QTL mapping of early life-history traits in Atlantic salmon
Source: BMC Genomics. 2010 Mar 8;11:156. doi: 10.1186/1471-2164-11-156 (PMC2838853; doi:10.1186/1471-2164-11-156)
Supplement: Additional file 2 — Information on developed 76 locus single-run INDEL panel in Atlantic salmon. Information on fluorescence labeling, primer concentrations, PCR pooling and links to alignments, INDEL motifs and GENESCAN (Burge and Karlin 1997) predictions of genes/exons are available in html format. [file 1471-2164-11-156-S2.ZIP › Additionalfile2/snpsummary13066.html]

```
Cluster 5208 Contig 1

prev  Summary    Contig List  next
```

Size of Consensus sequence = 1068

Number of sequences = 12

Minimum redundancy = 4

Key

A gi|117824001|gb|EG896697.1|EG896697 EST\_ssal\_evf\_5528 ssalevf mixed\_tissue Salmo salar cDNA Salmo salar cDNA clone ssal\_evf\_505\_278\_fwd 3', mRNA sequence  
B gi|117449460|gb|EG781679.1|EG781679 EST\_ssal\_evd\_2387 ssalevd thymus Salmo salar cDNA Salmo salar cDNA clone ssal\_evd\_501\_310\_fwd 3', mRNA sequence  
C gi|117449449|gb|EG781668.1|EG781668 EST\_ssal\_evd\_2386 ssalevd thymus Salmo salar cDNA Salmo salar cDNA clone ssal\_evd\_501\_310\_rev 5', mRNA sequence  
D gi|117831334|gb|EG904030.1|EG904030 EST\_ssal\_evf\_6784 ssalevf mixed\_tissue Salmo salar cDNA Salmo salar cDNA clone ssal\_evf\_507\_152\_rev 5', mRNA sequence  
E gi|117831336|gb|EG904032.1|EG904032 EST\_ssal\_evf\_6785 ssalevf mixed\_tissue Salmo salar cDNA Salmo salar cDNA clone ssal\_evf\_507\_152\_fwd 3', mRNA sequence  
F gi|24335267|gb|CA036425.1|CA036425 ssalob008025 reproductive Salmo salar cDNA, mRNA sequence  
G gi|29323588|gb|CB512362.1|CB512362 ssalrgb551148 mixed\_tissue Salmo salar cDNA, mRNA sequence  
H gi|117475375|gb|EG807594.1|EG807594 EST\_ssal\_evd\_28154 ssalevd thymus Salmo salar cDNA Salmo salar cDNA clone ssal\_evd\_536\_319\_fwd 3', mRNA sequence  
I gi|117475374|gb|EG807593.1|EG807593 EST\_ssal\_evd\_28153 ssalevd thymus Salmo salar cDNA Salmo salar cDNA clone ssal\_evd\_536\_319\_rev 5', mRNA sequence  
J gi|85033179|gb|DW561835.1|DW561835 EST\_ssal\_rgb2\_26254 rgb2 Salmo salar cDNA clone ssal\_rgb2\_542\_250\_fwd 3', mRNA sequence  
K gi|117824002|gb|EG896698.1|EG896698 EST\_ssal\_evf\_5529 ssalevf mixed\_tissue Salmo salar cDNA Salmo salar cDNA clone ssal\_evf\_505\_278\_rev 5', mRNA sequence  
L gi|84984227|gb|DW534577.1|DW534577 EST\_ssal\_plnb\_3213 plnb Salmo salar cDNA clone ssal\_plnb\_020\_309\_fwd 3', mRNA sequence

3 SNPs detected

A B C D E F G H I J K L  cosegregation weighted

692 - A A - - A A A A A - A   1/3 33.33
836 . T T T T - - T T - - T   2/3 61.11
837 . T T T T - - T T - - T   2/3 61.11
