# Supplementary material for: Discovery and application of insertion-deletion (INDEL) polymorphisms for QTL mapping of early life-history traits in Atlantic salmon
Source: BMC Genomics. 2010 Mar 8;11:156. doi: 10.1186/1471-2164-11-156 (PMC2838853; doi:10.1186/1471-2164-11-156)
Supplement: Additional file 2 — Information on developed 76 locus single-run INDEL panel in Atlantic salmon. Information on fluorescence labeling, primer concentrations, PCR pooling and links to alignments, INDEL motifs and GENESCAN (Burge and Karlin 1997) predictions of genes/exons are available in html format. [file 1471-2164-11-156-S2.ZIP › Additionalfile2/snpsummary1309.html]

```
Cluster 178 Contig 2

prev  Summary    Contig List  next
```

Size of Consensus sequence = 1753

Number of sequences = 78

Minimum redundancy = 6

Key

A gi|117476702|gb|EG808921.1|EG808921 EST\_ssal\_evd\_29349 ssalevd thymus Salmo salar cDNA Salmo salar cDNA clone ssal\_evd\_538\_184\_fwd 3', mRNA sequence  
B gi|117475898|gb|EG808117.1|EG808117 EST\_ssal\_evd\_28625 ssalevd thymus Salmo salar cDNA Salmo salar cDNA clone ssal\_evd\_537\_185\_fwd 3', mRNA sequence  
C gi|117476439|gb|EG808658.1|EG808658 EST\_ssal\_evd\_29111 ssalevd thymus Salmo salar cDNA Salmo salar cDNA clone ssal\_evd\_538\_058\_fwd 3', mRNA sequence  
D gi|117434435|gb|EG766658.1|EG766658 EST\_ssal\_evd\_42775 ssalevd thymus Salmo salar cDNA Salmo salar cDNA clone ssal\_evd\_557\_140\_fwd 3', mRNA sequence  
E gi|117437254|gb|EG769477.1|EG769477 EST\_ssal\_evd\_45313 ssalevd thymus Salmo salar cDNA Salmo salar cDNA clone ssal\_evd\_560\_321\_fwd 3', mRNA sequence  
F gi|117449079|gb|EG781298.1|EG781298 EST\_ssal\_evd\_37953 ssalevd thymus Salmo salar cDNA Salmo salar cDNA clone ssal\_evd\_550\_262\_fwd 3', mRNA sequence  
G gi|117436812|gb|EG769035.1|EG769035 EST\_ssal\_evd\_44915 ssalevd thymus Salmo salar cDNA Salmo salar cDNA clone ssal\_evd\_560\_115\_fwd 3', mRNA sequence  
H gi|117471793|gb|EG804012.1|EG804012 EST\_ssal\_evd\_52996 ssalevd thymus Salmo salar cDNA Salmo salar cDNA clone ssal\_evd\_571\_164\_fwd 3', mRNA sequence  
I gi|117544036|gb|EG875481.1|EG875481 EST\_ssal\_eve\_19292 ssaleve thyroid Salmo salar cDNA Salmo salar cDNA clone ssal\_eve\_526\_101\_fwd 3', mRNA sequence  
J gi|117483565|gb|EG815782.1|EG815782 EST\_ssal\_evd\_35524 ssalevd thymus Salmo salar cDNA Salmo salar cDNA clone ssal\_evd\_547\_145\_fwd 3', mRNA sequence  
K gi|117453678|gb|EG785897.1|EG785897 EST\_ssal\_evd\_42092 ssalevd thymus Salmo salar cDNA Salmo salar cDNA clone ssal\_evd\_556\_154\_fwd 3', mRNA sequence  
L gi|117437927|gb|EG770150.1|EG770150 EST\_ssal\_evd\_45919 ssalevd thymus Salmo salar cDNA Salmo salar cDNA clone ssal\_evd\_561\_258\_fwd 3', mRNA sequence  
M gi|117450635|gb|EG782854.1|EG782854 EST\_ssal\_evd\_39353 ssalevd thymus Salmo salar cDNA Salmo salar cDNA clone ssal\_evd\_552\_254\_fwd 3', mRNA sequence  
N gi|117462591|gb|EG794810.1|EG794810 EST\_ssal\_evd\_53968 ssalevd thymus Salmo salar cDNA Salmo salar cDNA clone ssal\_evd\_572\_282\_fwd 3', mRNA sequence  
O gi|117457869|gb|EG790088.1|EG790088 EST\_ssal\_evd\_53543 ssalevd thymus Salmo salar cDNA Salmo salar cDNA clone ssal\_evd\_572\_063\_fwd 3', mRNA sequence  
P gi|117490375|gb|EG822592.1|EG822592 EST\_ssal\_evd\_24313 ssalevd thymus Salmo salar cDNA Salmo salar cDNA clone ssal\_evd\_531\_229\_fwd 3', mRNA sequence  
Q gi|117484315|gb|EG816532.1|EG816532 EST\_ssal\_evd\_18858 ssalevd thymus Salmo salar cDNA Salmo salar cDNA clone ssal\_evd\_524\_078\_fwd 3', mRNA sequence  
R gi|117436019|gb|EG768242.1|EG768242 EST\_ssal\_evd\_44201 ssalevd thymus Salmo salar cDNA Salmo salar cDNA clone ssal\_evd\_559\_127\_fwd 3', mRNA sequence  
S gi|117473605|gb|EG805824.1|EG805824 EST\_ssal\_evd\_6561 ssalevd thymus Salmo salar cDNA Salmo salar cDNA clone ssal\_evd\_507\_182\_fwd 3', mRNA sequence  
T gi|117447988|gb|EG780207.1|EG780207 EST\_ssal\_evd\_36971 ssalevd thymus Salmo salar cDNA Salmo salar cDNA clone ssal\_evd\_549\_135\_rev 5', mRNA sequence  
U gi|29320206|gb|CB508688.1|CB508688 ssaltc020031 reproductive Salmo salar cDNA, mRNA sequence  
V gi|117462602|gb|EG794821.1|EG794821 EST\_ssal\_evd\_53969 ssalevd thymus Salmo salar cDNA Salmo salar cDNA clone ssal\_evd\_572\_282\_rev 5', mRNA sequence  
W gi|117477541|gb|EG809760.1|EG809760 EST\_ssal\_evd\_30104 ssalevd thymus Salmo salar cDNA Salmo salar cDNA clone ssal\_evd\_539\_202\_rev 5', mRNA sequence  
X gi|117471804|gb|EG804023.1|EG804023 EST\_ssal\_evd\_52997 ssalevd thymus Salmo salar cDNA Salmo salar cDNA clone ssal\_evd\_571\_164\_rev 5', mRNA sequence  
Y gi|117490374|gb|EG822591.1|EG822591 EST\_ssal\_evd\_24312 ssalevd thymus Salmo salar cDNA Salmo salar cDNA clone ssal\_evd\_531\_229\_rev 5', mRNA sequence  
Z gi|117544035|gb|EG875480.1|EG875480 EST\_ssal\_eve\_19291 ssaleve thyroid Salmo salar cDNA Salmo salar cDNA clone ssal\_eve\_526\_101\_rev 5', mRNA sequence  
a gi|117458937|gb|EG791156.1|EG791156 EST\_ssal\_evd\_11360 ssalevd thymus Salmo salar cDNA Salmo salar cDNA clone ssal\_evd\_514\_017\_rev 5', mRNA sequence  
b gi|117458664|gb|EG790883.1|EG790883 EST\_ssal\_evd\_11114 ssalevd thymus Salmo salar cDNA Salmo salar cDNA clone ssal\_evd\_513\_268\_rev 5', mRNA sequence  
c gi|117434281|gb|EG766504.1|EG766504 EST\_ssal\_evd\_42637 ssalevd thymus Salmo salar cDNA Salmo salar cDNA clone ssal\_evd\_557\_067\_rev 5', mRNA sequence  
d gi|117471437|gb|EG803656.1|EG803656 EST\_ssal\_evd\_52964 ssalevd thymus Salmo salar cDNA Salmo salar cDNA clone ssal\_evd\_571\_148\_fwd 3', mRNA sequence  
e gi|117471448|gb|EG803667.1|EG803667 EST\_ssal\_evd\_52965 ssalevd thymus Salmo salar cDNA Salmo salar cDNA clone ssal\_evd\_571\_148\_rev 5', mRNA sequence  
f gi|117447230|gb|EG779449.1|EG779449 EST\_ssal\_evd\_36287 ssalevd thymus Salmo salar cDNA Salmo salar cDNA clone ssal\_evd\_548\_169\_rev 5', mRNA sequence  
g gi|117439741|gb|EG771964.1|EG771964 EST\_ssal\_evd\_47552 ssalevd thymus Salmo salar cDNA Salmo salar cDNA clone ssal\_evd\_563\_346\_rev 5', mRNA sequence  
h gi|117439740|gb|EG771963.1|EG771963 EST\_ssal\_evd\_47551 ssalevd thymus Salmo salar cDNA Salmo salar cDNA clone ssal\_evd\_563\_346\_fwd 3', mRNA sequence  
i gi|117472147|gb|EG804366.1|EG804366 EST\_ssal\_evd\_1449 ssalevd thymus Salmo salar cDNA Salmo salar cDNA clone ssal\_evd\_006\_188\_rev 5', mRNA sequence  
j gi|117472078|gb|EG804297.1|EG804297 EST\_ssal\_evd\_1387 ssalevd thymus Salmo salar cDNA Salmo salar cDNA clone ssal\_evd\_006\_138\_rev 5', mRNA sequence  
k gi|117434122|gb|EG766345.1|EG766345 EST\_ssal\_evd\_42494 ssalevd thymus Salmo salar cDNA Salmo salar cDNA clone ssal\_evd\_556\_369\_rev 5', mRNA sequence  
l gi|24352678|gb|CA046508.1|CA046508 ssalbrh015095 head Salmo salar cDNA, mRNA sequence  
m gi|118193302|gb|EH034337.1|EH034337 Ss\_Fwd2\_12G01\_T3 Forward subtracted library from fast muscle of Salmo salar Salmo salar cDNA clone Ss\_Fwd2\_12G01, mRNA sequence  
n gi|117456534|gb|EG788753.1|EG788753 EST\_ssal\_evd\_9198 ssalevd thymus Salmo salar cDNA Salmo salar cDNA clone ssal\_evd\_511\_019\_rev 5', mRNA sequence  
o gi|117447987|gb|EG780206.1|EG780206 EST\_ssal\_evd\_36970 ssalevd thymus Salmo salar cDNA Salmo salar cDNA clone ssal\_evd\_549\_135\_fwd 3', mRNA sequence  
p gi|117478734|gb|EG810951.1|EG810951 EST\_ssal\_evd\_31176 ssalevd thymus Salmo salar cDNA Salmo salar cDNA clone ssal\_evd\_541\_071\_rev 5', mRNA sequence  
q gi|117444193|gb|EG776416.1|EG776416 EST\_ssal\_evd\_51559 ssalevd thymus Salmo salar cDNA Salmo salar cDNA clone ssal\_evd\_569\_182\_rev 5', mRNA sequence  
r gi|117437255|gb|EG769478.1|EG769478 EST\_ssal\_evd\_45314 ssalevd thymus Salmo salar cDNA Salmo salar cDNA clone ssal\_evd\_560\_321\_rev 5', mRNA sequence  
s gi|117479662|gb|EG811879.1|EG811879 EST\_ssal\_evd\_32010 ssalevd thymus Salmo salar cDNA Salmo salar cDNA clone ssal\_evd\_542\_182\_rev 5', mRNA sequence  
t gi|117449078|gb|EG781297.1|EG781297 EST\_ssal\_evd\_37952 ssalevd thymus Salmo salar cDNA Salmo salar cDNA clone ssal\_evd\_550\_262\_rev 5', mRNA sequence  
u gi|117457881|gb|EG790100.1|EG790100 EST\_ssal\_evd\_53544 ssalevd thymus Salmo salar cDNA Salmo salar cDNA clone ssal\_evd\_572\_063\_rev 5', mRNA sequence  
v gi|117483564|gb|EG815781.1|EG815781 EST\_ssal\_evd\_35523 ssalevd thymus Salmo salar cDNA Salmo salar cDNA clone ssal\_evd\_547\_145\_rev 5', mRNA sequence  
w gi|117475899|gb|EG808118.1|EG808118 EST\_ssal\_evd\_28626 ssalevd thymus Salmo salar cDNA Salmo salar cDNA clone ssal\_evd\_537\_185\_rev 5', mRNA sequence  
x gi|117436018|gb|EG768241.1|EG768241 EST\_ssal\_evd\_44200 ssalevd thymus Salmo salar cDNA Salmo salar cDNA clone ssal\_evd\_559\_127\_rev 5', mRNA sequence  
y gi|117436813|gb|EG769036.1|EG769036 EST\_ssal\_evd\_44916 ssalevd thymus Salmo salar cDNA Salmo salar cDNA clone ssal\_evd\_560\_115\_rev 5', mRNA sequence  
z gi|117479953|gb|EG812170.1|EG812170 EST\_ssal\_evd\_32273 ssalevd thymus Salmo salar cDNA Salmo salar cDNA clone ssal\_evd\_542\_334\_rev 5', mRNA sequence  
A gi|117437928|gb|EG770151.1|EG770151 EST\_ssal\_evd\_45920 ssalevd thymus Salmo salar cDNA Salmo salar cDNA clone ssal\_evd\_561\_258\_rev 5', mRNA sequence  
B gi|117450634|gb|EG782853.1|EG782853 EST\_ssal\_evd\_39352 ssalevd thymus Salmo salar cDNA Salmo salar cDNA clone ssal\_evd\_552\_254\_rev 5', mRNA sequence  
C gi|117491577|gb|EG823794.1|EG823794 EST\_ssal\_evd\_25396 ssalevd thymus Salmo salar cDNA Salmo salar cDNA clone ssal\_evd\_533\_015\_rev 5', mRNA sequence  
D gi|117466211|gb|EG798430.1|EG798430 EST\_ssal\_evd\_55370 ssalevd thymus Salmo salar cDNA Salmo salar cDNA clone ssal\_evd\_574\_248\_rev 5', mRNA sequence  
E gi|117438716|gb|EG770939.1|EG770939 EST\_ssal\_evd\_46629 ssalevd thymus Salmo salar cDNA Salmo salar cDNA clone ssal\_evd\_562\_247\_rev 5', mRNA sequence  
F gi|117443620|gb|EG775843.1|EG775843 EST\_ssal\_evd\_51044 ssalevd thymus Salmo salar cDNA Salmo salar cDNA clone ssal\_evd\_568\_289\_rev 5', mRNA sequence  
G gi|117461657|gb|EG793876.1|EG793876 EST\_ssal\_evd\_13809 ssalevd thymus Salmo salar cDNA Salmo salar cDNA clone ssal\_evd\_517\_134\_rev 5', mRNA sequence  
H gi|117451008|gb|EG783227.1|EG783227 EST\_ssal\_evd\_39688 ssalevd thymus Salmo salar cDNA Salmo salar cDNA clone ssal\_evd\_553\_046\_rev 5', mRNA sequence  
I gi|117489497|gb|EG821714.1|EG821714 EST\_ssal\_evd\_23523 ssalevd thymus Salmo salar cDNA Salmo salar cDNA clone ssal\_evd\_530\_198\_rev 5', mRNA sequence  
J gi|117476370|gb|EG808589.1|EG808589 EST\_ssal\_evd\_17944 ssalevd thymus Salmo salar cDNA Salmo salar cDNA clone ssal\_evd\_522\_376\_rev 5', mRNA sequence  
K gi|117544387|gb|EG875832.1|EG875832 EST\_ssal\_eve\_19609 ssaleve thyroid Salmo salar cDNA Salmo salar cDNA clone ssal\_eve\_526\_262\_rev 5', mRNA sequence  
L gi|117468562|gb|EG800781.1|EG800781 EST\_ssal\_evd\_57485 ssalevd thymus Salmo salar cDNA Salmo salar cDNA clone ssal\_evd\_577\_202\_rev 5', mRNA sequence  
M gi|117464986|gb|EG797205.1|EG797205 EST\_ssal\_evd\_54266 ssalevd thymus Salmo salar cDNA Salmo salar cDNA clone ssal\_evd\_573\_055\_rev 5', mRNA sequence  
N gi|84970380|gb|DW468781.1|DW468781 SGP286662 Atlantic salmon Testis cDNA library Salmo salar cDNA clone MG4-1756 5', mRNA sequence  
O gi|45327106|gb|CK897373.1|CK897373 SGP154535 Atlantic salmon Testis cDNA library Salmo salar cDNA clone MG5-0112 5', mRNA sequence  
P gi|45328376|gb|CK898643.1|CK898643 SGP162218 Atlantic salmon Testis cDNA library Salmo salar cDNA clone MG4-0844 5', mRNA sequence  
Q gi|45328788|gb|CK899055.1|CK899055 SGP162643 Atlantic salmon Testis cDNA library Salmo salar cDNA clone MG4-1377 5', mRNA sequence  
R gi|84972980|gb|DW471381.1|DW471381 SGP315097 Atlantic salmon Testis cDNA library Salmo salar cDNA clone MG4-4864 5', mRNA sequence  
S gi|84972630|gb|DW471031.1|DW471031 SGP313663 Atlantic salmon Testis cDNA library Salmo salar cDNA clone MG4-4460 5', mRNA sequence  
T gi|84971062|gb|DW469463.1|DW469463 SGP297800 Atlantic salmon Testis cDNA library Salmo salar cDNA clone MG4-2582 5', mRNA sequence  
U gi|45327633|gb|CK897900.1|CK897900 SGP155073 Atlantic salmon Testis cDNA library Salmo salar cDNA clone MG5-0711 5', mRNA sequence  
V gi|45328995|gb|CK899262.1|CK899262 SGP162851 Atlantic salmon Testis cDNA library Salmo salar cDNA clone MG4-1630 5', mRNA sequence  
W gi|84971479|gb|DW469880.1|DW469880 SGP298759 Atlantic salmon Testis cDNA library Salmo salar cDNA clone MG4-3081 5', mRNA sequence  
X gi|84972139|gb|DW470540.1|DW470540 SGP311831 Atlantic salmon Testis cDNA library Salmo salar cDNA clone MG4-3874 5', mRNA sequence  
Y gi|45328094|gb|CK898361.1|CK898361 SGP161932 Atlantic salmon Testis cDNA library Salmo salar cDNA clone MG4-0492 5', mRNA sequence  
Z gi|84970487|gb|DW468888.1|DW468888 SGP286769 Atlantic salmon Testis cDNA library Salmo salar cDNA clone MG4-1886 5', mRNA sequence

13 SNPs detected

A B C D E F G H I J K L M N O P Q R S T U V W X Y Z a b c d e f g h i j k l m n o p q r s t u v w x y z A B C D E F G H I J K L M N O P Q R S T U V W X Y Z  cosegregation weighted

477 A G A A G G A G A G G A A A G G A A A . . A G G G A A G . . . . G G G G G . . . . . G G . G G G G A A . A A G G G G G G G G G G G . . . . . . . . . . . . .   1/13 5.13
506 - - - - - - - - - - - - - - - - - - - . . - A - - - - A . . . . A A - A A . . . . . A - . - - - - - - - - - A A A A A A A A A A A . . . . . . . . . . . . .   10/13 52.27
507 - - - - - - - - - - - - - - - - - - - . . - G - - - - G . . . . G G - G G . . . . . G - . - - - - - - - - - G G G G G G G G G G G . . . . . . . . . . . . .   10/13 52.27
508 - - - - - - - - - - - - - - - - - - - . . - A - - - - A . . . . A A - A A . . . . . A - . - - - - - - - - - A A A A A A A A A A A . . . . . . . . . . . . .   10/13 52.27
509 - - - - - - - - - - - - - - - - - - - . . - G - - - - G . . . . G G - G G . . . . . G - . - - - - - - - - - G G G G G G G G G G G . . . . . . . . . . . . .   10/13 52.27
510 - - - - - - - - - - - - - - - - - - - . . - A - - - - A . . . . A A - A A . . . . . A - . - - - - - - - - - A A A A A A A A A A A . . . . . . . . . . . . .   10/13 52.27
511 - - - - - - - - - - - - - - - - - - - . . - A - - - - A . . . . A A - A A . . . . . A - . - - - - - - - - - A A A A A A A A A A A . . . . . . . . . . . . .   10/13 52.27
512 - - - - - - - - - - - - - - - - - - - . . - T - - - - T . . . . T T - T T . . . . . T - . - - - - - - - - - T T T T T T T T T T T . . . . . . . . . . . . .   10/13 52.27
513 - - - - - - - - - - - - - - - - - - - . . - G - - - - G . . . . G G - G G . . . . . G - . - - - - - - - - - G G G G G G G G G G G . . . . . . . . . . . . .   10/13 52.27
514 - - - - - - - - - - - - - - - - - - - . . - G - - - - G . . . . G G - G G . . . . . G - . - - - - - - - - - G G G G G G G G G G G . . . . . . . . . . . . .   10/13 52.27
515 - - - - - - - - - - - - - - - - - - - . . - T - - - - T . . . . T T - T T . . . . . T - . - - - - - - - - - T T T T T T T T T T T . . . . . . . . . . . . .   10/13 52.27
554 C C C C C C C C C C C C C C C C C C C . . C C C T C C C . C C T C C T T C . . C C C C C C C T C C C T T C C C C C C C C T C C C C . . . . . . . . . . . . .   1/13 5.92
632 . A A A A - A - A A A A A A - A A A A . A A A - A A A A . A A A A A A A A . . A A A A A A - - A A A A A A A A A A A A A A A - A A A A . . . . . . . . . . .   1/13 6.11
