# Supplementary material for: Discovery and application of insertion-deletion (INDEL) polymorphisms for QTL mapping of early life-history traits in Atlantic salmon
Source: BMC Genomics. 2010 Mar 8;11:156. doi: 10.1186/1471-2164-11-156 (PMC2838853; doi:10.1186/1471-2164-11-156)
Supplement: Additional file 2 — Information on developed 76 locus single-run INDEL panel in Atlantic salmon. Information on fluorescence labeling, primer concentrations, PCR pooling and links to alignments, INDEL motifs and GENESCAN (Burge and Karlin 1997) predictions of genes/exons are available in html format. [file 1471-2164-11-156-S2.ZIP › Additionalfile2/snpsummary1329.html]

```
Cluster 181 Contig 1

prev  Summary    Contig List  next
```

Size of Consensus sequence = 1371

Number of sequences = 41

Minimum redundancy = 6

Key

A gi|85040723|gb|DW568901.1|DW568901 EST\_ssal\_rgb2\_33320 rgb2 Salmo salar cDNA clone ssal\_rgb2\_553\_372\_fwd 3', mRNA sequence  
B gi|117297677|gb|EG647490.1|EG647490 SGP258516 Atlantic salmon normalized Brain cDNA library Salmo salar cDNA clone NB1-0613 5', mRNA sequence  
C gi|117547287|gb|EG878732.1|EG878732 EST\_ssal\_eve\_16973 ssaleve thyroid Salmo salar cDNA Salmo salar cDNA clone ssal\_eve\_523\_028\_fwd 3', mRNA sequence  
D gi|117514283|gb|EG846042.1|EG846042 EST\_ssal\_eve\_49091 ssaleve thyroid Salmo salar cDNA Salmo salar cDNA clone ssal\_eve\_566\_325\_fwd 3', mRNA sequence  
E gi|117505415|gb|EG837174.1|EG837174 EST\_ssal\_eve\_11748 ssaleve thyroid Salmo salar cDNA Salmo salar cDNA clone ssal\_eve\_515\_373\_fwd 3', mRNA sequence  
F gi|117452026|gb|EG784245.1|EG784245 EST\_ssal\_evd\_2618 ssalevd thymus Salmo salar cDNA Salmo salar cDNA clone ssal\_evd\_502\_045\_fwd 3', mRNA sequence  
G gi|57121933|gb|CX353374.1|CX353374 ssalrgb515284\_rev\_0 mixed\_tissue Salmo salar cDNA, mRNA sequence  
H gi|117469643|gb|EG801862.1|EG801862 EST\_ssal\_evd\_58459 ssalevd thymus Salmo salar cDNA Salmo salar cDNA clone ssal\_evd\_578\_342\_fwd 3', mRNA sequence  
I gi|117504957|gb|EG836716.1|EG836716 EST\_ssal\_eve\_11335 ssaleve thyroid Salmo salar cDNA Salmo salar cDNA clone ssal\_eve\_515\_153\_fwd 3', mRNA sequence  
J gi|85051411|gb|DW579589.1|DW579589 EST\_ssal\_rgb2\_44008 rgb2 Salmo salar cDNA clone ssal\_rgb2\_571\_118\_rev 5', mRNA sequence  
K gi|117861478|gb|EG934174.1|EG934174 EST\_ssal\_evf\_16907 ssalevf mixed\_tissue Salmo salar cDNA Salmo salar cDNA clone ssal\_evf\_521\_051\_fwd 3', mRNA sequence  
L gi|117545644|gb|EG877089.1|EG877089 EST\_ssal\_eve\_20740 ssaleve thyroid Salmo salar cDNA Salmo salar cDNA clone ssal\_eve\_528\_076\_fwd 3', mRNA sequence  
M gi|85017586|gb|DW546242.1|DW546242 EST\_ssal\_rgb2\_10661 rgb2 Salmo salar cDNA clone ssal\_rgb2\_518\_049\_rev 5', mRNA sequence  
N gi|85029088|gb|DW557744.1|DW557744 EST\_ssal\_rgb2\_22163 rgb2 Salmo salar cDNA clone ssal\_rgb2\_536\_012\_rev 5', mRNA sequence  
O gi|117835463|gb|EG908159.1|EG908159 EST\_ssal\_evf\_10501 ssalevf mixed\_tissue Salmo salar cDNA Salmo salar cDNA clone ssal\_evf\_512\_164\_fwd 3', mRNA sequence  
P gi|29328906|gb|CB517680.1|CB517680 ssalrgb501219\_rev mixed\_tissue Salmo salar cDNA, mRNA sequence  
Q gi|117298480|gb|EG648293.1|EG648293 SGP317882 Atlantic salmon normalized Brain cDNA library Salmo salar cDNA clone BRAIN2-F4 5', mRNA sequence  
R gi|116041015|gb|EG355050.1|EG355050 Ss\_Gill\_93A07\_M13 Gill SSH library Salmo salar cDNA clone Ss\_Gill\_93A07 5', mRNA sequence  
S gi|119020761|gb|EG355050.2|EG355050 Ss\_Gill\_93A07\_M13 Gill SSH library Salmo salar cDNA clone Ss\_Gill\_93A07 5', mRNA sequence  
T gi|45315507|gb|CK885876.1|CK885876 SGP166263 Atlantic salmon Intestine cDNA library Salmo salar cDNA clone T5-0306 5', mRNA sequence  
U gi|117457976|gb|EG790195.1|EG790195 EST\_ssal\_evd\_10496 ssalevd thymus Salmo salar cDNA Salmo salar cDNA clone ssal\_evd\_512\_319\_fwd 3', mRNA sequence  
V gi|117469642|gb|EG801861.1|EG801861 EST\_ssal\_evd\_58458 ssalevd thymus Salmo salar cDNA Salmo salar cDNA clone ssal\_evd\_578\_342\_rev 5', mRNA sequence  
W gi|85024917|gb|DW553573.1|DW553573 EST\_ssal\_rgb2\_17992 rgb2 Salmo salar cDNA clone ssal\_rgb2\_529\_201\_rev 5', mRNA sequence  
X gi|24394566|gb|CA064323.1|CA064323 ssalrgb501219 mixed\_tissue Salmo salar cDNA, mRNA sequence  
Y gi|24384853|gb|CA054610.1|CA054610 ssalrgb523243 mixed\_tissue Salmo salar cDNA, mRNA sequence  
Z gi|24383355|gb|CA053112.1|CA053112 ssalrgb515284 mixed\_tissue Salmo salar cDNA, mRNA sequence  
a gi|117504956|gb|EG836715.1|EG836715 EST\_ssal\_eve\_11334 ssaleve thyroid Salmo salar cDNA Salmo salar cDNA clone ssal\_eve\_515\_153\_rev 5', mRNA sequence  
b gi|117514294|gb|EG846053.1|EG846053 EST\_ssal\_eve\_49092 ssaleve thyroid Salmo salar cDNA Salmo salar cDNA clone ssal\_eve\_566\_325\_rev 5', mRNA sequence  
c gi|117861589|gb|EG934285.1|EG934285 EST\_ssal\_evf\_16908 ssalevf mixed\_tissue Salmo salar cDNA Salmo salar cDNA clone ssal\_evf\_521\_051\_rev 5', mRNA sequence  
d gi|117535654|gb|EG867099.1|EG867099 EST\_ssal\_eve\_28581 ssaleve thyroid Salmo salar cDNA Salmo salar cDNA clone ssal\_eve\_538\_321\_rev 5', mRNA sequence  
e gi|117457975|gb|EG790194.1|EG790194 EST\_ssal\_evd\_10495 ssalevd thymus Salmo salar cDNA Salmo salar cDNA clone ssal\_evd\_512\_319\_rev 5', mRNA sequence  
f gi|117835462|gb|EG908158.1|EG908158 EST\_ssal\_evf\_10500 ssalevf mixed\_tissue Salmo salar cDNA Salmo salar cDNA clone ssal\_evf\_512\_164\_rev 5', mRNA sequence  
g gi|117535653|gb|EG867098.1|EG867098 EST\_ssal\_eve\_28580 ssaleve thyroid Salmo salar cDNA Salmo salar cDNA clone ssal\_eve\_538\_321\_fwd 3', mRNA sequence  
h gi|117452037|gb|EG784256.1|EG784256 EST\_ssal\_evd\_2619 ssalevd thymus Salmo salar cDNA Salmo salar cDNA clone ssal\_evd\_502\_045\_rev 5', mRNA sequence  
i gi|117530574|gb|EG862030.1|EG862030 EST\_ssal\_eve\_55119 ssaleve thyroid Salmo salar cDNA Salmo salar cDNA clone ssal\_eve\_575\_051\_rev 5', mRNA sequence  
j gi|117547176|gb|EG878621.1|EG878621 EST\_ssal\_eve\_16972 ssaleve thyroid Salmo salar cDNA Salmo salar cDNA clone ssal\_eve\_523\_028\_rev 5', mRNA sequence  
k gi|85029089|gb|DW557745.1|DW557745 EST\_ssal\_rgb2\_22164 rgb2 Salmo salar cDNA clone ssal\_rgb2\_536\_012\_fwd 3', mRNA sequence  
l gi|85051410|gb|DW579588.1|DW579588 EST\_ssal\_rgb2\_44007 rgb2 Salmo salar cDNA clone ssal\_rgb2\_571\_118\_fwd 3', mRNA sequence  
m gi|85028840|gb|DW557496.1|DW557496 EST\_ssal\_rgb2\_21915 rgb2 Salmo salar cDNA clone ssal\_rgb2\_535\_245\_fwd 3', mRNA sequence  
n gi|117505416|gb|EG837175.1|EG837175 EST\_ssal\_eve\_11749 ssaleve thyroid Salmo salar cDNA Salmo salar cDNA clone ssal\_eve\_515\_373\_rev 5', mRNA sequence  
o gi|117864711|gb|EG937407.1|EG937407 EST\_ssal\_evf\_17167 ssalevf mixed\_tissue Salmo salar cDNA Salmo salar cDNA clone ssal\_evf\_521\_186\_rev 5', mRNA sequence

4 SNPs detected

A B C D E F G H I J K L M N O P Q R S T U V W X Y Z a b c d e f g h i j k l m n o  cosegregation weighted

584 . . C C C C T C C T C C T T C T C . . . C . T . . . . . . . . . . . . . . . . . .   4/4 41.46
1121 . . . . . . . . . . . . . . . . . . . . - - C C C C - - - - - - - C C C C C C C C   4/4 51.22
1122 . . . . . . . . . . . . . . . . . . . . - - A A A A - - - - - - - A A A A A A A A   4/4 51.22
1311 . . . . . . . . . . . . . . . . . . . . . . C C . C T T T T T T T T T T C C C T T   4/4 43.90
