# Supplementary material for: Discovery and application of insertion-deletion (INDEL) polymorphisms for QTL mapping of early life-history traits in Atlantic salmon
Source: BMC Genomics. 2010 Mar 8;11:156. doi: 10.1186/1471-2164-11-156 (PMC2838853; doi:10.1186/1471-2164-11-156)
Supplement: Additional file 2 — Information on developed 76 locus single-run INDEL panel in Atlantic salmon. Information on fluorescence labeling, primer concentrations, PCR pooling and links to alignments, INDEL motifs and GENESCAN (Burge and Karlin 1997) predictions of genes/exons are available in html format. [file 1471-2164-11-156-S2.ZIP › Additionalfile2/snpsummary1338.html]

```
Cluster 183 Contig 2

prev  Summary    Contig List  next
```

Size of Consensus sequence = 1946

Number of sequences = 58

Minimum redundancy = 6

Key

A gi|117467993|gb|EG800212.1|EG800212 EST\_ssal\_evd\_52654 ssalevd thymus Salmo salar cDNA Salmo salar cDNA clone ssal\_evd\_570\_371\_rev 5', mRNA sequence  
B gi|117468004|gb|EG800223.1|EG800223 EST\_ssal\_evd\_52655 ssalevd thymus Salmo salar cDNA Salmo salar cDNA clone ssal\_evd\_570\_371\_fwd 3', mRNA sequence  
C gi|24339947|gb|CA039417.1|CA039417 ssalwha004078 whole Salmo salar cDNA, mRNA sequence  
D gi|118419824|gb|AM412206.1|AM412206 AM412206 Salmo salar after live bacterial vaccine Salmo salar cDNA clone kid\_aki\_05D07, mRNA sequence  
E gi|117435840|gb|EG768063.1|EG768063 EST\_ssal\_evd\_44041 ssalevd thymus Salmo salar cDNA Salmo salar cDNA clone ssal\_evd\_559\_040\_rev 5', mRNA sequence  
F gi|117849152|gb|EG921848.1|EG921848 EST\_ssal\_evf\_1019 ssalevf mixed\_tissue Salmo salar cDNA Salmo salar cDNA clone ssal\_evf\_005\_182\_rev 5', mRNA sequence  
G gi|85034002|gb|DW562658.1|DW562658 EST\_ssal\_rgb2\_27077 rgb2 Salmo salar cDNA clone ssal\_rgb2\_544\_003\_fwd 3', mRNA sequence  
H gi|117830723|gb|EG903419.1|EG903419 EST\_ssal\_evf\_41978 ssalevf mixed\_tissue Salmo salar cDNA Salmo salar cDNA clone ssal\_evf\_556\_022\_rev 5', mRNA sequence  
I gi|117844400|gb|EG917096.1|EG917096 EST\_ssal\_evf\_56284 ssalevf mixed\_tissue Salmo salar cDNA Salmo salar cDNA clone ssal\_evf\_575\_187\_rev 5', mRNA sequence  
J gi|117832394|gb|EG905090.1|EG905090 EST\_ssal\_evf\_7737 ssalevf mixed\_tissue Salmo salar cDNA Salmo salar cDNA clone ssal\_evf\_508\_260\_rev 5', mRNA sequence  
K gi|117526608|gb|EG858335.1|EG858335 EST\_ssal\_eve\_51793 ssaleve thyroid Salmo salar cDNA Salmo salar cDNA clone ssal\_eve\_570\_208\_rev 5', mRNA sequence  
L gi|117527752|gb|EG859393.1|EG859393 EST\_ssal\_eve\_52745 ssaleve thyroid Salmo salar cDNA Salmo salar cDNA clone ssal\_eve\_571\_325\_rev 5', mRNA sequence  
M gi|117824994|gb|EG897690.1|EG897690 EST\_ssal\_evf\_36822 ssalevf mixed\_tissue Salmo salar cDNA Salmo salar cDNA clone ssal\_evf\_549\_024\_rev 5', mRNA sequence  
N gi|117844390|gb|EG917086.1|EG917086 EST\_ssal\_evf\_56275 ssalevf mixed\_tissue Salmo salar cDNA Salmo salar cDNA clone ssal\_evf\_575\_182\_rev 5', mRNA sequence  
O gi|117458267|gb|EG790486.1|EG790486 EST\_ssal\_evd\_10757 ssalevd thymus Salmo salar cDNA Salmo salar cDNA clone ssal\_evd\_513\_075\_rev 5', mRNA sequence  
P gi|117565741|gb|EG886717.1|EG886717 EST\_ssal\_evf\_3306 ssalevf mixed\_tissue Salmo salar cDNA Salmo salar cDNA clone ssal\_evf\_502\_276\_fwd 3', mRNA sequence  
Q gi|117565753|gb|EG886729.1|EG886729 EST\_ssal\_evf\_3307 ssalevf mixed\_tissue Salmo salar cDNA Salmo salar cDNA clone ssal\_evf\_502\_276\_rev 5', mRNA sequence  
R gi|117569856|gb|EG890832.1|EG890832 EST\_ssal\_evf\_48650 ssalevf mixed\_tissue Salmo salar cDNA Salmo salar cDNA clone ssal\_evf\_565\_030\_rev 5', mRNA sequence  
S gi|75981017|gb|AM041648.1|AM041648 AM041648 pGemT-easy Atlantic salmon gill subtracted library (Aeromonas salmonicda infected salmon subtracted with uninfected) Salmo salar cDNA clone GT3H4, mRNA sequence  
T gi|117438669|gb|EG770892.1|EG770892 EST\_ssal\_evd\_46586 ssalevd thymus Salmo salar cDNA Salmo salar cDNA clone ssal\_evd\_562\_223\_rev 5', mRNA sequence  
U gi|117500666|gb|EG832692.1|EG832692 EST\_ssal\_eve\_44716 ssaleve thyroid Salmo salar cDNA Salmo salar cDNA clone ssal\_eve\_560\_307\_rev 5', mRNA sequence  
V gi|117540890|gb|EG872335.1|EG872335 EST\_ssal\_eve\_33294 ssaleve thyroid Salmo salar cDNA Salmo salar cDNA clone ssal\_eve\_545\_075\_rev 5', mRNA sequence  
W gi|117844401|gb|EG917097.1|EG917097 EST\_ssal\_evf\_56285 ssalevf mixed\_tissue Salmo salar cDNA Salmo salar cDNA clone ssal\_evf\_575\_187\_fwd 3', mRNA sequence  
X gi|117445313|gb|EG777536.1|EG777536 EST\_ssal\_evd\_4165 ssalevd thymus Salmo salar cDNA Salmo salar cDNA clone ssal\_evd\_504\_081\_rev 5', mRNA sequence  
Y gi|117445311|gb|EG777534.1|EG777534 EST\_ssal\_evd\_4164 ssalevd thymus Salmo salar cDNA Salmo salar cDNA clone ssal\_evd\_504\_081\_fwd 3', mRNA sequence  
Z gi|84987528|gb|DW537878.1|DW537878 EST\_ssal\_rgb2\_2297 rgb2 Salmo salar cDNA clone ssal\_rgb2\_504\_286\_fwd 3', mRNA sequence  
a gi|117435839|gb|EG768062.1|EG768062 EST\_ssal\_evd\_44040 ssalevd thymus Salmo salar cDNA Salmo salar cDNA clone ssal\_evd\_559\_040\_fwd 3', mRNA sequence  
b gi|117548355|gb|EG879800.1|EG879800 EST\_ssal\_eve\_23180 ssaleve thyroid Salmo salar cDNA Salmo salar cDNA clone ssal\_eve\_531\_223\_rev 5', mRNA sequence  
c gi|89848542|gb|DY704665.1|DY704665 EST\_ssal\_rgb2\_60404 ssalrgb2 mixed\_tissue Salmo salar cDNA Salmo salar cDNA clone ssal\_rgb2\_597\_109\_fwd 3', mRNA sequence  
d gi|117491097|gb|EG823314.1|EG823314 EST\_ssal\_evd\_24963 ssalevd thymus Salmo salar cDNA Salmo salar cDNA clone ssal\_evd\_532\_176\_rev 5', mRNA sequence  
e gi|24382062|gb|CA051819.1|CA051819 ssalrga501158 mixed\_tissue Salmo salar cDNA, mRNA sequence  
f gi|117822120|gb|EG894816.1|EG894816 EST\_ssal\_evf\_4035 ssalevf mixed\_tissue Salmo salar cDNA Salmo salar cDNA clone ssal\_evf\_503\_269\_rev 5', mRNA sequence  
g gi|117509388|gb|EG841147.1|EG841147 EST\_ssal\_eve\_3925 ssaleve thyroid Salmo salar cDNA Salmo salar cDNA clone ssal\_eve\_504\_132\_rev 5', mRNA sequence  
h gi|117830722|gb|EG903418.1|EG903418 EST\_ssal\_evf\_41977 ssalevf mixed\_tissue Salmo salar cDNA Salmo salar cDNA clone ssal\_evf\_556\_022\_fwd 3', mRNA sequence  
i gi|29325474|gb|CB514248.1|CB514248 ssalrgb548307 mixed\_tissue Salmo salar cDNA, mRNA sequence  
j gi|117569855|gb|EG890831.1|EG890831 EST\_ssal\_evf\_48649 ssalevf mixed\_tissue Salmo salar cDNA Salmo salar cDNA clone ssal\_evf\_565\_030\_fwd 3', mRNA sequence  
k gi|24394050|gb|CA063807.1|CA063807 ssalrgb530059 mixed\_tissue Salmo salar cDNA, mRNA sequence  
l gi|84973447|gb|DW471848.1|DW471848 SGP285965 Atlantic salmon Spleen cDNA library Salmo salar cDNA clone MI5-1801 5', mRNA sequence  
m gi|117491096|gb|EG823313.1|EG823313 EST\_ssal\_evd\_24962 ssalevd thymus Salmo salar cDNA Salmo salar cDNA clone ssal\_evd\_532\_176\_fwd 3', mRNA sequence  
n gi|117822131|gb|EG894827.1|EG894827 EST\_ssal\_evf\_4036 ssalevf mixed\_tissue Salmo salar cDNA Salmo salar cDNA clone ssal\_evf\_503\_269\_fwd 3', mRNA sequence  
o gi|117458266|gb|EG790485.1|EG790485 EST\_ssal\_evd\_10756 ssalevd thymus Salmo salar cDNA Salmo salar cDNA clone ssal\_evd\_513\_075\_fwd 3', mRNA sequence  
p gi|117844389|gb|EG917085.1|EG917085 EST\_ssal\_evf\_56274 ssalevf mixed\_tissue Salmo salar cDNA Salmo salar cDNA clone ssal\_evf\_575\_182\_fwd 3', mRNA sequence  
q gi|89848264|gb|DY704387.1|DY704387 EST\_ssal\_rgb2\_60126 ssalrgb2 mixed\_tissue Salmo salar cDNA Salmo salar cDNA clone ssal\_rgb2\_596\_322\_rev 5', mRNA sequence  
r gi|85034003|gb|DW562659.1|DW562659 EST\_ssal\_rgb2\_27078 rgb2 Salmo salar cDNA clone ssal\_rgb2\_544\_003\_rev 5', mRNA sequence  
s gi|117438670|gb|EG770893.1|EG770893 EST\_ssal\_evd\_46587 ssalevd thymus Salmo salar cDNA Salmo salar cDNA clone ssal\_evd\_562\_223\_fwd 3', mRNA sequence  
t gi|117548356|gb|EG879801.1|EG879801 EST\_ssal\_eve\_23181 ssaleve thyroid Salmo salar cDNA Salmo salar cDNA clone ssal\_eve\_531\_223\_fwd 3', mRNA sequence  
u gi|117500667|gb|EG832693.1|EG832693 EST\_ssal\_eve\_44717 ssaleve thyroid Salmo salar cDNA Salmo salar cDNA clone ssal\_eve\_560\_307\_fwd 3', mRNA sequence  
v gi|117849153|gb|EG921849.1|EG921849 EST\_ssal\_evf\_1020 ssalevf mixed\_tissue Salmo salar cDNA Salmo salar cDNA clone ssal\_evf\_005\_182\_fwd 3', mRNA sequence  
w gi|117529389|gb|EG861030.1|EG861030 EST\_ssal\_eve\_54219 ssaleve thyroid Salmo salar cDNA Salmo salar cDNA clone ssal\_eve\_573\_332\_fwd 3', mRNA sequence  
x gi|117509387|gb|EG841146.1|EG841146 EST\_ssal\_eve\_3924 ssaleve thyroid Salmo salar cDNA Salmo salar cDNA clone ssal\_eve\_504\_132\_fwd 3', mRNA sequence  
y gi|117832395|gb|EG905091.1|EG905091 EST\_ssal\_evf\_7738 ssalevf mixed\_tissue Salmo salar cDNA Salmo salar cDNA clone ssal\_evf\_508\_260\_fwd 3', mRNA sequence  
z gi|84989865|gb|DW540215.1|DW540215 EST\_ssal\_rgb2\_4634 rgb2 Salmo salar cDNA clone ssal\_rgb2\_508\_332\_rev 5', mRNA sequence  
A gi|89848541|gb|DY704664.1|DY704664 EST\_ssal\_rgb2\_60403 ssalrgb2 mixed\_tissue Salmo salar cDNA Salmo salar cDNA clone ssal\_rgb2\_597\_109\_rev 5', mRNA sequence  
B gi|117526609|gb|EG858336.1|EG858336 EST\_ssal\_eve\_51794 ssaleve thyroid Salmo salar cDNA Salmo salar cDNA clone ssal\_eve\_570\_208\_fwd 3', mRNA sequence  
C gi|84987192|gb|DW537542.1|DW537542 EST\_ssal\_rgb2\_1961 rgb2 Salmo salar cDNA clone ssal\_rgb2\_504\_095\_rev 5', mRNA sequence  
D gi|29328554|gb|CB517328.1|CB517328 ssalrgb530059\_rev mixed\_tissue Salmo salar cDNA, mRNA sequence  
E gi|117540889|gb|EG872334.1|EG872334 EST\_ssal\_eve\_33293 ssaleve thyroid Salmo salar cDNA Salmo salar cDNA clone ssal\_eve\_545\_075\_fwd 3', mRNA sequence  
F gi|117527753|gb|EG859394.1|EG859394 EST\_ssal\_eve\_52746 ssaleve thyroid Salmo salar cDNA Salmo salar cDNA clone ssal\_eve\_571\_325\_fwd 3', mRNA sequence

7 SNPs detected

A B C D E F G H I J K L M N O P Q R S T U V W X Y Z a b c d e f g h i j k l m n o p q r s t u v w x y z A B C D E F  cosegregation weighted

208 - - T - T T - T T T T - - - - - - - T T T T . - - - T T . T - T T T T . T . T . . . . . . . . . . . . . . . . . . .   6/7 51.72
209 - - T - T T - T T T T - - - - - - - T T T T . - - - T T . T - T T T T . T . T . . . . . . . . . . . . . . . . . . .   6/7 51.72
210 - - A - A A - A A A A - - - - - - - A A A A . - - - A A . A - A A A A . A . A . . . . . . . . . . . . . . . . . . .   6/7 51.72
211 - - T - T T - T T T T - - - - - - - T T T T . - - - T T . T - T T T T . T . T . . . . . . . . . . . . . . . . . . .   6/7 51.72
212 - - T - T T - T T T T - - - - - - - T T T T . - - - T T . T - T T T T . T . T . . . . . . . . . . . . . . . . . . .   6/7 51.72
213 - - G - G G - G G G G - - - - - - - G G G G . - - - G G . G - G G G G . G . G . . . . . . . . . . . . . . . . . . .   6/7 51.72
436 . . C C C C A C C C C A A A A A A A C C C C C A A A C C C C A C C C C A C C C C A . . . . . . . . . . . . . . . . .   1/7 9.61
