# Supplementary material for: Discovery and application of insertion-deletion (INDEL) polymorphisms for QTL mapping of early life-history traits in Atlantic salmon
Source: BMC Genomics. 2010 Mar 8;11:156. doi: 10.1186/1471-2164-11-156 (PMC2838853; doi:10.1186/1471-2164-11-156)
Supplement: Additional file 2 — Information on developed 76 locus single-run INDEL panel in Atlantic salmon. Information on fluorescence labeling, primer concentrations, PCR pooling and links to alignments, INDEL motifs and GENESCAN (Burge and Karlin 1997) predictions of genes/exons are available in html format. [file 1471-2164-11-156-S2.ZIP › Additionalfile2/snpsummary13526.html]

```
Cluster 5504 Contig 1

prev  Summary    Contig List  next
```

Size of Consensus sequence = 1216

Number of sequences = 8

Minimum redundancy = 3

Key

A gi|85034524|gb|DW563180.1|DW563180 EST\_ssal\_rgb2\_27599 rgb2 Salmo salar cDNA clone ssal\_rgb2\_544\_322\_fwd 3', mRNA sequence  
B gi|24351430|gb|CA045260.1|CA045260 ssalplnb505296 gut Salmo salar cDNA, mRNA sequence  
C gi|24354112|gb|CA047942.1|CA047942 ssalbrh009038 head Salmo salar cDNA, mRNA sequence  
D gi|117860196|gb|EG932892.1|EG932892 EST\_ssal\_evf\_34760 ssalevf mixed\_tissue Salmo salar cDNA Salmo salar cDNA clone ssal\_evf\_546\_100\_rev 5', mRNA sequence  
E gi|117536671|gb|EG868116.1|EG868116 EST\_ssal\_eve\_29497 ssaleve thyroid Salmo salar cDNA Salmo salar cDNA clone ssal\_eve\_540\_026\_rev 5', mRNA sequence  
F gi|117536672|gb|EG868117.1|EG868117 EST\_ssal\_eve\_29498 ssaleve thyroid Salmo salar cDNA Salmo salar cDNA clone ssal\_eve\_540\_026\_fwd 3', mRNA sequence  
G gi|117860197|gb|EG932893.1|EG932893 EST\_ssal\_evf\_34761 ssalevf mixed\_tissue Salmo salar cDNA Salmo salar cDNA clone ssal\_evf\_546\_100\_fwd 3', mRNA sequence  
H gi|118192853|gb|EH033888.1|EH033888 Ss\_Fwd2\_19E02\_T3 Forward subtracted library from fast muscle of Salmo salar Salmo salar cDNA clone Ss\_Fwd2\_19E02, mRNA sequence

4 SNPs detected

A B C D E F G H  cosegregation weighted

677 . G A A G G A .   4/4 75.00
678 . G T T G G T .   4/4 75.00
679 . - A A - - A .   4/4 75.00
680 . - A A - - A .   4/4 75.00
