# Supplementary material for: Discovery and application of insertion-deletion (INDEL) polymorphisms for QTL mapping of early life-history traits in Atlantic salmon
Source: BMC Genomics. 2010 Mar 8;11:156. doi: 10.1186/1471-2164-11-156 (PMC2838853; doi:10.1186/1471-2164-11-156)
Supplement: Additional file 2 — Information on developed 76 locus single-run INDEL panel in Atlantic salmon. Information on fluorescence labeling, primer concentrations, PCR pooling and links to alignments, INDEL motifs and GENESCAN (Burge and Karlin 1997) predictions of genes/exons are available in html format. [file 1471-2164-11-156-S2.ZIP › Additionalfile2/snpsummary13587.html]

```
Cluster 5543 Contig 2

prev  Summary    Contig List  next
```

Size of Consensus sequence = 776

Number of sequences = 6

Minimum redundancy = 2

Key

A gi|24343509|gb|CA042589.1|CA042589 ssalplnb501360 gut Salmo salar cDNA, mRNA sequence  
B gi|24352927|gb|CA046757.1|CA046757 ssalga005044 head Salmo salar cDNA, mRNA sequence  
C gi|24387317|gb|CA057074.1|CA057074 ssalrga512338 mixed\_tissue Salmo salar cDNA, mRNA sequence  
D gi|85021671|gb|DW550327.1|DW550327 EST\_ssal\_rgb2\_14746 rgb2 Salmo salar cDNA clone ssal\_rgb2\_524\_195\_fwd 3', mRNA sequence  
E gi|89830396|gb|DY692726.1|DY692726 EST\_ssal\_plnb\_5636 ssalplnb mixed\_tissue Salmo salar cDNA Salmo salar cDNA clone ssal\_plnb\_027\_107\_rev 5', mRNA sequence  
F gi|89830395|gb|DY692725.1|DY692725 EST\_ssal\_plnb\_5635 ssalplnb mixed\_tissue Salmo salar cDNA Salmo salar cDNA clone ssal\_plnb\_027\_107\_fwd 3', mRNA sequence

4 SNPs detected

A B C D E F  cosegregation weighted

45 - A A A - .   3/4 62.50
46 - C C C - .   3/4 62.50
114 C C C C T T   1/4 25.00
574 T A A A T T   3/4 75.00
