# Supplementary material for: Discovery and application of insertion-deletion (INDEL) polymorphisms for QTL mapping of early life-history traits in Atlantic salmon
Source: BMC Genomics. 2010 Mar 8;11:156. doi: 10.1186/1471-2164-11-156 (PMC2838853; doi:10.1186/1471-2164-11-156)
Supplement: Additional file 2 — Information on developed 76 locus single-run INDEL panel in Atlantic salmon. Information on fluorescence labeling, primer concentrations, PCR pooling and links to alignments, INDEL motifs and GENESCAN (Burge and Karlin 1997) predictions of genes/exons are available in html format. [file 1471-2164-11-156-S2.ZIP › Additionalfile2/snpsummary15402.html]

```
Cluster 6824 Contig 2

prev  Summary    Contig List  next
```

Size of Consensus sequence = 1179

Number of sequences = 6

Minimum redundancy = 2

Key

A gi|117504491|gb|EG836250.1|EG836250 EST\_ssal\_eve\_47919 ssaleve thyroid Salmo salar cDNA Salmo salar cDNA clone ssal\_eve\_565\_069\_fwd 3', mRNA sequence  
B gi|85026232|gb|DW554888.1|DW554888 EST\_ssal\_rgb2\_19307 rgb2 Salmo salar cDNA clone ssal\_rgb2\_531\_224\_rev 5', mRNA sequence  
C gi|89858490|gb|DY714613.1|DY714613 EST\_ssal\_rgb2\_70353 ssalrgb2 mixed\_tissue Salmo salar cDNA Salmo salar cDNA clone ssal\_rgb2\_614\_024\_rev 5', mRNA sequence  
D gi|89880366|gb|DY736489.1|DY736489 EST\_ssal\_rgb2\_92228 ssalrgb2 mixed\_tissue Salmo salar cDNA Salmo salar cDNA clone ssal\_rgb2\_650\_338\_fwd 3', mRNA sequence  
E gi|89858491|gb|DY714614.1|DY714614 EST\_ssal\_rgb2\_70354 ssalrgb2 mixed\_tissue Salmo salar cDNA Salmo salar cDNA clone ssal\_rgb2\_614\_024\_fwd 3', mRNA sequence  
F gi|85026231|gb|DW554887.1|DW554887 EST\_ssal\_rgb2\_19306 rgb2 Salmo salar cDNA clone ssal\_rgb2\_531\_224\_fwd 3', mRNA sequence

2 SNPs detected

A B C D E F  cosegregation weighted

720 . . G - G -   2/2 66.67
721 . . A - A -   2/2 66.67
