# Supplementary material for: Discovery and application of insertion-deletion (INDEL) polymorphisms for QTL mapping of early life-history traits in Atlantic salmon
Source: BMC Genomics. 2010 Mar 8;11:156. doi: 10.1186/1471-2164-11-156 (PMC2838853; doi:10.1186/1471-2164-11-156)
Supplement: Additional file 2 — Information on developed 76 locus single-run INDEL panel in Atlantic salmon. Information on fluorescence labeling, primer concentrations, PCR pooling and links to alignments, INDEL motifs and GENESCAN (Burge and Karlin 1997) predictions of genes/exons are available in html format. [file 1471-2164-11-156-S2.ZIP › Additionalfile2/snpsummary15900.html]

```
Cluster 7195 Contig 1

prev  Summary    Contig List  next
```

Size of Consensus sequence = 808

Number of sequences = 8

Minimum redundancy = 3

Key

A gi|85026160|gb|DW554816.1|DW554816 EST\_ssal\_rgb2\_19235 rgb2 Salmo salar cDNA clone ssal\_rgb2\_531\_181\_fwd 3', mRNA sequence  
B gi|117495994|gb|EG828211.1|EG828211 EST\_ssal\_eve\_40682 ssaleve thyroid Salmo salar cDNA Salmo salar cDNA clone ssal\_eve\_555\_139\_fwd 3', mRNA sequence  
C gi|117495995|gb|EG828212.1|EG828212 EST\_ssal\_eve\_40683 ssaleve thyroid Salmo salar cDNA Salmo salar cDNA clone ssal\_eve\_555\_139\_rev 5', mRNA sequence  
D gi|117856815|gb|EG929511.1|EG929511 EST\_ssal\_evf\_31716 ssalevf mixed\_tissue Salmo salar cDNA Salmo salar cDNA clone ssal\_evf\_542\_048\_fwd 3', mRNA sequence  
E gi|117856816|gb|EG929512.1|EG929512 EST\_ssal\_evf\_31717 ssalevf mixed\_tissue Salmo salar cDNA Salmo salar cDNA clone ssal\_evf\_542\_048\_rev 5', mRNA sequence  
F gi|24390640|gb|CA060397.1|CA060397 ssalrga508006 mixed\_tissue Salmo salar cDNA, mRNA sequence  
G gi|85023241|gb|DW551897.1|DW551897 EST\_ssal\_rgb2\_16316 rgb2 Salmo salar cDNA clone ssal\_rgb2\_526\_362\_fwd 3', mRNA sequence  
H gi|117509424|gb|EG841183.1|EG841183 EST\_ssal\_eve\_3958 ssaleve thyroid Salmo salar cDNA Salmo salar cDNA clone ssal\_eve\_504\_149\_rev 5', mRNA sequence

5 SNPs detected

A B C D E F G H  cosegregation weighted

76 - C C . C - - C   5/5 87.50
77 - A A . A - - A   5/5 87.50
78 - A A . A - - A   5/5 87.50
79 - A A . A - - A   5/5 87.50
80 - A A . A - - A   5/5 87.50
