# Supplementary material for: Discovery and application of insertion-deletion (INDEL) polymorphisms for QTL mapping of early life-history traits in Atlantic salmon
Source: BMC Genomics. 2010 Mar 8;11:156. doi: 10.1186/1471-2164-11-156 (PMC2838853; doi:10.1186/1471-2164-11-156)
Supplement: Additional file 2 — Information on developed 76 locus single-run INDEL panel in Atlantic salmon. Information on fluorescence labeling, primer concentrations, PCR pooling and links to alignments, INDEL motifs and GENESCAN (Burge and Karlin 1997) predictions of genes/exons are available in html format. [file 1471-2164-11-156-S2.ZIP › Additionalfile2/snpsummary16424.html]

```
Cluster 7602 Contig 1

prev  Summary    Contig List  next
```

Size of Consensus sequence = 840

Number of sequences = 6

Minimum redundancy = 2

Key

A gi|84984723|gb|DW535073.1|DW535073 EST\_ssal\_plnb\_3709 plnb Salmo salar cDNA clone ssal\_plnb\_021\_229\_fwd 3', mRNA sequence  
B gi|84981346|gb|DW531696.1|DW531696 EST\_ssal\_plnb\_332 plnb Salmo salar cDNA clone ssal\_plnb\_004\_061\_fwd 3', mRNA sequence  
C gi|85028523|gb|DW557179.1|DW557179 EST\_ssal\_rgb2\_21598 rgb2 Salmo salar cDNA clone ssal\_rgb2\_535\_055\_fwd 3', mRNA sequence  
D gi|24390342|gb|CA060099.1|CA060099 ssalrgb513010 mixed\_tissue Salmo salar cDNA, mRNA sequence  
E gi|24393515|gb|CA063272.1|CA063272 ssalrgb535086 mixed\_tissue Salmo salar cDNA, mRNA sequence  
F gi|89853211|gb|DY709334.1|DY709334 EST\_ssal\_rgb2\_65073 ssalrgb2 mixed\_tissue Salmo salar cDNA Salmo salar cDNA clone ssal\_rgb2\_604\_367\_fwd 3', mRNA sequence

2 SNPs detected

A B C D E F  cosegregation weighted

200 - - T . T T   2/2 83.33
201 - - G . G G   2/2 83.33
