# Supplementary material for: Discovery and application of insertion-deletion (INDEL) polymorphisms for QTL mapping of early life-history traits in Atlantic salmon
Source: BMC Genomics. 2010 Mar 8;11:156. doi: 10.1186/1471-2164-11-156 (PMC2838853; doi:10.1186/1471-2164-11-156)
Supplement: Additional file 2 — Information on developed 76 locus single-run INDEL panel in Atlantic salmon. Information on fluorescence labeling, primer concentrations, PCR pooling and links to alignments, INDEL motifs and GENESCAN (Burge and Karlin 1997) predictions of genes/exons are available in html format. [file 1471-2164-11-156-S2.ZIP › Additionalfile2/snpsummary16730.html]

```
Cluster 7839 Contig 1

prev  Summary    Contig List  next
```

Size of Consensus sequence = 878

Number of sequences = 6

Minimum redundancy = 2

Key

A gi|84568235|gb|DW339854.1|DW339854 SGP302619 Atlantic salmon Ovaries cDNA library Salmo salar cDNA clone KG4-2971 5', mRNA sequence  
B gi|117428614|gb|EG760838.1|EG760838 EST\_ssal\_sjb\_9160 ssalsjb mixed\_tissue Salmo salar cDNA Salmo salar cDNA clone ssal\_sjb\_018\_205\_fwd 3', mRNA sequence  
C gi|89836069|gb|DY695925.1|DY695925 EST\_ssal\_rgb2\_51664 ssalrgb2 mixed\_tissue Salmo salar cDNA Salmo salar cDNA clone ssal\_rgb2\_583\_241\_fwd 3', mRNA sequence  
D gi|89845998|gb|DY702121.1|DY702121 EST\_ssal\_rgb2\_57860 ssalrgb2 mixed\_tissue Salmo salar cDNA Salmo salar cDNA clone ssal\_rgb2\_593\_145\_fwd 3', mRNA sequence  
E gi|117483305|gb|EG815522.1|EG815522 EST\_ssal\_evd\_35290 ssalevd thymus Salmo salar cDNA Salmo salar cDNA clone ssal\_evd\_547\_021\_rev 5', mRNA sequence  
F gi|89855070|gb|DY711193.1|DY711193 EST\_ssal\_rgb2\_66932 ssalrgb2 mixed\_tissue Salmo salar cDNA Salmo salar cDNA clone ssal\_rgb2\_608\_309\_fwd 3', mRNA sequence

6 SNPs detected

A B C D E F  cosegregation weighted

431 - T T - T .   6/6 83.33
432 - A A - A .   6/6 83.33
433 - T T - T .   6/6 83.33
434 - T T - T .   6/6 83.33
435 - A A - A .   6/6 83.33
436 - T T - T .   6/6 83.33
