# Supplementary material for: Discovery and application of insertion-deletion (INDEL) polymorphisms for QTL mapping of early life-history traits in Atlantic salmon
Source: BMC Genomics. 2010 Mar 8;11:156. doi: 10.1186/1471-2164-11-156 (PMC2838853; doi:10.1186/1471-2164-11-156)
Supplement: Additional file 2 — Information on developed 76 locus single-run INDEL panel in Atlantic salmon. Information on fluorescence labeling, primer concentrations, PCR pooling and links to alignments, INDEL motifs and GENESCAN (Burge and Karlin 1997) predictions of genes/exons are available in html format. [file 1471-2164-11-156-S2.ZIP › Additionalfile2/snpsummary1729.html]

```
Cluster 266 Contig 3

prev  Summary    Contig List  next
```

Size of Consensus sequence = 1339

Number of sequences = 77

Minimum redundancy = 6

Key

A gi|117478886|gb|EG811103.1|EG811103 EST\_ssal\_evd\_31312 ssalevd thymus Salmo salar cDNA Salmo salar cDNA clone ssal\_evd\_541\_144\_fwd 3', mRNA sequence  
B gi|117479247|gb|EG811464.1|EG811464 EST\_ssal\_evd\_31638 ssalevd thymus Salmo salar cDNA Salmo salar cDNA clone ssal\_evd\_541\_358\_fwd 3', mRNA sequence  
C gi|45319496|gb|CK889763.1|CK889763 SGP149985 Atlantic salmon Ovaries cDNA library Salmo salar cDNA clone KG6-0294 5', mRNA sequence  
D gi|84567528|gb|DW339147.1|DW339147 SGP299647 Atlantic salmon Ovaries cDNA library Salmo salar cDNA clone KG4-1862 5', mRNA sequence  
E gi|50134993|gb|CO469879.1|CO469879 SGP262407 Atlantic salmon Eye cDNA library Salmo salar cDNA clone OY4-1254 5', mRNA sequence  
F gi|84567863|gb|DW339482.1|DW339482 SGP300727 Atlantic salmon Ovaries cDNA library Salmo salar cDNA clone KG4-2488 5', mRNA sequence  
G gi|89866891|gb|DY723014.1|DY723014 EST\_ssal\_rgb2\_78753 ssalrgb2 mixed\_tissue Salmo salar cDNA Salmo salar cDNA clone ssal\_rgb2\_629\_225\_rev 5', mRNA sequence  
H gi|29317596|gb|CB506370.1|CB506370 ssalplnb506092\_rev gut Salmo salar cDNA, mRNA sequence  
I gi|84982986|gb|DW533336.1|DW533336 EST\_ssal\_plnb\_1972 plnb Salmo salar cDNA clone ssal\_plnb\_015\_050\_rev 5', mRNA sequence  
J gi|117444812|gb|EG777035.1|EG777035 EST\_ssal\_evd\_52116 ssalevd thymus Salmo salar cDNA Salmo salar cDNA clone ssal\_evd\_570\_090\_fwd 3', mRNA sequence  
K gi|117835207|gb|EG907903.1|EG907903 EST\_ssal\_evf\_10270 ssalevf mixed\_tissue Salmo salar cDNA Salmo salar cDNA clone ssal\_evf\_512\_041\_fwd 3', mRNA sequence  
L gi|45321334|gb|CK891601.1|CK891601 SGP163864 Atlantic salmon Ovaries cDNA library Salmo salar cDNA clone KG4-1379 5', mRNA sequence  
M gi|29316186|gb|CB504960.1|CB504960 ssalplnb513255\_rev gut Salmo salar cDNA, mRNA sequence  
N gi|117867560|gb|EG940256.1|EG940256 EST\_ssal\_evf\_23799 ssalevf mixed\_tissue Salmo salar cDNA Salmo salar cDNA clone ssal\_evf\_531\_162\_fwd 3', mRNA sequence  
O gi|117522713|gb|EG854440.1|EG854440 EST\_ssal\_eve\_15189 ssaleve thyroid Salmo salar cDNA Salmo salar cDNA clone ssal\_eve\_520\_242\_fwd 3', mRNA sequence  
P gi|117552985|gb|EG883740.1|EG883740 EST\_ssal\_eve\_26727 ssaleve thyroid Salmo salar cDNA Salmo salar cDNA clone ssal\_eve\_536\_129\_fwd 3', mRNA sequence  
Q gi|85029053|gb|DW557709.1|DW557709 EST\_ssal\_rgb2\_22128 rgb2 Salmo salar cDNA clone ssal\_rgb2\_535\_374\_rev 5', mRNA sequence  
R gi|85048794|gb|DW576972.1|DW576972 EST\_ssal\_rgb2\_41391 rgb2 Salmo salar cDNA clone ssal\_rgb2\_567\_050\_rev 5', mRNA sequence  
S gi|117460377|gb|EG792596.1|EG792596 EST\_ssal\_evd\_12657 ssalevd thymus Salmo salar cDNA Salmo salar cDNA clone ssal\_evd\_515\_305\_fwd 3', mRNA sequence  
T gi|117449111|gb|EG781330.1|EG781330 EST\_ssal\_evd\_37981 ssalevd thymus Salmo salar cDNA Salmo salar cDNA clone ssal\_evd\_550\_276\_fwd 3', mRNA sequence  
U gi|89877167|gb|DY733290.1|DY733290 EST\_ssal\_rgb2\_89029 ssalrgb2 mixed\_tissue Salmo salar cDNA Salmo salar cDNA clone ssal\_rgb2\_645\_350\_rev 5', mRNA sequence  
V gi|117840937|gb|EG913633.1|EG913633 EST\_ssal\_evf\_15427 ssalevf mixed\_tissue Salmo salar cDNA Salmo salar cDNA clone ssal\_evf\_519\_058\_fwd 3', mRNA sequence  
W gi|117500720|gb|EG832740.1|EG832740 EST\_ssal\_eve\_44759 ssaleve thyroid Salmo salar cDNA Salmo salar cDNA clone ssal\_eve\_560\_328\_fwd 3', mRNA sequence  
X gi|117485698|gb|EG817915.1|EG817915 EST\_ssal\_evd\_20104 ssalevd thymus Salmo salar cDNA Salmo salar cDNA clone ssal\_evd\_525\_340\_fwd 3', mRNA sequence  
Y gi|117507259|gb|EG839018.1|EG839018 EST\_ssal\_eve\_2009 ssaleve thyroid Salmo salar cDNA Salmo salar cDNA clone ssal\_eve\_501\_303\_fwd 3', mRNA sequence  
Z gi|89845622|gb|DY701745.1|DY701745 EST\_ssal\_rgb2\_57484 ssalrgb2 mixed\_tissue Salmo salar cDNA Salmo salar cDNA clone ssal\_rgb2\_592\_298\_rev 5', mRNA sequence  
a gi|117546069|gb|EG877514.1|EG877514 EST\_ssal\_eve\_21122 ssaleve thyroid Salmo salar cDNA Salmo salar cDNA clone ssal\_eve\_528\_274\_fwd 3', mRNA sequence  
b gi|117487446|gb|EG819663.1|EG819663 EST\_ssal\_evd\_21678 ssalevd thymus Salmo salar cDNA Salmo salar cDNA clone ssal\_evd\_528\_004\_fwd 3', mRNA sequence  
c gi|117827785|gb|EG900481.1|EG900481 EST\_ssal\_evf\_39335 ssalevf mixed\_tissue Salmo salar cDNA Salmo salar cDNA clone ssal\_evf\_552\_189\_fwd 3', mRNA sequence  
d gi|117468412|gb|EG800631.1|EG800631 EST\_ssal\_evd\_57351 ssalevd thymus Salmo salar cDNA Salmo salar cDNA clone ssal\_evd\_577\_133\_fwd 3', mRNA sequence  
e gi|25998328|gb|CA769073.1|CA769073 ssalsrkc010091 kidney Salmo salar cDNA, mRNA sequence  
f gi|117453935|gb|EG786154.1|EG786154 EST\_ssal\_evd\_6858 ssalevd thymus Salmo salar cDNA Salmo salar cDNA clone ssal\_evd\_507\_334\_fwd 3', mRNA sequence  
g gi|117509966|gb|EG841725.1|EG841725 EST\_ssal\_eve\_4446 ssaleve thyroid Salmo salar cDNA Salmo salar cDNA clone ssal\_eve\_505\_018\_fwd 3', mRNA sequence  
h gi|117849435|gb|EG922131.1|EG922131 EST\_ssal\_evf\_53292 ssalevf mixed\_tissue Salmo salar cDNA Salmo salar cDNA clone ssal\_evf\_571\_151\_fwd 3', mRNA sequence  
i gi|45319473|gb|CK889740.1|CK889740 SGP149962 Atlantic salmon Ovaries cDNA library Salmo salar cDNA clone KG6-0270 5', mRNA sequence  
j gi|117442378|gb|EG774601.1|EG774601 EST\_ssal\_evd\_49925 ssalevd thymus Salmo salar cDNA Salmo salar cDNA clone ssal\_evd\_567\_074\_rev 5', mRNA sequence  
k gi|117449110|gb|EG781329.1|EG781329 EST\_ssal\_evd\_37980 ssalevd thymus Salmo salar cDNA Salmo salar cDNA clone ssal\_evd\_550\_276\_rev 5', mRNA sequence  
l gi|24345935|gb|CA044350.1|CA044350 ssalplnb513255 gut Salmo salar cDNA, mRNA sequence  
m gi|117827784|gb|EG900480.1|EG900480 EST\_ssal\_evf\_39334 ssalevf mixed\_tissue Salmo salar cDNA Salmo salar cDNA clone ssal\_evf\_552\_189\_rev 5', mRNA sequence  
n gi|29315671|gb|CB504445.1|CB504445 ssalmgd502354 gut Salmo salar cDNA, mRNA sequence  
o gi|117461033|gb|EG793252.1|EG793252 EST\_ssal\_evd\_13247 ssalevd thymus Salmo salar cDNA Salmo salar cDNA clone ssal\_evd\_516\_227\_fwd 3', mRNA sequence  
p gi|117468413|gb|EG800632.1|EG800632 EST\_ssal\_evd\_57352 ssalevd thymus Salmo salar cDNA Salmo salar cDNA clone ssal\_evd\_577\_133\_rev 5', mRNA sequence  
q gi|117442379|gb|EG774602.1|EG774602 EST\_ssal\_evd\_49926 ssalevd thymus Salmo salar cDNA Salmo salar cDNA clone ssal\_evd\_567\_074\_fwd 3', mRNA sequence  
r gi|85029054|gb|DW557710.1|DW557710 EST\_ssal\_rgb2\_22129 rgb2 Salmo salar cDNA clone ssal\_rgb2\_535\_374\_fwd 3', mRNA sequence  
s gi|117449523|gb|EG781742.1|EG781742 EST\_ssal\_evd\_38352 ssalevd thymus Salmo salar cDNA Salmo salar cDNA clone ssal\_evd\_551\_086\_fwd 3', mRNA sequence  
t gi|117522712|gb|EG854439.1|EG854439 EST\_ssal\_eve\_15188 ssaleve thyroid Salmo salar cDNA Salmo salar cDNA clone ssal\_eve\_520\_242\_rev 5', mRNA sequence  
u gi|117487445|gb|EG819662.1|EG819662 EST\_ssal\_evd\_21677 ssalevd thymus Salmo salar cDNA Salmo salar cDNA clone ssal\_evd\_528\_004\_rev 5', mRNA sequence  
v gi|117840938|gb|EG913634.1|EG913634 EST\_ssal\_evf\_15428 ssalevf mixed\_tissue Salmo salar cDNA Salmo salar cDNA clone ssal\_evf\_519\_058\_rev 5', mRNA sequence  
w gi|29322095|gb|CB510869.1|CB510869 ssalnwh507108 whole Salmo salar cDNA, mRNA sequence  
x gi|117522620|gb|EG854347.1|EG854347 EST\_ssal\_eve\_15105 ssaleve thyroid Salmo salar cDNA Salmo salar cDNA clone ssal\_eve\_520\_198\_fwd 3', mRNA sequence  
y gi|117472365|gb|EG804584.1|EG804584 EST\_ssal\_evd\_1645 ssalevd thymus Salmo salar cDNA Salmo salar cDNA clone ssal\_evd\_006\_302\_fwd 3', mRNA sequence  
z gi|117460376|gb|EG792595.1|EG792595 EST\_ssal\_evd\_12656 ssalevd thymus Salmo salar cDNA Salmo salar cDNA clone ssal\_evd\_515\_305\_rev 5', mRNA sequence  
A gi|117478887|gb|EG811104.1|EG811104 EST\_ssal\_evd\_31313 ssalevd thymus Salmo salar cDNA Salmo salar cDNA clone ssal\_evd\_541\_144\_rev 5', mRNA sequence  
B gi|117471493|gb|EG803712.1|EG803712 EST\_ssal\_evd\_860 ssalevd thymus Salmo salar cDNA Salmo salar cDNA clone ssal\_evd\_005\_142\_rev 5', mRNA sequence  
C gi|84982985|gb|DW533335.1|DW533335 EST\_ssal\_plnb\_1971 plnb Salmo salar cDNA clone ssal\_plnb\_015\_050\_fwd 3', mRNA sequence  
D gi|117870233|gb|EG942929.1|EG942929 EST\_ssal\_evf\_26205 ssalevf mixed\_tissue Salmo salar cDNA Salmo salar cDNA clone ssal\_evf\_534\_268\_rev 5', mRNA sequence  
E gi|117509967|gb|EG841726.1|EG841726 EST\_ssal\_eve\_4447 ssaleve thyroid Salmo salar cDNA Salmo salar cDNA clone ssal\_eve\_505\_018\_rev 5', mRNA sequence  
F gi|24335738|gb|CA036732.1|CA036732 ssaltc005086 reproductive Salmo salar cDNA, mRNA sequence  
G gi|117434993|gb|EG767216.1|EG767216 EST\_ssal\_evd\_43277 ssalevd thymus Salmo salar cDNA Salmo salar cDNA clone ssal\_evd\_558\_025\_fwd 3', mRNA sequence  
H gi|117870625|gb|EG943321.1|EG943321 EST\_ssal\_evf\_26558 ssalevf mixed\_tissue Salmo salar cDNA Salmo salar cDNA clone ssal\_evf\_535\_069\_fwd 3', mRNA sequence  
I gi|117485699|gb|EG817916.1|EG817916 EST\_ssal\_evd\_20105 ssalevd thymus Salmo salar cDNA Salmo salar cDNA clone ssal\_evd\_525\_340\_rev 5', mRNA sequence  
J gi|24350007|gb|CA044967.1|CA044967 ssalplnb505113 gut Salmo salar cDNA, mRNA sequence  
K gi|117849424|gb|EG922120.1|EG922120 EST\_ssal\_evf\_53291 ssalevf mixed\_tissue Salmo salar cDNA Salmo salar cDNA clone ssal\_evf\_571\_151\_rev 5', mRNA sequence  
L gi|117546068|gb|EG877513.1|EG877513 EST\_ssal\_eve\_21121 ssaleve thyroid Salmo salar cDNA Salmo salar cDNA clone ssal\_eve\_528\_274\_rev 5', mRNA sequence  
M gi|29312909|gb|CB501683.1|CB501683 ssalga508150 head Salmo salar cDNA, mRNA sequence  
N gi|117552986|gb|EG883741.1|EG883741 EST\_ssal\_eve\_26728 ssaleve thyroid Salmo salar cDNA Salmo salar cDNA clone ssal\_eve\_536\_129\_rev 5', mRNA sequence  
O gi|117522619|gb|EG854346.1|EG854346 EST\_ssal\_eve\_15104 ssaleve thyroid Salmo salar cDNA Salmo salar cDNA clone ssal\_eve\_520\_198\_rev 5', mRNA sequence  
P gi|117500719|gb|EG832739.1|EG832739 EST\_ssal\_eve\_44758 ssaleve thyroid Salmo salar cDNA Salmo salar cDNA clone ssal\_eve\_560\_328\_rev 5', mRNA sequence  
Q gi|117453934|gb|EG786153.1|EG786153 EST\_ssal\_evd\_6857 ssalevd thymus Salmo salar cDNA Salmo salar cDNA clone ssal\_evd\_507\_334\_rev 5', mRNA sequence  
R gi|117472364|gb|EG804583.1|EG804583 EST\_ssal\_evd\_1644 ssalevd thymus Salmo salar cDNA Salmo salar cDNA clone ssal\_evd\_006\_302\_rev 5', mRNA sequence  
S gi|117449524|gb|EG781743.1|EG781743 EST\_ssal\_evd\_38353 ssalevd thymus Salmo salar cDNA Salmo salar cDNA clone ssal\_evd\_551\_086\_rev 5', mRNA sequence  
T gi|117867561|gb|EG940257.1|EG940257 EST\_ssal\_evf\_23800 ssalevf mixed\_tissue Salmo salar cDNA Salmo salar cDNA clone ssal\_evf\_531\_162\_rev 5', mRNA sequence  
U gi|117523263|gb|EG854990.1|EG854990 EST\_ssal\_eve\_15684 ssaleve thyroid Salmo salar cDNA Salmo salar cDNA clone ssal\_eve\_521\_116\_rev 5', mRNA sequence  
V gi|89845623|gb|DY701746.1|DY701746 EST\_ssal\_rgb2\_57485 ssalrgb2 mixed\_tissue Salmo salar cDNA Salmo salar cDNA clone ssal\_rgb2\_592\_298\_fwd 3', mRNA sequence  
W gi|117870624|gb|EG943320.1|EG943320 EST\_ssal\_evf\_26557 ssalevf mixed\_tissue Salmo salar cDNA Salmo salar cDNA clone ssal\_evf\_535\_069\_rev 5', mRNA sequence  
X gi|24344070|gb|CA043150.1|CA043150 ssalplnb506092 gut Salmo salar cDNA, mRNA sequence  
Y gi|117434992|gb|EG767215.1|EG767215 EST\_ssal\_evd\_43276 ssalevd thymus Salmo salar cDNA Salmo salar cDNA clone ssal\_evd\_558\_025\_rev 5', mRNA sequence

7 SNPs detected

A B C D E F G H I J K L M N O P Q R S T U V W X Y Z a b c d e f g h i j k l m n o p q r s t u v w x y z A B C D E F G H I J K L M N O P Q R S T U V W X Y  cosegregation weighted

518 . T T T A T T T T A A T A T A T T T T A A A T T T T T A A A . T T T T A A A A A A A A T T A A A A T T T . T T T T . . . . . . . . . . . . . . . . . . . .   6/7 60.11
741 . . . . . . . . . . . . . T . T T T T C C C T T T T T C C C C T T T T C C C C C C C C T T C C C C T T T T T T T T T T T T T T T T T T T T T T . . . . . .   6/7 63.45
791 . . . . . . . . . . . . . . . - - - - T T T - - - - - T T T T - - - - T T T T T T T T - - T T T T - - - - - - - - - - - - - - - - - - - - - - . . . - . .   6/7 63.45
792 . . . . . . . . . . . . . . . - - - - T T T - - - - - T T T T - - - - T T T T T T T T - - T T T T - - - - - - - - - - - - - - - - - - - - - - . . . - . .   6/7 63.45
844 . . . . . . . . . . . . . . . . . . . . . . - . G A A A A A A - A A - - A A A - A A - A A A A A A A A A A A A - A A A A A A A A A - A A - A A . . A A . .   1/7 9.28
1032 . . . . . . . . . . . . . . . . . . . . . . . . . . . . . . . . . . . T T T T T T T T - . T T T T - - - - - - - - - - - - - - - - - - - - - - - - - - - -   6/7 45.64
1033 . . . . . . . . . . . . . . . . . . . . . . . . . . . . . . . . . . . G G G G G G G G - . G G G G - - - - - - - - - - - - - - - - - - - - - - - - - - - -   6/7 45.64
