# Supplementary material for: Discovery and application of insertion-deletion (INDEL) polymorphisms for QTL mapping of early life-history traits in Atlantic salmon
Source: BMC Genomics. 2010 Mar 8;11:156. doi: 10.1186/1471-2164-11-156 (PMC2838853; doi:10.1186/1471-2164-11-156)
Supplement: Additional file 2 — Information on developed 76 locus single-run INDEL panel in Atlantic salmon. Information on fluorescence labeling, primer concentrations, PCR pooling and links to alignments, INDEL motifs and GENESCAN (Burge and Karlin 1997) predictions of genes/exons are available in html format. [file 1471-2164-11-156-S2.ZIP › Additionalfile2/snpsummary17300.html]

```
Cluster 8270 Contig 1

prev  Summary    Contig List  next
```

Size of Consensus sequence = 720

Number of sequences = 6

Minimum redundancy = 2

Key

A gi|85047171|gb|DW575349.1|DW575349 EST\_ssal\_rgb2\_39768 rgb2 Salmo salar cDNA clone ssal\_rgb2\_564\_254\_fwd 3', mRNA sequence  
B gi|117849312|gb|EG922008.1|EG922008 EST\_ssal\_evf\_1164 ssalevf mixed\_tissue Salmo salar cDNA Salmo salar cDNA clone ssal\_evf\_005\_258\_rev 5', mRNA sequence  
C gi|85027058|gb|DW555714.1|DW555714 EST\_ssal\_rgb2\_20133 rgb2 Salmo salar cDNA clone ssal\_rgb2\_532\_336\_fwd 3', mRNA sequence  
D gi|117475082|gb|EG807301.1|EG807301 EST\_ssal\_evd\_27890 ssalevd thymus Salmo salar cDNA Salmo salar cDNA clone ssal\_evd\_536\_180\_rev 5', mRNA sequence  
E gi|117838624|gb|EG911320.1|EG911320 EST\_ssal\_evf\_13345 ssalevf mixed\_tissue Salmo salar cDNA Salmo salar cDNA clone ssal\_evf\_516\_136\_rev 5', mRNA sequence  
F gi|117838625|gb|EG911321.1|EG911321 EST\_ssal\_evf\_13346 ssalevf mixed\_tissue Salmo salar cDNA Salmo salar cDNA clone ssal\_evf\_516\_136\_fwd 3', mRNA sequence

5 SNPs detected

A B C D E F  cosegregation weighted

236 - - - - C C   5/5 100.00
237 - - - - T T   5/5 100.00
238 - - - - A A   5/5 100.00
239 - - - - A A   5/5 100.00
582 . . T T C C   5/5 66.67
