# Supplementary material for: Discovery and application of insertion-deletion (INDEL) polymorphisms for QTL mapping of early life-history traits in Atlantic salmon
Source: BMC Genomics. 2010 Mar 8;11:156. doi: 10.1186/1471-2164-11-156 (PMC2838853; doi:10.1186/1471-2164-11-156)
Supplement: Additional file 2 — Information on developed 76 locus single-run INDEL panel in Atlantic salmon. Information on fluorescence labeling, primer concentrations, PCR pooling and links to alignments, INDEL motifs and GENESCAN (Burge and Karlin 1997) predictions of genes/exons are available in html format. [file 1471-2164-11-156-S2.ZIP › Additionalfile2/snpsummary17363.html]

```
Cluster 8316 Contig 1

prev  Summary    Contig List  next
```

Size of Consensus sequence = 854

Number of sequences = 6

Minimum redundancy = 2

Key

A gi|117521828|gb|EG853555.1|EG853555 EST\_ssal\_eve\_14392 ssaleve thyroid Salmo salar cDNA Salmo salar cDNA clone ssal\_eve\_519\_210\_fwd 3', mRNA sequence  
B gi|117426321|gb|EG758545.1|EG758545 EST\_ssal\_sjb\_3830 ssalsjb mixed\_tissue Salmo salar cDNA Salmo salar cDNA clone ssal\_sjb\_010\_216\_fwd 3', mRNA sequence  
C gi|24388224|gb|CA057981.1|CA057981 ssalrgb543177 mixed\_tissue Salmo salar cDNA, mRNA sequence  
D gi|117426432|gb|EG758656.1|EG758656 EST\_ssal\_sjb\_3831 ssalsjb mixed\_tissue Salmo salar cDNA Salmo salar cDNA clone ssal\_sjb\_010\_216\_rev 5', mRNA sequence  
E gi|117521829|gb|EG853556.1|EG853556 EST\_ssal\_eve\_14393 ssaleve thyroid Salmo salar cDNA Salmo salar cDNA clone ssal\_eve\_519\_210\_rev 5', mRNA sequence  
F gi|85041274|gb|DW569452.1|DW569452 EST\_ssal\_rgb2\_33871 rgb2 Salmo salar cDNA clone ssal\_rgb2\_554\_324\_fwd 3', mRNA sequence

4 SNPs detected

A B C D E F  cosegregation weighted

402 A G A G A A   1/4 25.00
600 C T T T C T   1/4 25.00
702 . G - G - G   2/4 41.67
703 . T - T - T   2/4 41.67
