# Supplementary material for: Discovery and application of insertion-deletion (INDEL) polymorphisms for QTL mapping of early life-history traits in Atlantic salmon
Source: BMC Genomics. 2010 Mar 8;11:156. doi: 10.1186/1471-2164-11-156 (PMC2838853; doi:10.1186/1471-2164-11-156)
Supplement: Additional file 3 — Information on GenBank accession numbers, primer sequences and literature references of the genomic and EST-derived microsatellite markers used for construction of Atlantic salmon linkage map. [file 1471-2164-11-156-S3.DOC]

**Appendix 3.** GenBank accession numbers, primer sequences and literature references of the genomic and EST-derived microsatellite markers used for construction of the linkage map.

| No | Marker name | Alternate | GenBank | Forward primer | Reverse primer | Reference |
| --- | --- | --- | --- | --- | --- | --- |
|  |  | name | Acc. No. | 5'-3' | 5'-3' |  |
| 1 | *BHMS144* | *Ssa0014NVH* | AF256665 | TTGTGCCGATTTAGGACG | GCCTTTAACGTAAGTGGTAG | http://www.salmongenome.no/ |
| 2 | *BHMS189* | *Ssa0026NVH* | AF256676 | AAACACCCTTCCCTTCAC | CAATTCAGGTCAAACCAAC | http://www.salmongenome.no/ |
| 3 | *BHMS269* | *Ssa0039NVH* | AF256689 | AACACACATGACGCAGCC | CAATTCAGCCTCTCCCAC | http://www.salmongenome.no/ |
| 4 | *BHMS311* | *Ssa0084NVH* | AF256730 | ACCTCAGCACATGAACAC | TGACAGAGCCATAGACCG | http://www.salmongenome.no/ |
| 5 | *BHMS365* | *Ssa0053NVH* | AF256703 | GGAATTGTGTGTAGATGGG | ACTTGGCAAGGAGCAGAC | http://www.salmongenome.no/ |
| 6 | *BHMS386* | *Ssa0062NVH* | AF256712 | CGTTAAAACCCCGTGGAG | GACTAAAAAGCGTCTGGC | http://www.salmongenome.no/ |
| 7 | *BHMS7-017* | *Ssa0090NVH* | AF256735 | GTCTTACATCATACCCCAG | TCAGAAGAAGAGGCGAGC | http://www.salmongenome.no/ |
| 8 | *BHMS7-043* | *Ssa0006NVH* | AF256659 | CACTCCCACTCACCATAC | CTCTTTGGCTTTGACATTAC | http://www.salmongenome.no/ |
| 9 | *CTAX* |  | BG935488 | TGACCCCACCAAGTTTTTCT | GTTTAAACACAGTAAGCCCATCTATTG | Vasemägi et al. 2005 |
| 10 | *EST101* |  | CA048828 | GAGGGCTTCCCATACAACAA | GTTTAAGCGGTGAGTTGACGAGAG | Vasemägi et al. 2005 |
| 11 | *EST103* |  | CA048302 | TTGCCACCTCTAAACGCTTC | GTTTAAATGAACCCCAGCCATACA | Vasemägi et al. 2005 |
| 12 | *EST105* |  | CA047718 | ATTTACCGCCTGGTGATGTC | GTTTGCAAAGCCCTCATGTTGATT | Vasemägi et al. 2005 |
| 13 | *EST107* |  | CA047220 | AGCGTTTACGTCGAATCCAA | GTTTCTCATGGAGGGTGGAAGTGT | Vasemägi et al. 2005 |
| 14 | *EST11* |  | CA060208 | GCAACAATTCCCTTTTGACC | GTTTCGTGCAGTAGGAAAGGGGTA | Vasemägi et al. 2005 |
| 15 | *EST115* |  | CA041953 | TCACAGTCGCAGGAGGTAAG | GTTTTGACCCAGAGACAGATGACCT | Vasemägi et al. 2005 |
| 16 | *EST123* |  | CA039983 | GCGGCCCTTAGTGTAATCAA | GTTTCTCGCCAGTCACTCTTCAA | Vasemägi et al. 2005 |
| 17 | *EST127* |  | CA038592 | AAGCATCAAACCAACCTCATT | GTTTCGGGGGTGAAGATGTCTACT | Vasemägi et al. 2005 |
| 18 | *EST138* |  | CB514761 | CTGTCCTTGGGCACATTTTT | GTTTACAGCTCTGGTTCCGACA | Vasemägi et al. 2005 |
| 19 | *EST141* |  | CB514369 | CAATCATGCCTTCAGACTGTG | GTTTCCCATTTCTCCCACAATCAC | Vasemägi et al. 2005 |
| 20 | *EST19* |  | CA060177 | CGCTTCCTGGACAAAAATTA | GTTTGAGCACACCCATTCTCA | Vasemägi et al. 2005 |
| 21 | *EST28* |  | CA064557 | CACAGGCACACACTCCTCAT | GTTTCAGGTGAAGAGCATGACCAA | Vasemägi et al. 2005 |
| 22 | *EST40* |  | CA053293 | TCTCATGGTGAGCAACAAACA | GTTTACTCTGGGGCATTCATTCAG | Vasemägi et al. 2005 |
| 23 | *EST41* |  | CA040282 | TGCAAGTAAAGGCAGGGTTT | GTTTGTGGTAGGATTGGGGTTCCT | Vasemägi et al. 2005 |
| 24 | *EST44* |  | CA047944 | GCCGCCCAGATTATCAGTAA | GTTTGTTTCCAAACCAAAAACTGAA | Vasemägi et al. 2005 |
| 25 | *EST46* |  | CA053162 | CTCAACTCGTTCCCCTTCTG | GTTTCAGAAAATCCTTTTGGCTCA | Vasemägi et al. 2005 |
| 26 | *EST53* |  | CA058580 | ATAACATGCAAGCGGTTTCC | GTTTGCTGGAAGTGTTGAGTTGC | Vasemägi et al. 2005 |
| 27 | *EST58* |  | CA056586 | GCCTACATCGCACACCATAA | GTTTCCACTCATTCTCGCTTTTCA | Vasemägi et al. 2005 |
| 28 | *EST6* |  | CA058557 | TCCAACACCACAATCTTCAAGT | GTTTGTCGTTTCGGGTGTAAATG | Vasemägi et al. 2005 |
| 29 | *EST68* |  | CA062844 | TGACACTGTGGCCTGTCTCT | GTTTGAGTTCTGGGTTATTTATTCACA | Vasemägi et al. 2005 |
| 30 | *EST70* |  | CA062621 | TTAAAACTCCTGCCCTGTGG | GTTTCTTCCAAGGCTTGATGTCC | Vasemägi et al. 2005 |
| 31 | *EST74* |  | CA059136 | AGGGTAGTGAGAAAGCAGCAA | GTTTAACTGGCTGGCCATAGG | Vasemägi et al. 2005 |
| 32 | *EST9* |  | CA055301 | AGAACCAAGGGTACCGATCC | GTTTGGGAAATGGGTGGTAAGAAAA | Vasemägi et al. 2005 |
| 33 | *HSP* |  | BG934281 | ACTGCTTCTCCCCTGCTACA | GTTTGCGAACCACACATATACCAC | Vasemägi et al. 2005 |
| 34 | *MHCI* |  |  | GGAGAGCTGCCCAGATGACTT | GTTTCAATTACCACAAGCCCGCTC | Grimholt et al. 2002 |
| 35 | *MHCII* |  |  | GATGGCAAAGAGGAAAGTGAG | GTTTGTTATGCTCTACCTCTGAA | Stet et al. 2002 |
| 36 | *Oc18* |  |  | TAGTGTTCCGTGTTCGCCTG | CACCTTCCATCTCTCATTCCAC | Lulla et al. (2005) |
| 37 | *Ogo4* |  | AF009796 | GTCGTCACTGGCATCAGCTA | GAGTGGAGATGCAGCCAAAG | http://www.salmongenome.no/ |
| 38 | *Omm1070* |  | AF375019 | GACAGGTTGTGTCGAATGGA | GGTGGGATTCAGTGTGTTAAAC | Rexroad et al. (2001) |
| 39 | *OMM1105* |  | AF352768 | GCACACTGTCTGGGTAAGAGA | GCAGAGCCACACTAAACCA | Rexroad et al. (2002) |
| 40 | *Omm1107* |  | AF375022 | ACCTTATCCTGTTGCTGCTAC | ATTGCCAGAGGAAACGTC | Rexroad et al. (2001) |
| 41 | *OMM1120* |  | AF352772 | TTGAAGACAAGTGAGCGAGAG | TTGGTGTTCCCAGGACAGTAA | Rexroad et al. (2002) |
| 42 | *Omm1121* |  | AF375027 | CTGCCAATGTTTGTCTATG | GGCTGTCAATCTGTCTTCTAC | Rexroad et al. (2001) |
| 43 | *OMM1308* |  |  | GCTCTGGTTCAAGTTGGTTTG | CATTCGGTCATAGCCTACA | Palti et al. (2002) |
| 44 | *OMM1400* |  |  | TGTGTCTCTCAATGCGACCTG | GGCAAAACTCCACGGACTGTA | Rodriquez et al. (2003) |
| 45 | *Omy1011UW* |  |  | AACTTGCTATGTGAATGTGC | GACAAAAGTGACTGGTTGGT | Nichols et al. (2003) |
| 46 | *Omy14INRA* |  |  | GTCAGCGATAATCCACATGG | CCGTTATGGAGATGTGTAGGG | Gharbi, K., and R. Guyomard, Jouy-en-Josas, INRA |
| 47 | *Omy272UOG* |  |  | CTGTGTGAAGGCATGCAAAGG | ATTCAGCAGTAGTGGGCTTTAACC | Jackson et al. (1998) |
| 48 | *Omy7INRA* |  |  | CAAGGAATGGCACAGCTTG | TTAAGTTTTGCCTAGATAAGGG | Gharbi, K., and R. Guyomard, Jouy-en-Josas, INRA |
| 49 | *OmyRGT13TUF* |  | AB087592 | GTACTCCAGCTCCTCCCTCC | ACACCCCACTTTCTCTCCCT | A. Ozaki & N. Okamoto, pers. comm., TUF, Japan |
| 50 | *OmyRGT44TUF* |  | AB087611 | GAGGGTTGGAGTACACAGAAGG | ATGTGGGGACATATTAACTGGC | A. Ozaki & N. Okamoto, pers. comm., TUF, Japan |
| 51 | *One108ADFG* |  | AF274523 | TGCAGAGCCATACTAAACCA | AAGAATTGAGAGATGCAGGG | A. Ozaki & N. Okamoto, pers. comm., TUF, Japan |
| 52 | *One2ASC* |  | U56700 | GGTGCCAAGGTTCAGTTTATGTT | CAGGAATTTACAGGACCCAGGTT | Scribner et al. (1996) |
| 53 | *Sleel53* |  | U86704 | TGATTTGTTGCCTGCTGCTTCC | GTTTCCTGCTGCCCACATCATCC | Gilbey et al. (2004) |
| 54 | *Sleen82* |  | U86706 | CATGGAGAATCCCACTTTCTTA | GTTTCAGGGAGTGATATGGGACATAA | Gilbey et al. (2004) |
| 55 | *Ssa124* |  | AF420561 | TGTTTCGTATGGTGTGTGTG | CCCTCTAGCACCAGGGTCA | http://www.salmongenome.no/ |
| 56 | *Ssa14* |  |  | CCTTTTGACAGATTTAGGATTTC | CAAACCAAACATACCTAAAGCC | McConnell et al. (1995) |
| 57 | *Ssa197* |  |  | TGGCAGGGATTTGACATAAC | GTTTGGGTTGAGTAGGGAGGCTTG | O'Reilly et al. (1996) |
| 58 | *Ssa224* |  | AF019168 | ACAGACAGAACTGTGCATC | TGACTGCATTTATCAGAGAG | http://www.salmongenome.no/ |
| 59 | *Ssa289* |  |  | CTTTACAAATAGACAGACT | TCATACAGTCACTATCATC | McConnell et al. (1995) |
| 60 | *Ssa401UOS* |  | AJ402718 | ACTGGTTGTTGCAGAGTTTGATGC | AAACATACCTGATTCCCGAACCAG | Cairney et al. (2000) |
| 61 | *Ssa405* |  |  | CTGAGTGGGAATGGACCAGACA | GTTTACTCGGGAGGCCCAGACTTGAT | Cairney et al. (2000) |
| 62 | *Ssa406UOS* |  | AJ402723 | ACCAACCTGCACATGTCTTCTATG | GCTGCCGCCTGTTGTCTCTTT | Cairney et al. (2000) |
| 63 | *Ssa407* |  |  | TCGTGACTACTAAGTCTTTGACCA | GTTTGTGTAGGCAGGTGTGGAC | Cairney et al. (2000) |
| 64 | *Ssa417UOS* |  | AJ402734 | AGACAGGTCCAGACAAGCACTCA | ATCAAATCCACTGGGGTTATACTG | Cairney et al. (2000) |
| 65 | *Ssa419UOS* |  | AJ402736 | GGTCGTATCGCGTTTCAGGA | TGCTGCAATAAAGAGATGCTTGTT | Cairney et al. (2000) |
| 66 | *Ssa4DIAS* |  |  | ACAATCACCATCCTGTATGAC | GCTACTGTATATGCTTCTGTCC | L-E. Holm, pers. comm., Danish Institute of Agricultural Sciences |
| 67 | *Ssa85* |  |  | ACCCGCTCCTCACTTAATC | GTTTCAAGCTACCCCATGCAGAG | McConnell et al. (1995) |
| 68 | *Ssd30* |  |  | TGTTGACTTCCTTCCCCAAG | GTTTAGCAGTAAAGAGAGAGACTG | O'Reilly et al. 1996 Can J Fish Aquat Sci 53:2292-2298 |
| 69 | *SSf43* |  |  | GAGTCACTCAAAGTGAGGCC | GTTTAGCGGCATAACGTGCTGTGT | Sánchez et al. 1996 |
| 70 | *Ssleer15.1* |  | U86708 | CATGTGCGTGTGCTTTTACAG | GTTTTCTGCATGTAGAACCCTGACC | Gilbey et al. (2004) |
| 71 | *Ssosl25* |  |  | ATCTACACAGCTCCTGGTGGCAG | GTTTCATGTAATGGGTCGAGAGAAGTG | Slettan et al. 1995a |
| 72 | *Ssosl438* |  |  | GACAACACACAACCAAGGCAC | GTTTATGCTAGGTCTTTATGCATTGT | Slettan et al. 1996 |
| 73 | *Ssosl85* |  |  | TGTGGATTTTTGTATTATGTTA | GTTTATACATTTCCTCCTCATTCAG | Slettan et al. 1995a |
| 74 | *SSsp1605* |  | AY081812 | TCTGAGGCTCCTTCTACACTGA | GTTTGGTAGGTGCAAGAAAAAAGGAC | Patterson et al. (2004) |
| 75 | *SSsp2201* |  | AY081807 | TTTAGATGGTGGGATACTGGGAGGC | GTTTCGGGAGCCCCATAACCCTACTAATAAC | Patterson et al. (2004) |
| 76 | *SSsp2210* |  | AY081808 | CCTTTTTCCAATGGGATTCA | GTTTCATGCACACACATTCACTGC | Patterson et al. (2004) |
| 77 | *SSsp2215* |  | AY081810 | GGTCAGTCAGTCACACCATGC | GTTTGTCACTAGCCAGGTGTCC | Patterson et al. (2004) |
