# Supplementary material for: Discovery and application of insertion-deletion (INDEL) polymorphisms for QTL mapping of early life-history traits in Atlantic salmon
Source: BMC Genomics. 2010 Mar 8;11:156. doi: 10.1186/1471-2164-11-156 (PMC2838853; doi:10.1186/1471-2164-11-156)
Supplement: Additional file 4 — Linkage information of 50 INDELs and 77 microsatellite markers that were segregating in two families used for generation of Atlantic salmon linkage map. [file 1471-2164-11-156-S4.DOC]

**Appendix 4.** Linkage information of 50 INDELs and 77 microsatellite markers in Atlantic salmon.

| No | GRASP LG | 57X67male map | | 62X91male map | | | combined male map | | | 57X67female map | | | 62X91female map | | | combined female map | | |
| --- | --- | --- | --- | --- | --- | --- | --- | --- | --- | --- | --- | --- | --- | --- | --- | --- | --- | --- |
|  | Group | Marker | cM |  | Marker | cM |  | Marker | cM |  | Marker | cM |  | Marker | cM |  | Marker | cM |
| 1 | AS-1 | *EST115* | 0 |  | *EST115* | 0 |  | *EST115* | 0 |  | *Ssa406UOS* | 0 |  | *Ssa406UOS* | 0 |  | *Ssa406UOS* | 0 |
|  |  | *Ssa406UOS* | 0 |  | *Ssa406UOS* | 0.5 |  | *Ssa406UOS* | 0.3 |  | *11971N* | 33 |  | *11971N* | 26.7 |  | *11971N* | 30 |
|  |  | *2044M* | 25.1 |  | *11971N* | 1.59 |  | *11971N* | 1.3 |  |  |  |  |  |  |  |  |  |
|  |  |  |  |  |  |  |  | *2044M* | 25.4 |  |  |  |  |  |  |  |  |  |
| 2 | AS-4 | *HSP* | 0 |  | *OMM1105* | UN |  |  |  |  | *HSP* | UN |  | *HSP* | 0 |  |  |  |
|  |  | *OMM1105* | 34.4 |  |  |  |  |  |  |  | *Ssa171* | 0 |  | *11005M* | 21.5 |  |  |  |
|  |  | *Ssa171* | 35.1 |  |  |  |  |  |  |  | *Ind2070* | 25 |  | *OMM1105* | 40.4 |  |  |  |
|  |  | *One108ADFG* | 35.1 |  |  |  |  |  |  |  |  |  |  |  |  |  |  |  |
| 3 | AS-5 | *BHMS7-017* | 0 |  | *BHMS7-017* | 0 |  |  |  |  | *Ind2377* | 0 |  | *BHMS7-017* | 0 |  | *Ind2377* | 0 |
|  |  | *4151e* | 0 |  | *4151e* | 35 |  |  |  |  | *4151e* | 3.1 |  | *EST9* | 2.8 |  | *BHMS7-017* | 0.3 |
|  |  | *EST9* | 0 |  | *EST9* | 35 |  |  |  |  | *EST9* | 3.1 |  | *Ind2130* | 4.2 |  | *4151e* | 3.1 |
|  |  | *OMM1308* | 0 |  | *Ind2130* | 35 |  |  |  |  | *SSsp2201* | 38.9 |  | *OMM1308* | 29.9 |  | *EST9* | 3.1 |
|  |  | *SSsp2201* | 0.3 |  | *SSsp2201* | 35 |  |  |  |  |  |  |  | *SSsp2201* | 52.7 |  | *Ind2130* | 4.3 |
|  |  |  |  |  |  |  |  |  |  |  |  |  |  |  |  |  | *OMM1308* | 26 |
|  |  |  |  |  |  |  |  |  |  |  |  |  |  |  |  |  | *SSsp2201* | 46 |
| 4 | AS-6 | *MHCII* | UN |  |  |  |  |  |  |  | *MHCII* | 0 |  | *MHCII* | 0 |  | *MHCII* | 0 |
|  |  |  |  |  |  |  |  |  |  |  | *SSsp2210* | 7.8 |  | *SSsp2210* | 8.9 |  | *SSsp2210* | 8.4 |
|  |  |  |  |  |  |  |  |  |  |  | *Ssa14* | UN |  |  |  |  |  |  |
| 5 | AS-7 | *BHMS269* | 0 |  | *BHMS269* | 0 |  | *BHMS269* | 0 |  | *BHMS269* | 0 |  | *BHMS269* | 0 |  | *BHMS269* | 0 |
|  |  | *SSsp2216* | 1 |  | *15900H* | 3.4 |  | *15900H* | 2.1 |  | *SSsp2216* | 34.4 |  | *22471D* | 17.8 |  | *22471D* | 18 |
|  |  | *SSsp2215* | UN |  | *SSsp2216* | 4.2 |  | *SSsp2216* | 2.6 |  | *SSsp2215* | 38.3 |  | *SSsp2216* | 33.4 |  | *SSsp2216* | 34 |
|  |  |  |  |  | *22471D* | 5 |  | *22471D* | 3.4 |  |  |  |  | *SSsp2215* | 37 |  | *SSsp2215* | 38 |
|  |  |  |  |  | *SSsp2215* | UN |  |  |  |  |  |  |  |  |  |  |  |  |
| 6 | AS-8 | *Ssa401UOS* | 0 |  | *Ssa401UOS* | 0 |  | *Ssa401UOS* | 0 |  | *Ssa401UOS* | 0 |  | *Ssa401UOS* | 0 |  | *Ssa401UOS* | 0 |
|  |  | *1338L* | 0 |  | *Ssa197* | 3.9 |  | *1338L* | 0 |  | *1338L* | 13.6 |  | *1338L* | 27.2 |  | *1338L* | 20 |
|  |  | *9520D* | 4.1 |  |  |  |  | *9520D* | 3.7 |  | *Ssa197* | 19.8 |  | *Ssa197* | 27.2 |  | *Ssa197* | 24 |
|  |  | *Ssa197* | 4.8 |  |  |  |  | *Ssa197* | 4.3 |  |  |  |  |  |  |  |  |  |
| 7 | AS-9 | *Ssosl438* | 0 |  | *EST141* | 0 |  | *Ssosl438* | 0 |  | *Ind139* | 0 |  | *Ssosl438* | 0 |  |  |  |
|  |  | *Ind139* | 1.8 |  | *BHMS189* | 0.4 |  | *Ind139* | 1.9 |  | *2479B* | 11.6 |  | *21188C* | 6.2 |  |  |  |
|  |  | *EST141* | 2.4 |  | *Ind1836* | 0.4 |  | *EST141* | 2.4 |  | *BHMS189* | 29.9 |  | *Ind139* | 27.6 |  |  |  |
|  |  | *BHMS189* | 2.4 |  | *32c* | 1.3 |  | *BHMS189* | 2.6 |  |  |  |  | *17300F* | 32.8 |  |  |  |
|  |  | *17300F* | 2.7 |  |  |  |  | *Ind1836* | 2.6 |  |  |  |  | *32c* | 39.7 |  |  |  |
|  |  | *32c* | 5 |  |  |  |  | *17300F* | 2.9 |  |  |  |  |  |  |  |  |  |
|  |  |  |  |  |  |  |  | *32c* | 4.4 |  |  |  |  |  |  |  |  |  |
| 8 | AS-10 | *CTAX* | 0 |  | *CTAX* | 0 |  | *CTAX* | 0 |  | *CTAX* | 0 |  | *CTAX* | 0 |  | *CTAX* | 0 |
|  |  | *EST19* | 0.3 |  | *EST58* | 0 |  | *EST58* | 0.2 |  | *EST58* | 0.7 |  | *8570Q* | 2.1 |  | *8570Q* | 1.3 |
|  |  | *EST58* | 0.3 |  | *Ind2880* | 0 |  | *Ind2880* | 0.2 |  | *EST19* | 1.7 |  | *EST58* | 2.5 |  | *EST58* | 1.6 |
|  |  | *Ind2880* | 0.3 |  | *8604L* | 0 |  | *8604L* | 0.2 |  |  |  |  | *EST19* | 2.9 |  | *EST19* | 2.3 |
|  |  | *Ind457C* | 0.3 |  | *Ind457C* | 0 |  | *Ind457C* | 0.2 |  |  |  |  | *Ind457C* | 2.9 |  | *Ind457C* | 2.3 |
|  |  | *7655N* | 0.3 |  | *EST19* | 0.9 |  | *EST19* | 0.2 |  |  |  |  | *13066I* | 23.5 |  | *13066I* | 23 |
|  |  |  |  |  | *EST107* | 12.3 |  | *7655N* | 0.2 |  |  |  |  | *Ssosl85* | 28 |  | *Ssosl85* | 27 |
|  |  |  |  |  |  |  |  | *EST107* | 12.5 |  |  |  |  | *EST107* | 51 |  | *EST107* | 50 |
| 9 | AS-11 | *Sleel53* | 0 |  | *EST41* | 0 |  |  |  |  | *Ssa417UOS* | UN |  | *Sleei53* | 0 |  |  |  |
|  |  | *EST6* | 0 |  | *Omm1121* | 0 |  |  |  |  | *EST41* | UN |  | *Ssa417UOS* | 2.1 |  |  |  |
|  |  | *Omm1121* | 0 |  | *EST6* | 0 |  |  |  |  |  |  |  | *EST41* | UN |  |  |  |
|  |  | *16424E* | 0 |  | *SleeI53* | 0 |  |  |  |  |  |  |  |  |  |  |  |  |
|  |  | *Ssa417UOS* | 0.3 |  | *Ssa417UOS* | 0.9 |  |  |  |  |  |  |  |  |  |  |  |  |
|  |  | *EST41* | 1.5 |  |  |  |  |  |  |  |  |  |  |  |  |  |  |  |
| 10 | AS-12 | *Omm1070* | UN |  | *Omy272UOG* | 0 |  |  |  |  | *Omm1070* | 0 |  |  |  |  |  |  |
|  |  |  |  |  | *OmyRGT13TUF* | 2.5 |  |  |  |  | *Omy272UOG* | 18.5 |  |  |  |  |  |  |
| 11 | AS-13 | *9552C* | 0 |  | *9552C* | 0 |  | *9552C* | 0 |  | *Ind2679* | 0 |  | *Ssosl25* | 0 |  |  |  |
|  |  | *Ssa407* | 0.6 |  | *EST74* | 3.4 |  | *Ssa407* | 2 |  | *9552C* | 1.3 |  | *9552C* | 17 |  |  |  |
|  |  | *EST74* | 0.6 |  | *Ssa289* | 5.4 |  | *EST74* | 2 |  | *Ssa407* | 8.1 |  | *EST74* | 23 |  |  |  |
|  |  | *Ssa289* | 0.9 |  |  |  |  | *Ssa289* | 3.1 |  | *Oc18* | 26.6 |  | *Ssa289* | 29 |  |  |  |
|  |  | *Ssosl25* | 1.9 |  |  |  |  | *Ssosl25* | 4.2 |  |  |  |  |  |  |  |  |  |
| 12 | AS-14 |  |  |  | *2571c* | 0 |  |  |  |  | *2571c* | 0 |  | *2571c* | 0 |  |  |  |
|  |  |  |  |  | *BHMS311* | 8 |  |  |  |  |  |  |  | *BHMS311* | 0.8 |  |  |  |
| 13 | AS-15 | *BHMS386* | UN |  | *BHMS386* | 0 |  |  |  |  | *MHCI* | 0 |  | *BHMS386* | 0 |  |  |  |
|  |  |  |  |  | *MHCI* | 0.8 |  |  |  |  | *2273K* | 17.7 |  | *MHCI* | 11.3 |  |  |  |
|  |  |  |  |  | *2273K* | 1.3 |  |  |  |  |  |  |  |  |  |  |  |  |
| 14 | AS-18 | *EST138* | 0 |  | *EST138* | 0 |  | *EST138* | 0 |  | *EST138* | 0 |  | *EST138* | UN |  |  |  |
|  |  | *SSsp1605* | 16.3 |  | *SSsp1605* | 18.9 |  | *SSsp1605* | 17.6 |  | *SSsp1605* | 0 |  | *Ssa85* | UN |  |  |  |
|  |  | *Ssa85* | 39.7 |  | *Ssa85* | 29.2 |  | *Ssa85* | 34.5 |  | *One2ASC* | 3.6 |  |  |  |  |  |  |
|  |  | *1729I* | 39.7 |  |  |  |  | *1729I* | 34.5 |  | *Ssa85* | 26.1 |  |  |  |  |  |  |
| 15 | AS-19 |  |  |  |  |  |  |  |  |  |  |  |  | *BHMS365* | UN |  |  |  |
| 16 | AS-21 | *EST105* | 0 |  | *2889J* | UN |  |  |  |  | *EST105* | UN |  |  |  |  |  |  |
|  |  | *OmyRGT44TUF* | 0.3 |  |  |  |  |  |  |  | *2889J* | UN |  |  |  |  |  |  |
|  |  | *2889J* | 0.3 |  |  |  |  |  |  |  |  |  |  |  |  |  |  |  |
| 17 | AS-23 | *EST53* | 0 |  | *Ssa124* | 0 |  |  |  |  | *BHMS7-043* | 0 |  | *BHMS7-043* | UN |  |  |  |
|  |  | *SSf43* | 0.6 |  | *2456V* | 0 |  |  |  |  | *Ssa124* | 7.1 |  |  |  |  |  |  |
|  |  | *2456V* | 0.6 |  |  |  |  |  |  |  | *SSf43* | 8.1 |  |  |  |  |  |  |
|  |  |  |  |  |  |  |  |  |  |  | *2456V* | 12.7 |  |  |  |  |  |  |
| 18 | AS-24 | *Omy14INRA* | UN |  |  |  |  |  |  |  |  |  |  | *Omy14INRA* | UN |  |  |  |
| 19 | AS-25 | *2136E* | 0 |  | *2136E* | 0 |  | *2136E* | 0 |  | *2136E* | 0 |  | *2136E* | 0 |  |  |  |
|  |  | *Ssa4DIAS* | 1.6 |  | *Ssa4DIAS* | 0 |  | *Ssa4DIAS* | 0.8 |  |  |  |  | *Ssa4DIAS* | 0 |  |  |  |
|  |  | *4493F* | 1.6 |  | *Ssleer15.1* | 0 |  | *4493F* | 0.8 |  |  |  |  | *4493F* | 22.3 |  |  |  |
|  |  | *Ssleer15.1* | 1.6 |  |  |  |  | *Ssleer15.1* | 0.8 |  |  |  |  |  |  |  |  |  |
|  |  | *Omy1011UW* | 3.6 |  |  |  |  | *Omy1011UW* | 2.7 |  |  |  |  |  |  |  |  |  |
| 20 | AS-28 | *Omm1134* | 0 |  | *Ssa405* | UN |  |  |  |  | *Omm1134* | UN |  | *Sleen82* | 0 |  |  |  |
|  |  | *Omy7INRA* | 0 |  |  |  |  |  |  |  | *OMM1120* | UN |  | *Ssa405* | 5.1 |  |  |  |
|  |  | *Omm1107* | 0.3 |  |  |  |  |  |  |  |  |  |  | *Omy7INRA* | UN |  |  |  |
| 21 | AS-31 |  |  |  | *Ogo4* | UN |  |  |  |  | *Ogo4* | UN |  | *Ogo4* | UN |  |  |  |
| 22 | AS-32 | *Ssa419UOS* | 0 |  | *EST44* | 0 |  |  |  |  | *1445a* | UN |  | *Ssa419UOS* | 0 |  |  |  |
|  |  | *EST44* | 9 |  | *4955H* | 0 |  |  |  |  | *4246L* | UN |  | *1904X* | 4.2 |  |  |  |
|  |  | *1445a* | 9 |  |  |  |  |  |  |  |  |  |  | *1094G2* | 6.8 |  |  |  |
|  |  |  |  |  |  |  |  |  |  |  |  |  |  | *1445a* | 21.6 |  |  |  |
|  |  |  |  |  |  |  |  |  |  |  |  |  |  | *4246L* | 42.4 |  |  |  |
| 23 | AS-33 |  |  |  |  |  |  |  |  |  | *BHMS144* | UN |  | *Ssa224* | UN |  |  |  |
|  |  |  |  |  |  |  |  |  |  |  | *Ssa224* | UN |  |  |  |  |  |  |
| 24 | X3 |  |  |  | *3960I* | UN |  |  |  |  | *EST28* | 0 |  | *3960I* | UN |  |  |  |
|  |  |  |  |  |  |  |  |  |  |  | *3960I* | 4.9 |  |  |  |  |  |  |
| 25 | X5 | *EST46* | 0 |  | *1309C* | UN |  |  |  |  | *EST46* | 0 |  | *EST46* | 0 |  |  |  |
|  |  | *11953L* | 0 |  |  |  |  |  |  |  | *1309C* | 25.09 |  | *11953L* | 31.9 |  |  |  |
| 26 | X8 | *190S* | 0 |  | *EST127* | UN |  |  |  |  | *EST123* | 0 |  | *EST127* | UN |  |  |  |
|  |  | *8396P* | 17.5 |  |  |  |  |  |  |  | *EST127* | 2.9 |  |  |  |  |  |  |
|  |  | *EST127* | 18.9 |  |  |  |  |  |  |  |  |  |  |  |  |  |  |  |
| 27 | X9 | *EST101* | 0 |  | *Ind1921* | 0 |  | *EST101* | 0 |  | *Ind1921* | 0 |  | *Ind1921* | 0 |  | *Ind1921* | 0 |
|  |  | *Ind1921* | 0 |  | *4868M* | 0 |  | *Ind1921* | 0 |  | *EST103* | 20.3 |  | *EST103* | 15.5 |  | *EST103* | 18 |
|  |  | *4868M* | 0 |  | *EST103* | 0 |  | *4868M* | 0 |  |  |  |  |  |  |  |  |  |
|  |  | *1271X* | 0 |  |  |  |  | *1271X* | 0 |  |  |  |  |  |  |  |  |  |
|  |  | *EST103* | 0.3 |  |  |  |  | *EST103* | 0.15 |  |  |  |  |  |  |  |  |  |
| 28 | X10 | *EST11* | 0 |  | *EST11* | 0 |  | *EST11* | 0 |  | *EST11* | 0 |  | *EST11* | 0 |  | *EST11* | 0 |
|  |  | *7157K* | 4.5 |  | *Ssd30* | 0.7 |  | *Ssd30* | 1.4 |  | *7157K* | 8.4 |  | *7157K* | 7.3 |  | *7157K* | 7.9 |
|  |  |  |  |  | *7157K* | 1.5 |  | *7157K* | 3 |  |  |  |  |  |  |  |  |  |
| 29 | X11 | *EST40* | UN |  |  |  |  |  |  |  |  |  |  |  |  |  |  |  |
| 30 | X12 | *EST70* | UN |  |  |  |  |  |  |  | *EST70* | UN |  |  |  |  |  |  |
| 31 | X13 |  |  |  | *Ind2231* | UN |  |  |  |  |  |  |  | *Ind2231* | UN |  |  |  |
| 32 | X14 |  |  |  |  |  |  |  |  |  | *EST68* | UN |  | *EST68* | UN |  |  |  |
| 33 | X15 |  |  |  |  |  |  |  |  |  |  |  |  | *8229A* | UN |  |  |  |
| 34 | X16 |  |  |  |  |  |  |  |  |  |  |  |  | *OMM1400* | UN |  |  |  |
