# Supplementary figures and images for: Discovery and application of insertion-deletion (INDEL) polymorphisms for QTL mapping of early life-history traits in Atlantic salmon
Source: BMC Genomics. 2010 Mar 8;11:156. doi: 10.1186/1471-2164-11-156 (PMC2838853; doi:10.1186/1471-2164-11-156)

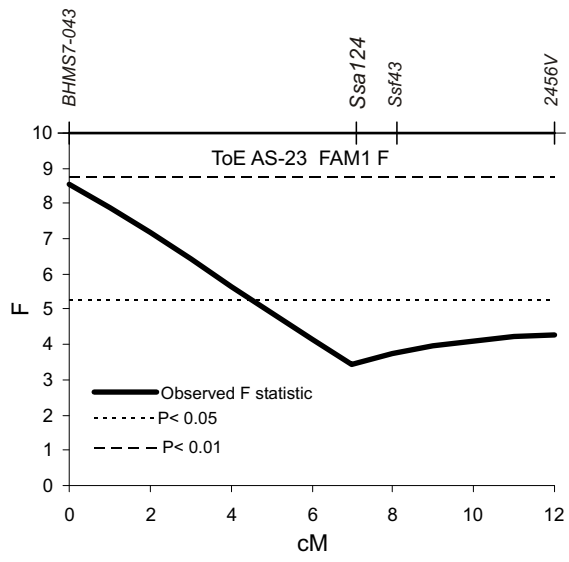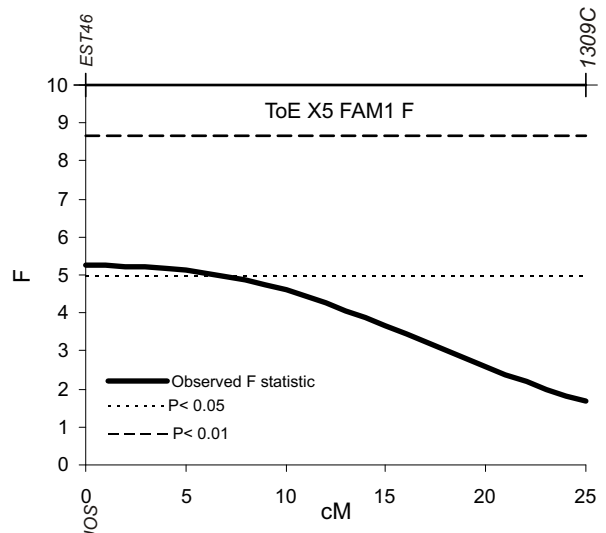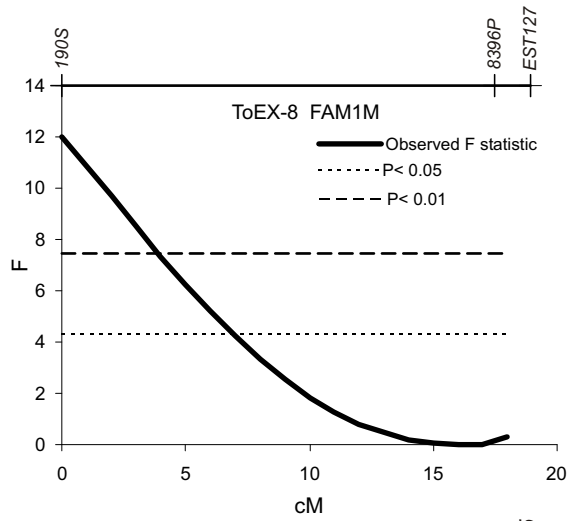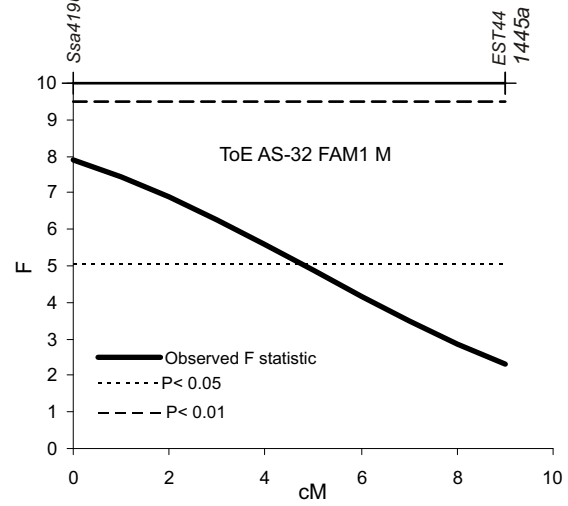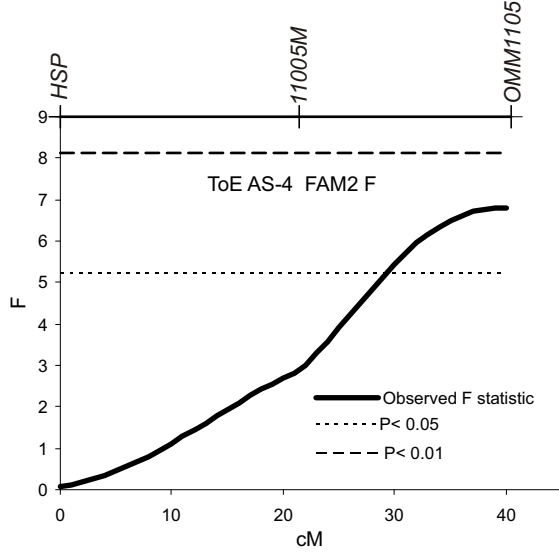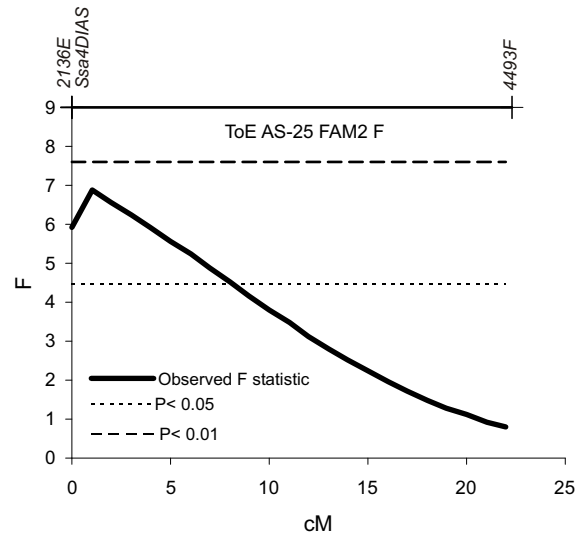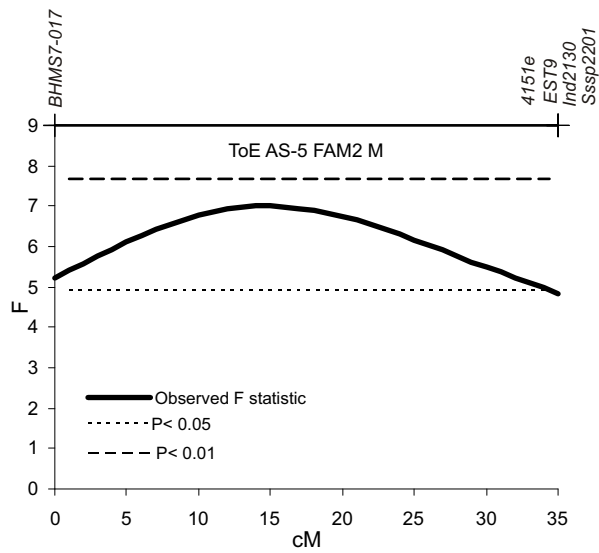

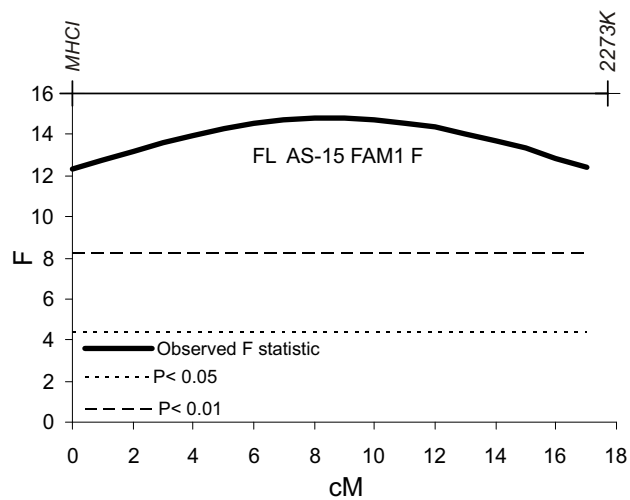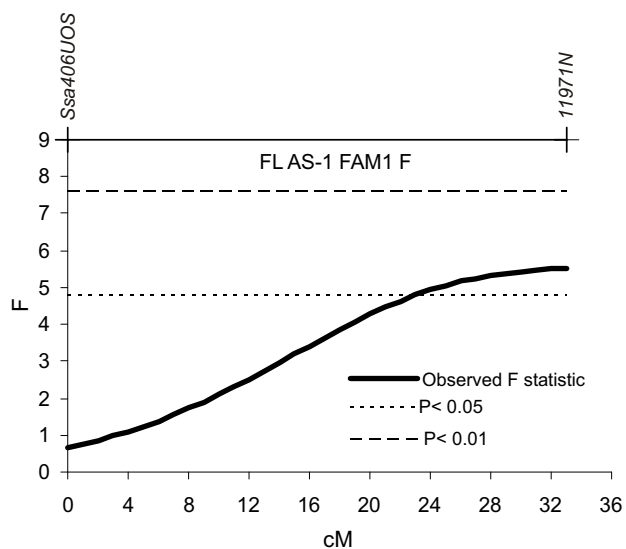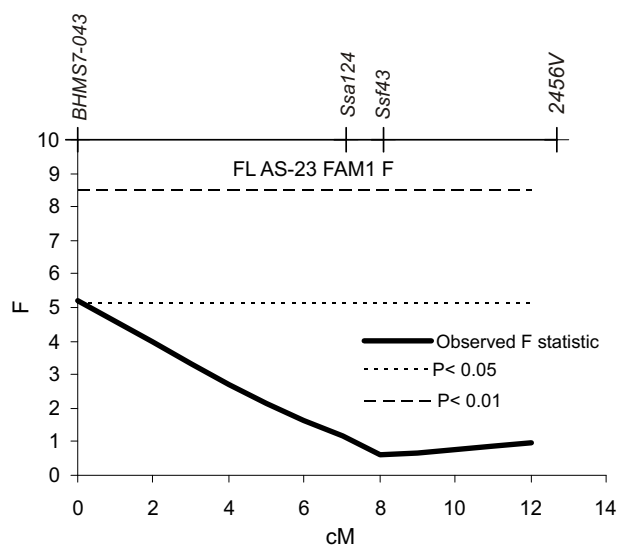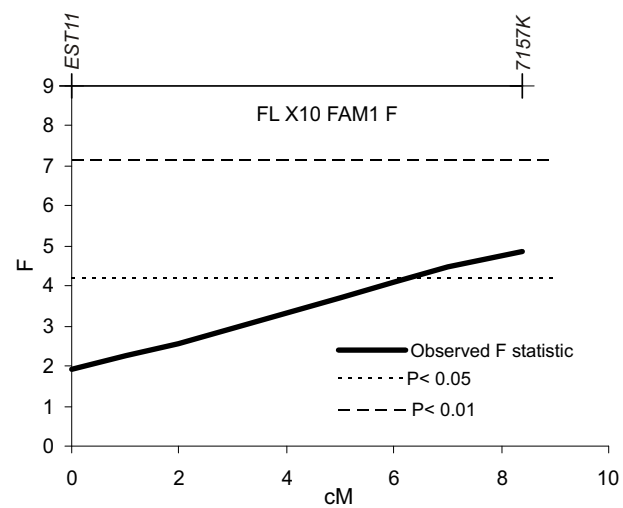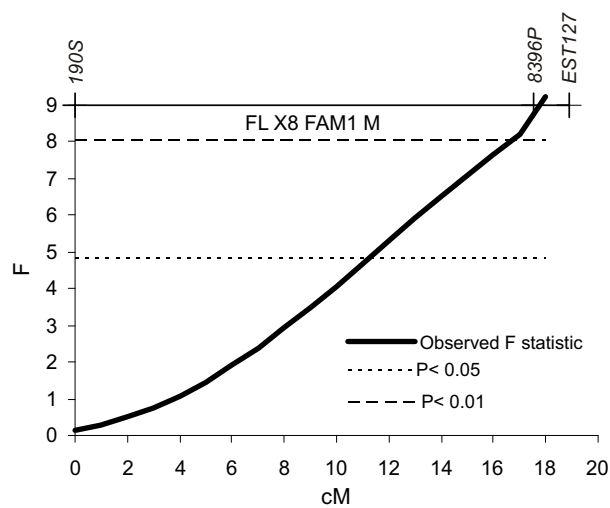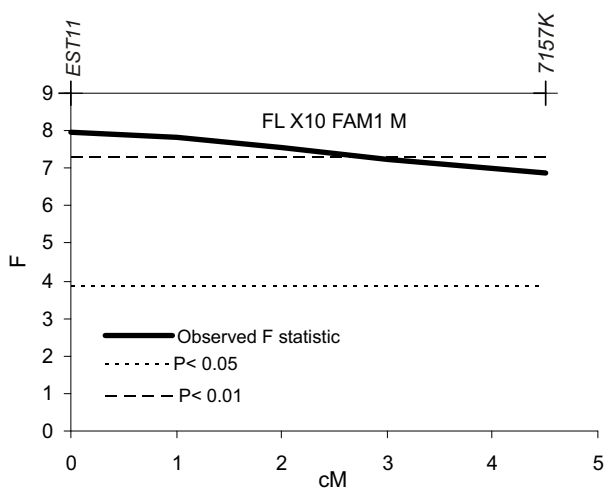

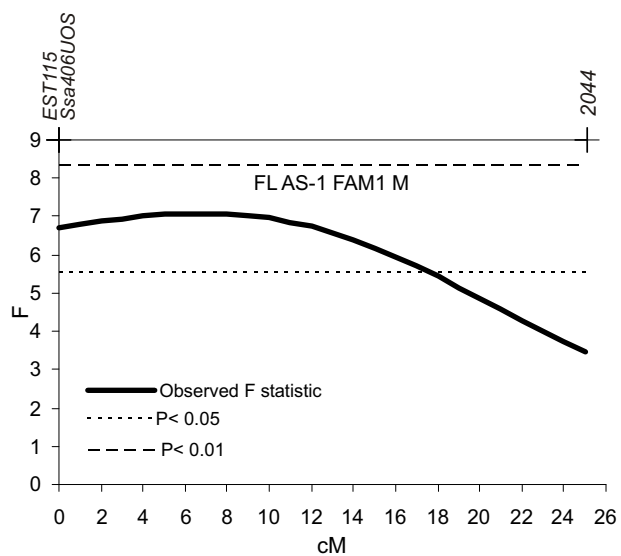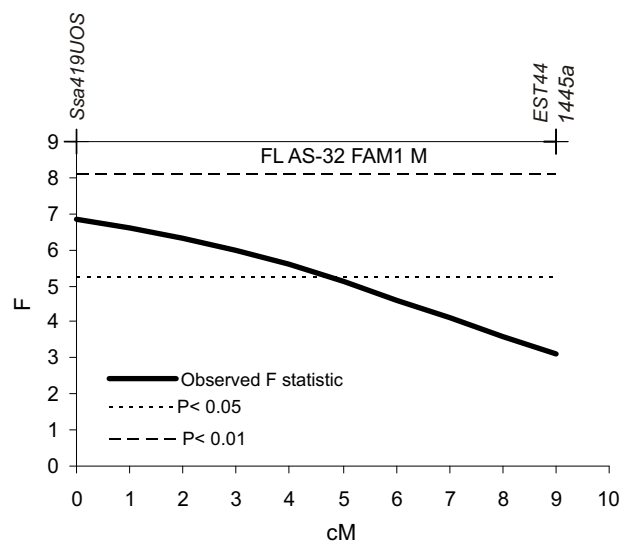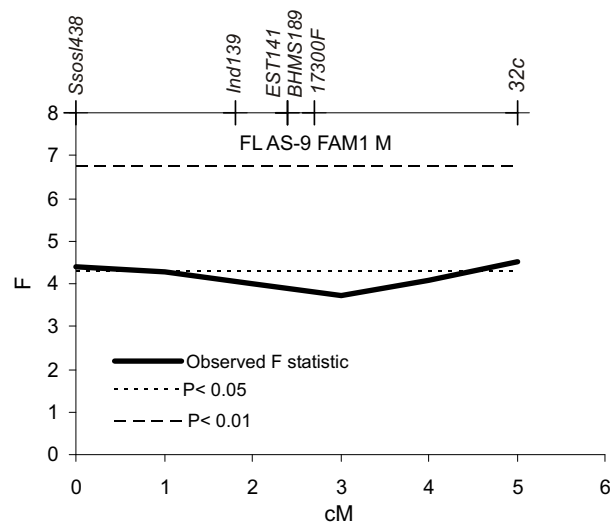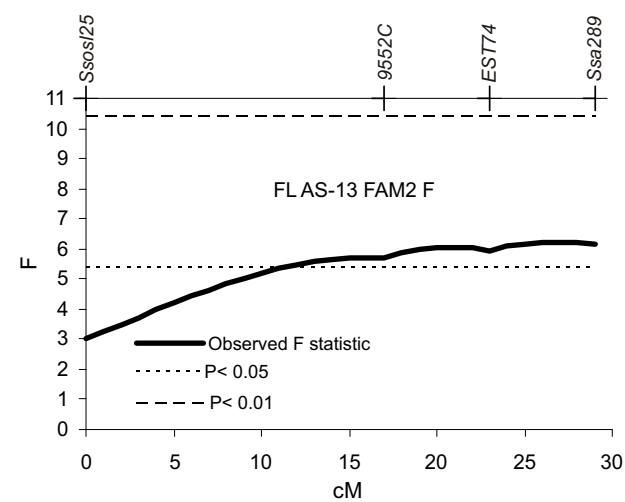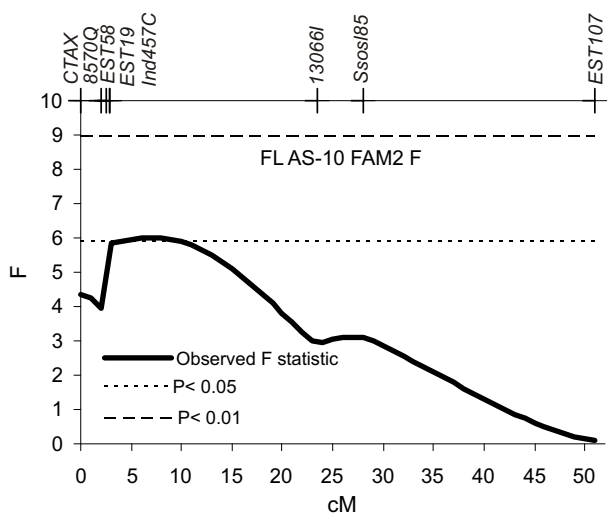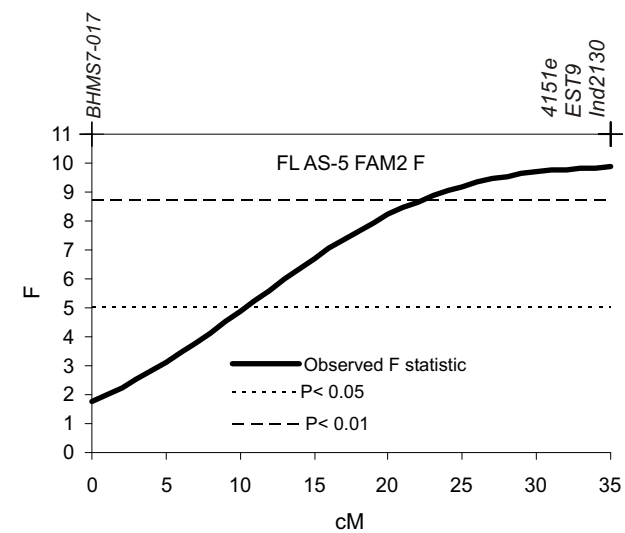

Supplement: Additional file 5 — Results from interval mapping using Haley-Knott regression in linkage groups larger than 5 cM. F = QTL Express F statistic; cM = Kosambi centi-Morgan. Marker positions are indicated at the top. Chromosome-wide permutation test significance thresholds (P < 0.05; P < 0.01) are indicated by dotted and dashed lines, respectively. [file 1471-2164-11-156-S5.PDF]
